# DNA-encoded library screening uncovers potent DNMT2 inhibitors targeting a cryptic allosteric binding site

**DOI:** 10.1016/j.isci.2025.113300

**Published:** 2025-08-05

**Authors:** Ariane F. Frey, Merlin Schwan, Annabelle C. Weldert, Valerie Kadenbach, Jürgen Kopp, Zarina Nidoieva, Robert A. Zimmermann, Lukas Gleue, Collin Zimmer, Marko Jörg, Kristina Friedland, Mark Helm, Irmgard Sinning, Fabian Barthels

**Affiliations:** 1 Institute of Pharmaceutical and Biomedical Sciences (IPBS), 55128 Mainz, Germany; 2 Heidelberg University Biochemistry Center (BZH), 69120 Heidelberg, Germany

**Keywords:** Biochemistry, Molecular Structure, Properties of biomolecules, Structural biology

## Abstract

DNMT2 (TRDMT1) is a human RNA methyltransferase implicated in various disease processes. However, small-molecule targeting of DNMT2 remains challenging due to poor selectivity and low cellular availability of known *S*-adenosylhomocysteine (SAH)-derived ligands. In this study, a DNA-encoded library (DEL) screen identified five non-SAH-like chemotypes that selectively bind DNMT2, including three peptidomimetics. Orthogonal assays confirmed target engagement, and X-ray crystallography revealed a previously unknown allosteric binding pocket formed via active site loop rearrangement. Guided by structural insights, the authors optimized a lead compound with a *K*_D_ of 3.04 μM that reduces m^5^C levels in MOLM-13 tRNA and synergizes with doxorubicin to impair cell viability. These inhibitors exhibit unprecedented selectivity over other methyltransferases, offering a promising scaffold for future DNMT2-targeting therapeutics. Beyond pharmacological implications, the study provides conceptual advances in understanding allosteric modulation and structural plasticity of DNMT2.

## Highlights

• DEL screening identifies non-SAH-like, selective DNMT2 inhibitor chemotypes
• Lead compound binds allosterically to a cryptic pocket revealed by X-ray crystallography
• DNMT2 inhibitors reduce m^5^C levels and enhance doxorubicin-induced cytotoxicity
• Structural insights enable selective targeting of DNMT2 over related methyltransferases

## Graphical abstract

**Figure:**
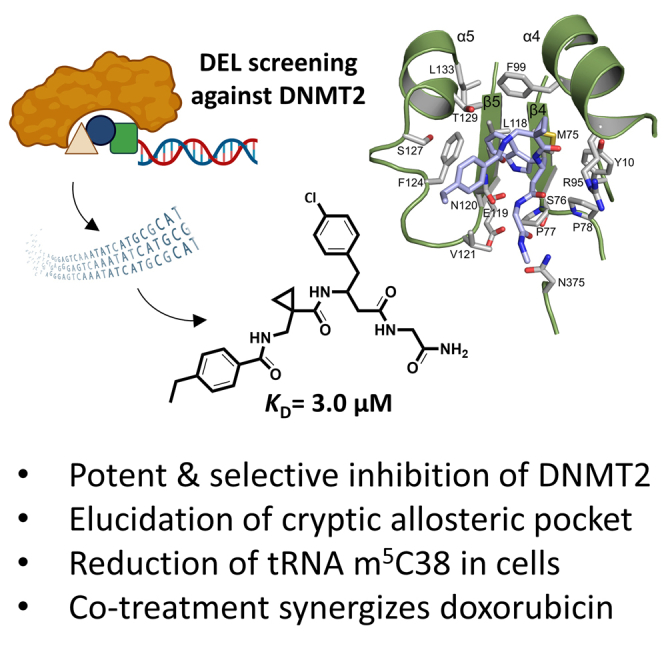

## Introduction

Targeting RNA methyltransferases (MTases) by active site-competitive small-molecule drugs proves challenging due to their universal dependence on *S*-adenosyl-l-methionine (SAM) as a methyl group donating substrate, which is transformed into *S*-adenosylhomocysteine (SAH) during this enzymatic reaction.^1^^,^^2^ SAH itself presents a pan methyltransferases inhibitor used as a starting point for several RNA MTase drug discovery campaigns.^3^^,^^4^^,^^5^ A unique representative of this enzyme family is the RNA MTase DNA methyltransferase 2 (DNMT2), also known as TRDMT1, which is structurally related to other DNMTs, but this paralogous enzyme deviates in function and cellular localization.^6^^,^^7^^,^^8^ Instead of DNA, which is the main target of DNMT1 and DNMT3, DNMT2 introduces the m^5^C38 modification in several tRNAs.^6^^,^^9^ Furthermore, DNMT2 is controversially discussed to be found in both nuclear compartments and the cytoplasm, hinting at its diverse roles and influence in protein biosynthesis.^9^^,^^10^^,^^11^ Its distinctive catalytic MTase domain, featuring the unique cysteine-phenylalanine-threonine (CFT) motif, underscores its evolutionary and functional uniqueness within the DNMT family.^11^ Physiologically, DNMT2 targets various tRNAs, including tRNA^Gly^, tRNA^Val^, and tRNA^Asp^.^9^^,^^12^ tRNA methylation by DNMT2 plays a role in regulating RNA degradation by ribonucleases, thereby enhancing tRNA stability and overall protein expression.^9^^,^^13^ Additionally, tRNA m^5^C38 methylation improves codon recognition, particularly distinguishing between aspartate and glutamate, thus enhancing translational accuracy.^14^ Given DNMT2’s role in regulating protein expression and translational accuracy, it is not surprising that DNMT2 misregulation has been implicated in cancer pathogenesis.^15^ DNMT2 seems to play a functional role in various cancer cell lines and tumorigenesis, such as cervical or bladder tissue, and leukemia.^15^^,^^16^^,^^17^ Additionally, DNMT2 silencing was found to correlate with miRNA upregulation which is also related to proliferation and function as tumor suppressor.^18^ Also, elevated m^5^C levels in small non-coding RNA (sncRNA), introduced by DNMT2, have been linked to metabolic disorders, where these influence the disorders’ inheritance induced by high-fat diets in mice.^19^ During oxidative stress, a DNMT2 upregulation could be detected, leading to the assumption that DNMT2 also helps cells cope with exogenous stress factors, underscoring DNMT2’s multifaceted involvement in disease mechanisms.^20^

Despite its association with various disease mechanisms, DNMT2 remains a challenging target for drug development.^21^ This is primarily due to the abundance of SAH and SAM and their ubiquitous binding proteins in cells with which inhibitors must compete.^22^^,^^23^^,^^24^ Moreover, the fact that over 200 human MTases depend on SAM further complicates the discovery of selective inhibitors.^25^ Efforts to develop inhibitors targeting DNMT2 have predominantly focused on SAH analogs, which have achieved on-target potency in the micromolar range, but all fail to demonstrate selectivity within the DNMT or NSUN family MTases.^1^^,^^3^^,^^4^ Currently available inhibitors also failed to inhibit DNMT2 within cellular assays, probably due to the low permeability of zwitterionic SAH derivates.^3^ The generation of first-in-class selective and cell-active DNMT2 inhibitors is a current requirement to further investigate the function and druggability of DNMT2.

To address the development of cellularly potent DNMT2 inhibitors, we have applied the DNA-encoded library (DEL) screening technology on DNMT2 to identify modulating small molecule chemotypes. The goal was to develop selective chemical tools for DNMT2 investigation, modulation, and inhibition. DEL have gained widespread popularity in both industry and academia as a powerful tool for drug-like hit generation.^26^^,^^27^^,^^28^ Comprising unique molecular structures tagged with DNA barcodes, DEL enables efficient diverse compound library screening and groundbreaking identification of novel drug scaffolds.^29^^,^^30^^,^^31^^,^^32^ Since 1992, the DEL technology has undergone continuous development, evolving from initial strategies based on amide bond formation to widely used reactions such as metal-catalyzed cross-couplings, heterocycle synthesis, Diels-Alder cycloaddition, nucleophilic aromatic substitution, and reductions.^32^^,^^33^ More recently, the methodology has expanded to include advanced transformations like photo-promoted reactions, C–H activation, and functionalization, thereby enabling access to a broad and diverse chemical space.^33^ Recently, Wuxi AppTec enabled academic users to open access DEL by providing a DELopen platform containing a library of 4.2 billion unique compounds.^34^ Latest DELopen successes include the ligand discovery for diverse proteins such as thrombin, RNase L, E3 ligases*,* the transcription factor TEAD, and antibacterial drug targets, highlighting its broad utility across various target classes.^30^^,^^35^^,^^36^^,^^37^^,^^38^^,^^39^

## Results and discussion

### DELopen screening reveals DNMT2 ligand chemotypes

**Figure 1:**
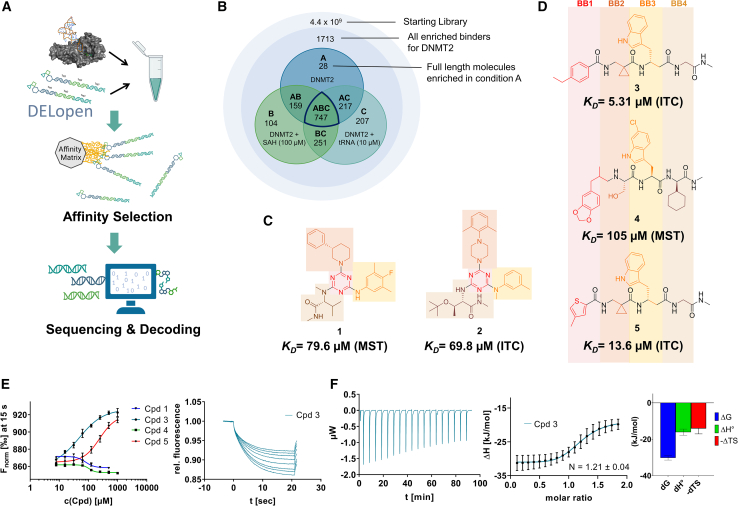
DEL screening reveals DNMT2 ligand chemotypes & biophysical characterization (A) DELopen screening workflow. DELopen libraries consist of 4.2B compounds encoded by individual DNA barcodes. During affinity selection, small molecule-DNA conjugates are enriched, while non-binders are washed away. (B) Conditions used during the DELopen screening: In condition A, native DNMT2 without additives was used; condition B: DNMT2 and SAH (100 μM) were mixed to block the SAH binding site; condition C: DNMT2 was incubated with tRNA^Asp^ (10 μM). Most full-length molecules were enriched in condition ABC (unaffected by competitors), from which 5 selected compounds **1**–**5** were chosen for off-DNA synthesis and downstream characterization. (C) Enriched triazine-based compounds selected from ABC. (D) Peptidomimetic compounds enriched in ABC and chosen for further study. Building blocks BB1–BB4 are highlighted accordingly. (E) MST dose-response curves for compounds **1**, **3**, **4**, and **5** with detailed MST traces for compound **3**. (F) ITC data of compound **3** titrated to DNMT2. From left to right: Titration thermogram, stoichiometry plot, and signature plot. Data are represented as mean ± SEM. A comprehensive overview of the biophysical data for all compounds binding to DNMT2 can be found in the SI.

Recombinant human DNMT2 was screened against the fourth-generation DELopen (WuXi AppTec) library (4.2 billion compounds, 27 sub-libraries) to identify small molecule RNA methyltransferase ligands (Figure 1A). DEL pannings were carried out with His-tagged DNMT2 protein stably immobilized on nickel chelate (Tris-NTA) affinity resin (Figure S1B). Selection screens against Tris-NTA resin without protein loading served as a nonspecific binding control. Three successive panning rounds were conducted, while the second round was chosen for deep sequencing based on the total retained DNA conjugates (Figures S1A and S1E). Sequenced hits were decoded and filtered to remove commonly encountered false positives and nonspecific binders to the affinity resin.^40^ After affinity enrichment, 1713 unique full-length molecules were identified across three positive selection conditions and their combinations: (**A**) native DNMT2 without additives (*n* = 28 compounds); (**B**) DNMT2 with SAH (competitor, 100 μM, *n* = 104 compounds); (**C**) DNMT2 with tRNA^Asp^ (competitor, 10 μM, *n* = 207 compounds); (**AB**) *n* = 159 compounds, (**AC**) *n* = 217 compounds, (**BC**) *n* = 251 compounds, (**ABC**) *n* = 747 compounds (Figure 1B).

These post-DEL screening compounds had enrichment scores of at least 100 for DNMT2. The enrichment score was calculated by decoding the DNA tags from next-generation sequencing (NGS) results, considering the corrected copy number, library size, sequencing depth, and other normalization factors (see https://hits.wuxiapptec.com/delopen).^41^ A molecule with an enrichment score of 100 is a hundred times more abundant in the sequencing data than the average molecule in the library. Interestingly, DEL sequencing analysis showed that most of the compounds enriched on DNMT2 were found within the condition **ABC**, which may reflect the inability of the low-affinity SAH competitor to completely occlude the active binding site during the panning. The five compounds with the highest confidence (enrichment scores >200 000 and copy number >10, Table S5) and structural diversity (from three sub-libraries in total, see Figure S1F) were selected for further investigation and downstream validation studies (Figures 1C and 1D).

Across the 747 **ABC**-enriched and the five highest-scoring representative molecules (“hits”), recurring patterns were apparent in each of the four building block positions (denoted BB1, BB2, BB3, and BB4), indicating structurally related families: two triazine-based DEL hits (**1**–**2**) and three peptide-based DEL hits (**3**–**5**). Hit molecules **3**–**5** can be described as β-homo-tripeptides with non-natural side chain groups in building block positions BB2 resp. BB3 and incorporating an N-terminal substituent in BB1. The BB4 C-terminus (corresponding to the DNA tag attachment position) was synthetically amidated with methylamine. These hit compounds were resynthesized without the encoding DNA tag and were studied for DNMT2 active site binding using an MST displacement assay as previously described by Zimmermann et al.^42^ For the thermophoretic detection, 5-FAM-triazolyl-adenosyl-diaminobutyric acid (FTAD) was used as a fluorescent probe, which can bind to the DNMT2 SAH binding pocket.^42^ When FTAD is bound to DNMT2 versus its unbound state, there is a measurable difference in the F_norm_ value represented by a distinct positive MST shift, and thus, a ligand that can displace the FTAD-DNMT2 interaction can be detected by competitive MST screening assays (Figures S2 and S3).

In this regard, two of the five tested DEL hit compounds, **3** and **5**, induced a quantitative FTAD displacement in the MST assay (Figure 1E). In subsequent dose-response experiments, **3** and **5** showed *K*_*D*_-values of 48.2 μM and 241 μM, respectively (detailed information in Table 1). Interestingly, **3** and **5** are similar in structure, differing only by the first building block, 4-ethylbenzoic acid for **3** and 4-methylthiophene-2-carboxylic acid for **5** (Figure 1D), while compound **4** features a similar peptidomimetic structure but without β-homo amino acids. In contrast, **1** and **2** share a common triazine core structure due to their origin from the same sub-library (Figure 1C). Notably, compounds **1** and **4** did not lead to FTAD displacement but rather induced a negative MST shift toward a higher FTAD-bound degree thermophoresis resp. strengthened interaction. We speculated about an allosteric binding mode of these compounds, which agrees with the observation that these compounds were identified in the **ABC** panning cluster.

**Table 1:** Biophysical data of compounds 1–5 determined by various ligand binding and enzyme inhibition experiments

	^3^H assay (IC_50_)	MST (*K*_*D*_)	ITC (*K*_*D*_)
1	n.i.	79.6 ± 10.0 μM	n.b.
2	n.i.	n.b.	69.8 ± 5.4 μM
3	38.9 ± 6.9 μM	48.2 ± 8.0 μM	5.31 ± 1.67 μM
4	44.4 ± 12.9 μM	105 ± 9 μM	n.b.
5	236 ± 63 μM	241 ± 36 μM	13.6 ± 1.6 μM

All results include the mean value and standard deviations from at least technical triplicate measurements. Raw data and analysis plots are depicted in Figure 1 and Figures S2–S7. n.i. = no inhibition/less than 50% inhibition at 500 μM//n.b. = no enthalpy signal observable (ITC)/no thermophoresis shift observable (MST).

As an orthogonal biophysical binding experiment, we implemented isothermal titration calorimetry (ITC) ligand binding studies. In these experiments, **2**, **3**, and **5** showed significant binding enthalpy signals when titrated to DNMT2, with *K*_*D*_-values of 69.8, 5.3, and 13.6 μM, respectively (Figure 1F; Figure S4). Of note, peptidomimetic hits **3** & **5** resulted in particularly long injection equilibration times in the minute magnitude, and thus, we speculated about a conformational change upon ligand binding, which could be confirmed by subsequent crystallography as described below. Interestingly, the thermodynamic profile showed that these hits had different enthalpy and entropy contributions despite their comparable affinity. During the ligand optimization from hit compound **3** to lead structure **16** (see below), this enthalpy contribution to the overall binding affinity increased continuously (Figure 5A). Additionally, an orthogonal and label-free affinity selection-mass spectrometry (AS-MS) assay for top-hit compound **3** was conducted (Figure S6), revealing a *K*_*D*_-value of 8.40 ± 2.20 μM, which is in agreement with the ITC data.^43^ Besides biophysical characterization utilizing DNMT2 binding assays, all five DEL hits were evaluated regarding their inhibitory effects using an enzymatic ^3^H-incorporation assay, where the tritium-labeled methyl group incorporation from ^3^H-SAM to the substrate tRNA^Asp^ can be determined.^3^^,^^4^ Compounds **3**, **4**, and **5** were able to inhibit DNMT2’s MTase activity with IC_50_-values of 38.9, 44.4, and 236 μM, respectively (Figure S7). Data and figures of additional ligand characterization experiments can be found in Figures S2–S7.

Taken together with the data gleaned from the DEL sequencing results, the combined ligand characterization data suggest that the DEL hits identified during this screening campaign are not binding to the DNMT2’s active site nor tRNA pockets because hit compounds were enriched in condition **ABC** and show only partly competitive behavior during ligand binding experiments. The experimental findings from MST, ITC, and enzymatic ^3^H-incorporation assay suggest that triazine compounds **1** and **2** act as non-competitive DNMT2 binders without inhibition potency, whereas β-homo-tripeptides **3**–**5** function as DNMT2 enzyme inhibitors. The mode of action resp. their binding site remained elusive from the biophysical investigations but could be finally solved by DNMT2 crystallography (vide infra). We speculated that the minor discrepancies between the enrichment scores’ relative order and determined affinities may be due to the DNA tag sequence absence in the off-DNA synthesized hit compounds.

### Development of fluorescent tracer molecules and binding site investigation

**Figure 2:**
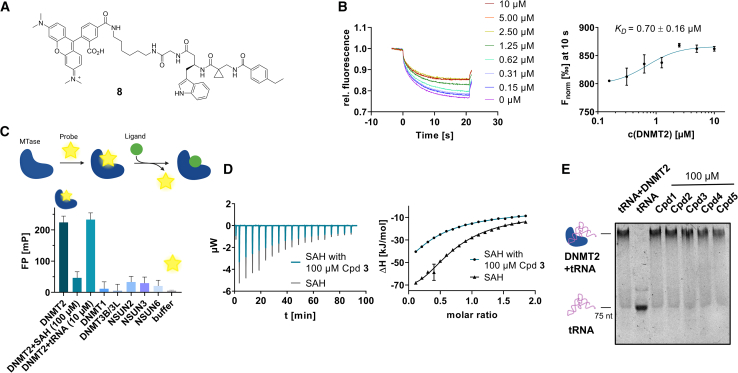
Investigating the ligand binding mode and selectivity with biophysical methods (A) Chemical structures of probe **8**: TAMRA-labeled β-homo-tripeptide. (B) MST dose-response curves for probe **8** binding to DNMT2. (C) Fluorescence polarization assay for probe **6** binding to native DNMT2 (2 μM), DNMT2 (2 μM) preincubated with SAH (100 μM), DNMT2 (2 μM) with tRNA^Asp^ (10 μM), and various related MTase off-targets (all 2 μM). (D) DNMT2-SAH ITC displacement experiment for compound **3**. SAH was titrated to DNMT2 in the absence resp. presence of compound **3** (100 μM), yielding a significant enthalpy reduction. (E) EMSA assay investigates the competition of hit compounds **1**–**5** with the tRNA substrate. Positive control: tRNA+DNMT2 (band at high molecular weights); negative control: only tRNA (band at low molecular weights). Hit compounds **1**–**5** (100 μM) were added to the DNMT2-tRNA complex (10 + 5 μM) and analyzed by native PAGE. No compound was able to dissociate the DNMT2-tRNA complex. Data are represented as mean ± SEM. Uncropped PAGE images can be found in Figure S22.

For further characterization of both triazine- and tripeptide-based DEL hits, fluorophore functionalized tracer compounds **6**–**8** were developed based on their parent compounds for target selectivity determination in fluorescence-based assays and as molecular probes for further investigations (Figure 2A; Figure S3). Three fluorescent probes were designed during this study for both identified DEL chemotypes: Alexa Fluor 488 (AF488) resp. tetramethylrhodamine (TAMRA) labeled β-homo-tripeptides (**6** and **8**), and a Cy5-labeled triazine-based probe (**7**). Apparent binding affinities of fluorescent probes were measured by MST experiments as described above with the modification that the respective probe was used instead of FTAD (Figure 2B; Figures S3E and S3F).^44^ By this, probe binding affinities (**6**: 1.65 μM, **7**: 1.36 μM, and **8**: 0.70 μM) were determined which resemble the original DEL hit compounds’ magnitude of potency, suggesting that the chosen fluorophores do not significantly interfere with their binding behavior and the probes are suitable for fluorometric MTase investigations.

Next, peptidomimetic probe **6** was utilized to determine MTase binding selectivity toward related DNMT resp. NSUN proteins and to investigate the competitivity of natural DNMT2 substrates (SAH and tRNA^Asp^) by fluorescence polarization (FP) displacement experiments (Figure 2C). Fluorometric assays using DNMT2 revealed that the compound **6**-DNMT2 complex yields high fluorescence polarization (220 mP) compared to enzyme-free buffer conditions (5 mP). By examining whether this effect can be reversed by the addition of natural ligands, we found that SAH (100 μM) is able to displace probe **6**, while tRNA^Asp^ (10 μM) does not show a significant effect, and high fluorescence polarization is retained during the tRNA substrate addition. These results were furthermore confirmed by an ITC displacement experiment, where **3** was able to displace SAH and vice versa (Figure 2D; Figure S5).^45^ Interestingly, compound **3** appears to be partly competitive with SAH, although it behaves seemingly non-competitive in the DEL screening. We hypothesize that this discrepancy is due to the low affinity of SAH for DNMT2, and SAH is not retained in DNMT2-bound form during stringent washing steps to remove unspecific DEL-binders.

Probe **6** also enabled the determination of ligand binding selectivity toward other m^5^C MTases using the FP assay setup. Since NSUN2, NSUN3, and NSUN6 introduce m^5^C in other tRNA and DNMT1 resp. DNMT3A/3L in DNA, these enzymes were selected for the ligand selectivity profile determination. By incubating probe **6** with various related m^5^C MTases, it was observed that this probe does not bind to related NSUN and DNMT families’ MTase representatives at concentrations up to 20 μM, highlighting its unique DNMT2 binding selectivity (Figure 2C; Figure S3H). Due to the highest potency and excellent selectivity of hit compound **3**, we decided to conduct the following characterizations and further developments using this molecule as a starting point.

To investigate the competition of DEL hit compounds with the tRNA^Asp^ substrate, we conducted an electrophoretic mobility shift assay (EMSA) to analyze the DNMT2-tRNA complex. Here, we found that none of the 5 hit compounds (100 μM) had an influence on the DNMT2-tRNA complex formation (Figure 2E).^46^ Analogous MST displacement experiments with *in situ* fluorescently labeled tRNA^Asp^ showed consistently no compound was able to dissociate the nucleic acid-protein complex formed by DNMT2 and tRNA^Asp^ (*K*_*D*_ = 218 nM, Figures S8A and S8B). Furthermore, no compound (100 μM) had a significant influence on the thermophoresis of tRNA^Asp^ in a protein-free solution (Figures S8C and S8D).^47^ This finding is supported by our crystal structure of compound **3** with DNMT2 (vide infra). The previously elucidated RNA-binding residues (K122, R160, R162, R275, R289, K295, K367, and R371; mutagenesis study of Jurkowski et al., 2012) are in significant distance to the compound **3** binding pocket, and thus, allow the simultaneous binding of compound **3** and tRNA.^48^ The exact binding mode of tRNA^Asp^ to DNMT2 could not be elucidated by crystallography until now, therefore, we have created an Alphafold model illustrating the ternary complex of the SAH resp. compound **3**, and tRNA^Asp^ (Figure S20).

### Crystallography unravels an allosteric binding pocket of hit compound 3

For our crystallographic studies, we used a deletion construct of human DNMT2, dubbed DNMT2Δ47, in which residues 191 to 237 were deleted since this region is predicted as intrinsically disordered. A similar construct was successfully used for the only available X-ray structure of human DNMT2 bound to SAH (PDB 1G55^49^). We found that the deletion mutant’s catalytical activity is indistinguishable from the full-length wild-type enzyme (Figure S7C), and that hit compound **3** displays medium-range micromolar affinity (48 vs. 102 μM) against both wild-type and DNMT2Δ47 enzymes (Figure 1E vs; Figure S3G).

**Figure 3:**
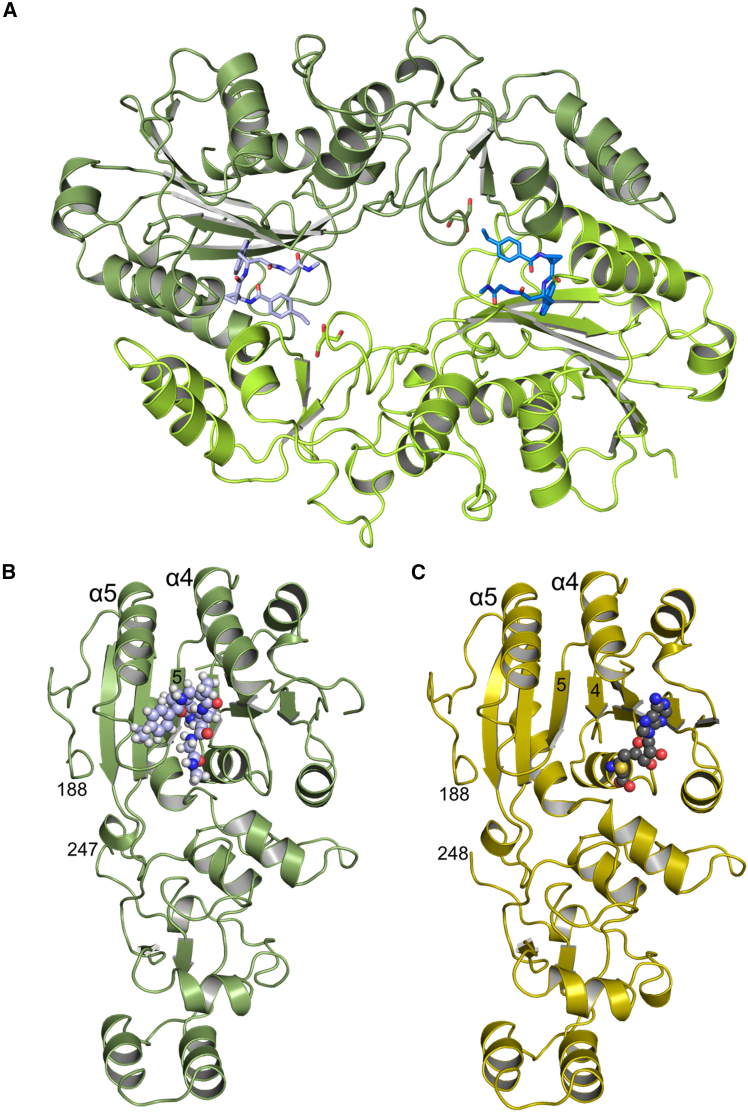
Crystallography unravels an allosteric binding pocket of hit compound 3 (A) X-ray structure of DNMT2Δ47 showing both molecules of the asymmetric unit. Bound compound **3** molecules are represented as sticks. Chain A is shown in green/light blue and chain B in light green/blue. (B) X-ray structure of DNMT2Δ47-compound **3** chain A depicted as cartoon (green) with compound **3** as spheres (carbon atoms in light blue). (C) The crystal structure of human DNMT2Δ47 (PDB 1G55) as cartoon (yellow) with bound SAH as spheres (carbon atoms in gray) is given for comparison.

We could obtain an X-ray structure of human DNMT2Δ47 with bound compound **3** at 2.6 Å resolution, which shows an overall fold similar to DNMT2Δ47-SAH (PDB 1G55) as indicated by an overall rmsd (C_α_) of 0.69 Å (Figure S17A). The asymmetric unit contains two very similar DNMT2Δ47-compound **3** complexes with an overall rmsd(C_α_) of 0.354 Å and the largest differences in the loop region comprising residues 82 to 95 (Figure 3A). A large common surface area of 1685 Å^2^ is present between these two protein complexes. Interface and packing analysis with PISA^50^ supports a stable dimer formed by the two complexes in the asymmetric unit. Therefore, we used analytical size exclusion chromatography to investigate the oligomeric state of DNMT2Δ47-compound **3** in solution. We could show that DNMT2Δ47 with bound compound **3** is indeed dimeric in solution at a high protein concentration of 10 mg/mL (Figure S18), which was also used for crystallization. In contrast, DNMT2Δ47 without ligand is monomeric in solution under the same conditions.

**Figure 4:**
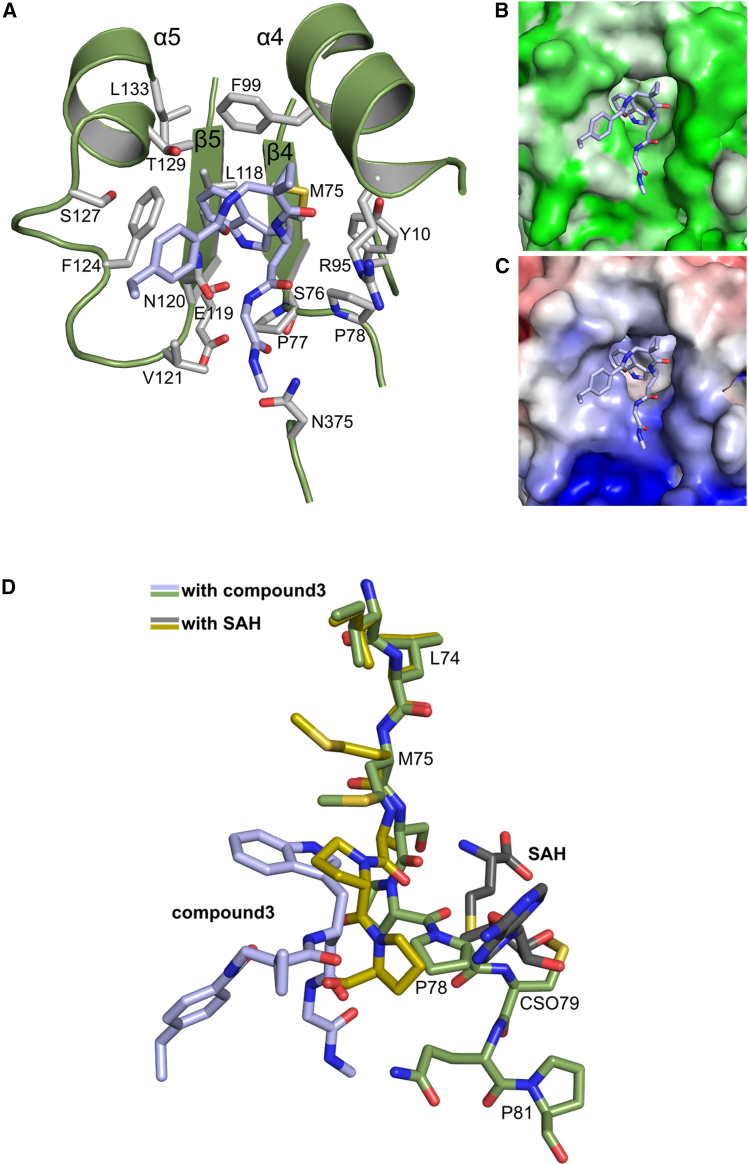
Ligand-protein interactions of compound **3** (A–C) (A) A detailed view of compound **3** (carbon atoms in light blue) bound to DNMT2Δ47 chain A (green cartoon) with interacting protein side chains in light gray. Side chains of chain B are omitted for clarity. Molecular surface representation of the binding site with compound **3** colored by (B) hydrophobicity (white -> green = high -> low) and (C) electrostatic potential from −5 kT/e (red) to +5 kT/e (blue). (D) Superposition of compound **3**- & SAH-binding protein segments. Local superposition onto the central β-sheet in the large domain of DNMT2Δ47-compound **3** (green/light blue) with DNMT2Δ47-SAH (yellow/gray) showing the end of β4 and following residues with respective ligands. Residues P77, P78, and C79 are shifted toward the SAH binding site upon compound **3** binding.

Both complexes in the asymmetric unit show clear electron density for compound **3** (Figure S17C). Most interestingly, compound **3** is bound in a largely hydrophobic pocket adjacent to the active site (Figure 3B). For comparison, the structure with SAH bound in the active site is depicted in Figure 3C, and superpositions of the two different complexes without and with ligands are given in Figures S17A and S17B. Compound **3** is bound by residues mainly from the two regions β4-α4 and β5-α5, which is depicted in Figure 4A for chain A (interacting residues of chain B have been omitted for clarity).

A comprehensive schematic ligand-protein interaction diagram was created with LigPlot+^51^ for both DNMT2Δ47-compound **3** complexes in the asymmetric unit (Figure S19). In the following, a detailed description of compound **3** binding to chain A and the differences between both complexes in the asymmetric unit are given. The indole ring of compound **3** is located in a hydrophobic cave formed by Y10, M75, backbone of S76, P77, F99, L118, F124, and L133. Additionally, the amine group of the indole ring forms a hydrogen bond with the backbone carbonyl of E119. The ethylbenzene moiety of compound **3** is bound between V121, F124, S127, T129 and L279_B_. The ethyl group is also in contact with a glycerol molecule (used for cryo-protection) bound in the dimer interface. The amide group, which connects the ethylbenzene and cyclopropane moieties, is in van der Waals contact with P78 and R95 and forms three hydrogen bonds, namely with the main chain carbonyl of L278_B_, with the side chain of N120, and an intramolecular H-bond to the amino group connecting the cyclopropane and indole moieties. The cyclopropane moiety is surrounded by the hydrophobic side chains of F99, L278_B_, and L279_B_. Finally, the two terminal amide groups of compound **3** form hydrogen bonds with the side chains of N120 and N375. Due to different side chain conformations in chain B, R95_B_ forms an additional hydrogen bond to this part of compound **3**_B_, and N281_A_ and E302_A_ are in non-polar contact with compound **3**_B_. The molecular surface representations of the ligand binding site colored by hydrophobicity (Figure 4B) and electrostatic potential (Figure 4C) show a clear correlation with the properties of the individual moieties of the bound ligand, e.g., the binding of the ethylbenzene moiety to a hydrophobic patch and the interaction of the carbonyl groups with a highly positive patch.

In order to analyze the structural rearrangements induced by compound **3** upon binding to DNMT2Δ47, we created a local superposition with DNMT2Δ47-SAH onto the central β-sheet of the large domain, namely onto β3 to β7. As depicted in Figure 4D, the largest local movement upon binding of compound **3** is observed in the loop following β4 (M75 to P78), where P77 and P78 are rotated and shifted toward the SAH binding site. Additionally, subsequent residues could be built in both complexes with compound **3** (A: C79–F82; B: C79–I87), whereas in DNMT2Δ47-SAH, residues 79 to 96 are not resolved. Binding of compound **3** shifts residues into the SAH binding pocket. Moreover, the active site residue C79 is now resolved and moved into the SAH binding region. The superposition depicted in Figure 4D shows that binding of compound **3** narrows the active site, which prohibits SAH binding due to steric hindrance by P77, P78, and C79. As a consequence, compound **3** acts as an allosteric inhibitor by reshaping the active site.

### Structure-activity study leads to structural optimization of hit compound 3

As the next step, we aimed to increase the affinity of the DEL hits to generate a cellularly effective DNMT2 modulator. Compound **3** was chosen for structure-activity-relationship studies (SAR) due to its favorable binding characteristics observed in ITC, MST, and ^3^H-based enzyme assays. Its suitability for SAR investigation was enhanced by the feasibility of conducting solid-phase peptide synthesis (SPPS) to generate a diverse ligand analog set. A major advantage for compound **3** optimization is given by the elucidated crystal structure, which allows rational considerations for improving ligand interactions in the DNMT2 inhibitors design. First, a computational SeeSAR analysis of the ligand binding pose was performed, which provided information on putative approaches for ligand optimization (Figure S21).^52^

The following hypotheses on the ligand binding characteristics were derived from this computer-assisted analysis: Most atoms of ligand **3** are already optimally positioned for interaction with the DNMT2 protein (green spheres). A few atoms that are assumed to have an unfavorable effect on the DNMT2 interaction (red spheres of the BB2 and BB3 carbonyl oxygens) cannot be exchanged without the loss of the characteristic U-shaped ligand. In conclusion, we hypothesized that modification to the peptide backbone would lead to a significant loss of binding affinity. The peptide backbone’s dihedral angles are mostly in optimal shape for BB3 and BB4 but show minor potential for improvement in BB1 and BB2. Interestingly, there are three regions in the ligand binding pose that allow ligand expansion (gray shading) and thus enable additional potentially beneficial interactions. These regions are localized (1) between BB1 and the C-terminus, which may allow ligand macrocyclization, (2) in the homo-tryptophan residue 5-position, and (3) near the BB2 cyclopropyl motif. These regions were to be investigated by substitutions and scaffold-hopping strategies in the SAR study. Subsequently, a total of 40 derivatives derived from hit compound **3** were synthesized and screened for DNMT2 binding using the MST assay at a final ligand concentration of 100 μM. If significant binding to DNMT2 was measured (>25% FTAD displacement), the binding affinity was determined by following ITC experiments. A comprehensive overview of compound and assay data can be found in Table S1.

**Figure 5:**
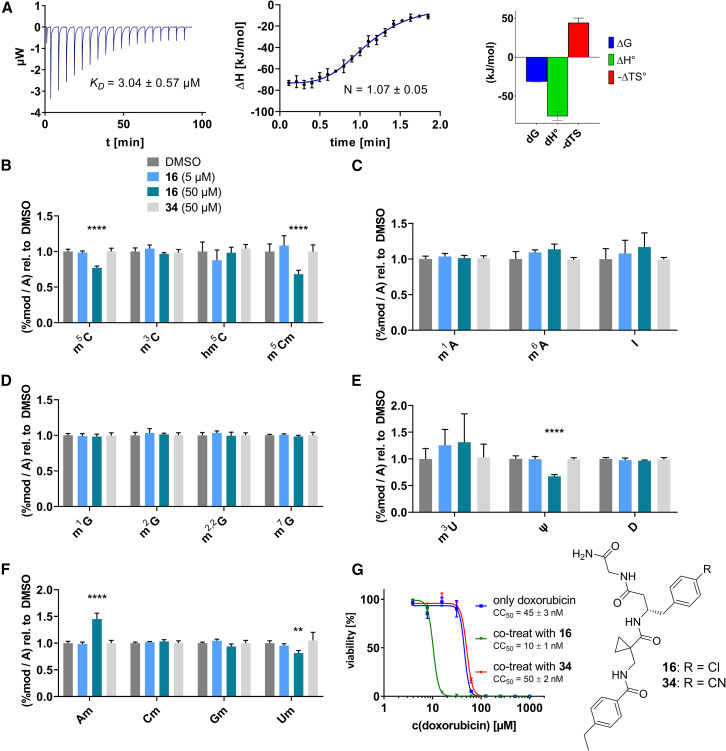
Biophysical and cellular evaluation of SAR study’s most potent compound **16** (A) Compound **16** showed a *K*_D_ = 3.04 ± 0.57 μM in ITC experiments. (B) MOLM-13 cells were treated for 72 h with compound **16** (5 and 50 μM) or **34** (50 μM). tRNA^Asp−GTC^, tRNA^Val−AAC^, and tRNA^Gly−GCC^ were enriched by an antisense pull-down strategy. LC-MS/MS-based assay results for cellular tRNA modification changes analysis. Changes in cytosine modifications relative to the DMSO control. (C) Changes in adenine modifications relative to the DMSO control. (D) Changes in guanine modifications relative to DMSO control. (E) Changes in uridine modifications relative to DMSO control. (F) Changes in 2′*O*-methylations relative to the DMSO control. ∗*p* < 0.05, ∗∗*p* < 0.01, ∗∗∗*p* < 0.001, ∗∗∗∗*p* < 0.0001. (G) Dose-response curves from cell viability assays of doxorubicin/compound **16** resp. **34** co-treated MOLM-13 cells. Cells were cultured for two passages of each three days in the presence of compound **16** or **34** (50 μM) and subsequently treated with varying concentrations of doxorubicin for 48 h. Data are represented as mean ± SEM. Supplementary cell viability assays can be found in Figure S9.

During this SAR study all building block positions (BB1–BB4) were systematically varied: For the BB1 variation (4-ethylbenzoyl), it was observed that the alkyl chain at the phenyl ring’s *para* position was replaceable, still resulting in significant binding affinities. Notably, the presence of a (rigidized) alkyne group in this position resulted in the complete DNMT2 binding abolition. Furthermore, substitutions other than the *para* position, as well as the aromatic moiety’s absence, failed to sustain ligand affinity. Next, we observed that any BB2 substitution, shortening, or decyclization of cyclopropyl-β-alanine was also not tolerated, highlighting the critical necessity of this special β-amino acid. For the BB3 substitution (β-homo-tryptophan), all attempts to shorten the peptide backbone, altering the stereochemistry, and most tryptophan ring system replacements yielded no binding to DNMT2. However, replacement with the phenyl-containing analog (β-homo-(*R*)-phenylalanine, (**14**)) resulted in significant binding. Subsequent synthesis of diverse *para*-substituted derivatives showed improved binding affinity compared to parent compound **3**, which could be confirmed by subsequent dose-response MST and ITC experiments (*K*_*D*_(**16**) = 3.04 ± 0.57 μM, Figure 5A). When substituting BB4 (glycine), extending the peptide backbone, or exchanging with either (*R*)- and (*S*)-alanine, BB4 derivates showed significantly lower DNMT2 affinities. Interestingly, amide group methylation between BB1 to BB4, yielded no significant ligand affinity to DNMT2. As an exception, the peptide C-terminus methylation among other modifications (see also probes **6** and **8**) did not disturb the ligand affinity, and thus, represented a potential exit vector from the DNMT2 pocket suitable for ligand conjugation.

In summary, our experimental SAR is a major confirmation of the SeeSAR hypotheses and agrees well with the crystal structure’s ligand pose. The BB3 replacement from β-homo-(*R*)-tryptophane (**3**) to β-homo-(*R*)-(4-chloro)phenylalanine (**16**) led to an improvement in ligand affinity, while most other modifications resulted in similar or lower ligand affinities. Since peptidomimetic drugs might be prone to cellular permeability issues, hit compound **3** and the most potent ligands **10**, **14**, **16**, **47**, and **54** were tested for passive membrane transport using a parallel artificial membrane permeation assay (PAMPA). While compounds **3**, **10**, **14**, **47**, and **54** failed to pass through the artificial membrane, compound **16** showed moderate passive permeability (*P*_*app*_ = 0.3 10^−6^ cm/s), rendering this compound as the most promising lead structure of this SAR optimization. Detailed PAMPA results can be found in the supporting information (Table S2). Overall, compound **16** showed the best ligand binding properties, and it also demonstrated the ability for passive membrane transport in the PAMPA, making it a promising candidate for cellular studies of DNMT2 inhibition.

### Cellular potency of allosteric inhibitors targeting DNMT2

Next, we aimed to investigate whether compound **16** can affect enzymatic DNMT2 activity in a cellular context and is thus able to reduce RNA modification contents in living systems. Previously, DNMT2^−/−^ mice were found to have reduced m^5^C levels in corresponding tRNAs (tRNA^Asp−GTC^, tRNA^Val−AAC^, and tRNA^Gly−GCC^), while only the double-knockout (DNMT2^−/−^ & NSUN2^−/−^) mutant resulted in a complete m^5^C elimination in tRNA substrates of DNMT2.^13^ Further experiments utilizing siRNA-mediated DNMT2 knockdown and chemical inhibition with the pan-DNMT/NSUN inhibitor azacitidine in leukemia cells demonstrated a total C38 methylation loss.^53^^,^^54^ Following these observations, we aimed to investigate whether **16** can reduce m^5^C modification levels in cells through isoacceptor-specific enrichment of DNMT2 tRNA substrates and modification analysis by means of an LC-MS/MS-based assay. In this regard, following previous studies investigating DNMT2’s role in leukemia cells,^54^^,^^55^ we treated MOLM-13 cells with **16** (5 and 50 μM) for 72 h, followed by the isolation of isoacceptor-specific tRNAs using biotinylated antisense DNA oligomers on magnetic beads. LC-MS/MS-based analysis of digested total tRNA isolates was used to determine relative changes in modification levels of 18 abundant tRNA modifications.^56^

As shown in Figure 5, levels of targeted tRNA modifications (m^5^C and m^5^Cm) were significantly reduced when MOLM-13 cells were treated with 50 μM of compound **16**. In this regard, the m^5^C reduction (∼25%) is in agreement with previous knock-down experiments, highlighting that 75% of tRNA m^5^C are mediated by NSUNs.^13^ Notably, treatment with 5 μM of compound **16** was found to be too low to stimulate any significant change in RNA modifications, probably due to its moderate cell permeability and proportionally lower cellular concentration. In addition, only minor other modification changes were observed, including mildly reduced pseudouridine and Um levels resp. slightly increased Am levels, highlighting our inhibitor chemotype’s DNMT2-selectivity. The origin of those off-target RNA modification alterations remains not entirely solved, but we speculate that RNA-modifying enzymes introducing those modifications are likely to be regulated by DNMT2-mediated m^5^C modification, rendering them as hierarchical-type modifications.^57^ In contrast, treatment of MOLM-13 cells with a tailor-made chemical negative control compound **34**, which does not bind DNMT2, showed, as expected, no reduction of m^5^C in tRNA (Figures 5B–5F).

**Figure 6:**
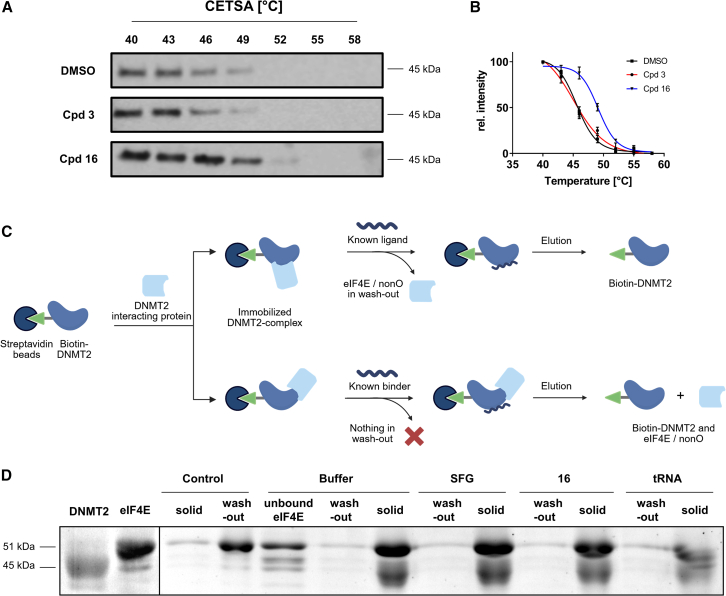
Investigation of allosteric DNMT2 interaction profiles and target engagement (A) Cellular thermal shift assay (CETSA) to evaluate the DNMT2 target engagement of compounds **3** and **16** in MOLM-13 cells. Uncropped Western blots can be found in Figure S22. (B) Densitometric analysis of CETSA Western blots highlights the stabilization of DNMT2 in the presence of compound **16** (50 μM) but not compound **3**. (C) Pull-down competition assay workflow. Biotinylated DNMT2 was immobilized on magnetic streptavidin beads and incubated with recombinant eIF4E protein. Subsequent treatment with the known ligands sinefungin (SFG), compound **16**, or tRNA^Asp^ was used to examine if such ligands can dissociate the DNMT2-eIF4E interaction. (D) Results of the SDS-PAGE-based analysis. Lane description from left to right: DNMT2/eIF4E: recombinant proteins for size comparison. References were applied at another gel position and merged in this figure; Control: beads without immobilized DNMT2 served as the negative control, i.e., on these beads no binding of eIF4E was observed. Treatment (buffer, SFG,**16**, tRNA): The lane “unbound eIF4E” contains the supernatant after the immobilization of eIF4E, which showed low protein levels, thus, most eIF4E is effectively immobilized on DNMT2 beads. Lanes labeled with (wash-out) contain the supernatant of the respective treatment conditions (buffer, 100 μM SFG, 100 μM **16**, 10 μM tRNA^Asp^), while bands labeled with (solid) contain the solid fraction obtained by boiling the residual beads in Laemmli buffer. Data are represented as mean ± SEM. Uncropped PAGE images can be found in Figure S22.

To confirm that our lead compound **16** successfully engages DNMT2 in the MOLM-13 cellular environment, we performed cellular thermal shift assays (CETSA) with compounds **16** (cell-active) and **3** (non-permeable control). As expected, we found thermal stabilization of DNMT2 in the CETSA assay after incubation with 50 μM of **16**, whereas no thermal stabilization occurred in a comparable experiment with compound **3** (Figures 6A and 6B). We conclude that our lead structure **16** engaged DNMT2 in MOLM-13 cells and that m^5^C inhibition is likely mediated by DNMT2 inhibition.

Compound **3**, on the other hand, did not lead to effective DNMT2 engagement due to its lack of passive permeability (Table S2). Based on predictions from Molinspiration and SuperCypPred toolkits, cell-evaluated compounds **3**, **16**, and **34** display highly similar physicochemical profiles, suggesting no significant differences in their expected pharmacokinetic profile (Table S4). The values for molecular weight, topological polar surface area (TPSA), logP, hydrogen bond donors and acceptors, as well as the number of rotatable bonds are all similar. The compounds comply with Lipinski’s Rule of Five, except for compound **3**, which violates one parameter (molecular weight >500 Da). According to SuperCypPred predictions, compounds **3** and **16** are inactive against all tested CYP isoforms except CYP3A4, while compound **34** shows no predicted CYP activity (Figure S23). In principle, these findings are in line with our expectations based on research in the literature: The DNMT2 inhibitors presented in this study belong to the class of “peptidomimetics with non-natural amino acids”, which are known to be limited in their passive permeability, but have good proteolytic and metabolic stability.^58^ From the combined results, including the CETSA assay results and the *in vitro* permeability studies (PAMPA), we conclude that only compound **16** but not compound **3** is permeable and that this permeability difference is the reason for the different cellular activities.

Since the genetic DNMT2 knockdown leads only to minor morphological changes in healthy and cancer cells, but the use of the FDA-approved pan-DNMT/NSUN inhibitor azacitidine is an effective cytostatic drug in leukemic cells, the question of whether DNMT2 is a cell viability-affecting drug target for cancer has been discussed for years.^59^ We therefore investigated the effect of the DEL hit compounds **1**–**5** and **16** on the cell viability of a diverse panel of cancer cell lines (MOLM-13, HCT, Huh-7, and MDA-MB-231) and one non-cancerous cell line (HEK293) and compared it with the effect of azacitidine (Figure S9).^54^^,^^55^ Using an ATP-quantifying cell viability assay, after treatment with varying concentrations of test compounds for 48 h, triazines (**1** and **2**) exhibited cellular toxicity only at concentrations >100 μM, while the peptidomimetic hit compounds (**3**–**5** and **16**) showed only slight reduction in cell viability even at high micromolar concentrations. This suggests that viability is not affected due to DNMT2 inhibition since 50 μM of **16** leads to an efficient m^5^C level reduction in MOLM-13 cells. This is further supported by cell viability experiments in HCT mutant cells, including the investigation of HCT DNMT2 knock-down and HCT NSUN2 knock-down cell lines, highlighting low toxicity of all peptidomimetic compounds at 100 μM. From these cell-based investigations, we conclude that the cytotoxic effect of azacitidine (CC_50_ < 10 μM in all cell lines) is not a cause of DNMT2 inhibition but is mediated by DNMT1/3 and NSUNs inhibition. In summary, our DNMT2 inhibitor **16** is well capable of reducing tRNA m^5^C levels in a cellular context, but at the same time not reducing the cell viability of cancer cells.

However, it is known from previous publications that a genetic DNMT2 knockdown makes cancer cells more vulnerable to chemotherapeutic treatment with doxorubicin on cancer cell lines.^60^ Hence, we exposed selected cell lines to a co-treatment with compound **16** (50 μM) and a variable dose of doxorubicin to analyze cell viability using the viability assay described above. Cultivation of MOLM-13, Huh-7 and MDA-MB-231 cells in the presence of 50 μM compound **16** for two passages of three days each led to no observable reduction in cellular viability but made the cells more susceptible to the subsequent treatment with doxorubicin (Figure 5G; Figures S9H and S9I). As expected, co-treatment with the tailor-made negative control **34** (50 μM) did not lead to a synergistic reduction in viability.

### Known DNMT2-interacting proteins do not bind to the allosteric pocket

The discovery of allosteric DNMT2 modulators mediating cellular m^5^C reduction implicates the question of whether this allosteric pocket may play a role in the cellular regulation of DNMT2 activity. The peptidomimetic structure of the identified hit compounds **3**–**5** might hint at a putative protein-based endogenous ligand, but so far, no allosteric DNMT2 interaction partners have been reported to regulate the enzymatic function. Yet, it was previously found by immunoprecipitation and peptide fingerprinting experiments that human DNMT2 shows interaction with a handful of cellular proteins, which are involved in RNA processing and stress response, including eIF4E, splicing factors NonO resp. SfpQ, and proteins involved in RNA transport.^20^ However, it is unknown in which conformation these proteins bind to DNMT2 and whether these can influence DNMT2 activity.

To study a potential connection to our elucidated allosteric pocket, we probed the interaction between recombinant eIF4E resp. NonO proteins and DNMT2 by means of a pull-down competition assay. In this regard, we immobilized biotinylated DNMT2 on streptavidin-coated magnetic beads and incubated with recombinant eIF4E or NonO, which are able to bind to DNMT2. Subsequent treatment with the established DNMT2 ligands sinefungin (100 μM), compound **16** (100 μM), or tRNA^Asp^ (10 μM) was used to analyze if those ligands are able to dissociate the complex between eIF4E/NonO and DNMT2. SDS-PAGE analysis of the pulled-down fractions, including the separated beads (solid) and supernatants (wash-out) revealed that NonO and eIF4E show efficient binding to DNMT2 on magnetic beads but do not bind to the unfunctionalized beads (Figures 6C and 6D; Figure S16), confirming the reports these proteins are in fact DNMT2 interacting proteins. Yet, neither NonO nor eIF4E could be displaced from binding to immobilized DNMT2 by competitive treatment with compound **16**, sinefungin (SFG), or tRNA^Asp^, indicating that both NonO and eIF4E do not bind to the cryptic allosteric binding pocket identified. We hypothesize that both proteins might bind to a distinctive epitope outside of the MTase active site and reject the hypothesis that the identified allosteric pocket is an endogenous binding site at least for these known DNMT2 interacting proteins.

To summarize this study, our DEL screening successfully identified compounds that target a cryptic allosteric pocket of DNMT2, inducing a conformational change that blocks the SAH-binding active site and leads to subsequent DNMT2 inhibition. In-depth characterization of biomolecular interactions and the development of fluorescent DNMT2 probes revealed that this ligand chemotype showed strong selectivity for DNMT2 within the DNMT/NSUN family. The DNMT2-compound **3** crystal structure revealed the unique inhibitor binding mode causing an active site loop displacement. Synthetic structure-activity relationship studies were performed to improve the affinity of the allosteric ligand, which led to the discovery of a lead compound (*K*_*D*_∼3 μM) with improved passive cell permeability and the ability to inhibit DNMT2 in a cellular context, as demonstrated by reduced tRNA m^5^C content. By this, we could also confirm that selective chemical DNMT2 inhibition is not affecting cell viability in different DNMT2-insensitive cells, but co-treatment with doxorubicin can inhibit MOLM-13 proliferation synergistically. In summary, allosteric inhibition of DNMT2 could be a potential target for a synergistic cancer treatment strategy.

### Limitations of the study

The physiological role of the ligand-induced conformational change remains partly unelucidated and will need further experimentation (e.g., the question of whether this allosteric pocket may play a role in the cellular regulation of DNMT2 activity) and the comprehensive assessment of potential non-m^5^C MTase off-targets. Furthermore, the elucidation of ternary DNMT2-tRNA-SAH resp. compound **3** complexes would strengthen the understanding of the catalytic regulation of this conformational change. Whether this type of DNMT2 inhibitor is effective in more advanced cancer assays including *in vivo* treatment should be investigated once the binding affinity to DNMT2 has been further increased and a cellular high-potent tool compound with low nanomolar potency has been achieved. The double-digit micromolar concentrations needed for cellular m^5^C reduction will leave room for improvement in future inhibitor designs. This could be attempted in the future by developing a compound **3**-based PROTAC, macrocyclization, or the development of covalent inhibitor conjugates, etc.

## Resource availability

### Lead contact

Further information and requests for resources and reagents should be directed to and will be fulfilled by the lead contact, Fabian Barthels (barthels@uni-mainz.de).

### Materials availability

Molecules generated in this study are available from the lead contact upon request and completion of an MTA. DNMT2Δ47 containing plasmid for recombinant protein expression has been deposited to Addgene: #198382.

### Data and code availability

• Coordinates and structure factors of the DNMT2Δ47-compound **3** complex are deposited at the Protein DataBank PDB under accession code 9HGM.
• This paper does not report original code. All software and algorithms are listed in the key resources table.
• Any additional information required to analyze the data reported in this paper is available from the lead contact upon request.

## Acknowledgments

We thank Francesca Tuorto (Heidelberg) for providing NSUN2 knock-down cell lines, Ganna Podoprygorina (Mainz) for support during LC/MS measurements, WuXi AppTec for support during sequencing and data analysis of the DEL screenings, Hanna Bodenstaff (Mainz) for support during the synthesis of the inhibitors, Karin Pauly (Mainz) for support during cell viability assays, and Michael Klein (Mainz) for support during the preparation of Biorender illustrations. We acknowledge access to ESRF Grenoble beamline ID30B and thank the ESRF staff for their support. We thank Claudia Siegmann from the CCTP/BZH crystallization platform for excellent technical support. F.B. acknowledges the support from TRR319 (Project-ID 439669440) TP A01. I.S., M.S., and J.K. acknowledge the service SDS@hd supported by the Ministry of Science, Research and Arts Baden-Württemberg and the 10.13039/501100001659DFG through grants INST 35/1314-1 FUGG, INST 35/1503-1 FUGG. Financial support by the 10.13039/501100001659Deutsche Forschungsgemeinschaft via TRR319 (Project-ID 439669440) B03 to I.S. and TP C01 to M.H. is gratefully acknowledged.

## Author contributions

A.F.F. performed DEL panning, analysis of hit clustering data, hit selection, microscale thermophoresis, ^3^H-assays, FP assays, synthesis of compounds, SeeSAR analysis, and writing of the manuscript; M.S. performed cloning, expression, purification, and characterization of DNMT2Δ47, protein crystallization, data collection, model building, and writing of the manuscript; A.C.W. performed ITC measurements, and cell cultivation; V.K. performed synthesis, MST measurements, AS-MS assay, and pull-down assays; J.K. performed protein crystallization, data collection, model building, and writing of the manuscript; Z.N. performed protein expression and tRNA preparation; R.A.Z. performed protein expression and developed the idea for DNMT2Δ47 mutant; L.G. performed cell viability assays and tRNA LC/MS sample preparation; C.Z. performed PAMPA; M.J. performed cell viability assays; K.F. performed concept of study, supervision, and writing of the manuscript; M.H. performed concept of study, supervision, and writing of the manuscript; I.S. performed concept of study, supervision, data analysis, and writing of the manuscript; F.B. performed concept of study, supervision, and writing of the manuscript. All authors commented on the manuscript.

## Declaration of interests

Mark Helm is a consultant for Moderna Inc.

## STAR★Methods

### Key resources table

**Table:** 

REAGENT or RESOURCE	SOURCE	IDENTIFIER
**Bacterial and virus strains**		

*E. coli* Rosetta 2 (DE3) pLysS cells	Merck KGaA	71403

**Chemicals, peptides, and recombinant proteins**		

Recombinant human DNMT2 (full-length)	Schwickert et al.^3^	N/A
Recombinant human DNMT2Δ47	Schwickert et al.^4^	N/A
Recombinant human DNMT1	ActiveMotif	31404
Recombinant human DNMT3A/3L	Schwickert et al.^3^	N/A
Recombinant human NSUN2	Schwickert et al.^3^	N/A
Recombinant human NSUN3	Schwickert et al.^3^	N/A
Recombinant human NSUN6	Schwickert et al.^3^	N/A
Recombinant human NonO	ActiveMotif	31539
Recombinant human eIF4E	Cayman Chemicals	15137
Anti-DNMT2 (rabbit)	Abcam	ab308120
IRDye 680RD Goat anti-Rabbit	LI-COR	926–68071
TEV protease	New England Biolabs	P8112S
*S*-adenosylhomocysteine (SAH)	Sigma-Aldrich	A9384
Sinefungin	Sigma-Aldrich	S8559
^3^H-*S*-adenosylmethionine (SAM)	Hartmann Analytics	ART0288
*S*-adenosylmethionine (cold)	New England Biolabs	B9003S
L-α-phosphatidylcholine	Sigma-Aldrich	P3556
5-FAM-triazolyl-adenosyl-Dab (fluorescent DNMT2 tracer)	Zimmermann et al.^42^	FTAD
ProteOrange staining	Lumiprobe	10210
Sybr Gold nucleic acid stain	ThermoFisher	S11494
RPMI-1640 medium	ThermoFisher	11875093
McCoys’s 5A medium	ThermoFisher	16600082
DMEM medium	ThermoFisher	10564011
Fetal bovine serum	ThermoFisher	12662029
Penicillin/Streptomycin	ThermoFisher	15070063
Doxorubicin	BLDpharm	BD32885
l-Glutamine	ThermoFisher	A2916801
TRIzol reagent	ThermoFisher	15596026
Nuclease P1 from Penicillium citrinum	Sigma-Aldrich	N8630
Snake venom phosphodiesterase from Crotalus adamanteus	Sigma-Aldrich	P3243
Bovine intestine phosphatase	Sigma-Aldrich	A2356
Benzonase	Sigma-Aldrich	70746
Pentostatin	Sigma-Aldrich	SML0508
Milk powder (blotting grade)	Carl-Roth	T145.3

**Critical commercial assays**		

DELopen screening library	WuXiAppTec	k00000664
Gold MV liquid scintillation cocktail	PerkinElmer	6013151
Cell Titer Glo assay kit	Promega	G7570
ViaLight Plus Cell Proliferation and Cytotoxicity BioAssay Kit	Lonza	LT07-221

**Deposited data**		

Crystal structure DNMT2Δ47-compound 3 complex	This paper	PDB 9HGM

**Experimental models: Cell lines**		

Human MOLM-13 cells	DSMZ	ACC 554; CVCL_2119
Human HCT 116 cells	ATCC	CCL-247; CVCL_0291
Human MDA-MB-231 cells	DSMZ	ACC 732; CVCL_0062
Human Huh-7 cells	DSMZ	PTA-7435; CVCL_0336
Human HEK-293 cells	ATCC	CRL-1573; CVCL_0045

**Oligonucleotides**		

Human IVT tRNA^Asp^-GUC	Schwickert et al.^3^	N/A
5′-GTGACAGGCGGGGATACTCACCACT-Biotin-3′	GenScript	Custom synthesis
5′-CGTGGCAGGCGAGAATTCTACCACT-Biotin-3′	GenScript	Custom synthesis
5′-CGTGTTAGGCGAACGTGATAACCACT-Biotin-3′	GenScript	Custom synthesis

**Recombinant DNA**		

pET-Dnmt3A3L-sc27	Addgene	Addgene_71827
pET-28a(+)-DNMT2Δ47	Addgene	Addgene_198382
pET-28a(+)-NSUN2	Addgene	Addgene_188059
pET-28a(+)-NSUN6	Addgene	Addgene_188060

**Software and algorithms**		

MO.Affinity Analysis 2.3	Nanotemper Technologies	https://nanotempertech.com/
PEAQ-ITC Analysis Software 1.21	MicroCal	https://www.malvernpanalytical.com/
Prism 8.0.1	GraphPad	https://www.graphpad.com/
Unicorn7	Cytiva	https://cytivalifesciences.com
CCP4 software package	Winn et al.^61^	https://ccp4.ac.uk
Phenix software package	Liebschner et al.^62^	https://phenix-online.org
Coot 0.9.8.5	Emsley et al.^63^	https://www2.mrc-lmb.cam.ac.uk/personal/pemsley/coot
MolProbity	Chen et al.^64^	http://molprobity.biochem.duke.edu
PyMol 2.5.7	Schrödinger LLC	https://pymol.org
BioLuminate 5.8	Schrödinger LLC	https://schrodinger.com
SeeSAR 13.0.1	BiosolveIT	https://biosolveit.de
Agilent MassHunter B.05.00	Agilent	https://agilent.com
MestReNova 14.0.1	Mestrelab	https://mestrelab.com
Alphafold 3	Jumper et al.^65^	https://alphafoldserver.com/
Molinspiration property calculation service	Molinspiration Cheminformatics	https://molinspiration.com
SuperCYPsPrep	Banerjee et al.^66^	https://insilico-cyp.charite.de/SuperCYPsPred
ImageLab 6.1	Bio-Rad	https://bio-rad.com

**Other**		

HisPur Ni-NTA magnetic beads	ThermoFisher	88831
Monolith standard capillaries	Nanotemper Technologies	MO-K022
96-well half-area plates	Greiner Bio-One	675076
Zeba Spin Desalting columns 7K MWCO	ThermoFisher	89882
MAIPNTR10 donor plates	Sigma-Aldrich	MAIPNTR10
Superdex 200 Increase 3.2/300	Cytiva	28990946
EZ-Link Sulfo-NHS-LC-Biotinylation kit	ThermoFisher	21435
Streptavidin MagBeads	GenScript	L00936
Monarch RNA cleanup (500 μg)	New England Biolabs	T2050S
Whatman glass microfiber filters (GF/C, 25 mm)	Sigma-Aldrich	WHA1822025
Protran nitrocellulose membrane (0.45 μm)	Cytiva	10600003
Criterion TGX Precast Protein Gels (4–20%)	Bio-Rad	5671095

### Method details

#### DEL screening

DEL screening against human DNMT2 protein was performed with the fourth-generation DELopen library (4.2 billion compounds, 27 sub-libraries) by WuXi AppTec. Screening was carried out according to the manufacturer’s protocol (available online: https://hits.wuxiapptec.com/assets/pdf/DELopen_Protocol.pdf). Briefly, the DEL panning was performed with recombinant human full-length DNMT2 protein immobilized via the His-tag on ThermoScientific HisPur Ni-NTA magnetic beads. The beads were first washed in a magnetic separation rack with washing buffer (50 mM HEPES, 150 mM NaCl, 5 mM MgCl_2_, 0.05% polysorbate-20, pH 7.5), and 250 μg beads were transferred for each selection condition to a separate tube. DNMT2 was diluted according to the manufacturer’s protocol in selection buffer (50 mM HEPES, 150 mM NaCl, 5 mM MgCl_2_, 0.05% Polysorbate-20, pH 7.5, 0.1 mg/mL sheared salmon sperm DNA, 1 mM TCEP, and 10 mM imidazole) and successful immobilization on the beads was confirmed by the SDS-PAGE protein capture assay described in manufacturer’s protocol. A differential panning procedure was employed with four different selection conditions (A–D): (**A**) Native DNMT2 without additives; (**B**) DNMT2 with SAH (100 μM); (**C**) DNMT2 with tRNA^Asp^ (10 μM); (**D**) No target control with no DNMT2 immobilized.

After three iterative selection rounds, the enriched DNA samples were transferred to WuXi AppTec for further processing, including PCR amplification, quantitation by qPCR, and deep sequencing. All samples passed the DELopen quality control (Figures S1A and S1E) and were forwarded to Illumina NovaSeq deep sequencing. Subsequently, DEL hits selection (Figure S1C) was decided based on enrichment scores, which were calculated by the DELopen unique algorithm described by Kuai et al.^41^

#### Microscale thermophoresis (MST)

Screening for ligand binding and evaluation of dose-response affinity profiles was performed via microscale thermophoresis (MST) as described previously.^42^ The measurements were conducted with a Nanotemper Technologies Monolith NT.115 device. All samples were measured in Monolith standard capillaries at 25 °C. The acquisition mode was set to “Nano-Blue” with an excitation power of 30% and a medium MST power, while the system was controlled using the software MO.Control (version 1.6.1) and for evaluation MO.Affinity Analysis (version 2.3) was used. The samples contained 2 μM DNMT2, 100 nM FTAD (5-FAM-triazolyl-adenosyl-Dab) probe, and 1.3% DMSO in MST buffer (50 mM HEPES, 150 mM NaCl, 1 mM dithiothreitol (DTT), 0.1% PEG-8000, 0.05% polysorbate-20, pH 7.4).^42^ All compounds were employed at a final concentration of 100 μM for the initial ligand screening.

For *K*_*D*_-value determination of the DEL hit-derived fluorescent tracers **6**–**8**, the respective probe was used at a concentration of 100 nM (instead of FTAD), and the DNMT2 concentration was varied (50 μM–0 μM). All measurements were performed as triplicates. The *K*_*D*_-value was calculated and plotted in GraphPad Prism 8.0.1. MST experiments with fluorescently labeled tRNA (non-covalent labeling with SybrGold) were used to investigate if hit compounds **1**–**5** can dissociate the nucleic acid-protein complex formed by DNMT2 and were conducted as described previously.^47^ To assess the DEL ligands’ effect on the RNA binding, the tRNA^Asp^ substrate (100 nM) was mixed with the DNMT2 enzyme (2 μM) supplemented with 1x SybrGold and 100 μM of the respective ligand.

#### Isothermal titration calorimetry (ITC)

The DNMT2 protein was purified via size exclusion chromatography eluting in isothermal titration calorimetry (ITC) buffer (50 mM sodium phosphate pH 8.0, 300 mM NaCl, 1 mM EDTA, 2 mM β-mercaptoethanol, 0.1% polysorbate-20) and directly used for measurements.^3^ All compounds were dissolved in DMSO to a concentration of 5 mM and were then diluted in ITC buffer to a final concentration of 500 μM. DNMT2 was diluted to a final concentration of 50 μM with 10% DMSO to match the ligand stocks' DMSO concentration. For the ITC measurements, a MicroCal PEAQ-ITC Automated workstation (Malvern Panalytical) with a 200 μL Hastelloy cell and an injection syringe volume of 40 μL was used. All experiments were performed in triplicates at 25 °C unless otherwise noted. For each measurement, 19 compound injections (2 μL each) with an injection speed of 0.5 μL/s were added to the reaction cell containing DNMT2. The spacing time between sequential injections was 300 s, and the stirring speed was set to 750 rpm with a reference power of 42 μW. As control experiments, ligands were titrated into ITC buffer. Data analysis and fitting were carried out using the MicroCal PEAQ-ITC Analysis Software 1.21 and plotted in GraphPad Prism 8.0.1.

Furthermore, displacement ITC experiments were performed as described for the direct titrations above with the following modifications: DNMT2 (50 μM) was preincubated with compound **10** (500 μM, 10% DMSO) or compound **3** (100 μM, 10% DMSO). Subsequently, SAH (500 μM, 10% DMSO) was titrated to preincubated protein. During the same sample set, SAH (500 μM) was titrated directly into DNMT2 (50 μM) as a reference titration. Data analysis and displacement curve fitting were accomplished via the MicroCal PEAQ-ITC Analysis Software.

#### Fluorescence polarization (FP)

Fluorescence polarization (FP) measurements with RNA MTases and small-molecule fluorescent tracers were conducted in black Greiner 96-well half-area plates using a Tecan Spark 10M plate reader as described previously.^44^ The reader was equipped with polarization filters coupled to a monochromator setup (λ_ex_ = 480 nm, λ_em_ = 530 nm). For selectivity screening, reaction mixtures contained varying concentrations of recombinant enzyme (DNMT1, DNMT2, DNMT3A/3L, NSUN2, NSUN3, or NSUN6; preparation see below) and 10 nM DEL hit-derived probe **6** in MST buffer. For displacement experiments, 100 μM SAH or 10 μM tRNA^Asp^ were added from buffered stock solutions to the DNMT2-containing mixtures. All measurements were performed as triplicates. Polarization values (in mP) were calculated from polarization-specific parallel and orthogonal fluorescence intensities according to the Tecan in-built calculation routine. *K*_*D*_-values were calculated and plotted in GraphPad Prism 8.0.1.

#### ^3^H-based methyltransferase enzyme activity assays

Enzymatic DNMT2 inhibition assays were carried out in 100 mM Tris-HCl, pH 8.0, 100 mM NH_4_OAc, 0.1 mM EDTA, 10 mM MgCl_2_, and 10 mM DTT as described previously.^3^^,^^4^ First, the tRNA^Asp^ substrate was heated to 75 °C for 5 min and cooled down to room temperature (RT) before it was added to the reaction mixture to a final concentration of 5 μM. The reaction mixture contained 250 nM DNMT2 and the ligand compounds at variable concentrations (5% DMSO). Enzymatic reactions were started by the addition of 0.9 μM SAM as a mixture of ^3^H-SAM (Hartmann Analytics) resp. cold SAM (NEB) to a final activity of 0.025 μCi μL^−1^. Enzyme assays were carried out for 30 min at 37 °C. As a negative control served the reaction mixture without DNMT2 and as a positive control the reaction mixture without test compound. After 30 min, aliquots of 8 μL were taken from the reaction mixture and spotted on Whatman glass microfiber filters (GF/C, 25 mm). The RNA was precipitated on the filters with 5% ice-cold TCA for 15 min. The filters were washed twice with 5% TCA at RT for 20 min and 10 min and once in EtOH for 10 min. After drying, the filters were transferred into scintillation vials, and 3 mL of PerkinElmer Gold MV liquid scintillation cocktail was added. Scintillation was measured with a scintillation counter (TriCarb Liquid Scintillation Analyzer 4810 TR; measurement time of 1 min). For IC_50_-value determination, compounds were analyzed at a minimum of six different concentrations in triplicates. IC_50_-values were calculated, evaluated, and plotted in GraphPad Prism 8.0.1.

#### Affinity selection-mass spectrometry (AS-MS)

Orthogonal *K*_*D*_-value determination for compound **3** was conducted by an affinity selection-mass spectrometry (AS-MS) assay according to Prudent et al.^43^ and Simon et al.^67^ Compound **3** was dissolved in DMSO (20 mM) and a dilution series in Tris buffer (25 mM Tris, 100 mM NaCl, pH 7.5) was prepared to give final ligand concentrations of 100 μM, 50 μM, 25 μM, 12.5 μM, 6.25 μM, 3.12 μM, 1.56 μM, and 0 μM. DNMT2 was diluted in Tris buffer (130 μM) and added to the dilution series to give a final concentration of 5 μM DNMT2. Zeba Spin Desalting columns 7K MWCO were used for the affinity-based separation. These columns were centrifuged at 2000*g* for 1 min to remove the storage buffer. 20 μL of the prepared samples were loaded to the columns, and the protein-ligand complex was eluted by centrifugation at 2000*g* for 2 min. The centrifuged solutions were diluted with 10 μL ACN and analyzed by LC/MS with an Agilent 1100 Series HPLC system. The data evaluation and subsequent binding affinity determination were performed with MestReNova (version 14.0.1) and GraphPad Prism 8.0.1.

#### Electrophoretic mobility shift assay (EMSA)

EMSA assays were performed as described previously.^46^ DNMT2 (10 μM) was mixed with tRNA^Asp^ (5 μM) and treated with 100 μM of the respective hit compound in DNMT2 assay buffer (100 mM Tris-HCl, pH 8, 100 mM NH_4_OAc, 0.1 mM EDTA, 10 mM MgCl_2_, 10 mM DTT). Subsequently, reaction samples (each 5 μL) were loaded to a native 10% polyacrylamide gel and separated electrophoretically (1 h, 180 V) in 1x TBE. RNA was stained *in situ* with 1x GelRed, and PAGE analysis was conducted by fluorescence scanning at an excitation wavelength of 532 nm (Amersham Typhoon 9400).

#### *In vitro* permeability assay (PAMPA)

To determine the passive permeability of selected DNMT2 ligands, a parallel artificial membrane permeation assay (PAMPA) was performed as described previously.^68^ The experiment was performed in duplicates with Sigma-Aldrich MAIPNTR10 donor plates with 5 μL of an artificial membrane (1% w/v l-α-phosphatidylcholine in *n*-dodecane) per well. After incubation for 7 h, the acceptor solutions were evaluated by LC/MS (HP Agilent 1100 Series). The peak areas in the UV chromatogram (λ = 210 nm) were used to calculate the areas under the curve (AUCs). The apparent permeability *P*_*app*_ was calculated using the following equation with V_D_ and V_A_ as volumes of donor and acceptor solutions (0.15 cm^3^ and 0.4 cm^3^), AUC_acc_ and AUC_ref_ as the area of the analyte signal in the respective chromatogram of sample and reference solutions, A as the porosity-corrected filter area (0.2113 cm^2^) and t as the incubation time given in seconds: *P*_*app*_ = −V_D_·V_A_·ln(1−AUC_acc_/AUC_ref_)/[(V_D_ + V_A_)·A·t].

#### Protein and tRNA production

DNMT2, NSUN2, NSUN6, and DNMT3A/3L were expressed from *E. coli* as described previously, while tRNA^Asp^ was synthesized by *in vitro* transcription as described previously.^3^

#### DNMT2 cloning, expression, and purification for co-crystallization

For the co-crystallization of DNMT2Δ47 with compound **3**, the coding DNA sequence of human DNMT2 lacking residues 191–237 was amplified by PCR and cloned into a pETHis_1a vector.^69^ For this purpose, overhangs for the restriction enzymes NcoI/BamHI were used. The plasmid was transformed in Rosetta II (DE3) *E. coli* (Merck-Novagen) and expressed in ZY autoinduction medium supplemented with trace elements, kanamycin, and chloramphenicol. The cells were grown to OD_600_ = 0.8 at 37 °C (220 rpm shaking) and further cultivated overnight at 20 °C. Harvesting was performed by centrifugation and washing with 1xPBS buffer (0.137 M NaCl, 2.7 mM KCl, 10 mM Na_2_HPO_4_, 1.8 mM KH_2_PO_4_). The resulting cell pellet was then resuspended in lysis buffer (20 mM HEPES, pH 7.5, 500 mM NaCl, 20 mM imidazole, 1 M urea, 4 mM β-mercaptoethanol), supplemented with a protease inhibitor mix (Roche) and 1:10000 benzonase. Lysis was performed with a microfluidizer (M1-10L, Microfluidics) followed by clearing the lysate applying centrifugation (40 min at 50,000*g*) and filtering through a 0.45 μm membrane. The supernatant was loaded twice on 4 mL Ni-NTA Agarose (Qiagen) for Ni-IMAC using gravity flow columns (Biorad). The beads were washed with ten column volumes (10 CV) wash buffer (20 mM HEPES, pH 7.5, 150 mM NaCl, 4 mM β-mercaptoethanol), followed by 10 CV high salt buffer (20 mM HEPES, pH 7.5, 2 M NaCl, 4 mM β-mercaptoethanol) and again 10 CV wash buffer. Elution of the His-tagged DNMT2Δ47 was performed with 5 CV elution buffer (20 mM HEPES, pH 7.5, 150 mM NaCl, 300 mM imidazole, 4 mM β-mercaptoethanol). Protein concentration was measured with a NanoPhotometer NP80 (Implen), and TEV protease was added in a 1:75 ratio. Cleavage of the protein by TEV results in two additional residues, namely Gly-Ala, at the N-terminus. The DNMT2Δ47-protease mix was dialyzed overnight against wash buffer using a 6–8 kDa dialysis tube (Spectra/Por). The sample was applied to a reverse Ni-IMAC using 2 mL Ni-NTA Agarose, which was washed with 5 CV wash buffer. The flow-through and wash fractions were pooled, concentrated with an Amicon Ultra concentrator (10 kDa cutoff, Merck KGaA) to 2 mL and loaded for further purification by SEC (size-exclusion chromatography) on a Superdex 75 16/600 gel filtration column (Cytiva) in wash buffer. The peak fractions were pooled, incubated with compound **3** in a 2-fold molar excess, and concentrated with an Amicon Ultra concentrator (10 kDa cutoff, Merck KGaA) to 10 mg/mL for subsequent crystallization.

#### Analytical size-exclusion chromatography of DNMT2Δ47 with compound **3**

To assess the oligomeric state of DNMT2Δ47 upon compound **3** binding, *in vitro* analytical SEC runs were performed on a Superdex 200 Increase 3.2/300 (Cytiva). For the first run, the apo-protein was injected at a concentration of 10 mg/mL; for the second run, it was pre-incubated with a 2-fold molar excess of compound **3**. The resulting chromatograms were analyzed by superposing their 280 nm signals using the Unicorn7 evaluation software (Cytiva).

#### Crystallization, data collection, model building, and refinement

Crystals were grown at 291 K using sitting drop vapor diffusion. The crystallization reservoir was composed of 0.2 M MgCl_2_, 0.1 M Tris, pH 8.5, and 12% PEG-8000. Crystallization drops contained 300 nL reservoir solution and 300 nL concentrated protein. Needle-shaped crystals grew within three days. Crystals were frozen in liquid nitrogen using glycerol as the cryo-protectant. Data were collected at ESRF beamline ID30B at cryogenic conditions, integrated using XDS,^70^ and scaled using AIMLESS^71^ as part of the CCP4 software package.^61^ Phases were obtained by molecular replacement with PHASER^72^ implemented in the Phenix package^62^ using the structure of human DNMT2 (Dong et al.^49^; PDB 1G55) as the search model. The resulting dataset has space group P2_1_2_1_2_1_, a maximum resolution of 2.60 Å, and contains two DNMT2 molecules per asymmetric unit. Compound **3** was parameterized from SMILES description using phenix.elbow.^73^ Iterative model building and refinement were performed with Coot^63^ and phenix.refine.^74^ C24, C79, and C287 side chains show additional electron density in both chains and, therefore, were built as *S*-hydroxycysteine residues (CSO). The quality of the resulting structural models was analyzed with MolProbity.^64^ Structure figures were prepared with PyMol 2.5.7 (The PyMol Molecular Graphics System, Schrödinger LLC.). Hydrophobicity of the binding site was visualized by a molecular surface representation colored from high (white) to low (green) hydrophobicity according to the Eisenberg hydrophobicity scale.^75^ The electrostatic potential was calculated and visualized using the PyMol APBS plugin.^76^ Crystallographic data are summarized in Table S3. Coordinates and structure factors are deposited at the Protein DataBank PDB under accession code 9HGM.

#### SeeSAR analysis

BiosolveIT SeeSAR (v13.0.1) was used to visualize the estimated binding affinity contributions of the co-crystallized DNMT2 ligand. SeeSAR concedes a semi-quantitative calculation of the thermodynamics using the HYDE algorithm and was used to evaluate torsions, desolvation, and interaction contributions for each ligand’s heavy atom.^52^ For this, the DNMT2Δ47-binding compound **3** complex (PDB 9HGM) was imported to SeeSAR and evaluated using the “Analyzer” functionalities.

#### Pull-down DNMT2 interaction assay

For immobilization on streptavidin beads, DNMT2 was biotinylated using the EZ-Link Sulfo-NHS-LC-Biotinylation kit. A 20-fold excess of Sulfo-NHS-LC-Biotin (10 mM in water) was added to DNMT2 and incubated for 2 h on ice. To remove the excess biotin reagent, Zeba Spin desalting columns were equilibrated with 1× PBS buffer, and the reaction mixture was loaded and centrifuged for 2 min at 1000*g*. The biotinylation level was estimated using a HABA assay included in the kit according to the manufacturer’s protocols. By this, 4.7 biotin molecules were found to be attached per DNMT2 molecule. To confirm that the biotinylation did not affect the binding competence of the SAH and drug binding pockets, MST measurements with FTAD as a fluorescent tracer were performed as described above.

For *in vitro* pull-down assays, biotinylated DNMT2 was immobilized on streptavidin-coated paramagnetic beads (GenScript Streptavidin MagBeads). For this, beads were washed with PBS buffer containing 0.05% Triton X-100, and subsequently, DNMT2 (30 μg per 50 μL beads) was immobilized for 45 min at RT. As a negative control, beads were treated with a protein-free buffered solution. Beads were washed with PBS buffer, and for each sample, 5 μL beads were added to either 1.5 μg of recombinant NonO protein (Active Motif) or 3.0 μg of recombinant eIF4E protein (Cayman). Beads were incubated for 1 h at RT, and subsequently, the supernatant was removed. Afterward, 10 μL PBS-buffered elution solution (containing either 100 μM sinefungin or 100 μM compound **16** or 10 μM tRNA) was added. The samples were incubated for 45 min at RT, and the supernatant was treated with 2 μL 5× Laemmli buffer. For analysis of the proteins still bound to the beads after elution treatment, 10 μL Laemmli buffer supplemented with 10 mM biotin was added to the residual beads. All samples were heated to 95 °C for 10 min and analyzed via SDS-PAGE. Fluorometric protein detection was conducted using ProteOrange staining (Lumiprobe) according to the manufacturer’s protocols. Protein bands were visualized using a GE Healthcare Typhoon Trio biomolecular scanner, with laser excitation at 488 nm and fluorescence detection at 580 nm.

#### tRNA modification analysis by LC/MS

MOLM-13 cells were seeded at a density of 5 × 10^6^ cells in 15 mL RPMI-1640 medium (supplemented with 10% FBS) containing DNMT2 inhibitor **16** (5 μM and 50 μM), negative control compound **34** (50 μM), or DMSO as mock treatment. Cells were incubated for 72 h at 37 °C. Post-incubation, cells were harvested and washed with PBS. Cell pellets were lysed in 1.5 mL TRIzol, mixed thoroughly by pipetting, and incubated for 5 min at RT. Subsequently, 150 μL chloroform was added, vortexed, and incubated for 10 min at RT. Samples were centrifuged at 12,000*g* for 15 min at 4 °C. The upper aqueous layer was transferred to new tubes and mixed with 750 μL isopropanol, vortexed, and incubated for 10 min at RT. Following another centrifugation at 12,000*g* for 15 min at 4 °C, the supernatant was discarded. Pellets were washed with 750 μL of 75% ethanol, vortexed, and centrifuged again at 12,000*g* for 15 min at 4 °C. After discarding the supernatant, the pellets were air-dried at 37 °C and dissolved in 50 μL nuclease-free water. Total RNA samples were further purified by New England Biolabs Monarch RNA Cleanup (500 μg) kits according to the manufacturer’s instructions (protocol for RNA size fractionation). Small RNA molecules (<100 nt) containing total tRNA were eluted in 50 μL nuclease-free water, and concentrations were determined using a Nanodrop spectrophotometer.

To enrich the DNMT2 tRNA substrates, a literature-known hybridization-based protocol for the purification of thermostable tRNA was adopted.^77^ Therefore, three biotin-labeled anti-sense DNA oligonucleotides suitable for tRNA pull-down were designed and purchased from GenScript. Sequences complementary to tRNA^Asp−GUC^: 5′-GTGACAGGCGGGGATACTCACCACT-Biotin-3′, tRNA^Gly−GCC^: 5′-CGTGGCAGGCGAGAATTCTACCACT-Biotin-3′, tRNA^Val−AAC^: 5′-CGTGTTAGGCGAACGTGATAACCACT-Biotin-3’. Next, GenScript streptavidin MagBeads (200 μL) were washed thrice with 1 mL TBST. Biotin-labeled DNA-oligonucleotide solutions were prepared at 7.5 μM in 3 × 400 μL TBST. MagBeads were incubated with 400 μL of each Biotin-oligo for 10 min at RT, then washed with TBST. Unbound oligos were measured by Nanodrop to confirm loading, assuming a theoretical capacity of 3 nmol per 200 μL beads. MagBeads were pooled in 1200 μL TBST and distributed into 12 PCR tubes with 100 μL TBST each. Total tRNA samples (100 μL) were mixed with 100 μL hybridization buffer (20 mM Tris, pH 7.6, 1.8 M NaCl, 0.2 mM EDTA) and added to the prepared MagBeads. The mixture was heated RT to 75 °C over 40 min using a PCR cycler and then cooled to 4 °C. Following centrifugation, supernatants were collected and saved. The beads were washed repeatedly with TBST at 4 °C until the absorbance at 260 nm was less than 0.01. tRNA was eluted by heating the beads in 30 μL Tris buffer (10 mM Tris, pH 7.6) at 75 °C, with the supernatant collected after each elution step, repeated three times. tRNA concentration and purity were determined using a Nanodrop spectrophotometer and 10% denaturing PAGE, respectively.

100 ng of enriched tRNA per sample was enzymatically digested into nucleosides using a mixture of 0.6 U nuclease P1 from *Penicillium citrinum* (Sigma-Aldrich), 0.2 U snake venom phosphodiesterase from *Crotalus adamanteus* (Worthington), 0.2 U bovine intestine phosphatase (Sigma-Aldrich), 10 U benzonase (Sigma-Aldrich), along with 200 ng of Pentostatin (Sigma-Aldrich). This reaction was conducted for 2 h at 37 °C in a buffer containing 5 mM Tris (pH 8) and 1 mM magnesium chloride. Following digestion, the nucleosides were spiked with an internal standard of ^13^C stable isotope-labeled nucleosides derived from *Saccharomyces cerevisiae* and analyzed. 100 ng of the digested tRNA and 25 ng of the internal standard were analyzed using LC/MS (Agilent 1260 series coupled with an Agilent 6460 Triple Quadrupole mass spectrometer featuring an electrospray ion source). The mobile phase consisted of a 5 mM ammonium acetate buffer (pH 5.3) and LC/MS grade acetonitrile (VWR). The elution process began with 100% ammonium acetate buffer at a flow rate of 0.35 mL/min, transitioning to a linear gradient that reached 8% acetonitrile at 10 min and 40% at 20 min. The initial conditions were restored to 100% ammonium acetate buffer for an additional 10 min. A Synergi Fusion column (4 μm particle size, 80 Å pore size, 250 × 2.0 mm; Phenomenex) was utilized for the separation. The UV signal at 254 nm was monitored using a multiple wavelength detector (MWD) to track the primary nucleosides. The ESI parameters were set as follows: gas temperature at 350 °C, gas flow at 8 L/min, nebulizer pressure at 50 psi, sheath gas temperature at 350 °C, sheath gas flow at 12 L/min, and capillary voltage at 3000 V. The mass spectrometer operated in positive ion mode, employing Agilent MassHunter B.05.00 software in dynamic MRM (multiple reaction monitoring) mode for analysis. Quantification was achieved through a combination of external and internal calibration methods, as outlined in Kellner et al.^78^

#### Cell viability assays

Cell viability assays were conducted with MOLM-13 cells, Huh-7, MDA-MB-231, HEK293, and HCT MTase mutant cell lines (WT, knock-down DNMT2, and knock-down NSUN2) to investigate the DNMT2 inhibiting compounds’ cellular effect. Cells were cultured according to standard protocols: MOLM-13 (RPMI, 10% FBS, 1x Penicillin/Streptomycin, 37 °C, 5% CO_2_), Huh-7/MDA-MB-231/HEK293 (DMEM, 10% FBS, 1x Penicillin/Streptomycin, 37 °C, 5% CO_2_) and HCT (McCoys’s 5A Medium, 10% FBS, l-Glutamine, 1x Penicillin/Streptomycin, 37 °C, 5% CO_2_). Cell viability was assessed using Promega Cell Titer Glo assay kit (MOLM-13, Huh-7, MDA-MB-231, HEK293) or Lonza ViaLight Plus Cell Proliferation and Cytotoxicity BioAssay Kit (HCT cell lines). Briefly, 1000 cells were seeded in white Greiner half-area 96-well plates and incubated for 48 h with inhibitors/ligands from DMSO stocks (final: 0.1% DMSO) under standard cultivation conditions. Then, the cell titer reagent was added to each well according to the manufacturer’s protocols, and luminescence was measured using a Tecan Spark 10M plate reader (MOLM-13, Huh-7, MDA-MB-231, HEK293) or Molecular Devices FlexStation (HCT cell lines). For the co-treatment with doxorubicin/compound **16** or **34**, cells were cultured for two passages of each three days in the presence of compound **16** or **34** (50 μM) and subsequently 1000 cells each in 96-well plates were treated with varying concentrations of doxorubicin for 48 h.

#### Cellular thermal shift assay (CETSA)

MOLM-13 cells were seeded at a density of 5 × 10^6^ cells in 3 mL RPMI-1640 medium. Subsequently, compound **3** or **16** was added to the MOLM-13 cells to achieve a final concentration of 50 μM, with the same volume of DMSO (final: 0.2%) added as a control. After incubation for 16 h (37 °C, 5% CO_2_), the cell suspensions were collected. The cells were washed twice and then counted, followed by the addition of 1 mL PBS. The cell suspension was divided into eight parts, and then heated for 4 min at various temperatures (40, 43, 46, 49, 52, 55, 58 °C) in a PCR thermocycler. The cells were lysed through a freeze-thaw process using liquid nitrogen. The supernatant solutions were used for immunoblotting analysis. Insoluble cellular parts were separated by centrifugation (12,000*g* for 10 min), subsequently, the supernatant was boiled with 1× Laemmli sample buffer for 5 min at 95 °C. Proteins were separated by SDS−PAGE on 4–20% Criterion TGX Precast Protein Gels (Bio-Rad) and transferred to Amersham Protean 0.45 μm nitrocellulose membrane (Cytiva) using semi-dry transfer (25 V, 1 A, Towbin buffer). The membrane was blocked with 5% milk (Carl Roth) in TBST buffer and incubated with anti-DNMT2 antibody (1:1000, Abcam, ab308120) overnight at 4 °C. Subsequently, the membrane was incubated for 3 h at RT with IRDye 680RD Goat anti-Rabbit (1:1000, LICOR, 926–68071). Protein bands were visualized using a GE Healthcare Typhoon Trio biomolecular scanner, with laser excitation at 637 nm and fluorescence detection at 670 nm. The ImageLab software (Bio-Rad) was used for band quantification.

#### Synthesis and characterization of inhibitors and probes

Rink amide AM resin (loading capacity: 0.6–0.8 mmol/g) or Wang Gly resin (loading capacity: 0.135 mmol/g) was swelled twice for 5 min in DMF. Fmoc-deprotection was performed by treating with 40% piperidine in DMF (2 × 10 min shaking). The resin was rinsed with DCM (3x) and DMF (3x). In the next step, the corresponding building block was coupled to the resin by dissolving the amino acid (3.0 eq. or 1.2 eq.), TBTU (3.0 eq. or 1.2 eq.), and NMM (9.0 eq.) in DMF and shaking for 1.5 h (or 16 h for 1.2 eq.). Subsequently, the resin was rinsed with DCM (3x) and DMF (3x). To couple the next amino acids, the procedure of deprotection and coupling was repeated. When the last amino acid was coupled, the resin was rinsed with DMF (3x) and DCM (3x). To cleave the peptide from the resin, the resin was treated with a mixture of TFA:TIPS:DCM (50:10:40) for 1.5 h. The resin was washed with DCM (3x) and treated again with TFA:TIPS:DCM (50:10:40) for 16 h. All cleavage and wash solutions combined were evaporated under reduced pressure. The crude product was purified by reversed-phase flash column chromatography (Biotage Isolera, ACN/H_2_O + TFA), and the product containing fractions were lyophilized. Detailed characterization of the compounds synthesized can be found below:

**Figure:**
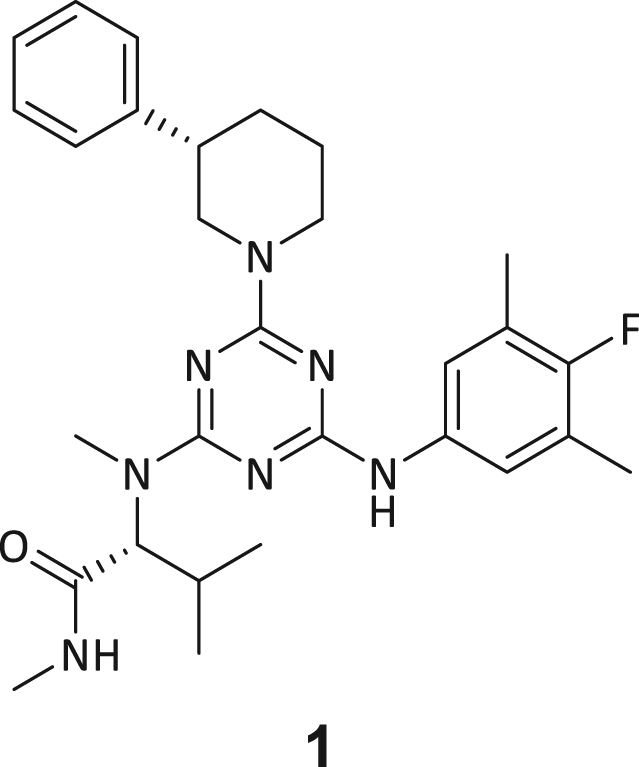

Compound **1**: ^1^H-NMR (400 MHz, DMSO-*d*_6_): δ [ppm] = 8.94 (s, 1H), 7.74–7.16 (m, 6H), 4.76 (s, 2H), 3.32 (d, *J* = 2.8 Hz, 2H), 3.05–2.83 (m, 3H), 2.58 (s, 13H), 2.34–1.90 (m, 6H), 1.79 (d, *J* = 13.0 Hz, 1H), 0.93 (d, *J* = 4.3 Hz, 2H), 0.79 (d, *J* = 6.6 Hz, 2H).

^13^C-NMR (101 MHz, DMSO): δ [ppm] = 170.7, 170.1, 165.9, 165.8, 143.9, 143.9, 136.0, 128.4, 127.0, 126.4, 123.1, 119.8, 62.6, 49.7, 43.4, 42.2, 31.8, 26.4, 25.7, 25.5, 25.1, 20.0, 19.1, 18.8, 14.5.

LC/MS: m/z calculated for C_29_H_38_FN_7_O [M + H]^+^: 520.3, found: 520.3.

**Figure:**
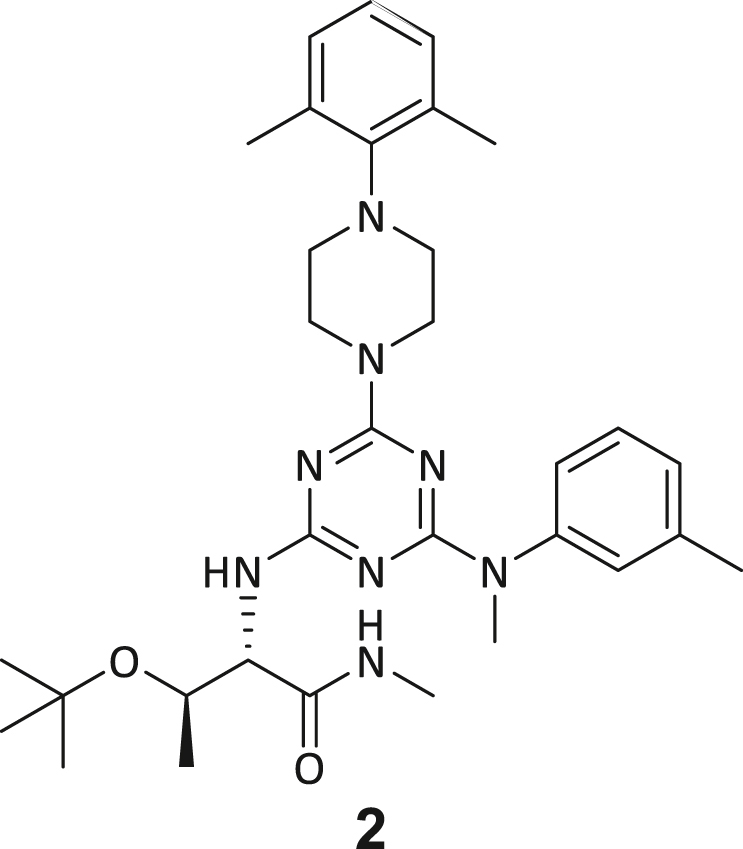

Compound **2**: ^1^H-NMR (400 MHz, DMSO-*d*_6_): δ [ppm] = 7.77–7.68 (m, 1H), 7.27–7.07 (m, 3H), 6.95 (h, *J* = 7.7, 7.1 Hz, 4H), 4.02 (t, *J* = 5.3 Hz, 1H), 3.78–3.68 (m, 3H), 3.38 (s, 3H), 3.32 (s, 3H), 2.97 (d, *J* = 6.0 Hz, 4H), 2.57 (d, *J* = 4.4 Hz, 3H), 2.32–2.23 (m, 9H), 1.06 (dd, *J* = 15.2, 8.3 Hz, 12H).

^13^C-NMR (101 MHz, DMSO): δ [ppm] = 171.0, 165.6, 165.2, 164.4, 148.0, 144.7, 144.5, 137.6, 137.3, 136.1, 128.9, 128.1, 127.9, 127.0, 126.7, 125.7, 125.4, 124.9, 123.7, 123.0, 73.3, 67.2, 59.2, 49.3, 44.1, 37.3, 28.0, 25.7, 21.0, 20.7, 20.5, 19.4.

LC/MS: m/z calculated for C_32_H_46_N_8_O_2_ [M + H]^+^: 575.3, found: 575.2.

**Figure:**
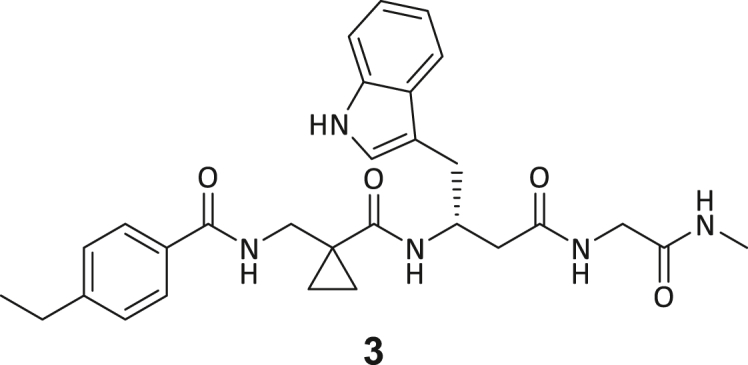

Compound **3**: ^1^H-NMR (600 MHz, DMSO-*d*_6_): δ [ppm] = 10.80–10.76 (m, 1H), 8.58 (t, *J* = 6.1 Hz, 1H), 8.13 (t, *J* = 5.9 Hz, 1H), 8.05 (d, *J* = 7.9 Hz, 1H), 7.76 (d, *J* = 8.2 Hz, 2H), 7.66 (d, *J* = 4.6 Hz, 1H), 7.59 (d, *J* = 7.9 Hz, 1H), 7.29 (t, *J* = 8.2 Hz, 3H), 7.09 (d, *J* = 2.2 Hz, 1H), 7.07–7.01 (m, 1H), 6.98–6.92 (m, 1H), 4.38–4.31 (m, 1H), 3.60 (ddd, *J* = 25.4, 15.6, 6.2 Hz, 2H), 3.48 (dd, *J* = 16.5, 5.7 Hz, 1H), 3.41 (dd, *J* = 14.8, 6.2 Hz, 1H), 2.83 (d, *J* = 6.5 Hz, 2H), 2.64 (q, *J* = 7.6 Hz, 2H), 2.54 (d, *J* = 4.6 Hz, 3H), 2.40–2.30 (m, 2H), 1.18 (t, *J* = 7.6 Hz, 3H), 0.91 (t, *J* = 3.6 Hz, 2H), 0.79 (dd, *J* = 9.4, 3.6 Hz, 2H).

^13^C-NMR (151 MHz, DMSO): δ [ppm] = 171.6, 170.8, 169.3, 167.2, 147.5, 136.1, 131.4, 127.7, 127.6, 127.5, 123.3, 120.9, 118.6, 118.2, 111.3, 111.2, 47.8, 42.2, 42.1, 29.9, 28.1, 25.5, 25.3, 15.4, 13.2, 12.9.

LC/MS: m/z calculated for C_29_H_35_N_5_O_4_ [M + H]^+^: 518.3, found: 518.2.

**Figure:**
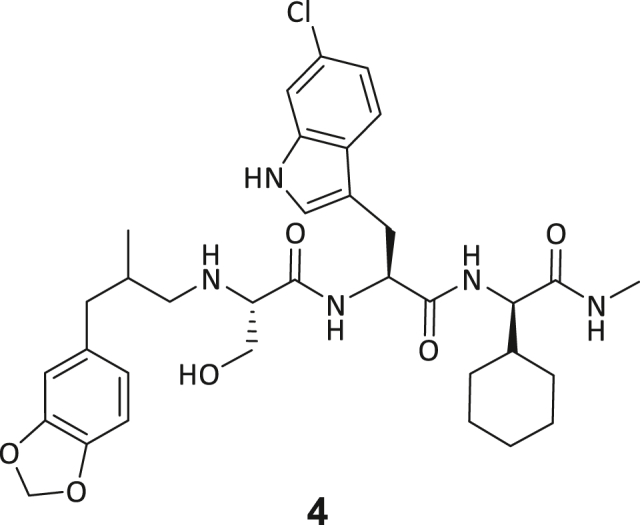

Compound **4**: ^1^H-NMR (400 MHz, DMSO-*d*_6_): δ [ppm] = 10.97 (s, 1H), 8.03 (d, *J* = 8.2 Hz, 1H), 7.90 (d, *J* = 10.1 Hz, 2H), 7.51 (d, *J* = 8.5 Hz, 1H), 7.31 (d, *J* = 6.4 Hz, 1H), 7.09 (s, 1H), 6.92 (d, *J* = 8.5 Hz, 1H), 6.76 (d, *J* = 7.8 Hz, 1H), 6.66 (s, 1H), 6.52 (d, *J* = 7.9 Hz, 1H), 5.94 (s, 2H), 4.92 (s, 1H), 4.62 (q, *J* = 6.9 Hz, 1H), 4.08 (t, *J* = 8.2 Hz, 1H), 3.47 (d, *J* = 11.1 Hz, 1H), 3.03 (ddt, *J* = 37.3, 22.9, 5.8 Hz, 3H), 2.62–2.54 (m, 4H), 2.37–1.94 (m, 4H), 1.58 (dt, *J* = 18.1, 11.0 Hz, 6H), 1.45 (d, *J* = 12.6 Hz, 1H), 1.21–0.77 (m, 6H), 0.63 (d, *J* = 6.4 Hz, 3H).

^13^C-NMR (101 MHz, DMSO): δ [ppm] = 171.8, 171.0, 170.5, 147.0, 145.1, 136.3, 134.7, 126.4, 125.5, 124.9, 121.7, 120.0, 118.4, 110.7, 109.9, 109.2, 107.8, 100.5, 64.8, 62.1, 57.1, 53.9, 52.6, 35.1, 29.1, 28.3, 27.5, 25.8, 25.5, 25.4, 17.3.

LC/MS: m/z calculated for C_34_H_44_ClN_5_O_6_ [M + H]^+^: 654.3, found: 654.3.

**Figure:**
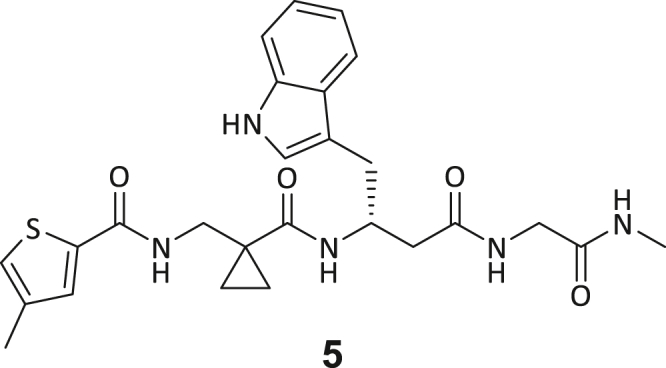

Compound **5**: ^1^H-NMR (400 MHz, DMSO-*d*_6_): δ [ppm] = 10.76 (s, 1H), 8.49 (t, *J* = 7.0 Hz, 1H), 8.10 (d, *J* = 6.6 Hz, 1H), 7.86–7.80 (m, 1H), 7.60 (q, *J* = 8.9, 7.7 Hz, 3H), 7.35 (s, 1H), 7.30 (d, *J* = 8.2 Hz, 1H), 7.09 (s, 1H), 7.07–7.00 (m, 1H), 6.99–6.92 (m, 1H), 4.41–4.30 (m, 1H), 2.82 (t, *J* = 4.7 Hz, 2H), 2.54 (d, *J* = 4.3 Hz, 4H), 2.34 (d, *J* = 7.0 Hz, 2H), 2.21 (d, *J* = 3.2 Hz, 3H), 1.24 (s, 1H), 1.03–0.85 (m, 3H), 0.76 (s, 2H).

^13^C-NMR (101 MHz, DMSO): δ [ppm] = 172.0, 171.3, 169.7, 162.4, 139.2, 138.3, 136.6, 130.9, 128.0, 126.9, 123.8, 121.3, 119.0, 118.6, 111.7, 111.6, 48.3, 42.6, 30.4, 25.9, 25.6, 23.8, 15.9, 13.4, 13.1.

LC/MS: m/z calculated for C_26_H_31_N_5_O_4_S [M + H]^+^: 510.2, found: 510.1.

**Figure:**
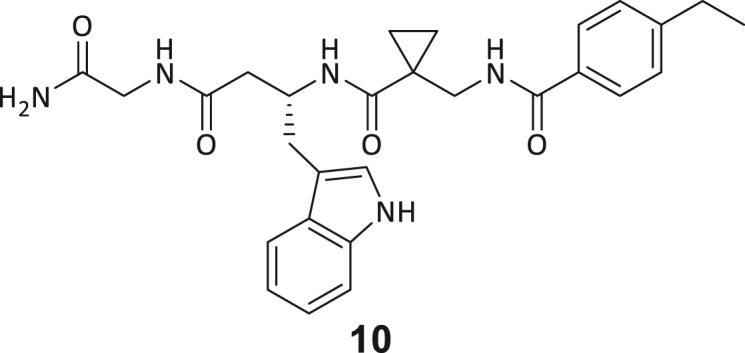

Compound **10**: Yield: 2.9 mg (5.80 μmol ≙ 13% of th.), colorless solid.

^1^H-NMR (600 MHz, DMSO-*d*_*6*_): δ [ppm] = 10.77 (s, 1H), 8.59 (t, *J* = 6.2 Hz, 1H), 8.07 (t, *J* = 5.9 Hz, 1H), 8.04 (d, *J* = 7.9 Hz, 1H), 7.81–7.71 (m, 2H), 7.59 (d, *J* = 7.9 Hz, 1H), 7.33–7.26 (m, 3H), 7.24 (s, 1H), 7.09 (d, *J* = 2.2 Hz, 1H), 7.04 (t, *J* = 7.3 Hz, 2H), 6.95 (t, *J* = 7.4 Hz, 1H), 4.34 (h, *J* = 6.9 Hz, 1H), 3.61 (dd, *J* = 16.8, 6.0 Hz, 1H), 3.56 (dd, *J* = 14.8, 6.1 Hz, 1H), 3.48 (dd, *J* = 16.8, 5.5 Hz, 1H), 3.41 (dd, *J* = 14.8, 6.1 Hz, 1H), 2.82 (d, *J* = 6.7 Hz, 2H), 2.64 (q, *J* = 7.6 Hz, 2H), 2.33 (d, *J* = 6.8 Hz, 1H), 1.18 (t, *J* = 7.6 Hz, 3H), 0.98–0.72 (m, 4H).

^13^C-NMR (151 MHz, DMSO): δ [ppm] = 171.6, 171.1, 170.6, 167.2, 147.5, 136.1, 131.4, 127.7, 127.6, 127.5, 123.3, 120.8, 118.6, 118.2, 111.3, 111.2, 47.7, 42.3, 41.9, 40.1, 29.8, 28.1, 25.3, 15.4, 13.3, 12.9.

LC/MS: m/z calculated for C_28_H_33_N_5_O_4_ [M + H]^+^: 504.3, found: 504.1.

**Figure:**
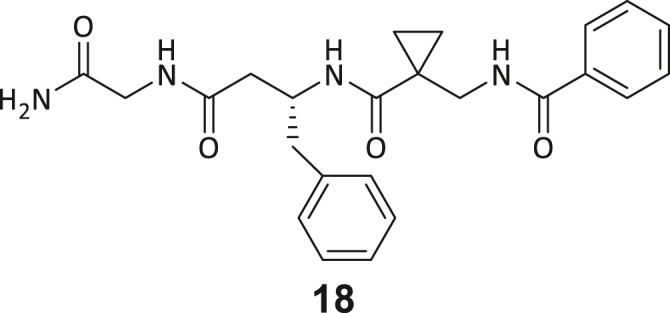

Compound **18**: Yield: 15.0 mg (0.034 mmol ≙ 75% of th.), colorless solid.

^1^H-NMR (300 MHz, DMSO-*d*_6_): δ [ppm] = 8.62 (t, *J* = 6.1 Hz, 1H), 8.10 (t, *J* = 5.9 Hz, 1H), 7.96 (d, *J* = 8.1 Hz, 1H), 7.91–7.75 (m, 2H), 7.67–7.36 (m, 3H), 7.24 (s, 1H), 7.20–7.03 (m, 5H), 7.03 (s, 1H), 4.30 (q, *J* = 7.5 Hz, 1H), 3.74–3.39 (m, 4H), 2.93–2.59 (m, 2H), 2.32 (d, *J* = 6.8 Hz, 2H), 1.04–0.56 (m, 4H).

^13^C-NMR (75 MHz, DMSO): δ [ppm] = 171.5, 171.1, 170.4, 167.2, 138.8, 134.0, 131.5, 129.2, 128.3, 128.0, 127.4, 126.0, 48.4, 42.3, 41.9, 40.3, 25.2, 13.0.

LC/MS: m/z calculated for C_24_H_28_N_4_O_4_ [M + H]^+^: 437.2, found: 437.2.

**Figure:**
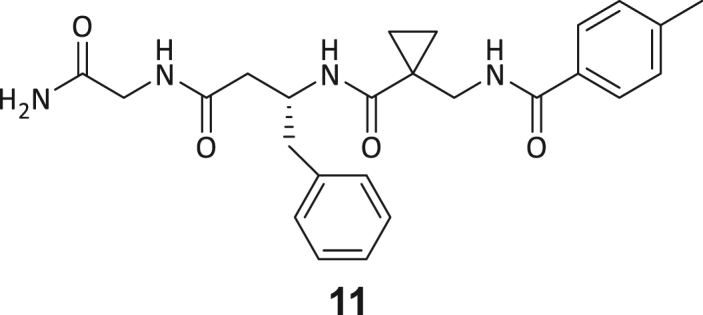

Compound **11**: Yield: 15.0 mg (0.033 mmol ≙ 75% of th.), colorless solid.

^1^H-NMR (300 MHz, DMSO-*d*_6_): δ [ppm] = 8.55 (t, *J* = 6.1 Hz, 1H), 8.09 (t, *J* = 5.9 Hz, 1H), 8.00 (d, *J* = 8.1 Hz, 1H), 7.82–7.69 (m, 2H), 7.32–7.26 (m, 2H), 7.24 (s, 1H), 7.17–7.07 (m, 5H), 7.03 (s, 1H), 4.30 (q, *J* = 7.0 Hz, 1H), 3.72–3.38 (m, 4H), 2.90–2.59 (m, 2H), 2.36 (s, 3H), 2.31 (d, *J* = 6.9 Hz, 2H), 0.96–0.68 (m, 4H).

^13^C-NMR (75 MHz, DMSO): δ [ppm] = 171.5, 171.1, 170.3, 167.1, 141.4, 138.8, 131.1, 129.2, 128.9, 128.0, 127.4, 126.0, 48.4, 42.3, 41.9, 25.3, 21.0, 13.0.

LC/MS: m/z calculated for C_25_H_30_N_4_O_4_ [M + H]^+^: 451.2, found: 451.1.

**Figure:**
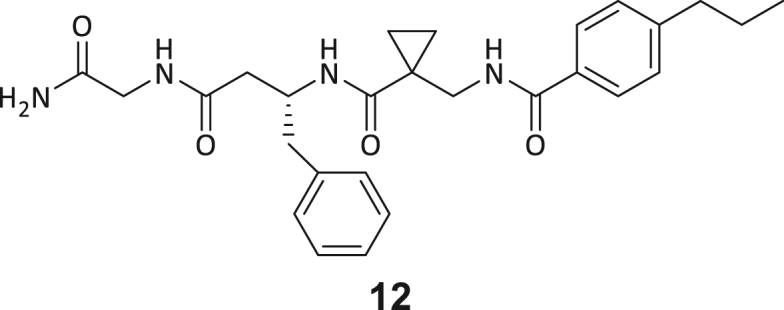

Compound **12**: Yield: 5.0 mg (0.010 mmol ≙ 23% of th.), colorless solid.

^1^H-NMR (400 MHz, DMSO-*d*_6_): δ [ppm] = 8.54 (t, *J* = 6.3 Hz, 1H), 8.08 (t, *J* = 6.0 Hz, 1H), 7.98 (d, *J* = 8.1 Hz, 1H), 7.81–7.73 (m, 2H), 7.33–7.26 (m, 2H), 7.23 (s, 1H), 7.17–7.05 (m, 5H), 7.01 (s, 1H), 4.29 (p, *J* = 7.2 Hz, 1H), 3.70–3.43 (m, 4H), 2.87–2.64 (m, 2H), 2.61 (t, *J* = 7.7 Hz, 2H), 2.31 (d, *J* = 6.9 Hz, 2H), 1.61 (h, *J* = 7.1 Hz, 2H), 0.93–0.72 (m, 7H).

^13^C-NMR (101 MHz, DMSO): δ [ppm] = 171.5, 171.1, 170.3, 167.2, 145.9, 138.8, 131.4, 129.1, 128.3, 127.9, 127.4, 125.9, 48.4, 42.3, 41.9, 40.3, 37.0, 25.2, 23.9, 13.6, 13.0, 13.0.

LC/MS: m/z calculated for C_27_H_34_N_4_O_4_ [M + H]^+^: 479.3, found: 479.2.

**Figure:**
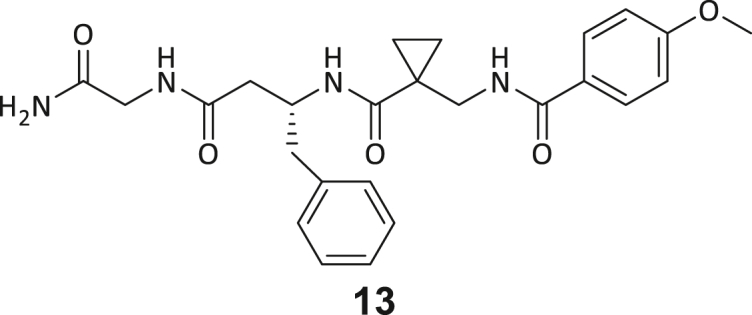

Compound **13**: Yield: 18.0 mg (0.040 mmol ≙ 86% of th.), colorless solid.

^1^H-NMR (300 MHz, DMSO-*d*_6_): δ [ppm] = 8.50 (t, *J* = 6.1 Hz, 1H), 8.16–7.98 (m, 2H), 7.91–7.76 (m, 2H), 7.24 (s, 1H), 7.20–7.07 (m, 4H), 7.06–6.91 (m, 3H), 4.29 (q, *J* = 8.5 Hz, 1H), 3.82 (s, 3H), 3.72–3.40 (m, 4H), 2.90–2.58 (m, 2H), 2.31 (d, *J* = 6.8 Hz, 2H), 0.98–0.61 (m, 4H).

^13^C-NMR (75 MHz, DMSO): δ [ppm] = 171.5, 171.1, 170.4, 166.8, 161.8, 138.8, 129.3, 129.2, 128.0, 126.1, 126.0, 113.5, 55.4, 48.4, 42.3, 41.9, 25.3, 13.1.

LC/MS: m/z calculated for C_25_H_30_N_4_O_5_ [M + H]^+^: 467.2, found: 467.1.

**Figure:**
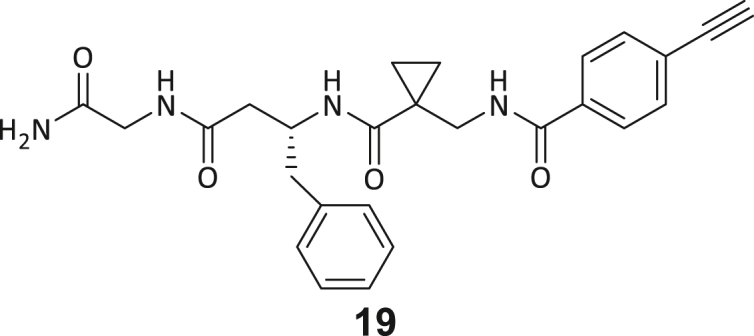

Compound **19**: Yield: 15.0 mg (0.033 mmol ≙ 71% of th.), pale brown solid.

^1^H-NMR (300 MHz, DMSO-*d*_6_): δ [ppm] = 8.69 (t, *J* = 6.1 Hz, 1H), 8.10 (t, *J* = 5.8 Hz, 1H), 7.96–7.76 (m, 3H), 7.59 (d, *J* = 8.2 Hz, 2H), 7.24 (s, 1H), 7.20–7.06 (m, 5H), 7.03 (s, 1H), 4.39 (s, 1H), 4.30 (q, *J* = 8.2 Hz, 1H), 3.70–3.38 (m, 4H), 2.86–2.57 (m, 2H), 2.32 (d, *J* = 6.8 Hz, 2H), 0.96–0.61 (m, 4H).

^13^C-NMR (75 MHz, DMSO): δ [ppm] = 171.4, 171.1, 170.4, 166.4, 138.8, 134.0, 131.7, 129.1, 128.0, 127.7, 126.0, 124.7, 83.0, 82.9, 48.4, 42.5, 41.9, 40.2, 25.1, 12.9.

LC/MS: m/z calculated for C_26_H_28_N_4_O_4_ [M + H]^+^: 461.2, found: 461.1.

**Figure:**
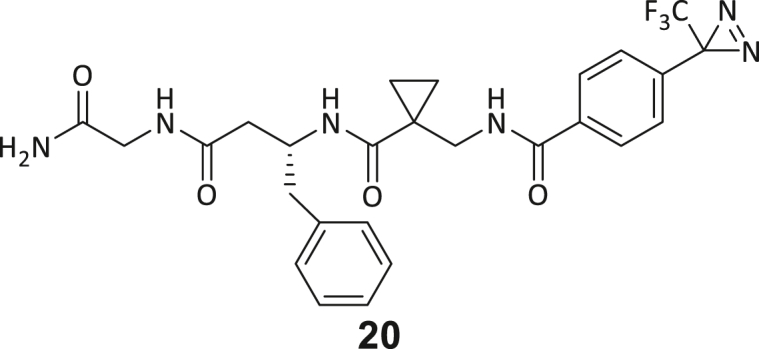

Compound **20**: Yield: 17.0 mg (0.031 mmol ≙ 69% of th.), colorless solid.

^1^H-NMR (300 MHz, DMSO-*d*_6_): δ [ppm] = 8.74 (t, *J* = 6.0 Hz, 1H), 8.09 (t, *J* = 5.9 Hz, 1H), 8.01–7.89 (m, 2H), 7.82 (d, *J* = 8.2 Hz, 1H), 7.39 (d, *J* = 8.1 Hz, 2H), 7.23 (s, 1H), 7.18–7.03 (m, 4H), 7.03 (s, 1H), 4.29 (p, *J* = 7.1 Hz, 1H), 3.80–3.39 (m, 4H), 2.94–2.57 (m, 2H), 2.31 (d, *J* = 6.8 Hz, 2H), 0.99–0.69 (m, 4H).

^13^C-NMR (75 MHz, DMSO): δ [ppm] = 171.4, 171.1, 170.4, 166.1, 138.8, 135.6, 130.4, 129.1, 128.3, 127.9, 126.4, 125.9, 123.1, 120.4, 48.3, 42.5, 41.9, 40.2, 39.4, 28.3, 27.9, 25.1, 12.8.

LC/MS: m/z calculated for C_26_H_27_F_3_N_6_O_4_ [M + H]^+^: 545.2, found: 545.1.

**Figure:**
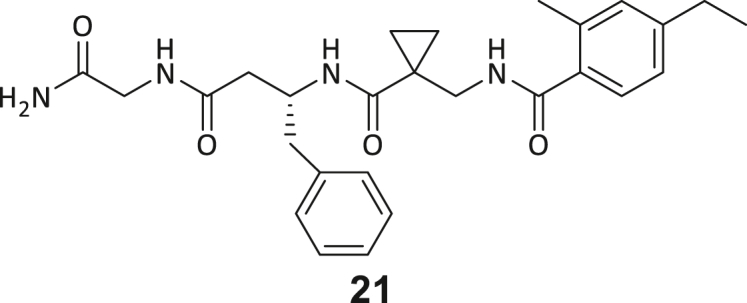

Compound **21**: Yield: 10.1 mg (0.021 mmol ≙ 46% of th.), colorless solid.

^1^H-NMR (300 MHz, DMSO-*d*_6_): δ [ppm] = 8.36 (t, *J* = 6.2 Hz, 1H), 8.09 (t, *J* = 5.8 Hz, 1H), 7.93 (d, *J* = 8.1 Hz, 1H), 7.30–7.11 (m, 7H), 7.11–6.92 (m, 3H), 4.40–4.20 (m, 1H), 3.69–3.45 (m, 3H), 3.43–3.25 (m, 1H), 2.74 (qd, *J* = 13.5, 6.8 Hz, 2H), 2.58 (q, *J* = 7.6 Hz, 2H), 2.39–2.16 (m, 5H), 1.17 (t, *J* = 7.6 Hz, 3H), 0.99–0.62 (m, 4H).

^13^C-NMR (75 MHz, DMSO): δ [ppm] = 171.6, 171.1, 170.3, 170.0, 145.4, 138.9, 135.3, 134.0, 130.0, 129.2, 128.1, 127.4, 126.0, 124.9, 48.6, 41.9, 40.4, 28.0, 25.2, 19.6, 15.6, 12.9, 12.8.

LC/MS: m/z calculated for C_27_H_34_N_4_O_4_ [M + H]^+^: 479.3, found: 479.2.

**Figure:**
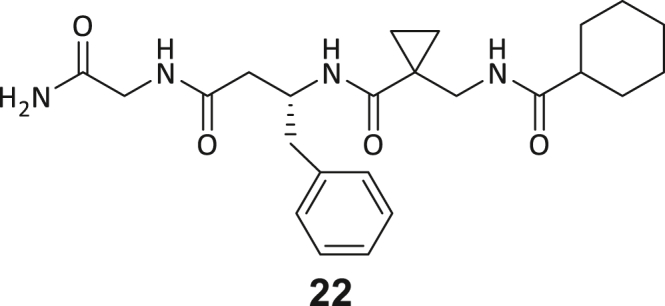

Compound **22**: Yield: 16.0 mg (0.036 mmol ≙ 79% of th.), colorless solid.

^1^H-NMR (300 MHz, DMSO-*d*_6_): δ [ppm] = 8.09 (t, *J* = 5.8 Hz, 1H), 7.96 (t, *J* = 6.2 Hz, 1H), 7.86 (d, *J* = 8.1 Hz, 1H), 7.34–7.09 (m, 6H), 7.02 (s, 1H), 4.38–4.10 (m, 1H), 3.60 (qd, *J* = 16.8, 5.8 Hz, 2H), 3.45–3.21 (m, 1H), 3.12 (dd, *J* = 14.7, 6.1 Hz, 1H), 2.89–2.56 (m, 2H), 2.27 (d, *J* = 6.9 Hz, 2H), 2.19–1.98 (m, 1H), 1.80–1.47 (m, 5H), 1.47–1.02 (m, 5H), 0.91–0.70 (m, 2H), 0.69–0.55 (m, 2H).

^13^C-NMR (75 MHz, DMSO): δ [ppm] = 176.3, 171.5, 171.1, 170.3, 138.9, 129.2, 128.1, 126.0, 48.4, 43.9, 41.9, 41.3, 40.3, 29.2, 29.1, 25.4, 25.3, 25.1, 12.9, 12.8.

LC/MS: m/z calculated for C_24_H_34_N_4_O_4_ [M + H]^+^: 443.3, found: 443.2.

**Figure:**
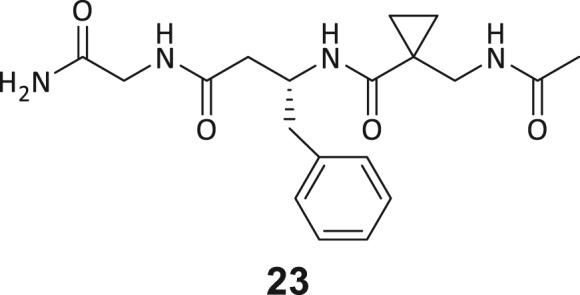

Compound **23**: Yield: 7.2 mg (0.018 mmol ≙ 41% of th.), colorless solid.

^1^H-NMR (300 MHz, DMSO-*d*_6_): δ [ppm] = 8.08 (q, *J* = 5.9 Hz, 2H), 7.78 (d, *J* = 8.0 Hz, 1H), 7.42–7.10 (m, 6H), 7.03 (s, 1H), 4.25 (p, *J* = 6.9 Hz, 1H), 3.80–3.46 (m, 2H), 3.22 (qd, *J* = 14.8, 6.2 Hz, 2H), 2.93–2.56 (m, 2H), 2.29 (d, *J* = 6.9 Hz, 2H), 1.80 (s, 3H), 1.01–0.72 (m, 2H), 0.71–0.56 (m, 2H).

^13^C-NMR (75 MHz, DMSO): δ [ppm] = 171.4, 171.1, 170.3, 170.3, 138.8, 129.2, 128.1, 126.0, 48.3, 41.9, 41.7, 40.0, 39.4, 25.1, 22.5, 12.9.

LC/MS: m/z calculated for C_19_H_26_N_4_O_4_ [M + H]^+^: 375.2, found: 375.2.

**Figure:**
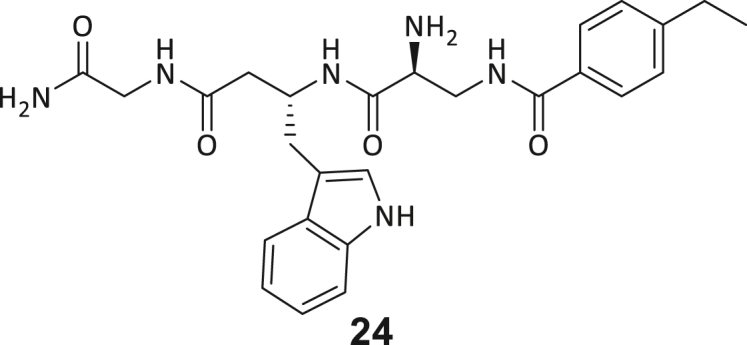

Compound **24**: Yield: 5.0 mg (0.010 mmol ≙ 23% of th.), colorless solid.

^1^H-NMR (400 MHz, DMSO-*d*_6_): δ [ppm] = 10.82 (s, 1H), 8.49 (d, *J* = 8.0 Hz, 1H), 8.41 (t, *J* = 5.7 Hz, 1H), 8.21 (s, 3H), 8.02 (t, *J* = 5.8 Hz, 1H), 7.76 (d, *J* = 7.9 Hz, 2H), 7.59 (d, *J* = 7.9 Hz, 1H), 7.43–7.21 (m, 4H), 7.13 (s, 1H), 7.10–7.00 (m, 2H), 6.95 (t, *J* = 7.4 Hz, 1H), 4.35 (q, *J* = 7.0 Hz, 1H), 3.94 (t, *J* = 6.1 Hz, 1H), 3.63 (d, *J* = 5.7 Hz, 2H), 3.59 (t, *J* = 5.9 Hz, 2H), 2.83 (m, 2H), 2.65 (q, J = 7.5 Hz, 2H), 2.44–2.29 (m, 2H), 1.18 (t, *J* = 7.6 Hz, 3H).

^13^C-NMR (101 MHz, DMSO): δ [ppm] = 171.1, 170.3, 167.1, 166.0, 147.6, 136.2, 131.3, 127.6, 127.5, 127.4, 123.5, 120.9, 118.5, 118.2, 111.3, 110.7, 52.3, 47.8, 41.7, 40.2, 39.0, 29.9, 28.0, 15.4.

LC/MS: m/z calculated for C_26_H_32_N_6_O_4_ [M + H]^+^: 493.3, found: 493.2.

**Figure:**
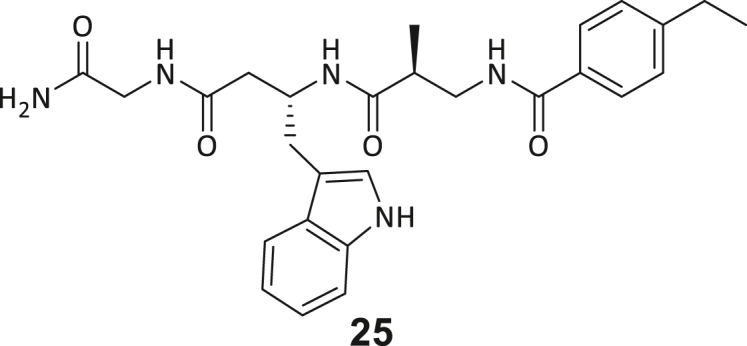

Compound **25**: Yield: 1.2 mg (2.520 μmol ≙ 1% of th.), colorless solid.

^1^H-NMR (400 MHz, DMSO-*d*_6_): δ [ppm] = 10.75 (s, 1H), 8.33 (t, *J* = 5.8 Hz, 1H), 7.96 (t, *J* = 5.8 Hz, 1H), 7.78 (d, *J* = 8.2 Hz, 1H), 7.73 (d, *J* = 7.9 Hz, 2H), 7.58 (d, *J* = 7.9 Hz, 1H), 7.31 (d, *J* = 8.1 Hz, 1H), 7.25 (d, *J* = 7.5 Hz, 3H), 7.09 (s, 1H), 7.07–6.99 (m, 2H), 6.94 (t, *J* = 7.4 Hz, 1H), 4.32 (q, *J* = 7.0 Hz, 1H), 3.69–3.48 (m, 2H), 3.28–3.12 (m, 2H), 2.81 (m, 2H), 2.69–2.53 (m, 3H), 2.38–2.16 (m, 2H), 1.21–1.09 (m, 3H), 0.98 (d, *J* = 6.8 Hz, 3H).

^13^C-NMR (101 MHz, DMSO): δ [ppm] = 173.7, 171.1, 170.6, 166.3, 147.0, 136.1, 132.1, 127.6, 127.6, 127.2, 123.3, 120.8, 118.6, 118.2, 111.3, 111.1, 47.1, 42.5, 41.9, 30.2, 28.0, 15.6, 15.4.

LC/MS: m/z calculated for C_27_H_33_N_5_O_4_ [M + H]^+^: 492.3, found: 492.1.

**Figure:**
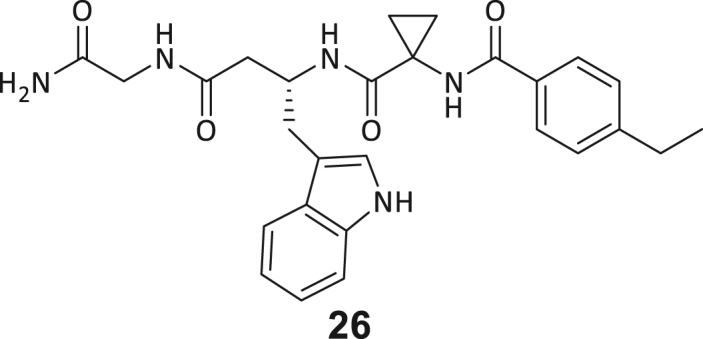

Compound **26**: Yield: 5.0 mg (0.010 mmol ≙ 23% of th.), colorless solid.

^1^H-NMR (400 MHz, DMSO-*d*_6_): δ [ppm] = 10.81 (s, 1H), 8.85 (s, 1H), 7.94 (t, *J* = 5.9 Hz, 1H), 7.87–7.73 (m, 3H), 7.60 (d, *J* = 7.9 Hz, 1H), 7.42–7.27 (m, 3H), 7.25 (s, 1H), 7.16 (s, 1H), 7.09–6.97 (m, 2H), 6.94 (t, *J* = 7.4 Hz, 1H), 4.30 (h, *J* = 6.6 Hz, 1H), 3.61 (t, *J* = 5.2 Hz, 2H), 2.99–2.71 (m, 2H), 2.66 (q, *J* = 7.6 Hz, 2H), 2.29 (ddd, *J* = 48.3, 14.5, 6.2 Hz, 2H), 1.44–1.28 (m, 2H), 1.26–1.10 (m, 3H), 1.06–0.86 (m, 2H).

^13^C-NMR (101 MHz, DMSO): δ [ppm] = 171.1, 170.8, 170.8, 167.4, 147.4, 136.1, 131.8, 127.8, 127.5, 127.5, 123.6, 120.8, 118.5, 118.2, 111.2, 110.9, 41.8, 38.8, 34.7, 29.7, 28.1, 16.2, 15.5.

LC/MS: m/z calculated for C_27_H_31_N_5_O_4_ [M + H]^+^: 490.2, found: 490.1.

**Figure:**
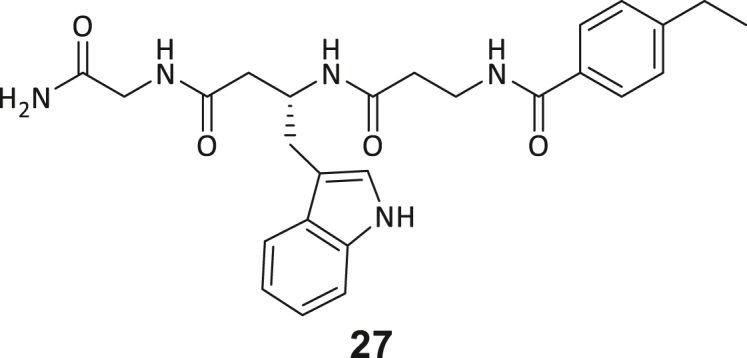

Compound **27**: Yield: 11.0 mg (0.023 mmol ≙ 50% of th.), colorless solid.

^1^H-NMR (300 MHz, DMSO-*d*_6_): δ [ppm] = 10.79 (d, *J* = 2.4 Hz, 1H), 8.38 (t, *J* = 5.6 Hz, 1H), 8.03 (t, *J* = 5.8 Hz, 1H), 7.87 (d, *J* = 8.2 Hz, 1H), 7.79–7.66 (m, 2H), 7.58 (d, *J* = 7.8 Hz, 1H), 7.37–7.19 (m, 4H), 7.11 (d, *J* = 2.2 Hz, 1H), 7.09–7.00 (m, 2H), 7.00–6.90 (m, 1H), 4.33 (q, *J* = 7.2 Hz, 1H), 3.69–3.50 (m, 3H), 3.49–3.29 (m, 2H), 2.83 (d, *J* = 6.5 Hz, 2H), 2.64 (q, *J* = 7.6 Hz, 2H), 2.42–2.18 (m, 3H), 1.18 (t, *J* = 7.6 Hz, 3H).

^13^C-NMR (75 MHz, DMSO): δ [ppm] = 171.1, 170.6, 169.9, 166.1, 147.1, 136.1, 132.0, 127.6, 127.2, 123.4, 120.8, 118.6, 118.2, 111.3, 111.1, 47.2, 41.9, 40.0, 36.1, 35.7, 29.8, 28.0, 15.4.

LC/MS: m/z calculated for C_26_H_31_N_5_O_4_ [M + H]^+^: 478.2, found: 478.1.

**Figure:**
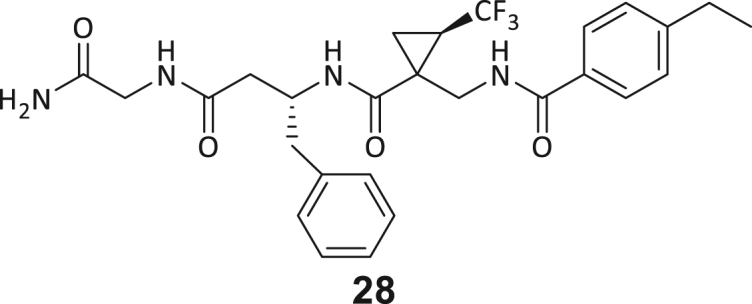

Compound **28**: Yield: 23.2 mg (0.040 mmol ≙ 87% of th.), colorless solid.

^1^H-NMR (300 MHz, DMSO-*d*_6_): δ [ppm] = 8.51 (s, 1H), 8.15 (d, *J* = 7.5 Hz, 1H), 7.81–7.74 (m, 2H), 7.37–7.13 (m, 6H), 7.07 (s, 4H), 3.70 (s, 4H), 3.63–3.56 (m, 2H), 2.72–2.58 (m, 3H), 2.41–2.29 (m, 2H), 2.05–1.83 (m, 1H), 1.48–1.28 (m, 2H), 1.19 (ddd, *J* = 7.5, 5.1, 2.7 Hz, 3H).

^13^C-NMR (75 MHz, DMSO): δ [ppm] = 244.5, 237.0, 214.3, 204.3, 166.2, 148.9, 147.6, 138.2, 131.3, 129.3, 129.1, 128.0, 127.9, 127.7, 127.5, 127.5, 40.3, 40.0, 39.1, 38.8, 38.5, 28.1, 15.4.

LC/MS: m/z calculated for C_27_H_31_F_3_N_4_O_4_ [M + H]^+^: 533.2, found: 533.2.

**Figure:**
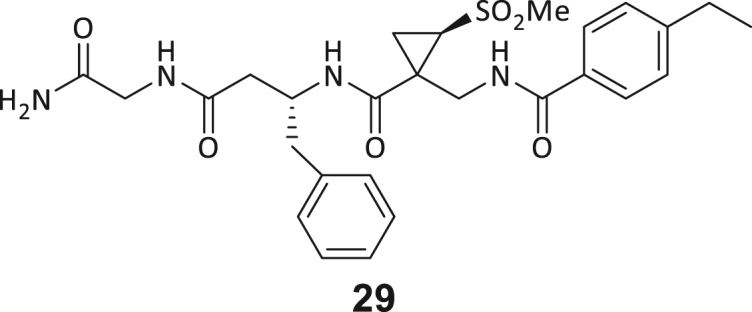

Compound **29**: Yield: 19.5 mg (0.036 mmol ≙ 72% of th.), colorless solid.

^1^H-NMR (300 MHz, DMSO-*d*_6_): δ [ppm] = 8.58–8.41 (m, 1H), 8.26 (t, *J* = 8.8 Hz, 1H), 8.12 (q, *J* = 5.8 Hz, 1H), 7.79 (d, *J* = 7.3 Hz, 2H), 7.36–7.09 (m, 6H), 7.04 (s, 3H), 4.33–4.16 (m, 5H), 3.83 (ddd, *J* = 30.8, 14.8, 5.2 Hz, 1H), 3.64–3.48 (m, 2H), 3.14 (s, 1H), 3.00–2.80 (m, 1H), 2.66 (qd, *J =* 7.4, 3.3 Hz, 3H), 2.41–2.26 (m, 2H), 1.73–1.62 (m, 2H), 1.19 (td, *J* = 7.6, 2.7 Hz, 3H).

^13^C-NMR (75 MHz, DMSO): δ [ppm] = 171.1, 171.1, 170.3, 170.2, 167.8, 167.6, 167.3, 167.2, 147.8, 147.7, 138.6, 138.5, 131.2, 131.2, 129.3, 129.1, 128.1, 127.9, 127.7, 127.6, 127.5, 42.8, 42.8, 32.9, 32.8, 28.1, 15.4.

LC/MS: m/z calculated for C_27_H_34_N_4_O_6_S [M + H]^+^: 543.2, found: 543.2.

**Figure:**
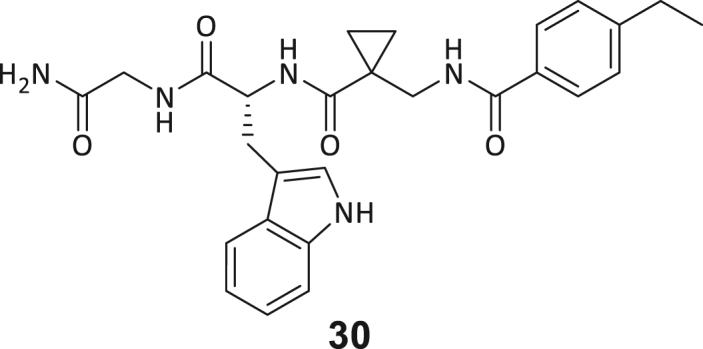

Compound **30**: Yield: 24.0 mg (0.049 mmol ≙ quant.), colorless solid.

^1^H-NMR (300 MHz, DMSO-*d*_6_): δ [ppm] = 10.77 (d, *J* = 2.4 Hz, 1H), 8.63 (t, *J* = 6.1 Hz, 1H), 8.44 (d, *J* = 6.7 Hz, 1H), 8.32 (t, *J* = 5.9 Hz, 1H), 7.80–7.67 (m, 2H), 7.60 (d, *J* = 7.7 Hz, 1H), 7.35–7.23 (m, 3H), 7.20 (d, *J* = 2.3 Hz, 1H), 7.14–7.01 (m, 3H), 6.97 (ddd, *J* = 8.0, 6.9, 1.1 Hz, 1H), 4.41 (dt, *J* = 9.4, 6.3 Hz, 1H), 3.74–3.56 (m, 2H), 3.56–3.44 (m, 2H), 3.17 (dd, *J* = 14.7, 4.8 Hz, 1H), 3.01 (dd, *J* = 14.7, 9.3 Hz, 1H), 2.65 (q, *J* = 7.6 Hz, 2H), 1.19 (t, *J* = 7.6 Hz, 3H), 0.98–0.71 (m, 4H).

^13^C-NMR (75 MHz, DMSO): δ [ppm] = 172.9, 172.1, 171.1, 167.4, 147.5, 136.1, 131.4, 127.7, 127.5, 127.3, 123.6, 120.9, 118.4, 118.3, 111.4, 110.3, 54.8, 42.3, 42.2, 28.1, 27.1, 25.2, 15.4, 13.6.

LC/MS: m/z calculated for C_27_H_31_N_5_O_4_ [M + H]^+^: 490.2, found: 490.1.

**Figure:**
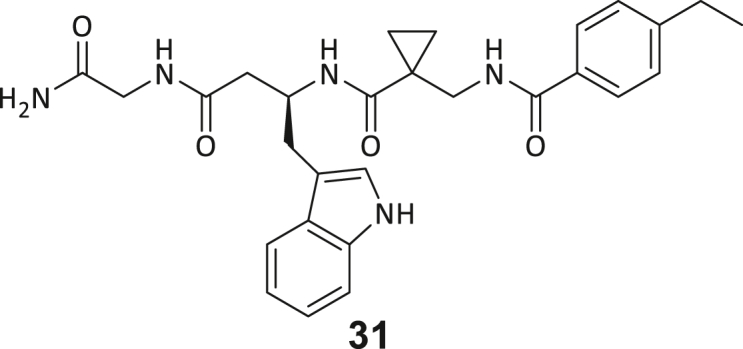

Compound **31**: Yield: 8.0 mg (0.016 mmol ≙ 35% of th.), colorless solid.

^1^H-NMR (300 MHz, DMSO-*d*_6_): δ [ppm] = 10.76 (d, *J* = 2.4 Hz, 1H), 8.57 (t, *J* = 6.1 Hz, 1H), 8.16–7.90 (m, 2H), 7.84–7.68 (m, 2H), 7.60 (d, *J* = 7.8 Hz, 1H), 7.35–7.25 (m, 3H), 7.23 (s, 1H), 7.09 (d, *J* = 2.2 Hz, 1H), 7.07–6.98 (m, 2H), 6.98–6.88 (m, 1H), 4.34 (q, *J* = 6.9 Hz, 1H), 3.72–3.36 (m, 4H), 2.83 (d, *J* = 6.5 Hz, 2H), 2.65 (q, *J* = 7.6 Hz, 2H), 2.34 (d, *J* = 6.8 Hz, 2H), 1.19 (t, *J* = 7.6 Hz, 3H), 1.06–0.59 (m, 4H).

^13^C-NMR (75 MHz, DMSO): δ [ppm] = 171.5, 171.1, 170.6, 167.2, 147.5, 136.1, 131.5, 127.7, 127.6, 127.4, 123.3, 120.8, 118.6, 118.2, 111.3, 111.2, 47.7, 42.3, 41.9, 39.9, 29.8, 28.1, 25.3, 15.4, 13.2, 12.9.

LC/MS: m/z calculated for C_28_H_33_N_5_O_4_ [M + H]^+^: 504.3, found: 504.1.

**Figure:**
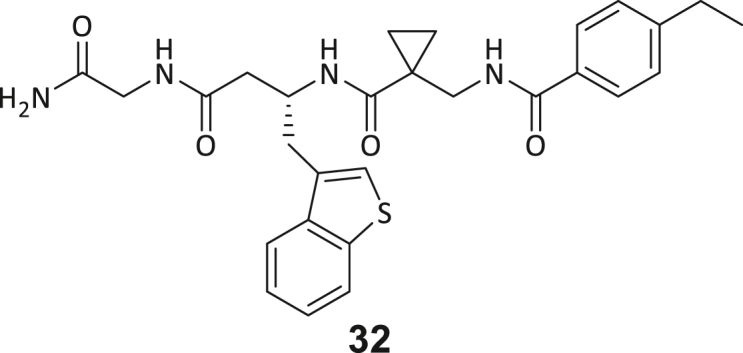

Compound **32**: Yield: 10.0 mg (0.019 mmol ≙ 43% of th.), colorless solid.

^1^H-NMR (300 MHz, DMSO-*d*_6_): δ [ppm] = 8.58 (t, *J* = 6.1 Hz, 1H), 8.22–8.05 (m, 2H), 8.02–7.93 (m, 1H), 7.93–7.85 (m, 1H), 7.81–7.68 (m, 2H), 7.47–7.18 (m, 6H), 7.03 (s, 1H), 4.42 (q, *J* = 7.0 Hz, 1H), 3.75–3.43 (m, 4H), 3.00 (qd, *J* = 14.2, 6.6 Hz, 2H), 2.65 (q, *J* = 7.5 Hz, 2H), 2.41 (d, *J* = 6.9 Hz, 2H), 1.19 (t, *J* = 7.6 Hz, 3H), 0.98–0.68 (m, 4H).

^13^C-NMR (75 MHz, DMSO): δ [ppm] = 171.7, 171.0, 170.4, 167.2, 147.5, 139.6, 139.0, 133.2, 131.4, 127.7, 127.4, 124.2, 124.0, 123.3, 122.8, 122.1, 46.7, 42.3, 41.9, 40.2, 32.8, 28.1, 25.3, 15.4, 13.1.

LC/MS: m/z calculated for C_28_H_32_N_4_O_4_S [M + H]^+^: 521.2, found: 521.1.

**Figure:**
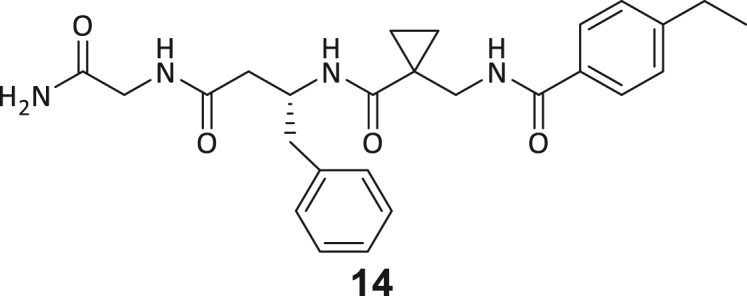

Compound **14**: Yield: 16.0 mg (0.034 mmol ≙ 77% of th.), colorless solid.

^1^H-NMR (600 MHz, DMSO-*d*_6_): δ [ppm] = 8.58 (t, *J* = 6.2 Hz, 1H), 8.11 (t, *J* = 5.9 Hz, 1H), 8.01 (d, *J* = 8.2 Hz, 1H), 7.82–7.74 (m, 2H), 7.32 (d, *J* = 8.2 Hz, 2H), 7.25 (s, 1H), 7.15–7.12 (m, 4H), 7.12–7.07 (m, 1H), 7.04 (s, 1H), 4.33–4.22 (m, 1H), 3.61 (dd, *J* = 16.8, 6.0 Hz, 1H), 3.54–3.45 (m, 3H), 2.78 (dd, *J* = 13.6, 5.1 Hz, 1H), 2.68–2.63 (m, 3H), 2.30 (d, *J* = 6.9 Hz, 2H), 1.19 (t, *J* = 7.6 Hz, 3H), 0.92–0.80 (m, 2H), 0.80–0.71 (m, 2H).

^13^C-NMR (151 MHz, DMSO): δ [ppm] = 171.5, 171.1, 170.4, 167.2, 147.6, 138.9, 131.4, 129.2, 128.0, 127.7, 127.5, 126.0, 48.5, 42.3, 41.9, 40.3, 40.1, 28.1, 25.3, 15.5, 13.1.

LC/MS: m/z calculated for C_26_H_32_N_4_O_4_ [M + H]^+^: 465.3, found: 465.1.

**Figure:**
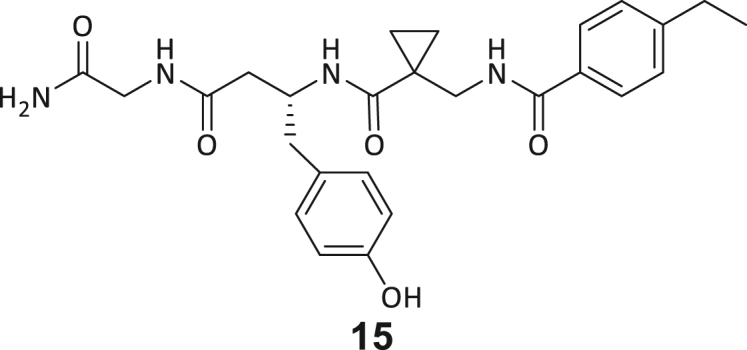

Compound **15**: Yield: 15.0 mg (0.031 mmol ≙ 69% of th.), colorless solid.

^1^H-NMR (600 MHz, DMSO-*d*_6_): δ [ppm] = 9.11 (s, 1H), 8.57 (t, *J* = 6.1 Hz, 1H), 8.05 (t, *J* = 5.8 Hz, 1H), 7.95 (d, *J* = 8.1 Hz, 1H), 7.85–7.71 (m, 2H), 7.41–7.27 (m, 2H), 7.22 (s, 1H), 7.02 (s, 1H), 6.97–6.86 (m, 2H), 6.64–6.49 (m, 2H), 4.21 (h, *J* = 7.3 Hz, 1H), 3.69–3.44 (m, 4H), 2.74–2.53 (m, 4H), 2.27 (d, *J* = 6.8 Hz, 2H), 1.19 (t, *J* = 7.6 Hz, 3H), 0.96–0.60 (m, 4H).

^13^C-NMR (151 MHz, DMSO): δ [ppm] = 171.5, 171.1, 170.4, 167.2, 155.5, 147.6, 131.4, 130.0, 128.9, 127.7, 127.5, 114.8, 48.7, 42.3, 41.9, 40.2, 38.8, 28.1, 25.2, 15.4, 13.0.

LC/MS: m/z calculated for C_26_H_32_N_4_O_5_ [M + H]^+^: 481.2, found: 481.1.

**Figure:**
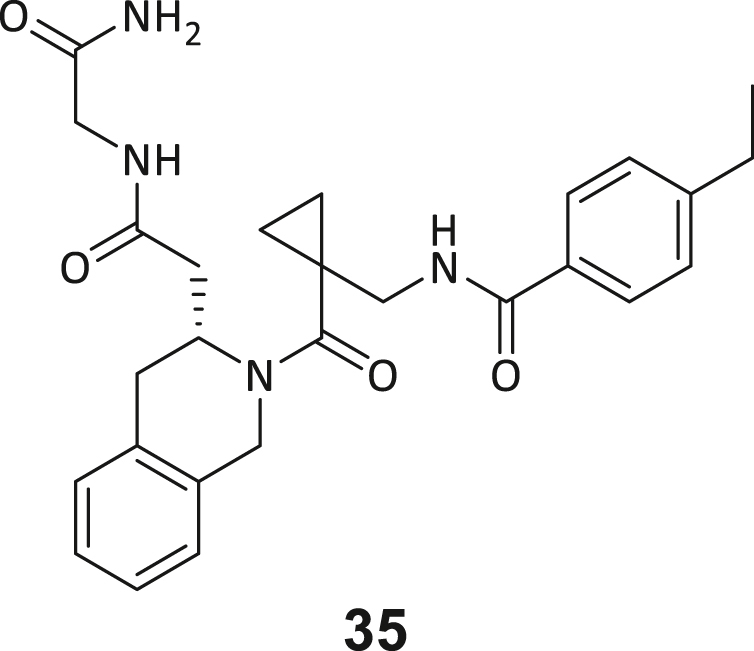

Compound **35**: Yield: 11.0 mg (0.023 mmol ≙ 50% of th.), colorless solid.

^1^H-NMR (400 MHz, DMSO-*d*_6_): δ [ppm] = 8.42 (t, *J* = 6.1 Hz, 1H), 8.08 (t, *J* = 6.0 Hz, 1H), 7.70 (d, *J* = 7.8 Hz, 2H), 7.26 (d, *J* = 7.9 Hz, 2H), 7.24–7.18 (m, 1H), 7.18–7.08 (m, 4H), 7.00 (s, 1H), 5.39–4.83 (m, 2H), 4.58 (s, 1H), 3.78–3.50 (m, 3H), 2.82–2.67 (m, 1H), 2.63 (q, *J* = 7.6 Hz, 3H), 2.45–2.23 (m, 2H), 1.18 (t, *J* = 7.6 Hz, 3H), 0.93–0.49 (m, 4H).

^13^C-NMR (101 MHz, DMSO): δ [ppm] = 170.9, 170.2, 170.1, 166.5, 147.2, 131.8, 127.6, 127.3, 126.6, 126.0, 41.9, 37.0, 32.0, 28.0, 26.0, 15.4, 9.8, 9.5.

LC/MS: m/z calculated for C_27_H_32_N_4_O_4_ [M + H]^+^: 477.3, found: 477.1.

**Figure:**
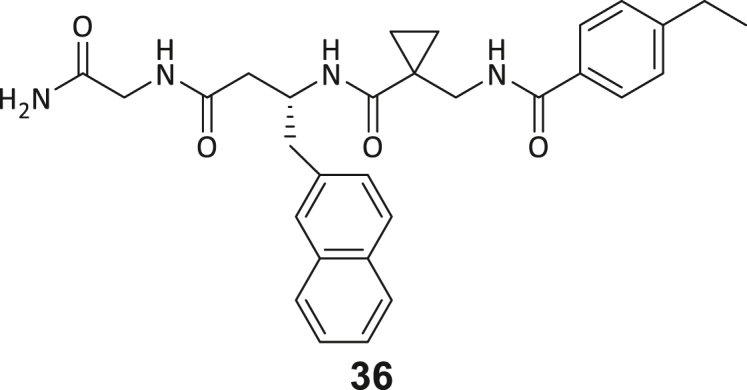

Compound **36**: Yield: 19.0 mg (0.037 mmol ≙ 80% of th.), colorless solid.

^1^H-NMR (300 MHz, DMSO-*d*_6_): δ [ppm] = 8.59 (t, *J* = 6.1 Hz, 1H), 8.23–8.03 (m, 2H), 7.88–7.72 (m, 3H), 7.69 (d, *J* = 8.4 Hz, 1H), 7.64–7.49 (m, 2H), 7.50–7.36 (m, 2H), 7.36–7.27 (m, 3H), 7.25 (s, 1H), 7.04 (s, 1H), 4.41 (p, *J* = 6.9 Hz, 1H), 3.77–3.46 (m, 3H), 3.46–3.26 (m, 1H), 2.98 (dd, *J* = 13.6, 4.9 Hz, 1H), 2.91–2.75 (m, 1H), 2.67 (q, *J* = 7.6 Hz, 2H), 2.43–2.28 (m, 2H), 1.21 (t, *J* = 7.6 Hz, 3H), 0.98–0.66 (m, 4H).

^13^C-NMR (75 MHz, DMSO): δ [ppm] = 171.5, 171.1, 170.3, 167.2, 147.6, 136.6, 132.9, 131.6, 131.3, 127.9, 127.7, 127.5, 127.4, 127.4, 127.3, 127.2, 125.8, 125.2, 48.4, 42.3, 41.9, 40.5, 39.7, 28.1, 25.3, 15.4, 13.3, 13.0.

LC/MS: m/z calculated for C_30_H_34_N_4_O_4_ [M + H]^+^: 515.3, found: 515.2.

**Figure:**
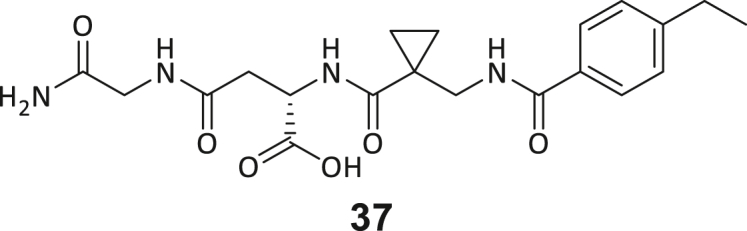

Compound **37**: Yield: 5.0 mg (0.012 mmol ≙ 26% of th.), colorless solid.

^1^H-NMR (600 MHz, DMSO-*d*_6_): δ [ppm] = 8.60 (t, *J* = 6.1 Hz, 1H), 8.26–8.20 (m, 2H), 7.81–7.74 (m, 2H), 7.35 (s, 1H), 7.30 (d, *J* = 8.1 Hz, 2H), 7.08 (s, 1H), 4.49 (q, *J* = 7.0 Hz, 1H), 3.62 (dd, *J* = 16.9, 6.1 Hz, 1H), 3.58–3.50 (m, 2H), 3.47 (dd, *J* = 16.9, 5.6 Hz, 1H), 2.71–2.60 (m, 3H), 1.19 (t, *J* = 7.6 Hz, 4H), 0.99–0.91 (m, 2H), 0.87–0.78 (m, 2H).

^13^C-NMR (151 MHz, DMSO): δ [ppm] = 173.1, 172.2, 172.1, 171.2, 169.6, 167.3, 147.5, 131.5, 127.7, 127.6, 49.8, 42.1, 42.1, 40.1, 37.1, 28.1, 24.9, 15.5, 13.3, 13.0.

LC/MS: m/z calculated for C_20_H_26_N_4_O_6_ [M + H]^+^: 419.2, found: 419.1.

**Figure:**
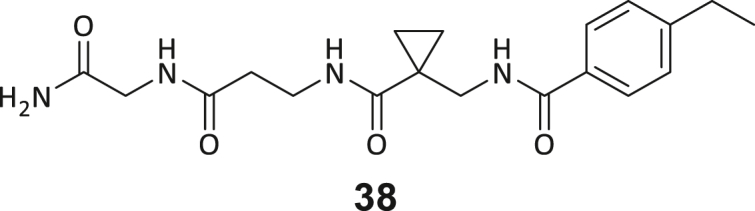

Compound **38**: Yield: 5.0 mg (0.013 mmol ≙ 29% of th.), colorless solid.

^1^H-NMR (600 MHz, DMSO-*d*_6_): δ [ppm] = 8.60 (t, *J* = 6.1 Hz, 1H), 8.10 (dt, *J* = 15.2, 5.6 Hz, 2H), 7.82–7.75 (m, 2H), 7.36–7.26 (m, 3H), 7.05 (s, 1H), 3.58 (d, *J* = 5.8 Hz, 2H), 3.51 (d, *J* = 6.0 Hz, 2H), 3.28 (td, *J* = 7.2, 5.3 Hz, 2H), 2.65 (q, *J* = 7.6 Hz, 2H), 2.30 (t, *J* = 7.3 Hz, 2H), 1.18 (t, *J* = 7.6 Hz, 3H), 0.95 (q, *J* = 3.7 Hz, 2H), 0.81 (q, *J* = 3.7 Hz, 2H).

^13^C-NMR (151 MHz, DMSO): δ [ppm] = 172.2, 171.2, 170.8, 167.2, 147.6, 131.5, 127.8, 127.5, 42.2, 41.9, 40.1, 36.0, 35.2, 28.1, 25.1, 15.5, 13.1.

LC/MS: m/z calculated for C_19_H_26_N_4_O_4_ [M + H]^+^: 375.2, found: 375.1.

**Figure:**
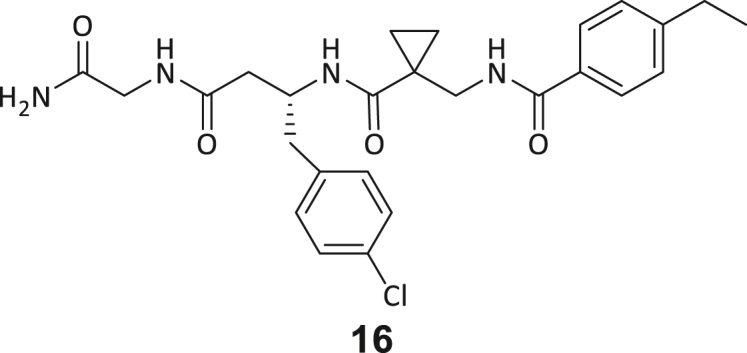

Compound **16**: Yield: 21.0 mg (0.042 mmol ≙ 26% of th.), colorless solid.

^1^H-NMR (300 MHz, DMSO-*d*_6_): δ [ppm] = 8.58 (t, *J* = 6.1 Hz, 1H), 8.11 (t, *J* = 5.8 Hz, 1H), 8.01 (d, *J* = 8.1 Hz, 1H), 7.77 (d, *J* = 8.2 Hz, 2H), 7.31 (d, *J* = 8.2 Hz, 2H), 7.25 (s, 1H), 7.13 (s, 4H), 7.03 (s, 1H), 4.28 (q, *J* = 7.8 Hz, 1H), 3.68–3.40 (m, 4H), 2.82 (dd, *J* = 13.6, 4.8 Hz, 1H), 2.74–2.60 (m, 3H), 2.32 (d, *J* = 6.9 Hz, 2H), 1.20 (t, *J* = 7.6 Hz, 3H), 0.93–0.72 (m, 4H).

^13^C-NMR (75 MHz, DMSO): δ [ppm] = 171.6, 171.1, 170.3, 167.2, 147.6, 137.8, 131.4, 131.0, 130.6, 127.8, 127.7, 127.5, 54.9, 48.2, 42.3, 41.9, 40.2, 38.6, 28.1, 25.3, 15.4, 13.2, 13.1.

LC/MS: m/z calculated for C_26_H_31_ClN_4_O_4_ [M + H]^+^: 499.2, found: 498.9.

**Figure:**
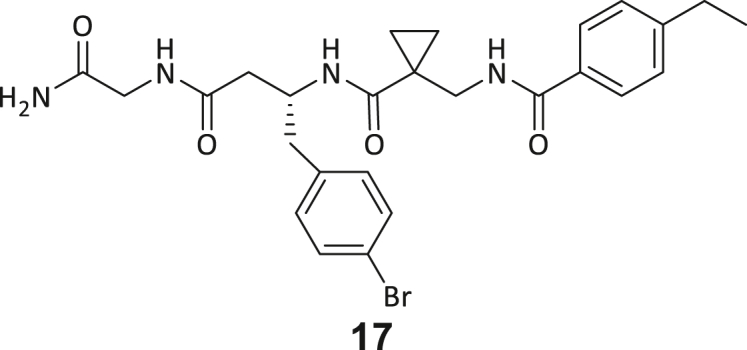

Compound **17**: Yield: 29.1 mg (0.053 mmol ≙ 34% of th.), colorless solid.

^1^H-NMR (300 MHz, DMSO-*d*_6_): δ [ppm] = 8.59 (t, *J* = 6.1 Hz, 1H), 8.12 (t, *J* = 5.9 Hz, 1H), 8.01 (d, *J* = 8.1 Hz, 1H), 7.77 (d, *J* = 8.3 Hz, 2H), 7.35–7.29 (m, 2H), 7.28–7.20 (m, 3H), 7.11–6.99 (m, 3H), 4.28 (td, *J* = 7.8, 4.8 Hz, 1H), 3.69–3.47 (m, 3H), 3.42 (d, *J* = 5.9 Hz, 1H), 2.80 (dd, *J* = 13.6, 4.8 Hz, 1H), 2.65 (p, *J* = 7.6 Hz, 3H), 2.32 (d, *J* = 6.9 Hz, 2H), 1.20 (t, *J* = 7.6 Hz, 3H), 0.95–0.71 (m, 4H).

^13^C-NMR (75 MHz, DMSO): δ [ppm] = 172.0, 171.5, 170.7, 167.6, 148.0, 138.7, 131.8, 131.2, 128.2, 127.9, 119.6, 48.6, 42.8, 42.4, 28.5, 25.7, 15.9, 13.7, 13.5.

LC/MS: m/z calculated for C_26_H_31_BrN_4_O_4_ [M + H]^+^: 543.1, found: 543.1.

**Figure:**
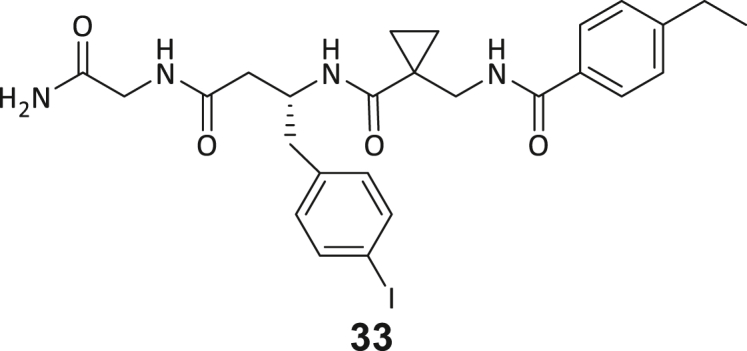

Compound **33**: Yield: 32.1 mg (0.054 mmol ≙ 34% of th.), colorless solid.

^1^H-NMR (300 MHz, DMSO-*d*_6_): δ [ppm] = 8.59 (t, *J* = 6.1 Hz, 1H), 8.11 (t, *J* = 5.9 Hz, 1H), 8.00 (d, *J* = 8.1 Hz, 1H), 7.77 (d, *J* = 8.3 Hz, 2H), 7.46–7.37 (m, 2H), 7.36–7.28 (m, 2H), 7.25 (s, 1H), 7.03 (s, 1H), 6.93 (d, *J* = 8.3 Hz, 2H), 4.27 (q, *J* = 7.2 Hz, 1H), 3.74–3.47 (m, 4H), 2.78 (dd, *J* = 13.7, 4.8 Hz, 1H), 2.72–2.56 (m, 3H), 2.31 (d, *J* = 6.9 Hz, 2H), 1.21 (t, *J* = 7.6 Hz, 3H), 0.95–0.71 (m, 4H).

^13^C-NMR (75 MHz, DMSO): δ [ppm] = 172.0, 171.5, 170.7, 167.6, 148.0, 139.0, 137.1, 132.0, 131.8, 128.2, 127.9, 92.2, 48.6, 42.8, 42.4, 40.7, 28.6, 25.7, 15.9, 13.7, 13.5.

LC/MS: m/z calculated for C_26_H_31_ClN_4_O_4_ [M + H]^+^: 591.1, found: 591.1.

**Figure:**
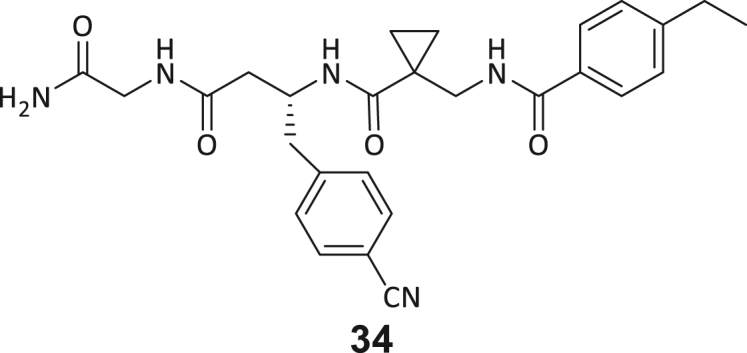

Compound **34**: Yield: 26.0 mg (0.044 mmol ≙ 28% of th.), colorless solid.

^1^H-NMR (300 MHz, DMSO-*d*_6_): δ [ppm] = 8.59 (t, *J* = 6.1 Hz, 1H), 8.15 (t, *J* = 5.9 Hz, 1H), 8.06 (d, *J* = 8.1 Hz, 1H), 7.74 (d, *J* = 8.3 Hz, 2H), 7.50 (d, *J* = 8.3 Hz, 2H), 7.30 (t, *J* = 7.9 Hz, 5H), 7.03 (s, 1H), 4.33 (dq, *J* = 11.7, 6.7 Hz, 1H), 3.68–3.49 (m, 3H), 3.33 (d, *J* = 5.9 Hz, 1H), 2.95 (dd, *J* = 13.6, 4.6 Hz, 1H), 2.81–2.60 (m, 3H), 2.35 (d, *J* = 7.0 Hz, 2H), 1.21 (t, *J* = 7.6 Hz, 3H), 0.94–0.70 (m, 4H).

^13^C-NMR (75 MHz, DMSO): δ [ppm] = 172.1, 171.5, 170.6, 167.6, 148.1, 145.3, 132.2, 131.7, 130.6, 128.1, 127.9, 119.4, 109.2, 48.3, 42.8, 42.3, 40.7, 28.5, 25.7, 15.8, 13.7, 13.5.

LC/MS: m/z calculated for C_27_H_31_N_5_O_4_ [M + H]^+^: 490.2, found: 490.2.

**Figure:**
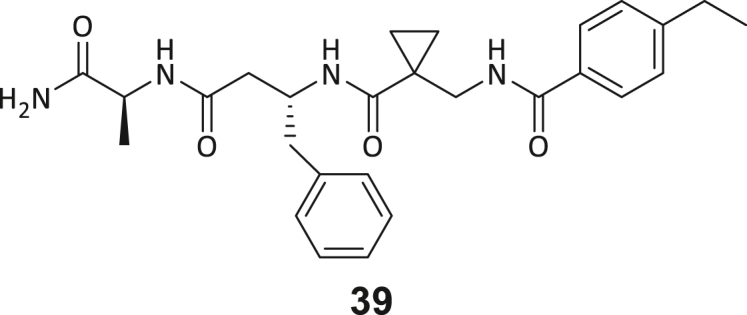

Compound **39**: Yield: 14 mg (0.029 mmol ≙ 64% of th.), colorless solid.

^1^H-NMR (300 MHz, DMSO-*d*_6_): δ [ppm] = 8.53 (t, *J* = 6.1 Hz, 1H), 8.03–7.90 (m, 2H), 7.85–7.69 (m, 2H), 7.39–7.28 (m, 2H), 7.26 (s, 1H), 7.20–7.01 (m, 5H), 6.96 (s, 1H), 4.29 (h, *J* = 7.4 Hz, 1H), 4.16 (p, *J* = 7.1 Hz, 1H), 3.48 (p, *J* = 8.5 Hz, 2H), 2.78 (dd, *J* = 13.5, 5.2 Hz, 1H), 2.72–2.58 (m, 3H), 2.28 (d, *J* = 6.9 Hz, 2H), 1.24–1.11 (m, 6H), 0.92–0.71 (m, 4H).

^13^C-NMR (75 MHz, DMSO): δ [ppm] = 174.4, 171.5, 169.7, 167.2, 147.6, 138.8, 131.5, 129.1, 128.0, 127.7, 127.5, 126.0, 48.5, 48.0, 42.2, 28.1, 25.2, 18.2, 15.4, 13.0, 12.9.

LC/MS: m/z calculated for C_27_H_34_N_4_O_4_ [M + H]^+^: 479.3, found: 479.2.

**Figure:**
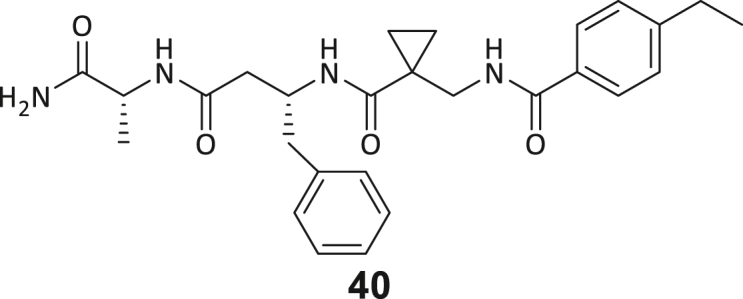

Compound **40**: Yield: 3.0 mg (6.263 μmol ≙ 14% of th.), colorless solid.

^1^H-NMR (400 MHz, DMSO-*d*_6_): δ [ppm] = 8.54 (t, *J* = 6.0 Hz, 1H), 8.01–7.90 (m, 2H), 7.77 (d, *J* = 7.8 Hz, 2H), 7.31 (d, *J* = 7.8 Hz, 2H), 7.25 (s, 1H), 7.18–7.02 (m, 5H), 6.95 (s, 1H), 4.28 (q, *J* = 7.0 Hz, 1H), 4.16 (p, *J* = 7.2 Hz, 1H), 3.62–3.51 (m, 1H), 3.44–3.34 (m, 1H), 2.83–2.60 (m, 4H), 2.30 (d, *J* = 6.9 Hz, 2H), 1.28–1.09 (m, 6H), 0.95–0.67 (m, 4H).

^13^C-NMR (101 MHz, DMSO): δ [ppm] = 174.3, 171.4, 169.7, 167.2, 147.6, 138.8, 131.4, 129.1, 127.9, 127.7, 127.5, 125.9, 48.4, 48.0, 42.3, 40.2, 28.1, 25.2, 18.2, 15.4, 13.0, 12.9.

LC/MS: m/z calculated for C_27_H_34_N_4_O_4_ [M + H]^+^: 479.3, found: 479.1.

**Figure:**
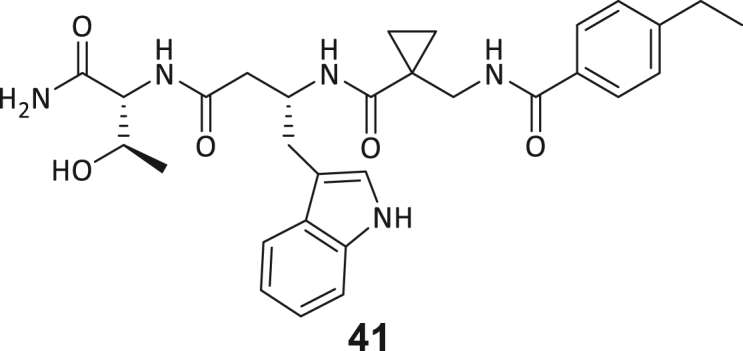

Compound **41**: Yield: 7.0 mg (0.013 mmol ≙ 28% of th.), colorless solid.

^1^H-NMR (300 MHz, DMSO-*d*_6_): δ [ppm] = 10.75 (d, *J* = 2.4 Hz, 1H), 8.56 (t, *J* = 6.1 Hz, 1H), 8.00 (d, *J* = 7.9 Hz, 1H), 7.86 (d, *J* = 8.6 Hz, 1H), 7.82–7.68 (m, 3H), 7.58 (d, *J* = 7.8 Hz, 1H), 7.37–7.19 (m, 5H), 7.12–6.98 (m, 3H), 6.98–6.88 (m, 1H), 4.35 (q, *J* = 6.9 Hz, 1H), 4.21 (dd, *J* = 8.6, 6.1 Hz, 1H), 3.84 (p, *J* = 6.3 Hz, 1H), 2.82 (d, *J* = 6.5 Hz, 2H), 2.65 (q, *J* = 7.6 Hz, 2H), 2.38 (d, *J* = 6.9 Hz, 2H), 1.19 (t, *J* = 7.6 Hz, 4H), 1.03 (d, *J* = 6.3 Hz, 3H), 0.93–0.86 (m, 2H), 0.81–0.73 (m, 2H).

^13^C-NMR (75 MHz, DMSO): δ [ppm] = 172.3, 171.6, 170.5, 167.2, 147.5, 136.1, 131.5, 127.7, 127.7, 127.6, 127.4, 127.4, 123.2, 120.8, 118.6, 118.2, 111.3, 111.2, 66.8, 58.1, 47.9, 42.2, 40.2, 29.7, 28.1, 25.3, 19.5, 15.4, 13.3, 13.0.

LC/MS: m/z calculated for C_30_H_37_N_5_O_5_ [M + H]^+^: 548.3, found: 547.8.

**Figure:**
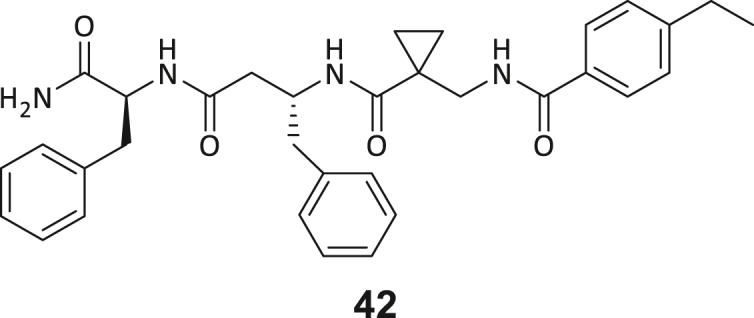

Compound **42**: Yield: 15.0 mg (0.027 mmol ≙ 59% of th.), colorless solid.

^1^H-NMR (300 MHz, DMSO-*d*_6_): δ [ppm] = 8.54 (t, *J* = 6.1 Hz, 1H), 8.14 (d, *J* = 8.4 Hz, 1H), 7.98 (d, *J* = 8.2 Hz, 1H), 7.87–7.71 (m, 2H), 7.39 (s, 1H), 7.31 (d, *J* = 8.2 Hz, 2H), 7.27–7.22 (m, 3H), 7.22–6.95 (m, 8H), 4.55–4.35 (m, 1H), 4.24 (q, *J* = 7.3 Hz, 1H), 3.59–3.42 (m, 2H), 3.02 (dd, *J* = 13.8, 4.7 Hz, 1H), 2.84–2.42 (m, 5H), 2.23 (qd, *J* = 14.2, 6.9 Hz, 2H), 1.19 (t, *J* = 7.6 Hz, 3H), 0.97–0.51 (m, 4H).

^13^C-NMR (75 MHz, DMSO): δ [ppm] = 173.3, 171.5, 170.0, 167.2, 147.6, 138.8, 138.3, 131.5, 129.1, 128.1, 127.9, 127.7, 127.5, 126.2, 125.9, 53.9, 48.4, 42.3, 37.5, 28.1, 25.2, 15.4, 13.0, 13.0.

LC/MS: m/z calculated for C_33_H_38_N_4_O_4_ [M + H]^+^: 555.3, found: 555.2.

**Figure:**
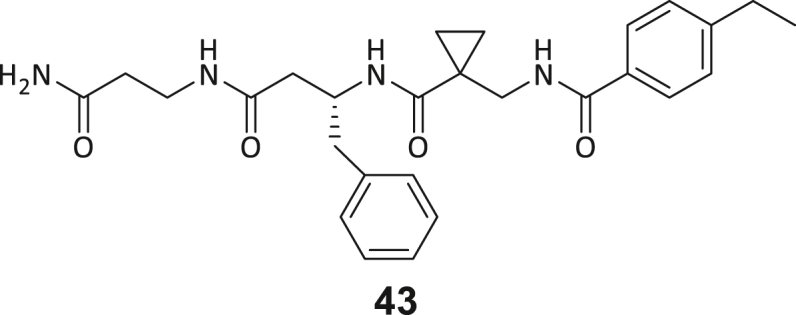

Compound **43**: Yield: 18.0 mg (0.038 mmol ≙ 82% of th.), colorless solid.

^1^H-NMR (300 MHz, DMSO-*d*_6_): δ [ppm] = 8.57 (t, *J* = 6.1 Hz, 1H), 8.05–7.84 (m, 2H), 7.85–7.70 (m, 2H), 7.40–7.24 (m, 3H), 7.22–7.01 (m, 5H), 6.82 (s, 1H), 4.26 (h, *J* = 7.1 Hz, 1H), 3.61–3.39 (m, 2H), 3.29–3.06 (m, 2H), 2.81–2.57 (m, 4H), 2.32–2.04 (m, 4H), 1.19 (t, *J* = 7.6 Hz, 3H), 0.98–0.69 (m, 4H).

^13^C-NMR (75 MHz, DMSO): δ [ppm] = 172.6, 171.4, 169.9, 167.2, 147.6, 138.8, 131.4, 129.2, 128.0, 127.7, 127.5, 125.9, 48.4, 42.2, 40.2, 35.2, 35.0, 28.1, 25.2, 15.4, 13.0.

LC/MS: m/z calculated for C_27_H_34_N_4_O_4_ [M + H]^+^: 479.3, found: 479.2.

**Figure:**
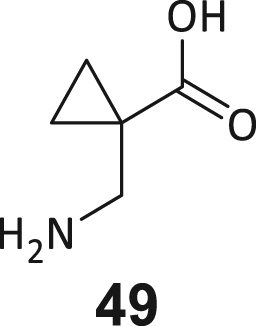

Compound **49**: 1-Cyanocyclopropane-1-carboxylic acid (500 mg, 4.500 mmol, 1.0 eq.) was dissolved in methanol (10 mL), ammonia in methanol (7 m, 1.0 mL), and Raney-nickel (50 mg, 5%) were added. The mixture was stirred under a hydrogen atmosphere (4 bar) for 16 h at RT. The reaction mixture was adjusted to alkaline pH (pH = 8) by adding ammonia in methanol (7 m, 0.5 mL), filtered over Celite S, and the solvent was removed under reduced pressure.

Yield: 574.0 mg (4.986 mmol ≙ quant.), colorless solid.

^1^H-NMR (300 MHz, MeO*D*): δ [ppm] = 3.06 (s, 2H), 1.36–1.29 (m, 2H), 1.02–0.94 (m, 2H).

^13^C-NMR (75 MHz, MeOD): δ [ppm] = 177.8, 101.2, 45.2, 15.0.

LC/MS: m/z calculated for C_5_H_9_NO_2_ [M + H]^+^: 116.1, found: 116.0.

**Figure:**
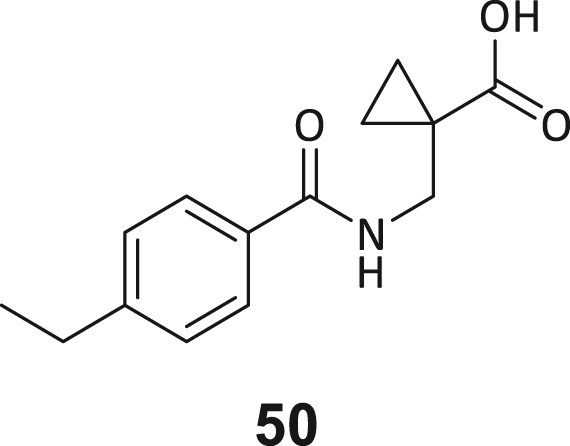

Compound **50**: The crude product **49** (518 mg, 4.500 mmol, 1.0 eq.) was suspended in DCM (10 mL), DMF (0.5 mL), and TEA (620 μL, 4.473 mmol, 1.0 eq.). The mixture was cooled to 0 °C, and a solution of acid 4-ethylbenzoyl chloride (662 μL, 4.500 mmol, 1.0 eq.) in DCM (0.3 mL) was added. The mixture was warmed to RT and stirred for 16 h. The reaction mixture was washed with 1 m hydrochloric acid thrice (5 mL each). The organic phase was separated, dried over sodium sulfate, and the solvents were removed under reduced pressure. The crude product was purified by column chromatography (mobile phase: CH:EtOAc 2:1 with 0.1% TFA)

Yield: 413.0 mg (1.670 mmol ≙ 37% of th.), colorless solid.

^1^H-NMR (300 MHz, DMSO-*d*_6_): δ [ppm] = 9.99 (s, 1H), 7.80–7.62 (m, 2H), 7.29–7.20 (m, 2H), 7.00 (t, *J* = 6.2 Hz, 1H), 3.62 (d, *J* = 6.1 Hz, 2H), 2.68 (q, *J* = 7.6 Hz, 2H), 1.35 (q, *J* = 4.2 Hz, 2H), 1.24 (t, *J* = 7.6 Hz, 3H), 1.15 (q, *J* = 4.3 Hz, 2H).

^13^C-NMR (75 MHz, DMSO): δ [ppm] = 181.1, 167.9, 148.5, 131.6, 128.2, 127.3, 42.9, 28.9, 24.8, 16.0, 15.5.

LC/MS: m/z calculated for C_14_H_17_NO_3_ [M + H]^+^: 248.1, found: 248.0.

**Figure:**
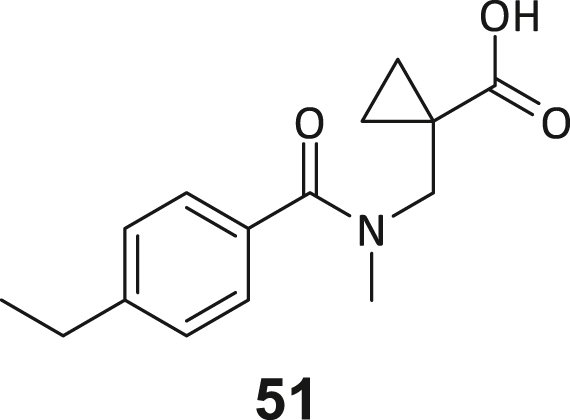

Compound **51**: Compound **50** (50 mg, 0.202 mmol, 1.0 eq.) was dissolved in anhydrous THF (5 mL) and cooled to 0 °C. Sodium hydride (60% suspension, 21 mg, 0.525 mmol, 2.6 eq.) was added and the mixture was stirred for 5 min. Then, methyl iodide (38 μL, 0.610 mmol, 3.0 eq.) was added dropwise. The mixture was warmed to RT and stirred for 16 h. A solution of sodium hydroxide (18 mg, 0.450 mmol, 2.2 eq.) in water (5 mL) was added and the mixture was stirred for 1 h. The mixture was adjusted to acidic pH (pH = 1) with 1 m hydrochloric acid and the solvents were removed under reduced pressure. The crude product was dissolved in sodium hydroxide solution (pH = 12) and was extracted three times with DCM (3 mL each). Then, the aqueous phase was brought to an acidic pH (pH = 1) with 1 m hydrochloric acid and extracted thrice with DCM (3 mL each). The organic phases from the acidic extraction were combined, and dried over sodium sulfate, and the solvent was removed under reduced pressure.

Yield: 44.0 mg (0.168 mmol ≙ 83% of th.), colorless solid.

^1^H-NMR (300 MHz, C*D*Cl_3_, 60 °C): δ [ppm] = 9.87 (s, 1H), 7.30 (d, *J* = 8.2 Hz, 2H), 7.19 (d, *J* = 7.9 Hz, 2H), 3.82 (s, 2H), 3.05 (s, 3H), 2.66 (q, *J* = 7.6 Hz, 2H), 1.39 (q, *J* = 4.1 Hz, 2H), 1.24 (t, *J* = 7.6 Hz, 3H), 1.15–0.94 (m, 2H).

^13^C-NMR (75 MHz, *C*DCl_3_, 60 °C): δ [ppm] = 179.1, 173.0, 146.2, 133.7, 128.1, 127.9, 127.4, 127.3, 28.8, 23.2, 16.2, 15.3.

LC/MS: m/z calculated for C_15_H_19_NO_3_[M + H]^+^: 262.2, found: 262.0.

**Figure:**
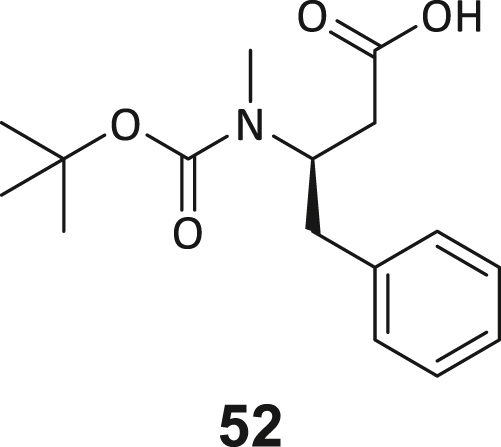

Compound **52**: (*R*)-3-*tert*-butoxycarbonylamino-4-phenylbutyric acid (100 mg, 0.358 mmol, 1.0 eq.) was dissolved in anhydrous THF (7 mL) and cooled to 0 °C. Sodium hydride (60% suspension, 39 mg, 0.975 mmol, 2.7 eq.) was added and the mixture was stirred for 5 min. Then, methyl iodide (67 μL, 1.074 mmol, 3.0 eq.) was added dropwise. The mixture was warmed to RT and stirred for 16 h. Because the reaction was not complete, methyl iodide (46 μL, 0.739 mmol, 2.1 eq.) and sodium hydride (34 mg, 0.850 mmol, 2.4 eq.) were added and the mixture was stirred for an additional 16 h. Water (2 mL) was added, and the pH was confirmed to be > 12. The mixture was stirred for 1 h and all solvents were removed under reduced pressure and the crude product was purified by reversed-phase flash column chromatography (Biotage Isolera, ACN/H_2_O + TFA).

Yield: 39.0 mg (0.133 mmol ≙ 37% of th.), colorless solid.

^1^H-NMR (300 MHz, C*D*Cl_3_, 60 °C): δ [ppm] = 8.92 (s, 1H), 7.28–6.94 (m, 5H), 4.45 (tt, *J* = 8.9, 6.1 Hz, 1H), 2.93–2.52 (m, 5H), 2.45 (dd, *J* = 15.5 Hz, 5.8 Hz, 1H), 1.27 (s, 9H).

^13^C-NMR (75 MHz, *C*DCl_3_, 60 °C): δ [ppm] = 176.1, 155.9, 138.2, 129.2, 128.7, 126.8, 80.3, 55.7, 38.9, 37.6, 30.8, 28.5.

LC/MS: m/z calculated for C_16_H_23_NO_4_ [M-Boc+H]^+^: 194.1, found: 194.0.

**Figure:**
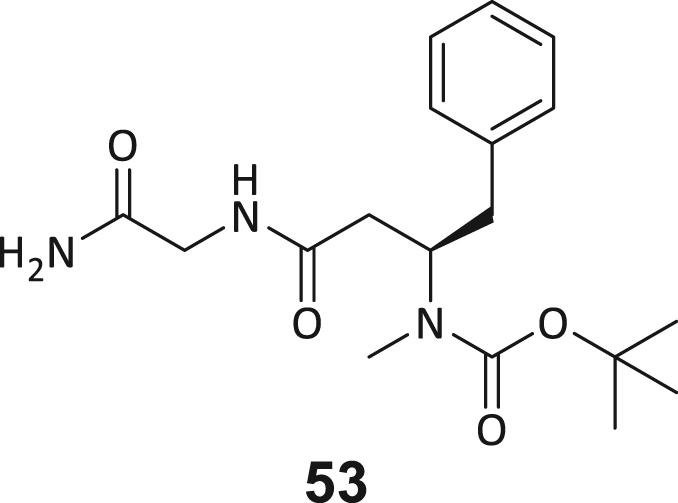

Compound **53**: Compound **52** (35 mg, 0.119 mmol, 1.0 eq.), glycine amide hydrochloride (14 mg, 0.127 mmol, 1.1 eq.), and TBTU (38 mg, 0.119 mmol, 1.0 eq.) were dissolved in anhydrous DMF (1.5 mL), and DIPEA (63 μL, 0.357 mmol, 3.0 eq.) was added. The mixture was stirred for 16 h at RT. All solvents were removed under reduced pressure, and the crude product was purified by reversed-phase flash column chromatography (Biotage Isolera, ACN/H_2_O + TFA).

Yield: 33.0 mg (0.094 mmol ≙ 79% of th.), colorless solid.

^1^H-NMR (300 MHz, C*D*Cl_3_): δ [ppm] = 7.37–6.99 (m, 6H), 6.85 (s, 1H), 5.92 (s, 1H), 4.62 (s, 1H), 4.17–3.65 (m, 2H), 3.11 (s, 1H), 2.97–2.54 (m, 5H), 2.55–2.34 (m, 1H), 1.33 (s, 9H).

^13^C-NMR (75 MHz, *C*DCl_3_): δ [ppm] = 138.0, 129.1, 128.6, 126.7, 80.2, 55.6, 43.0, 38.9, 30.6, 28.4.

LC/MS: m/z calculated for C_18_H_27_N_3_O_4_ [M-Boc+H]^+^: 250.2, found: 250.1.

**Figure:**
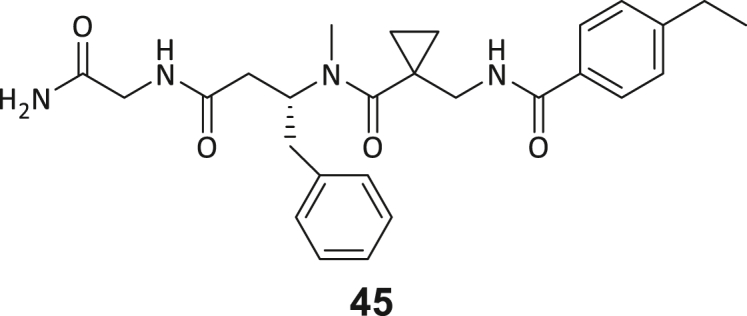

Compound **45**: Compound **53** (26 mg, 0.074 mmol, 1.0 eq.) was dissolved in DCM (1 mL) and TFA (1 mL) was added. The mixture was stirred at RT for 1.5 h. The solvents were removed under reduced pressure. The deprotected crude product (14 mg, 0.127 mmol, 1.1 eq.), **51** (18 mg, 0.074 mmol, 1.0 eq.), and TBTU (25 mg, 0.078 mmol, 1.1 eq.) were dissolved in anhydrous DMF (1.5 mL), and DIPEA (39 μL, 0.224 mmol, 3.0 eq.) was added. The mixture was stirred for 16 h at RT. The solvents were removed under reduced pressure and the crude product was purified by reversed-phase flash column chromatography (Biotage Isolera, ACN/H_2_O + TFA).

Yield: 32.0 mg (0.669 mmol ≙ 90% of th.), colorless solid.

^1^H-NMR (300 MHz, DMSO-*d*_6_): δ [ppm] = 8.34–8.08 (m, 2H), 7.71 (d, *J* = 7.9 Hz, 2H), 7.31–7.08 (m, 8H), 7.03 (s, 1H), 4.82 (s, 1H), 3.60 (s, 2H), 3.35 (s, 2H), 3.08–2.70 (m, 5H), 2.63 (q, *J* = 7.6 Hz, 2H), 2.57–2.33 (m, 2H), 1.18 (t, *J* = 7.6 Hz, 3H), 0.70–0.28 (m, 4H).

^13^C-NMR (75 MHz, DMSO): δ [ppm] = 171.2, 171.0, 170.5, 166.4, 147.3, 138.6, 131.8, 128.9, 128.1, 127.6, 127.3, 126.1, 41.9, 41.5, 37.4, 37.1, 30.7, 28.0, 26.0, 15.4, 9.5.

LC/MS: m/z calculated for C_27_H_34_N_4_O_4_ [M + H]^+^: 479.3, found: 479.1.

**Figure:**
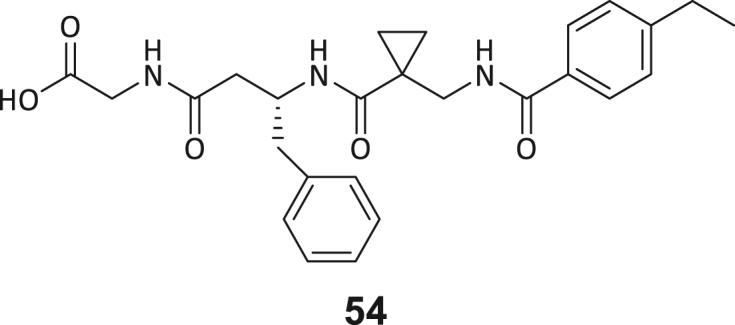

Compound **54**: Yield: 48.0 mg (0.103 mmol ≙ 76% of th.): colorless solid.

^1^H-NMR (300 MHz, DMSO-*d*_6_): δ [ppm] = 12.51 (s, 1H), 8.57 (t, *J* = 6.0 Hz, 1H), 8.24 (t, *J* = 5.9 Hz, 1H), 7.99 (d, *J* = 8.0 Hz, 1H), 7.78 (d, *J* = 7.9 Hz, 2H), 7.32 (d, *J* = 7.9 Hz, 2H), 7.23–6.96 (m, 5H), 4.41–4.10 (m, 1H), 3.88–3.56 (m, 2H), 3.58–3.16 (m, 2H), 2.82 (dd, *J* = 13.5, 5.0 Hz, 1H), 2.66 (q, *J* = 7.6 Hz, 3H), 2.40–2.14 (m, 2H), 1.20 (t, *J* = 7.5 Hz, 3H), 0.97–0.59 (m, 4H).

^13^C-NMR (75 MHz, DMSO): δ [ppm] = 171.3, 170.4, 167.2, 147.6, 131.4, 138.9, 129.1, 128.0, 125.9, 127.7, 127.5, 48.5, 42.3, 39.8, 39.1, 28.1, 25.2, 15.4, 13.1.

LC/MS: m/z calculated for C_26_H_31_N_3_O_5_ [M + H]^+^: 466.2, found: 466.1.

**Figure:**
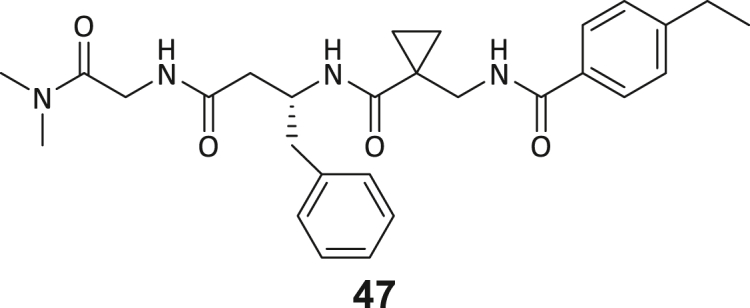

Compound **47**: Compound **54** (10 mg, 0.022 mmol, 1.0 eq.), dimethylamine hydrochloride (2.9 mg, 0.036 mmol, 1.7 eq.), DCC (5.6 mg, 0.027 mmol, 1.3 eq.) and HOBt (3.6 mg, 0.027 mmol, 1.3 eq.) were dissolved in DCM (1 mL). DIPEA (16 μL, 0.092 μmol, 4.3 eq.) was added dropwise and the mixture was stirred for 16 h at RT. More solvent (DCM, 1 mL), dimethylamine hydrochloride (2.6 mg, 0.032 mmol, 1.5 eq.), DCC (5 mg, 0.024 mmol, 1.1 eq.), and DIPEA (11.7 μL, 0.067 mmol, 2.5 eq.) were added and the mixture was stirred for additional 16 h at RT because the reaction was not completed. The solvents were removed under reduced pressure and the crude product was purified by reversed-phase column chromatography (Biotage Isolera, ACN/H_2_O + TFA).

Yield: 8.0 mg (0.016 mmol ≙ 76% of th.), colorless solid.

^1^H-NMR (300 MHz, DMSO-*d*_6_): δ [ppm] = 8.54 (t, *J* = 6.0 Hz, 1H), 8.00–7.89 (m, 2H), 7.81–7.73 (m, 2H), 7.35–7.25 (m, 2H), 7.17–7.06 (m, 5H), 4.25 (p, *J* = 7.1 Hz, 1H), 3.96–3.66 (m, 2H), 3.47 (qd, *J* = 14.8, 6.1 Hz, 2H), 2.89 (s, 3H), 2.80 (s, 3H), 2.74–2.59 (m, 2H), 2.41–2.22 (m, 2H), 1.19 (t, *J* = 7.6 Hz, 3H), 0.95–0.67 (m, 4H).

^13^C-NMR (75 MHz, DMSO): δ [ppm] = 171.4, 170.1, 168.1, 167.1, 147.5, 138.9, 131.4, 129.2, 128.0, 127.6, 127.5, 125.9, 48.5, 42.3, 40.5, 39.1, 35.6, 35.1, 28.1, 25.2, 15.4, 13.1, 13.0.

LC/MS: m/z calculated for C_28_H_36_N_4_O_4_ [M + H]^+^: 493.3, found: 493.2.

**Figure:**
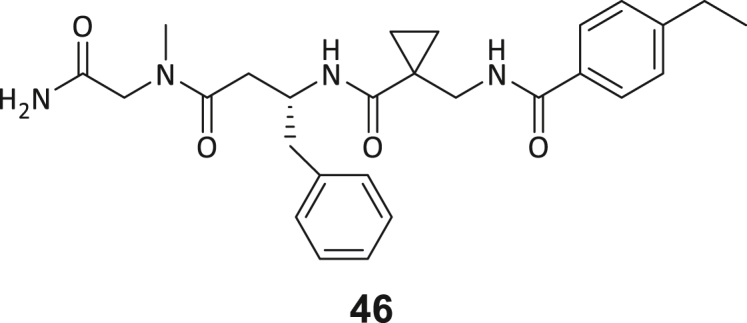

Compound **46**: Yield: 16.0 mg (0.033 mmol ≙ 74% of th.), colorless solid.

^1^H-NMR (300 MHz, DMSO-*d*_6_, 60 °C): δ [ppm] = 8.41–8.23 (m, 1H), 7.93–7.79 (m, 1H), 7.79–7.68 (m, 2H), 7.36–7.25 (m, 2H), 7.24–7.00 (m, 6H), 4.33 (q, *J* = 7.3 Hz, 1H), 4.04–3.69 (m, 2H), 3.62–3.34 (m, 2H), 2.99–2.72 (m, 4H), 2.72–2.60 (m, 2H), 2.60–2.27 (m, 2H), 1.21 (t, *J* = 7.6 Hz, 3H), 1.02–0.63 (m, 4H).

^13^C-NMR (75 MHz, DMSO, 60 °C): δ [ppm] = 171.3, 170.3, 170.0, 166.9, 147.2, 138.8, 131.4, 128.8, 127.7, 127.3, 127.1, 125.6, 50.0, 48.2, 48.0, 42.2, 37.2, 35.9, 27.7, 25.0, 14.9, 12.6.

LC/MS: m/z calculated for C_27_H_34_N_4_O_4_ [M + H]^+^: 479.3, found: 479.2.

**Figure:**
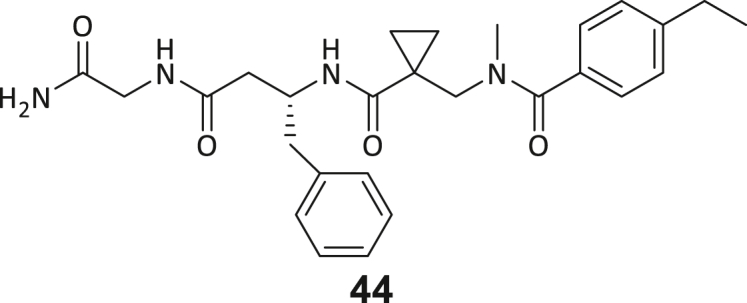

Compound **44**: Yield: 17.0 mg (0.036 mmol ≙ 79% of th.), colorless solid.

^1^H-NMR (300 MHz, DMSO-*d*_6_, 60 °C): δ [ppm] = 7.85 (t, *J* = 5.7 Hz, 1H), 7.53 (s, 1H), 7.28–7.10 (m, 10H), 4.34 (h, *J* = 7.1 Hz, 1H), 3.80–3.45 (m, 4H), 2.88–2.70 (m, 5H), 2.65 (q, *J* = 7.6 Hz, 2H), 2.38–2.28 (m, 2H), 1.21 (t, *J* = 7.6 Hz, 3H), 1.03–0 0.81 (m, 2H), 0.80–0.58 (m, 2H).

^13^C-NMR (75 MHz, DMSO, 60 °C): δ [ppm] = 170.9, 170.9, 170.7, 170.0, 144.9, 138.5, 133.5, 128.8, 127.7, 127.2, 126.7, 125.7, 47.9, 41.8, 40.0, 39.4, 27.7, 23.7, 14.8, 12.0.

LC/MS: m/z calculated for C_27_H_34_N_4_O_4_ [M + H]^+^: 479.3, found: 479.2.

**Figure:**
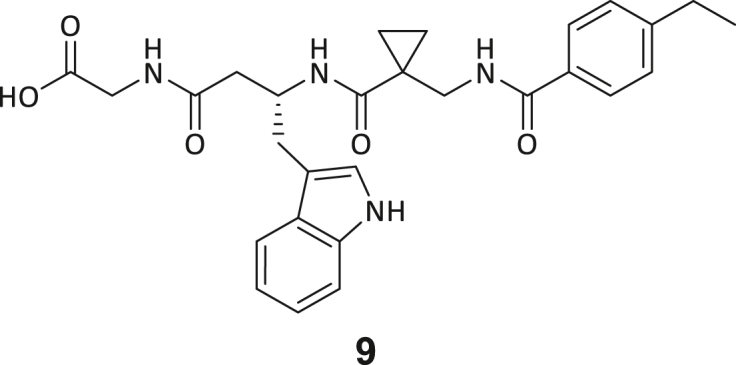

Compound **9**: Yield: 1.4 mg (0.028 mmol ≙ 43% of th.), colorless solid.

^1^H-NMR (400 MHz, MeO*D*): δ [ppm] = 8.24 (d, *J* = 7.6 Hz, 1H), 7.66 (d, *J* = 7.8 Hz, 2H), 7.60 (d, *J* = 7.7 Hz, 1H), 7.25 (d, *J* = 6.5 Hz, 3H), 7.09–6.94 (m, 4H), 6.65 (d, *J* = 7.7 Hz, 1H), 4.56 (d, *J* = 6.4 Hz, 1H), 3.74 (q, *J* = 17.8 Hz, 2H), 3.55 (s, 2H), 3.01 (p, *J* = 7.9 Hz, 2H), 2.69 (q, *J* = 7.5 Hz, 2H), 2.51 (d, *J* = 6.5 Hz, 2H), 2.22 (s, 1H), 1.45 (s, 1H), 1.25 (t, *J* = 7.5 Hz, 3H), 1.10 (q, *J* = 9.8 Hz, 2H), 0.86 (s, 2H).

^13^C-NMR (101 MHz, MeOD): δ [ppm] = 174.9, 173.8, 170.8, 156.0, 149.9, 138.0, 132.5, 130.8, 129.6, 129.2, 129.0, 128.6, 124.3, 122.2, 119.7, 119.6, 116.0, 112.2, 44.6, 40.9, 30.8, 29.7, 28.0, 26.8, 20.5, 15.8, 14.6, 14.4.

LC/MS: m/z calculated for C_28_H_32_N_4_O_5_ [M + H]^+^: 505.2, found: 504.7.

**Figure:**
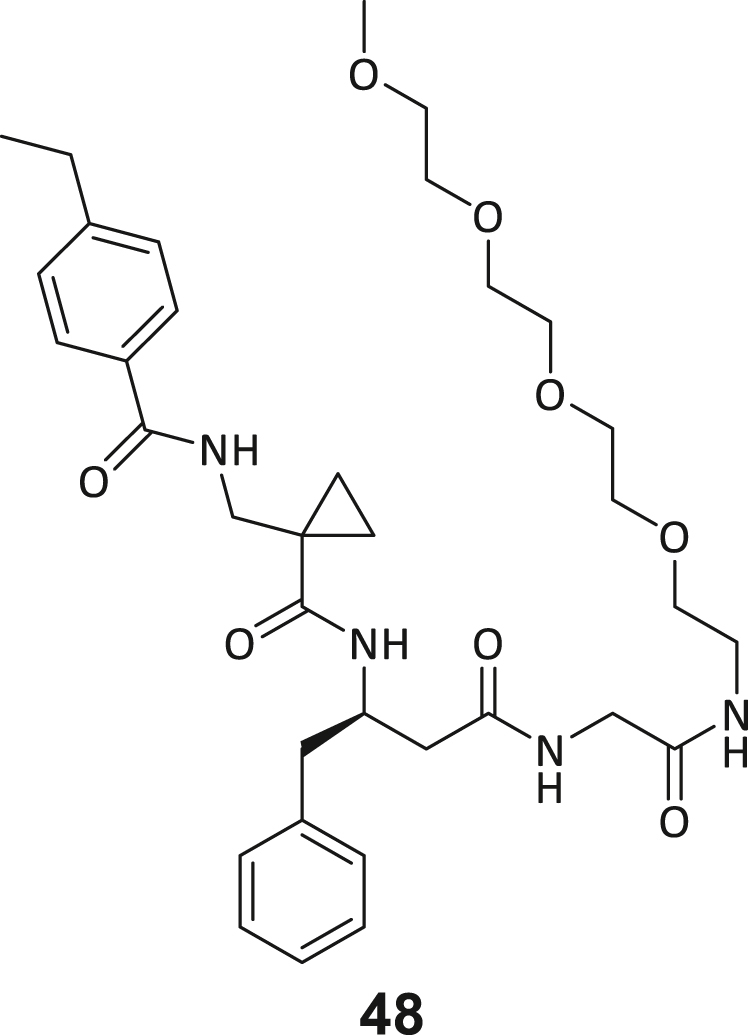

Compound **48**: Yield: 5.0 mg (7.641 μmol ≙ 44% of th.), colorless solid.

^1^H-NMR (400 MHz, DMSO-*d*_*6*_): δ [ppm] = 8.55 (t, *J* = 6.1 Hz, 1H), 8.13 (t, *J* = 5.9 Hz, 1H), 7.99 (d, *J* = 8.1 Hz, 1H), 7.83 (t, *J* = 5.7 Hz, 1H), 7.80–7.73 (m, 2H), 7.31 (d, *J* = 8.1 Hz, 2H), 7.21–7.02 (m, 5H), 4.29 (q, *J* = 7.1 Hz, 1H), 3.79–3.44 (m, 14H), 3.44–3.27 (m, 4H), 3.26–3.12 (m, 5H), 2.79 (dd, *J* = 13.5, 5.2 Hz, 1H), 2.72–2.58 (m, 3H), 2.31 (d, *J* = 6.8 Hz, 2H), 1.20 (t, *J* = 7.6 Hz, 3H), 0.96–0.66 (m, 4H).

^13^C-NMR (101 MHz, DMSO): δ [ppm] = 171.5, 170.4, 169.0, 167.2, 147.6, 138.8, 131.4, 129.1, 128.0, 127.7, 127.5, 126.0, 71.3, 69.8, 69.7, 69.6, 69.0, 58.1, 48.4, 42.3, 42.0, 38.6, 30.7, 28.1, 25.2, 15.4, 13.0.

LC/MS: m/z calculated for C_35_H_50_N_4_O_8_ [M + H]^+^: 655.4, found: 655.3.

**Figure:**
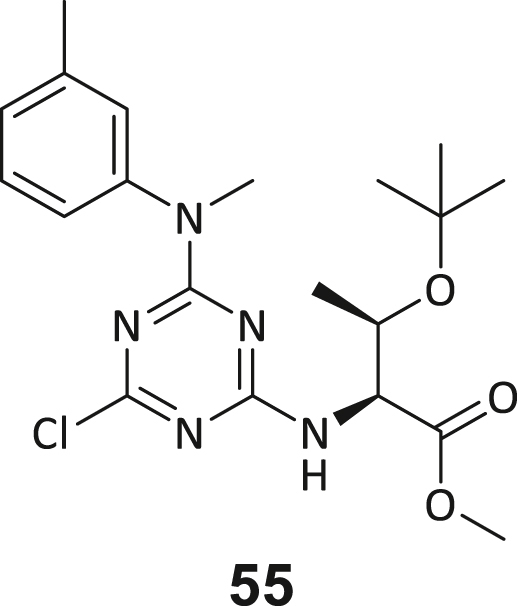

Compound **55**: Cyanuric chloride (195 mg, 1.057 mmol, 1.0 eq.) and methyl *O*-(*tert*-butyl)-l-threoninate (200 mg, 1.057 mmol, 1.0 eq.) were suspended in 2 mL of DCM. The solution was cooled to 0 °C and DIPEA (370 μL, 2.114 mmol, 2.0 eq.) was added. The reaction was allowed to stir for 10 min at 0 °C. Subsequently, *N*,3-dimethylaniline (128 mg, 1.057 mmol, 1.0 eq.), and DIPEA (370 μL, 2.114 mmol, 2.0 eq.) were added at 0 °C and the mixture was stirred for 12 h at 40 °C in a sealed tube. The crude product was purified by column chromatography (mobile phase: CH:EtOAc 20:1–10:1).

Yield: 331.0 mg (0.786 mmol ≙ 74% of th.), colorless oil.

^1^H-NMR (300 MHz, C*D*Cl_3_): δ [ppm] = 7.26 (s, 1H), 7.14–6.91 (m, 3H), 6.20–5.73 (m, 1H), 4.41–3.97 (m, 1H), 3.68 (d, *J* = 13.7 Hz, 3H), 3.44 (d, *J* = 7.4 Hz, 3H), 2.36 (d, *J* = 8.2 Hz, 3H), 1.38–0.97 (m, 12H).

^13^C-NMR (75 MHz, *C*DCl_3_): δ [ppm] = 171.6, 165.3, 143.5, 138.8, 129.0, 127.4, 127.0, 124.0, 123.6, 77.4, 74.2, 67.3, 59.6, 52.2, 38.8, 28.4, 21.5.

LC/MS: m/z calculated for C_20_H_28_ClN_5_O_3_ [M + H]^+^: 422.2, found: 422.1.

**Figure:**
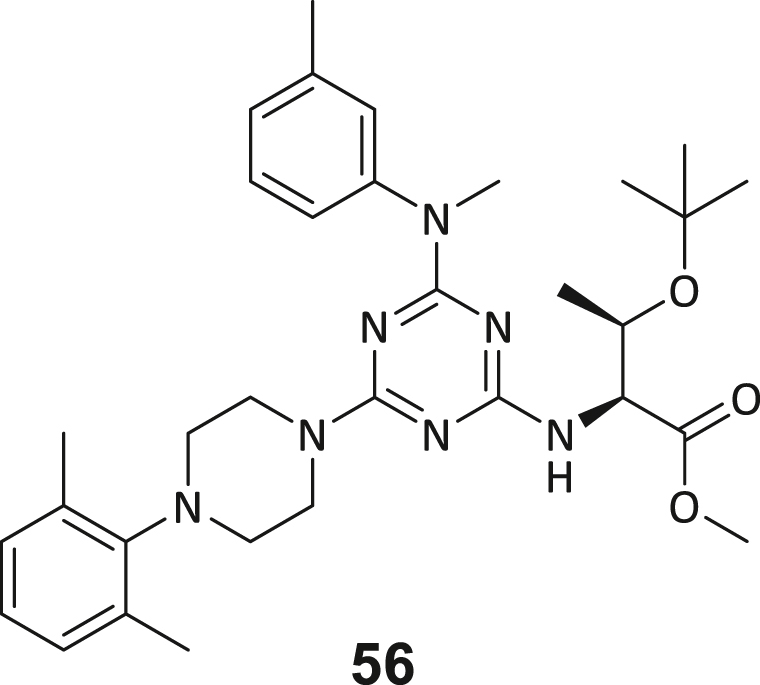

Compound **56**: Compound **55** (80 mg, 0.190 mmol, 1.0 eq.) was dissolved in DMF, 1-(2,6-dimethylphenyl)piperazine (43 mg, 0.228 mmol, 1.2 eq.) and potassium carbonate (65 mg, 0.475 mmol, 2.5 eq.) were added, and the mixture was allowed to stir for 45 min at 100 °C. The solvent was removed in vacuo and the residue was dissolved in DCM, which was washed three times with water. The organic phase was filtered over silica and the product was obtained as an orange oil.

Yield: 103.0 mg (0.179 mmol ≙ 94% of th.), orange oil.

^1^H-NMR (300 MHz, C*D*Cl_3_): δ [ppm] = 7.28–7.09 (m, 3H), 6.99 (h, *J* = 4.6, 4.1 Hz, 4H), 4.27–4.11 (m, 1H), 3.81 (d, *J* = 22.4 Hz, 3H), 3.69 (s, 3H), 3.47 (d, *J* = 11.3 Hz, 3H), 3.15–3.00 (m, 4H), 2.95 (s, 1H), 2.88 (s, 1H), 2.34 (dd, *J* = 8.9, 2.4 Hz, 9H), 1.24 (dd, *J* = 9.4, 6.3 Hz, 3H), 1.13 (d, *J* = 4.8 Hz, 9H).

^13^C-NMR (75 MHz, *C*DCl_3_): δ [ppm] = 172.8, 162.6, 161.1, 148.3, 137.9, 136.9, 129.0, 128.0, 127.1, 125.7, 125.2, 123.5, 77.4, 73.9, 67.6, 59.6, 59.4, 51.9, 50.7, 49.8, 47.3, 44.6, 41.6, 36.5, 31.5, 28.4, 21.2, 19.8.

LC/MS: m/z calculated for C_32_H_45_N_7_O_3_ [M + H]^+^: 576.4, found: 576.3.

**Figure:**
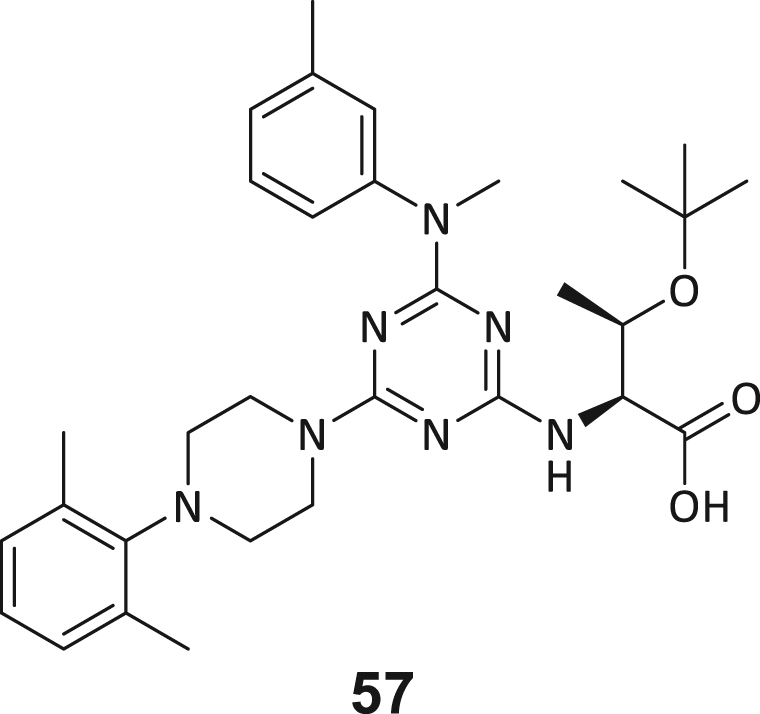

Compound **57**: Compound **56** (88 mg, 0.152 mmol, 1.0 eq.) was dissolved in 1 mL water and 1 mL THF, and sodium hydroxide (30 mg, 0.750 mmol, 5.0 eq.) was added. The mixture was stirred at RT for 48 h. The solvents were removed under reduced pressure and the crude product was purified by reversed-phase column chromatography (Biotage Isolera, ACN/H_2_O + TFA).

Yield: 15.0 mg (0.027 mmol ≙ 18% of th.), colorless solid.

^1^H-NMR (300 MHz, C*D*Cl3): δ [ppm] = 7.39 (t, *J* = 7.5 Hz, 1H), 7.06–6.95 (m, 7H), 4.55 (d, *J* = 7.9 Hz, 1H), 4.21 (dd, *J* = 6.4, 2.3 Hz, 1H), 3.92 (s, 3H), 3.47 (s, 3H), 3.12 (t, *J* = 5.4 Hz, 4H), 2.37 (d, *J* = 8.3 Hz, 3H), 2.33 (s, 7H), 1.16 (d, *J* = 20.0 Hz, 12H).

^13^C-NMR (75 MHz, *C*DCl_3_): δ [ppm] = 184.5, 161.2, 147.6, 137.0, 136.8, 129.3, 129.3, 127.5, 125.9, 123.9, 77.4, 76.9, 75.3, 68.8, 67.0, 49.6, 46.0, 45.9, 39.4, 28.1, 21.4, 20.4, 19.8.

LC/MS: m/z calculated for C_31_H_43_N_7_O_3_ [M + H]^+^: 562.3, found: 562.3.

**Figure:**
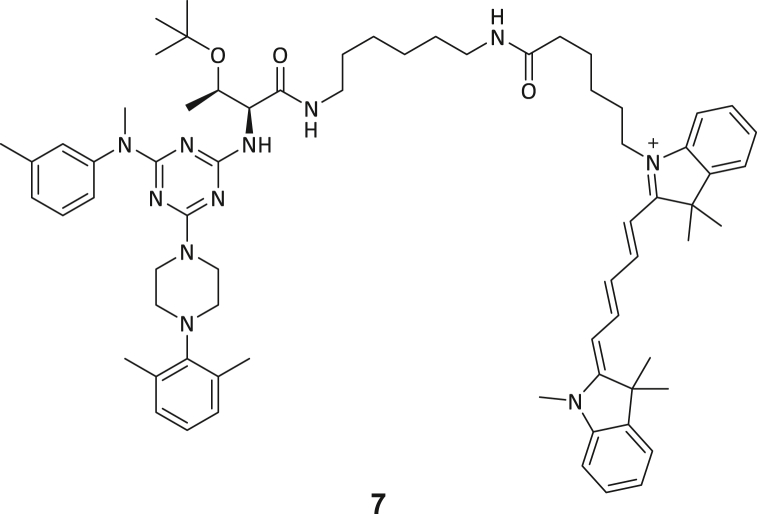

Compound **7**: Compound **57** (1.0 mg, 1.700 μmol, 1.0 eq.), Cy5 amine (1.0 mg, 1.700 μmol, 1.0 eq.), HATU (1 mg, 2.000 μmol, 1.2 eq.), and DIPEA (3 μL, 16.000 μmol, 9.5 eq.) were dissolved in 20 μL anhydrous DMF. The reaction was allowed to incubate for 16 h. The crude product was purified by reversed-phase column chromatography (Biotage Isolera, ACN/H_2_O + TFA).

Yield: 1.0 mg (0.817 μmol ≙ 48% of th.), blue solid.

LC/MS: m/z calculated for C_69_H_94_N_11_O_3_^+^ [M + H]^2+^: 562.8, found: 562.9.

**Figure:**
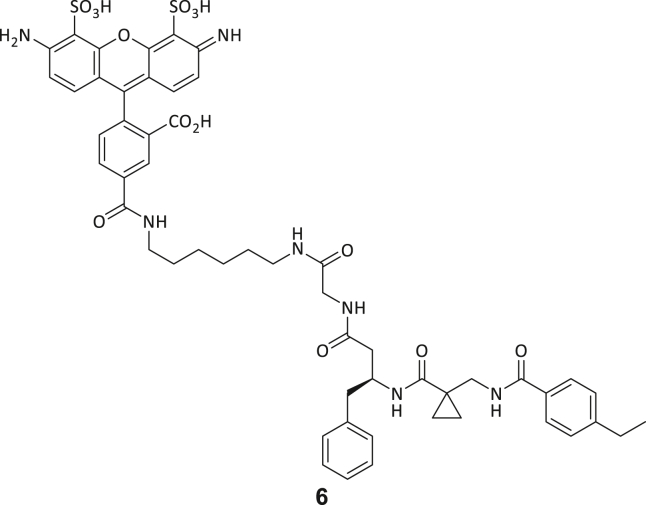

Compound **6**: Compound **54** (0.8 mg, 1.640 μmol, 1.2 eq.), AF 488 amine (1.0 mg, 1.390 μmol, 1.0 eq.), TBTU (0.5 mg, 1.680 μmol, 1.2 eq.), and DIPEA (2.3 μL, 13.200 μmol, 9.5 eq.) were dissolved in anhydrous DMF (0.6 mL) and stirred at RT for 48 h. The crude product was purified by reversed-phase column chromatography (Biotage Isolera, ACN/H_2_O + TFA).

Yield: 0.4 mg (0.37 mmol ≙ 27% of th.), orange solid.

^1^H-NMR (600 MHz, DMSO-*d*_6_): δ [ppm] = 9.00 (s, 2H), 8.86 (s, 1H), 8.69 (s, 1H), 8.58 (t, *J* = 6.2 Hz, 1H), 8.50 (s, 2H), 8.26 (dd, *J* = 8.0, 1.8 Hz, 1H), 8.16 (t, *J* = 6.0 Hz, 1H), 8.03 (d, *J* = 8.1 Hz, 1H), 7.81–7.73 (m, 2H), 7.50 (d, *J* = 7.8 Hz, 1H), 7.31 (d, *J* = 8.0 Hz, 1H), 7.15–7.11 (m, 2H), 7.11–7.06 (m, 1H), 7.02–6.88 (m, 3H), 4.28 (dt, *J* = 14.2, 6.9 Hz, 1H), 3.69–3.60 (m, 1H), 3.58–3.50 (m, 1H), 3.50–3.45 (m, 2H), 3.33–3.22 (m, 1H), 3.07–2.99 (m, 2H), 2.78 (dd, *J* = 13.6, 5.2 Hz, 1H), 2.70–2.59 (m, 3H), 2.38 (p, *J* = 1.8 Hz, 1H), 2.31 (d, *J* = 6.9 Hz, 2H), 2.11 (s, 1H), 2.08 (s, 1H), 2.04–1.91 (m, 2H), 1.58–1.48 (m, 2H), 1.38 (q, *J* = 9.4 Hz, 2H), 1.35–1.20 (m, 5H), 1.19 (t, *J* = 7.6 Hz, 2H), 1.13 (s, 1H), 0.91–0.69 (m, 4H).

LC/MS: m/z calculated for C_53_H_57_N_7_O_14_S_2_ [M+2H]^2+^: 540.7 found: 540.6.

**Figure:**
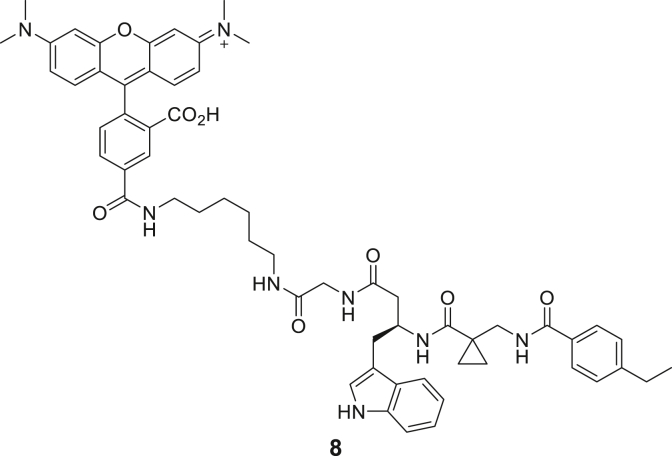

Compound **8**: Compound **9** (1.1 mg, 2.112 μmol, 1.2 eq.), TBTU (0.7 mg, 2.112 μmol, 1.2 eq.), and TAMRA-amine (1.0 mg, 1.760 μmol, 1.0 eq.) were dissolved in DMF (3 mL), and DIPEA (0.4 μL, 2.112 μmol, 1.2 eq.) was added. The reaction mixture was shaken for 16 h at RT. Afterward, the solvent was removed under reduced pressure and the crude product was purified by reversed-phase flash column chromatography (Biotage Isolera, ACN/H_2_O + TFA).

Yield: 1.9 mg (1.871 μmol ≙ 89% of th.), red solid.

^1^H-NMR (600 MHz, MeO*D*): δ [ppm] = 8.51 (s, 1H), 8.02 (d, *J* = 7.8 Hz, 1H), 7.88 (d, *J* = 8.2 Hz, 1H), 7.68 (dd, *J* = 8.2, 5.1 Hz, 1H), 7.61 (dd, *J* = 15.0, 8.3 Hz, 2H), 7.50 (d, *J* = 7.9 Hz, 1H), 7.33–7.23 (m, 3H), 7.20–7.12 (m, 3H), 6.98–6.85 (m, 5H), 4.60 (s, 2H), 4.52 (dd, *J* = 14.4, 5.9 Hz, 1H), 3.73 (d, *J* = 16.7 Hz, 1H), 3.66 (s, 1H), 3.63–3.52 (m, 3H), 3.45–3.35 (m, 3H), 3.32 (t, 1H), 3.21 (d, *J* = 2.8 Hz, 6H), 3.17–3.06 (m, 2H), 2.98–2.88 (m, 2H), 2.70–2.60 (m, 4H), 2.52 (dd, *J* = 14.3, 4.9 Hz, 1H), 2.43 (dd, *J* = 14.4, 8.7 Hz, 1H), 1.62–1.54 (m, 2H), 1.49–1.41 (m, 2H), 1.39–1.28 (m, 4H), 1.27–1.16 (m, 6H), 1.06–0.97 (m, 2H), 0.93 (t, *J* = 7.4 Hz, 1H), 0.89–0.76 (m, 3H).

^13^C-NMR (151 MHz, MeOD): δ [ppm] = 175.0, 174.1, 171.6, 170.7, 169.0, 162.1, 159.0, 158.7, 149.8, 137.9, 132.5, 132.3, 131.0, 129.8, 129.1, 129.0, 128.9, 128.6, 128.5, 128.4, 124.2, 122.2, 119.6, 119.6, 115.1, 114.8, 112.2, 112.1, 97.3, 57.6, 57.5, 57.3, 52.6, 49.9, 49.6, 44.6, 43.7, 41.7, 40.9, 40.7, 40.2, 32.7, 31.1, 30.3, 29.9, 29.8, 27.5, 27.4, 26.9, 21.2, 17.4, 17.3, 17.2, 15.9, 15.9, 14.9, 14.6, 14.6, 14.2.

LC/MS: m/z calculated for C_59_H_67_N_8_O_8_^+^ [M + H]^2+^: 508.3, found: 508.4.

### Quantification and statistical analysis

For all experiments, details of quantification, statistical methods, and the software used are described in the corresponding figure legends and methods. All data are expressed as mean ± SEM. *N* values indicate the number of technical or biological replicates per experiment. Each experiment was performed at least in triplicate. The statistical significance of differences of tRNA modification levels was determined by Student’s t test with *p* values (∗*p* < 0.05, ∗∗*p* < 0.01, ∗∗∗*p* < 0.001, ∗∗∗∗*p* < 0.0001). All statistical test methods and analyses were performed using GraphPad Prism 8.05 and are shown in the figure legends. The dose-response curves were fit to four-parameter logistic (4-PL) curve using GraphPad Prism 8 (log[agonist] vs. response – variable slope; Equation: Y = Bottom + (Top–Bottom)/(1 + 10ˆ((LogEC_50_-X)∗HillSlope)).

## Supplemental information

Document S1. Figures S1–S23, Tables S1–S4, and Data S1

Table S5. DEL hit clustering spreadsheet
