# Supplementary material for: DNA-encoded library screening uncovers potent DNMT2 inhibitors targeting a cryptic allosteric binding site
Source: iScience. 2025 Aug 5;28(9):113300. doi: 10.1016/j.isci.2025.113300 (PMC12396291; doi:10.1016/j.isci.2025.113300)
Supplement: Document S1. Figures S1–S23, Tables S1–S4, and Data S1 [file mmc1.pdf]

iScience, Volume *28*

Supplemental information

DNA-encoded library screening uncovers potent DNMT2 inhibitors targeting a cryptic allosteric binding site

Ariane F. Frey, Merlin Schwan, Annabelle C. Weldert, Valerie Kadenbach, Jürgen Kopp, Zarina Nidoieva, Robert A. Zimmermann, Lukas Gleue, Collin Zimmer, Marko Jörg, Kristina Friedland, Mark Helm, Irmgard Sinning, and Fabian Barthels

Supplementary data of DNA encoded library screening

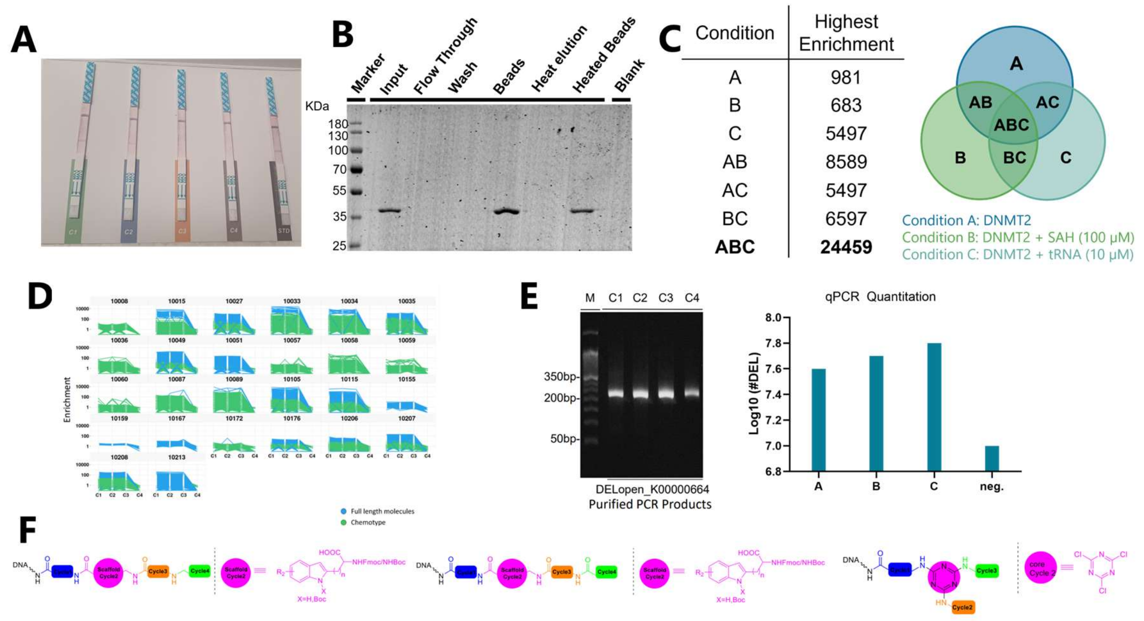

Figure S1: Supplementary data for the DNA encoded library screening on DNMT2. (**A**) Quality control test as provided by Wuxi AppTec. The four tested conditions mentioned in the manuscript are labeled here as C1–C4 (in the manuscript A–D). STD represents the internal standard according to the WuXi protocol (https://hits.wuxiapptec.com/assets/pdf/DELopen_Protocol.pdf). (**B**) DNMT2 capture assay was performed according to the protocol instructions to verify that DNMT2 can be stably immobilized on magnetic beads. The labeling is according to the original protocol. (**C**) Total count of highest enrichment scores for different panning conditions. The conditions are explained in the Venn diagram. (**D**) Overall performance and enrichment scores of the different libraries after the removal of NTC (no target control) binders. Most hits were enriched across C1–C3 equally, meaning neither SAH nor tRNA led to DEL hit displacement. (**E**) Quality control (gel electrophoresis and qPCR) was performed by Wuxi AppTec for all selection conditions after the second panning round. (**F**) Structures of the libraries featuring hit compounds chosen for off-DNA synthesis and further investigations.

Summary of all synthesized compound 3 analogs

Table S1: Overview of all synthesized compounds during the SAR study around compound 3 and their characterization by MST (F_{norm}) and ITC (K_D). All results include the mean value and standard deviations from at least technical triplicate measurements. Raw data and analysis plots are depicted in Figures S2–S5.

Cpd	BB1	BB2	BB3	BB4	C-Term.	F_{norm} (% displ.)	K_D (ITC) [µM]
**3**	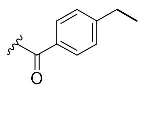 (4-ethylbenzoyl)	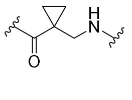 (cyclopropyl-β-alanine)	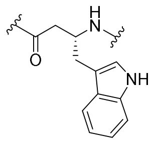 (β-homo-(*R*)-tryptophane)	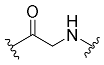 (glycine)	NH-Me	912.1 (53%)	5.31 ± 1.67
**5**	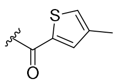	cyclopropyl-β-alanine	β-homo-(*R*)-tryptophane	glycine	NH-Me	879.3 (13%)	13.6 ± 1.6
**9**	4-ethylbenzoyl	cyclopropyl-β-alanine	β-homo-(*R*)-tryptophane	glycine	OH	907.7 (46%)	n.d.
**10**	4-ethylbenzoyl	cyclopropyl-β-alanine	β-homo-(*R*)-tryptophane	glycine	NH_2	903.3 (40%)	n.d.
**11**	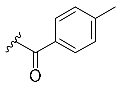	cyclopropyl-β-alanine	β-homo-(*R*)-phenylalanine	glycine	NH_2	891.2 (24%)	n.d.
**12**	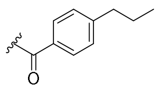	cyclopropyl-β-alanine	β-homo-(*R*)-phenylalanine	glycine	NH_2	890.4 (23%)	n.d.
**13**	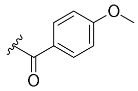	cyclopropyl-β-alanine	β-homo-(*R*)-phenylalanine	glycine	NH_2	897.7 (32%)	n.d.

**14**	4-ethylbenzoyl	cyclopropyl-β-alanine	β-homo-(*R*)-phenylalanine	glycine	NH_2	896.0 (29%)	3.27 ± 0.97
**15**	4-ethylbenzoyl	cyclopropyl-β-alanine	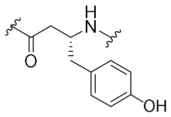	glycine	NH_2	862.9 (5%)	n.d.
**16**	4-ethylbenzoyl	cyclopropyl-β-alanine	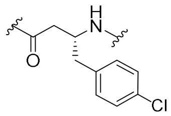	glycine	NH_2	928.7 (76%)	3.04 ± 0.57
**17**	4-ethylbenzoyl	cyclopropyl-β-alanine	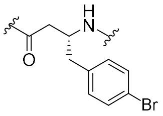	glycine	NH_2	924.6 (71%)	n.d.
**18**	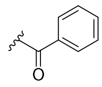	cyclopropyl-β-alanine	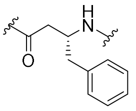 (β-homo-(*R*)-phenylalanine)	glycine	NH_2	868.2 (7%)	n.d.
**19**	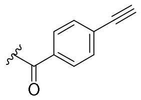	cyclopropyl-β-alanine	β-homo-(*R*)-phenylalanine	glycine	NH_2	871.4 (8%)	n.d.
**20**	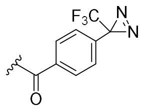	cyclopropyl-β-alanine	β-homo-(*R*)-phenylalanine	glycine	NH_2	876.5 (11%)	n.d.
**21**	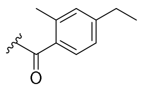	cyclopropyl-β-alanine	β-homo-(*R*)-phenylalanine	glycine	NH_2	862.4 (5%)	n.d.
**22**	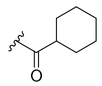	cyclopropyl-β-alanine	β-homo-(*R*)-phenylalanine	glycine	NH_2	864.4 (5%)	n.d.

**23**	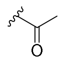	cyclopropyl-β-alanine	β-homo-(*R*)-phenylalanine	glycine	NH_2	864.4 (5%)	n.d.
**24**	4-ethylbenzoyl	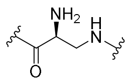	β-homo-(*R*)-tryptophane	glycine	NH_2	860.8 (4%)	n.d.
**25**	4-ethylbenzoyl	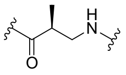	β-homo-(*R*)-tryptophane	glycine	NH_2	855.5 (3%)	n.d.
**26**	4-ethylbenzoyl	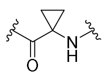	β-homo-(*R*)-tryptophane	glycine	NH_2	861.9 (5%)	n.d.
**27**	4-ethylbenzoyl	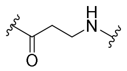	β-homo-(*R*)-tryptophane	glycine	NH_2	863.8 (5%)	n.d.
**28**	4-ethylbenzoyl	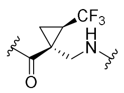	β-homo-(*R*)-phenylalanine	glycine	NH_2	871.9 (8%)	n.d.
**29**	4-ethylbenzoyl	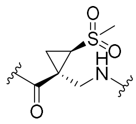	β-homo-(*R*)-phenylalanine	glycine	NH_2	872.3 (9%)	n.d.
**30**	4-ethylbenzoyl	cyclopropyl-β-alanine	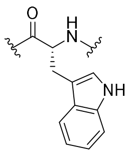	glycine	NH_2	861.5 (5%)	n.b.
**31**	4-ethylbenzoyl	cyclopropyl-β-alanine	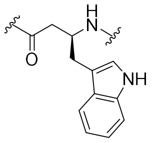	glycine	NH_2	857.0 (4%)	n.d.
**32**	4-ethylbenzoyl	cyclopropyl-β-alanine	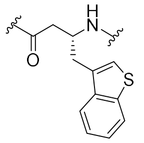	glycine	NH_2	856.7 (3%)	n.d.

**33**	4-ethylbenzoyl	cyclopropyl-β-alanine	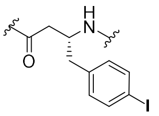	glycine	NH_2	885.6 (18%)	n.d.
**34**	4-ethylbenzoyl	cyclopropyl-β-alanine	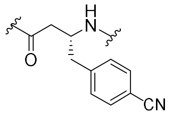	glycine	NH_2	879.4 (13%)	n.d.
**35**	4-ethylbenzoyl	cyclopropyl-β-alanine	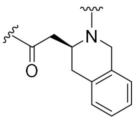	glycine	NH_2	861.5 (5%)	n.d.
**36**	4-ethylbenzoyl	cyclopropyl-β-alanine	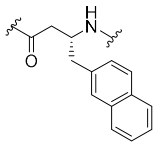	glycine	NH_2	861.7 (5%)	n.d.
**37**	4-ethylbenzoyl	cyclopropyl-β-alanine	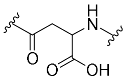	glycine	NH_2	858.6 (4%)	n.d.
**38**	4-ethylbenzoyl	cyclopropyl-β-alanine	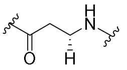	glycine	NH_2	859.4 (4%)	n.d.
**39**	4-ethylbenzoyl	cyclopropyl-β-alanine	β-homo-(*R*)-phenylalanine	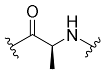	NH_2	871.4 (8%)	n.d.
**40**	4-ethylbenzoyl	cyclopropyl-β-alanine	β-homo-(*R*)-phenylalanine	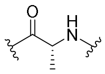	NH_2	870.1 (8%)	n.d.
**41**	4-ethylbenzoyl	cyclopropyl-β-alanine	β-homo-(*R*)-phenylalanine	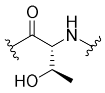	NH_2	865.3 (6%)	n.d.
**42**	4-ethylbenzoyl	cyclopropyl-β-alanine	β-homo-(*R*)-phenylalanine	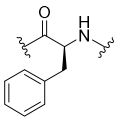	NH_2	858.8 (4%)	n.d.
**43**	4-ethylbenzoyl	cyclopropyl-β-alanine	β-homo-(*R*)-phenylalanine	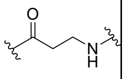	NH_2	874.7 (10%)	n.d.

**44**	4-ethylbenzoyl	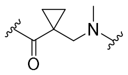	β-homo-(*R*)-phenylalanine	glycine	NH_2	869.7 (7%)	n.d.
**45**	4-ethylbenzoyl	cyclopropyl-β-alanine	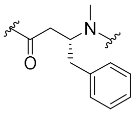	glycine	NH_2	862.6 (5%)	n.d.
**46**	4-ethylbenzoyl	cyclopropyl-β-alanine	β-homo-(*R*)-phenylalanine	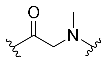	NH_2	868.4 (7%)	n.d.
**47**	4-ethylbenzoyl	cyclopropyl-β-alanine	β-homo-(*R*)-phenylalanine	glycine	NMe_2	901.0 (36%)	n.d.
**48**	4-ethylbenzoyl	cyclopropyl-β-alanine	β-homo-(*R*)-phenylalanine	glycine	NH-PEG4	894.1 (27%)	n.d.

MST supplementary data

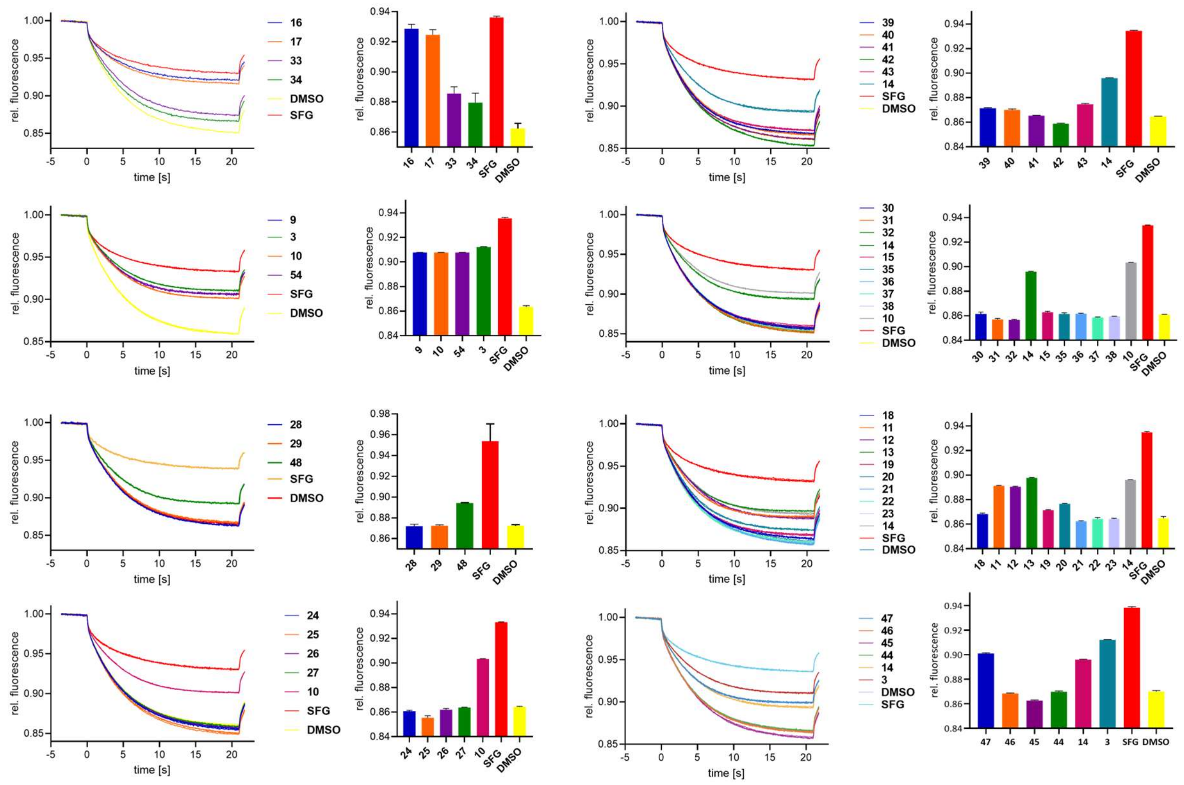

Figure S2: MST screening results of all compounds synthesized during the SAR study. Compounds were measured at a concentration of 100 µM. MST experiments were performed as described in the methods section using the fluorescent FTAD probe.

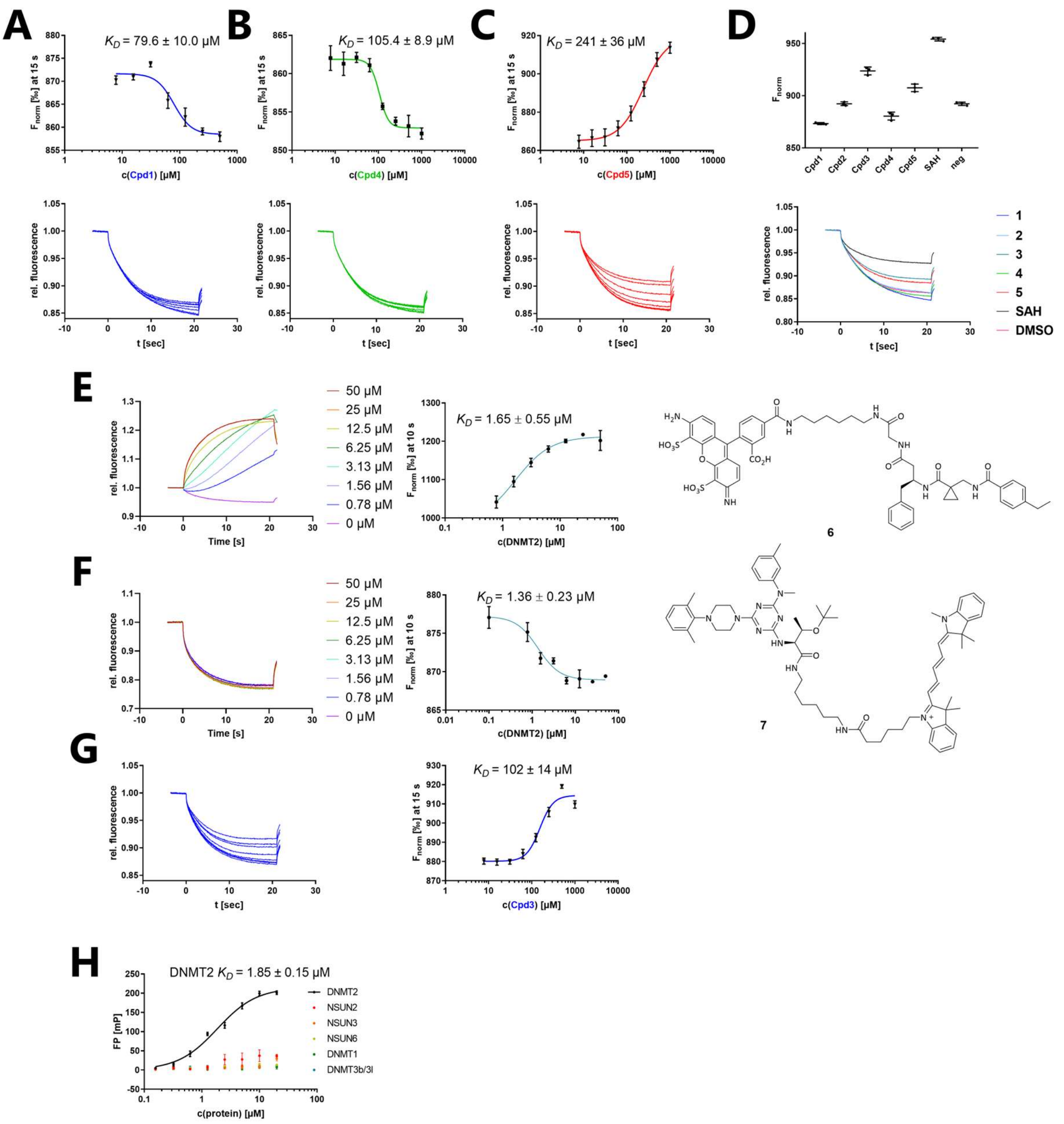

Figure S3: Supplementary MST and FP results for the hit compounds selected from the DEL screening. MST experiments were performed as described in the methods section using the fluorescent FTAD probe. (**A**) K_D-value determination of compound **1**. (**B**) K_D-value determination of compound **4**. (**C**) K_D-value determination of compound **5**. (**D**) Screening of all hit compounds **1–5** from the DEL Screening at 100 µM. (**E**) K_D-value determination of probe **6** by dose-response experiments using variable concentrations of DNMT2. (**F**) K_D-value determination of probe **7** by dose-response experiments using variable concentrations of DNMT2. (**G**) K_D-value determination of compound **3** using the DNMT2 deletion mutant DNMT2Δ47 (2 µM). (**H**) FP assay for probe **6** binding to native DNMT2 and various related MTase off-targets as determined by dose-response experiments.

ITC supplementary data

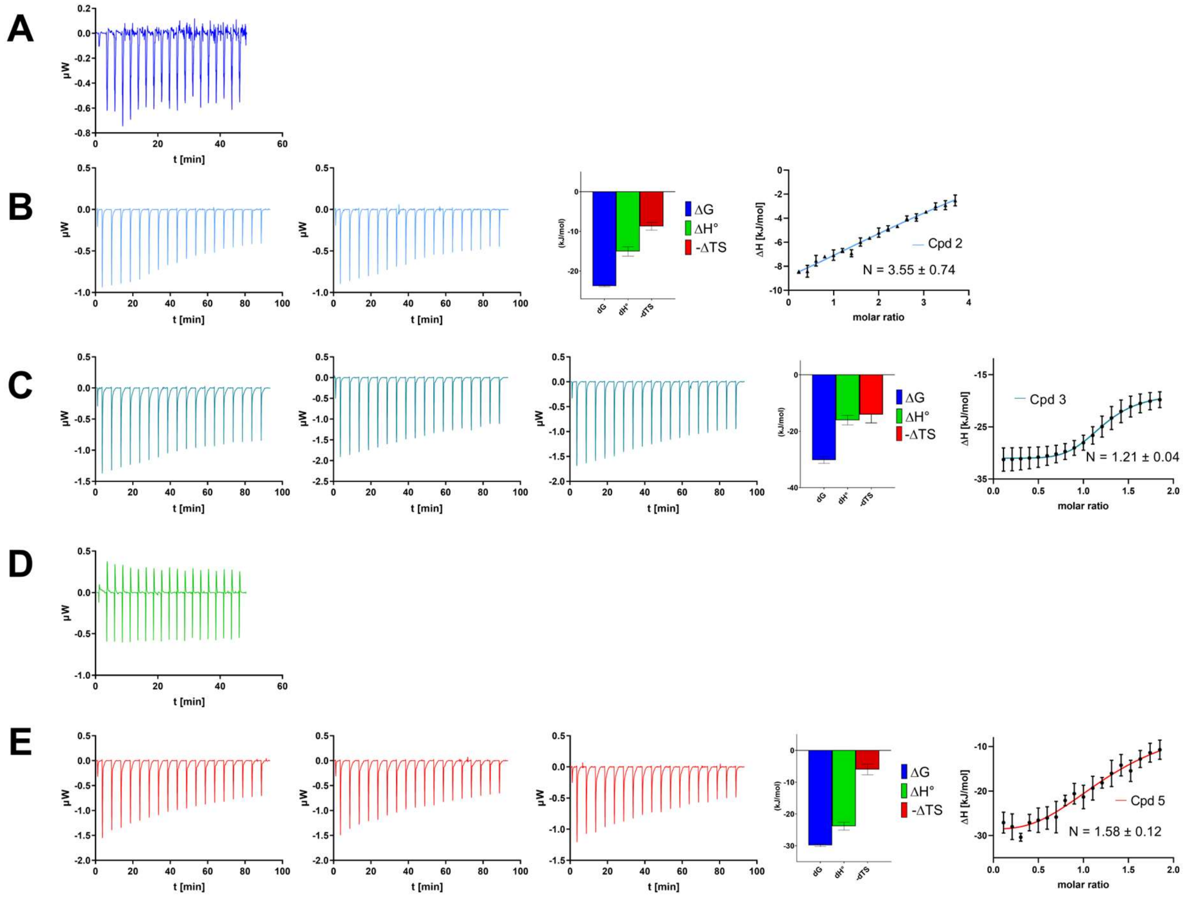

Figure S4: Supplementary ITC data for compounds 1–5. (**A**) Compound **1** was titrated into DNMT2. No enthalpy signal change was observed. (**B**) Compound **2** was titrated into DNMT2. Note: due to low solubility, the upper resp. lower limits of the stoichiometry plot are not reached, and thus, ΔH/ΔS determination might not be accurate. (**C**) Compound **3** was titrated into DNMT2. (**D**) Compound **4** was titrated into DNMT2. No enthalpy signal change was observed. (**E**) Compound **5** was titrated into DNMT2.

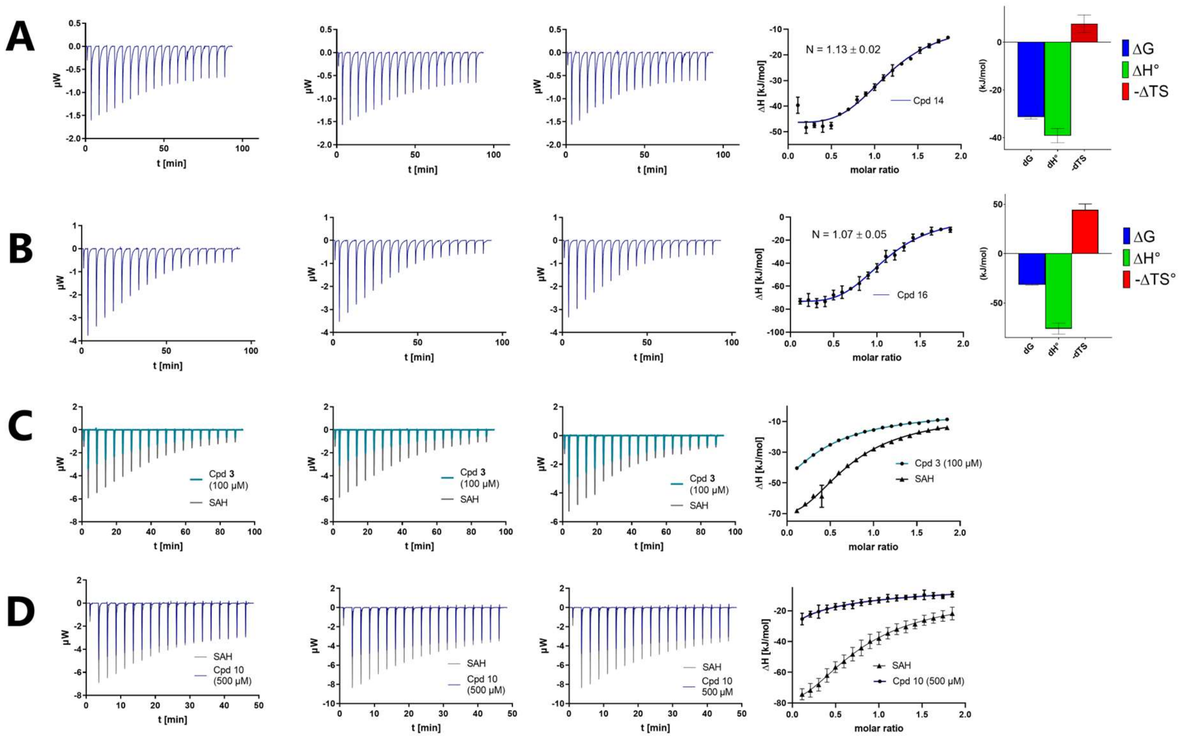

Figure S5: Supplementary ITC data for synthesized compound 3 analogs. (**A**) Compound **14** was determined by direct titration. (**B**) Compound **16** was determined by direct titration. (**C**) Compound **3** measured by SAH displacement. (**D**) Compound **10** measured by SAH displacement.

Affinity selection-mass spectrometry (AS-MS)

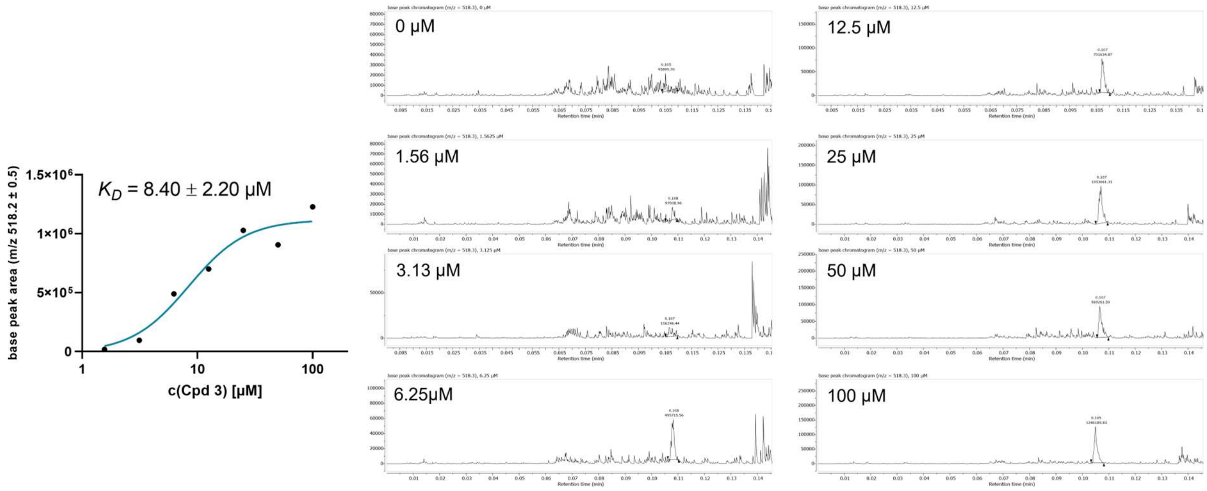

Figure S6: AS-MS-based determination of compound 3 binding affinity to DNMT2. (**left**) Dose-response curve for K_D determination. (**right**) Base peak chromatograms for compound **3** content analysis in the analyzed fractions.

Supplementary data of ^{3}H-based methyltransferase DNMT2 activity assays

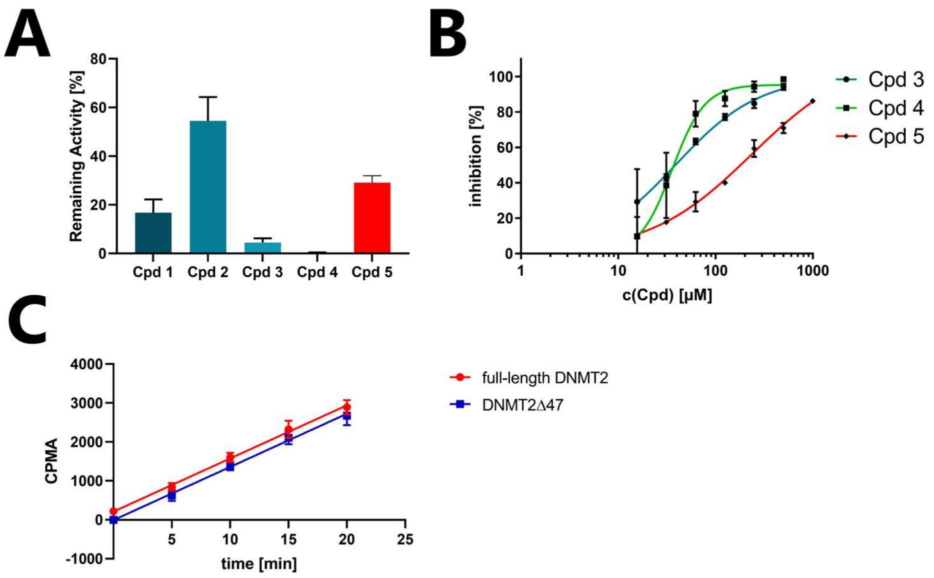

Figure S7: Data from ^{3}H-based methyltransferase enzyme activity assay. (**A**) Screening of compounds **1**–**5**, the remaining DNMT2 activity was determined at 500 µM of the respective compound. (**B**) IC_{50} determination for compounds **3**, **4**, and **5**. (**C**) ^{3}H-based methyltransferase enzyme activity assays with full-length and deletion mutant enzymes (250 nM DNMT2, 5 µM $tRNA^{Asp}$, 0.9 µM SAM), highlighting virtually identical substrate conversion plots (counts per minute [CPMA]) vs. time.

DNMT2-tRNA MST displacement experiments

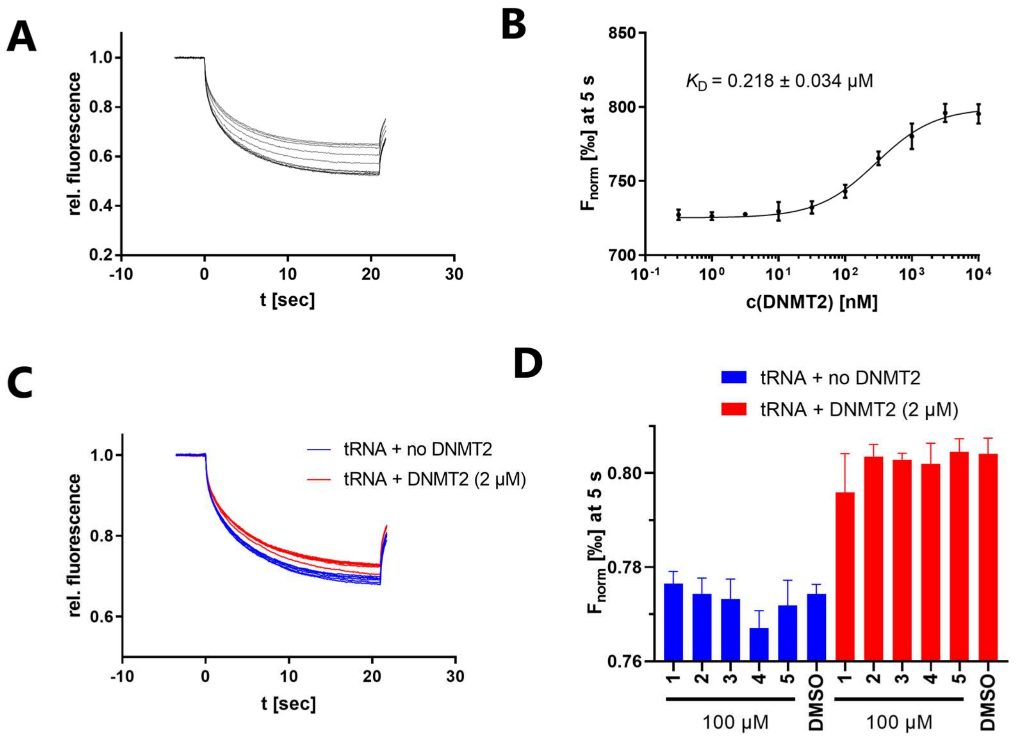

Figure S8: Analysis of DNMT2-RNA interaction by in situ labeling with SybrGold (1×). (**A**, **B**) Dissociation constant (K_D) of $tRNA^{Asp}$ (100 nM, labeled with SybrGold) towards DNMT2 (varying concentrations) was determined by dose-response experiments. (**C**, **D**). Neither the native $tRNA^{Asp}$ samples (blue) nor the DNMT2-tRNA complex (red) showed significant thermophoresis alterations when treated with the hit compounds **1**–**5**.

Cell viability assays

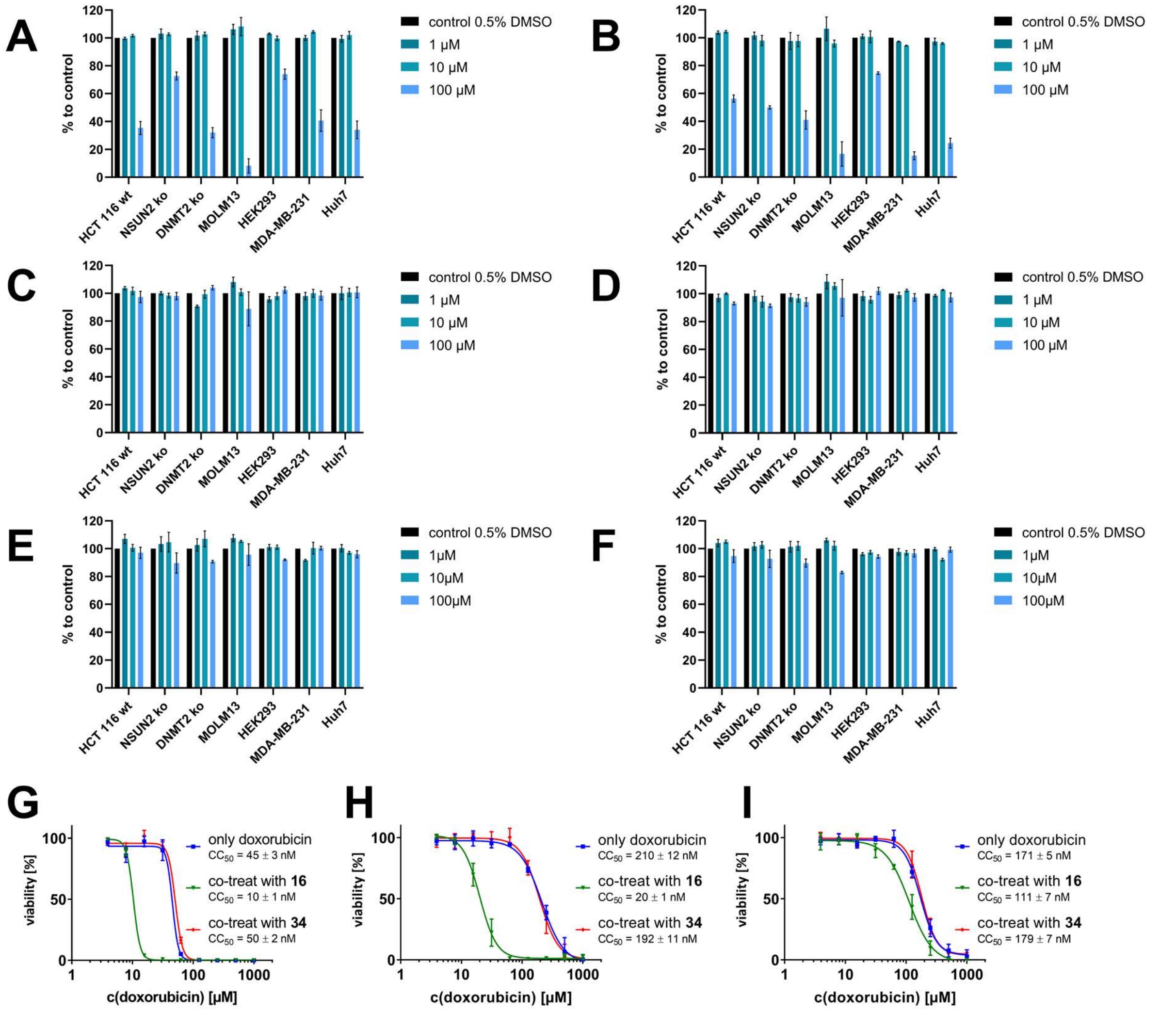

Figure S9: Cell viability assay data for compounds 1–5 and 16 used for the treatment of various cell lines. (**A**) Data for compound **1**. (**B**) Data for compound **2**. (**C**) Data for compound **3**. (**D**) Data for compound **4**. (**E**) Data for compound **5**. (**F**) Data for compound **16**. (**G**) Dose-response curves from cell viability assays of doxorubicin/compound **16** resp. **34** co-treated MOLM-13 cells, (**H**) cotreated MDA-MB-231 cells, and (**I**) cotreated Huh-7 cells.

Parallel Artificial Membrane Permeation Assay (PAMPA)

Table S2: PAMPA results of the investigated compounds determined in duplicates.

Compound	Compound structure	P_{app} [*10^{-6} cm/s]
**3**	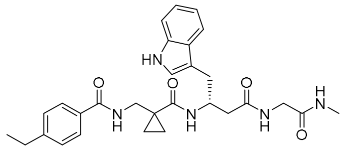	below detection limit
**10**	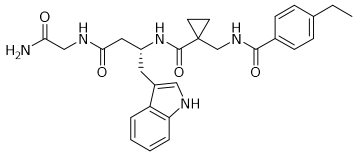	below detection limit
**14**	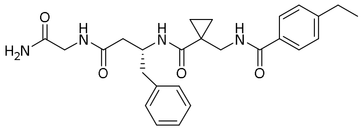	below detection limit
**16**	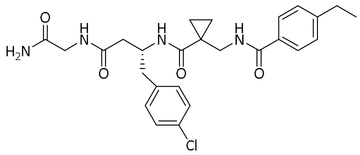	~0.30
**47**	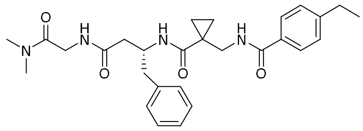	below detection limit
**54**	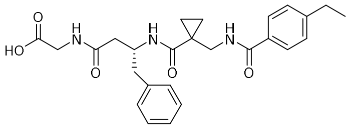	below detection limit

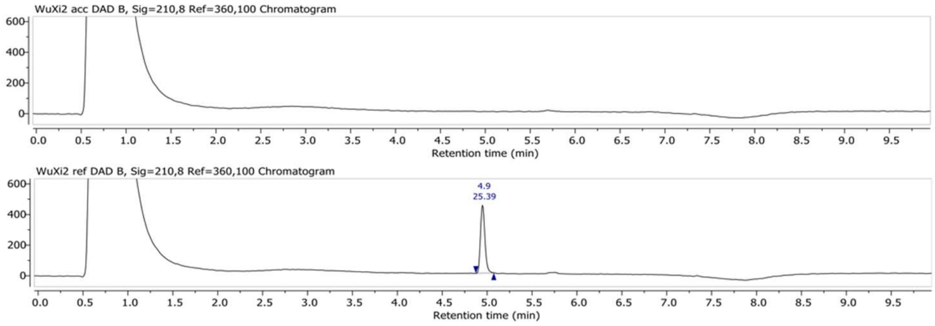

Figure S10: Base peak chromatogram at m/z = 518.3 ± 0.5 of acceptor and reference solution after incubating for compound 3. The peak of compound **3** is marked, and the area under the peak is stated above the peak.

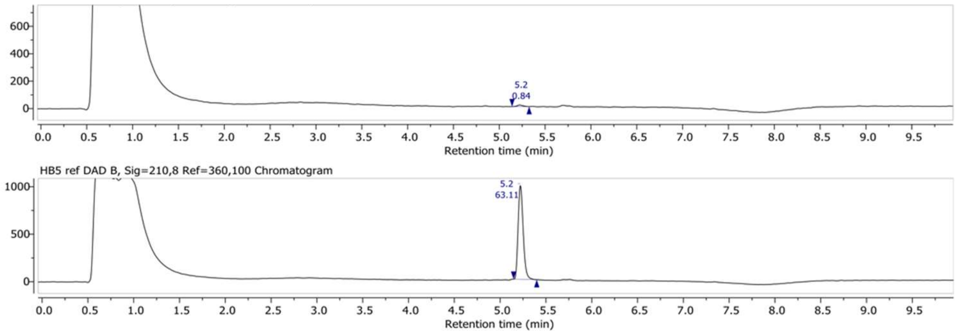

Figure S11: Base peak chromatogram at m/z = 499.2 ± 0.5 of acceptor and reference solution after incubating for compound 16. The peak of compound **16** is marked, and the area under the peak is stated above the peak.

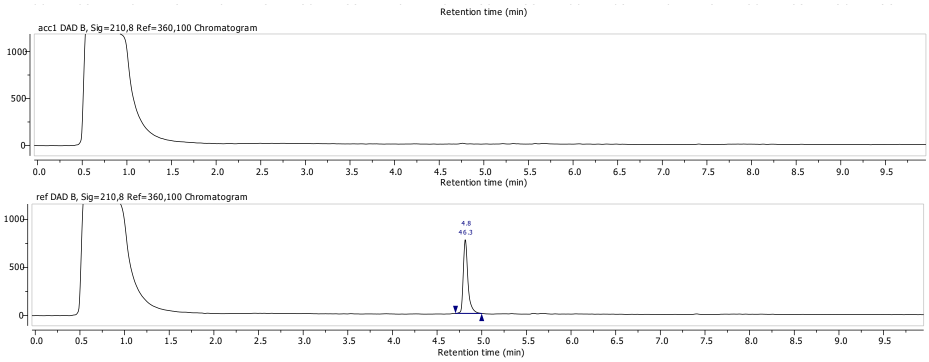

Figure S12: Base peak chromatogram at m/z = 504.3 ± 0.5 of acceptor and reference solution after incubating for compound 10. The peak of compound **10** is marked, and the area under the peak is stated above the peak.

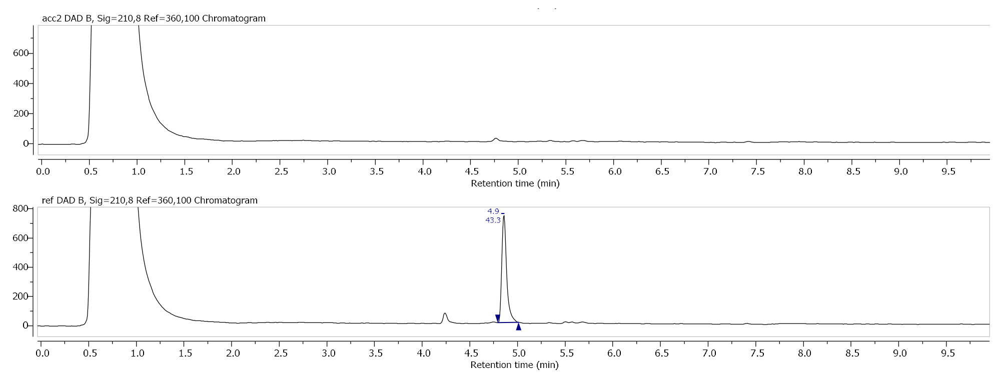

Figure S13: Base peak chromatogram at m/z = 465.3 ± 0.5 of acceptor and reference solution after incubating for compound 14. The peak of compound **14** is marked, and the area under the peak is stated above the peak.

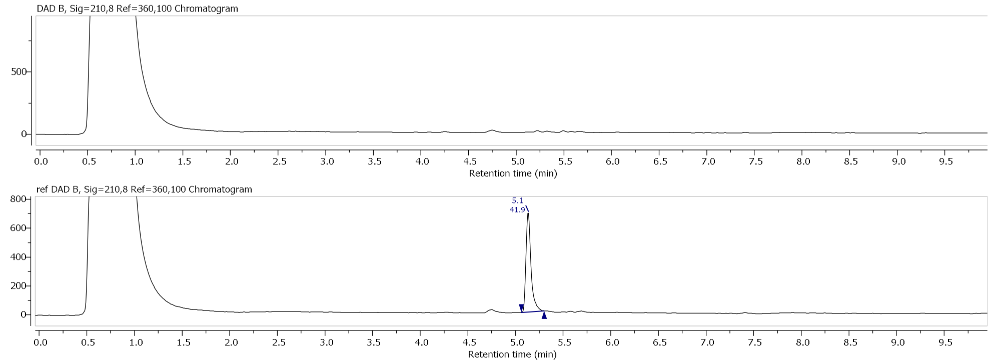

Figure S14: Base peak chromatogram at m/z = 493.3 ± 0.5 of acceptor and reference solution after incubating for compound 47. The peak of compound **47** is marked, and the area under the peak is stated above the peak.

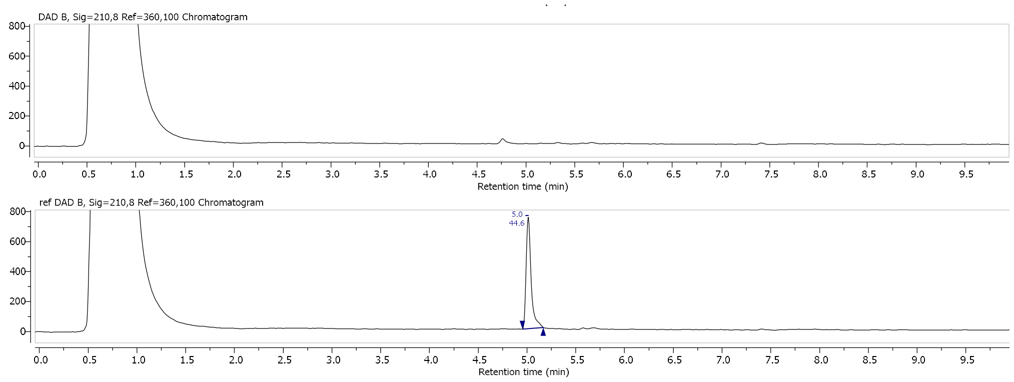

Figure S15: Base peak chromatogram at m/z = 466.2 ± 0.5 of acceptor and reference solution after incubating for compound 54. The peak of compound **54** is marked, and the area under the peak is stated above the peak.

Pull-down interaction assay

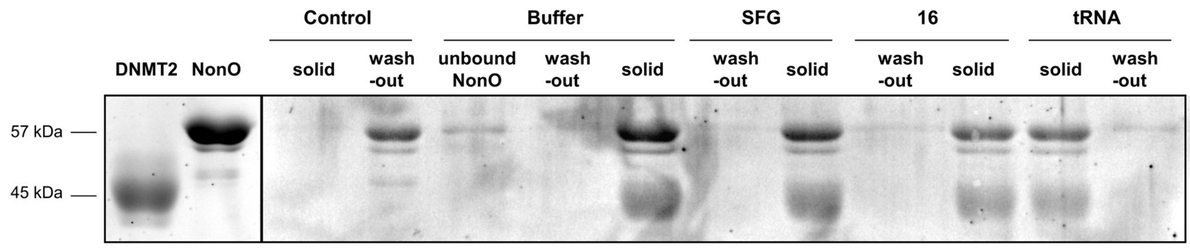

Figure S16: NonO does not bind to the allosteric pocket. Results of the SDS-PAGE-based analysis. Lane description from left to right: DNMT2/NonO: recombinant proteins for size comparison. References were applied at another gel position and merged in this figure; Control: beads without immobilized DNMT2 served as the negative control, i.e. on these beads no binding of NonO was observed. Treatments (buffer, SFG, **16**, tRNA): The lane "unbound NonO" contains the supernatant after immobilization of NonO, which showed low protein levels, thus most NonO is effectively immobilized on DNMT2 beads. Lanes labeled with (wash-out) contain the supernatant of the respective treatment conditions (buffer, 100 µM SFG, 100 µM **16**, 10 µM $tRNA^{Asp}$), while bands labeled with (solid) contain the solid fraction obtained by boiling the residual beads in Laemmli buffer. NonO could not be displaced from binding DNMT2 by competitive treatment with compound **16**, SFG, nor $tRNA^{Asp}$, indicating that NonO does not bind to the allosteric binding pocket identified. Uncropped PAGE images can be found in Figure S22.

Supplementary data for crystallographic investigations

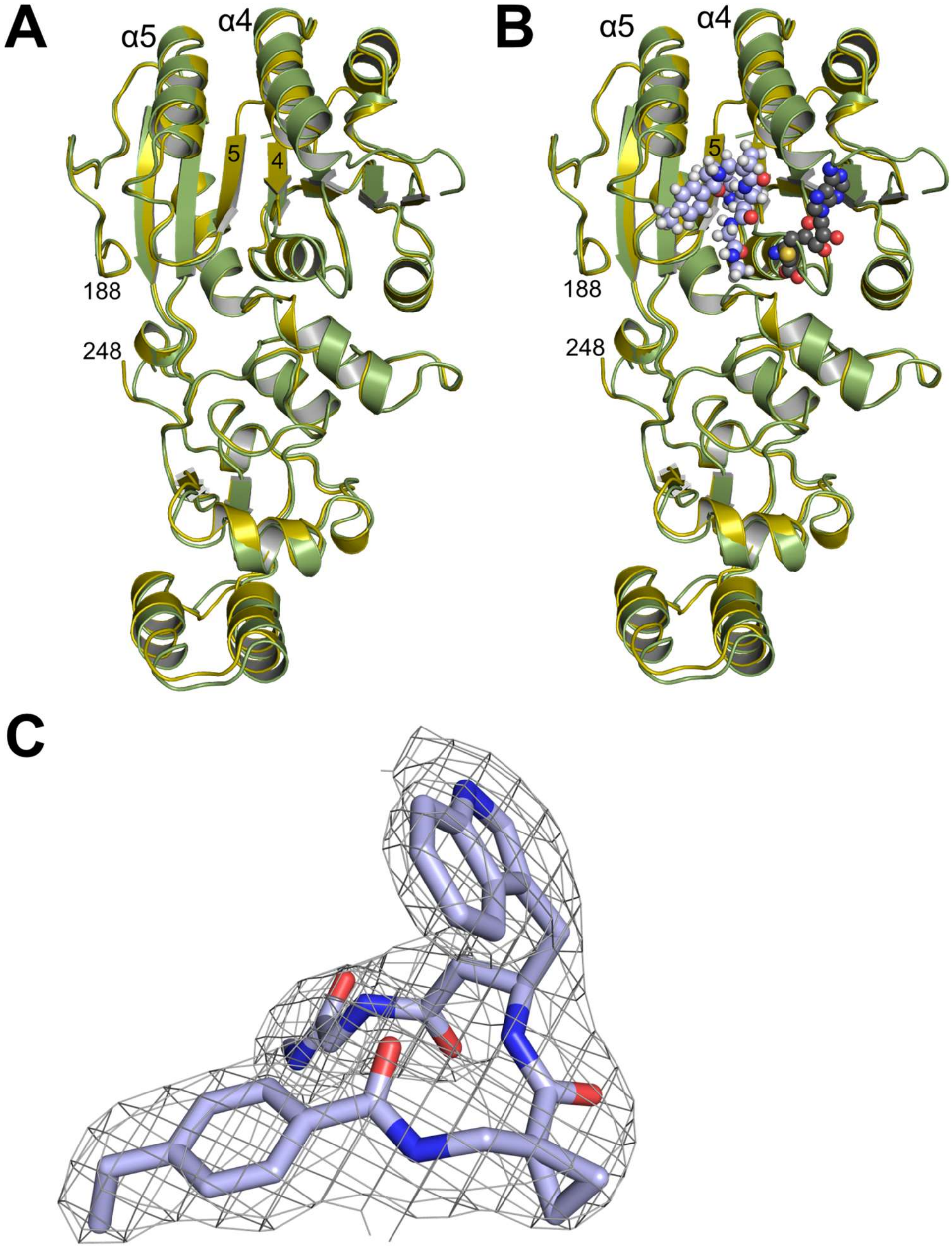

Figure S17: Global superposition of the X-ray structures of human DNMT2Δ47-compound 3 (green / light blue). Human DNMT2Δ47-SAH (yellow / grey) without (**A**) and with (**B**) ligands. (**C**) Compound **3** bound to DNMT2Δ47 (chain A) with $2mF_{obs}$-DF_{calc} electron density map at a contour level of +1σ.

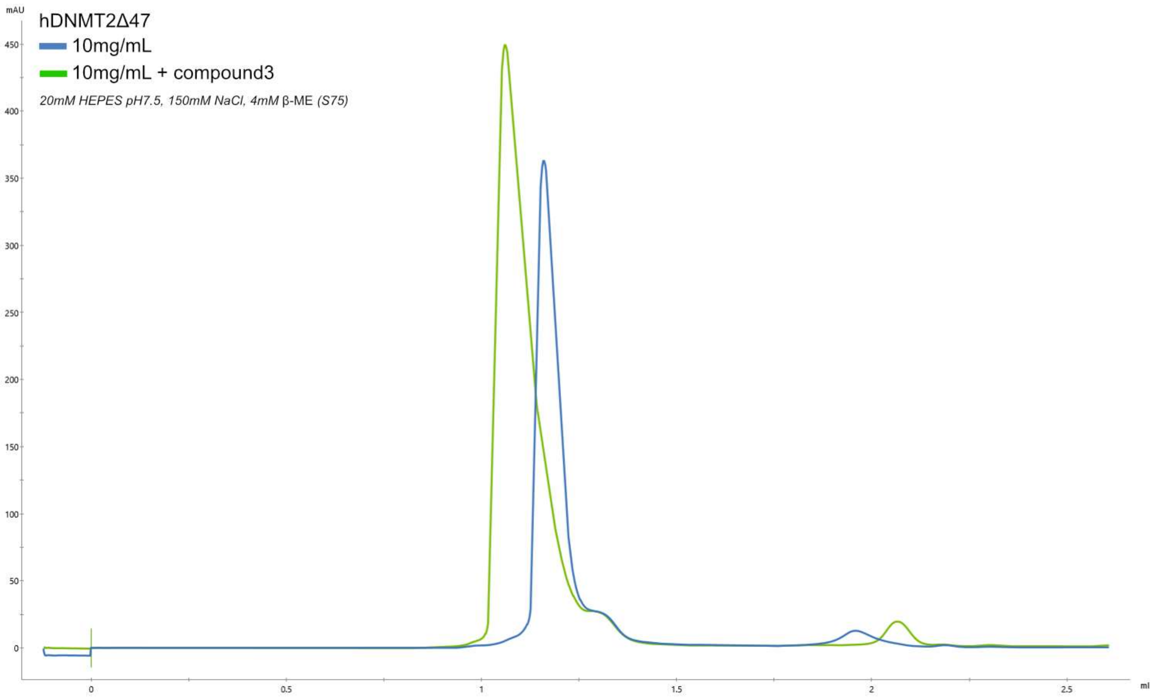

Figure S18: Analytical SEC elution profile of DNMT2Δ47 with and without compound 3. Superposition of two chromatograms showing the 280 nm signal of analytical SEC runs for hDNMT2Δ47 without ligand (blue) and pre-incubated with a 2-fold molar excess of compound **3** (green). For both runs the protein concentration was at 10 mg/mL as used for crystallization. A Superdex S75 3.2/300 column was used with a buffer consisting of 20 mM HEPES pH 7.5, 150 mM NaCl and 4 mM β-mercaptoethanol. The peak shift indicates the dimerization of DNMT2Δ47 upon compound **3** binding.

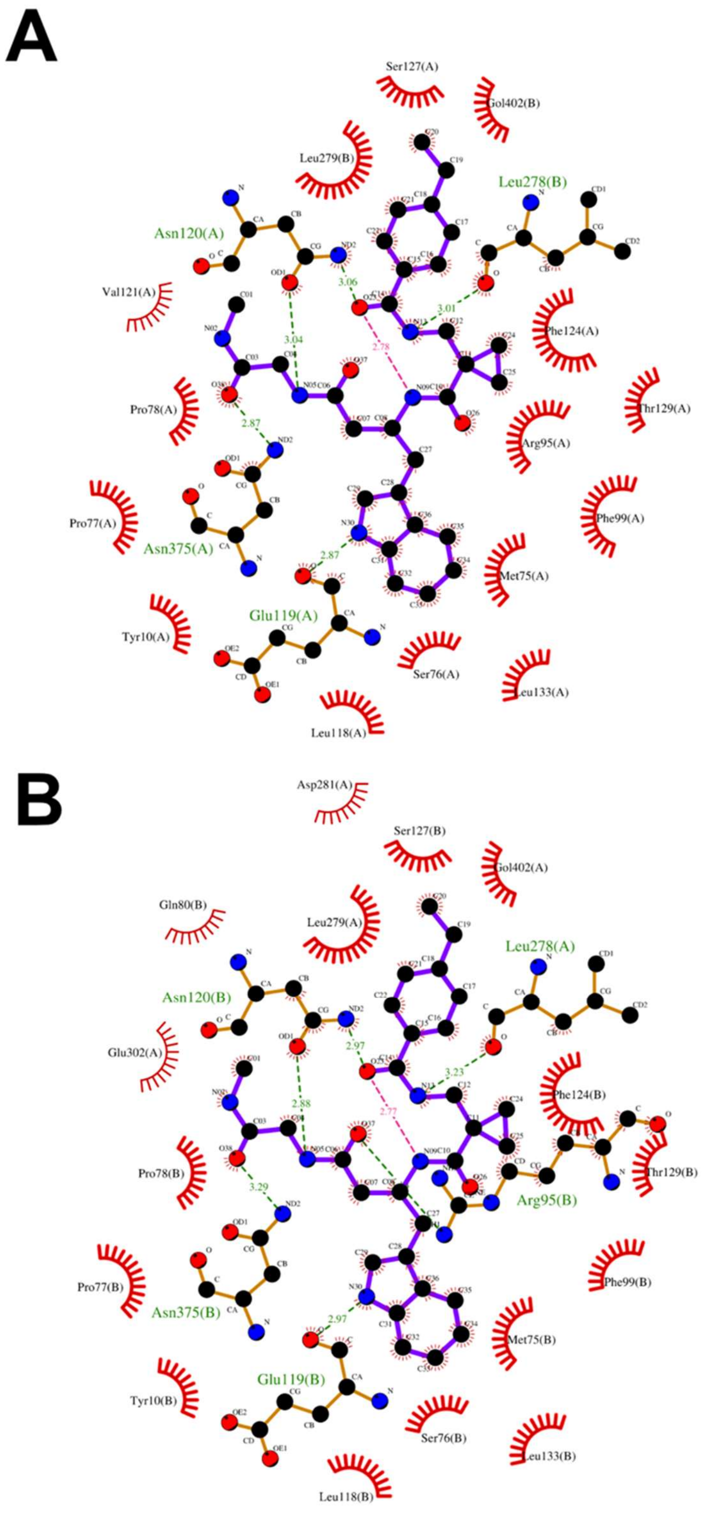

Figure S19: A schematic ligand-protein interaction diagram created with LigPlot+. For compound **3** bound to chain A (**A**) and chain B (**B**). Intermolecular hydrogen bonds are given in green, and the intramolecular hydrogen bond in compound **3** was added to the diagram in magenta.

Table S3: X-ray data collection refinement statistics.

**PDB entry 9HGM**	
**Data Collection**	
Beamline	ESRF Grenoble ID30B
Wavelength (Å)	0.8731
Resolution range (Å)	48.67-2.60 (2.72-2.60)
Space group	$P2_12_12_1$
a,b,c (Å)	71.20, 97.35, 125.02
α,β,ɣ (°)	90, 90, 90
Total reflections	263886 (33598)
Unique reflections	27445 (3300)
Multiplicity	9.6 (10.2)
Completeness (%)	100 (100)
Mean I/sigma(I)	10.6 (1.2)
R-merge	0.117 (1.966)
R-pim	0.042 (0.688)
CC1/2	0.999 (0.626)
**Refinement**	
Resolution range (Å)	48.67-2.60 (2.69-2.60)
Reflections used in refinement	27366 (2667)
Reflections used for R-free	1396 (132)
R-work	0.1995 (0.3377)
R-free	0.2454 (0.3798)
Number of non-hydrogen atoms	5410
Macromolecules	5276
Ligands	88
Solvent	46
Protein residues	656
RMS (bonds) (Å)	0.005
RMS (angles) (°)	0.580

Ramachandran favored (%)	97.4
Ramachandran allowed (%)	2.6
Ramachandran outliers (%)	0
Rotamer outliers (%)	0.84
Clashscore	2.02
Average B-factor (Å^2)	83.3
Macromolecules (Å^2)	83.6
Ligands (Å^2)	68.1
Solvent (Å^2)	74.1

Statistics for the highest-resolution shell are shown in parentheses.

Modeling of a ternary complex consisting of DNMT2-tRNA^{Asp} and small molecules

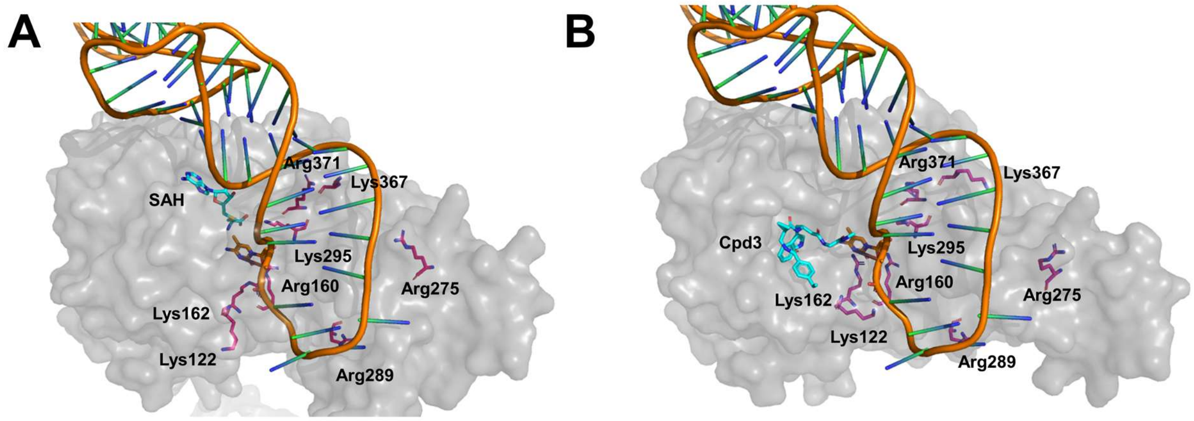

Figure S20: Modelling of ternary DNMT2-tRNA^{Asp} complexes. (**A**) Ternary complex (DNMT2-tRNA-SAH) modeled by AlphaFold, consisting of full-length DNMT2 (sequence from UniProt: O14717), tRNA^{Asp} (sequence: GGGUCGUUAGUAUAGUGGUGAGUAUCCCCGCCUGUCA{m5C}GCGGGAGACCGGGGUUCGAU UCCCCGACGGCCCGCCA), and SAH (relative coordinates from structural alignment with PDB 1G55). (**B**) Ternary complex (DNMT2-tRNA-cpd **3**) modelled by Schrödinger BioLuminate 5.8 consisting of compound **3**-bound DNMT2 (PDB 9HGM) and tRNA^{Asp}. The previously elucidated RNA-binding residues (K122, R160, R162, R275, R289, K295, K367, and R371) are at a significant distance from the allosteric binding pocket, and thus, allow the simultaneous binding of compound **3** and tRNA.

SeeSAR analysis by binding pose inspection of hit compound 3

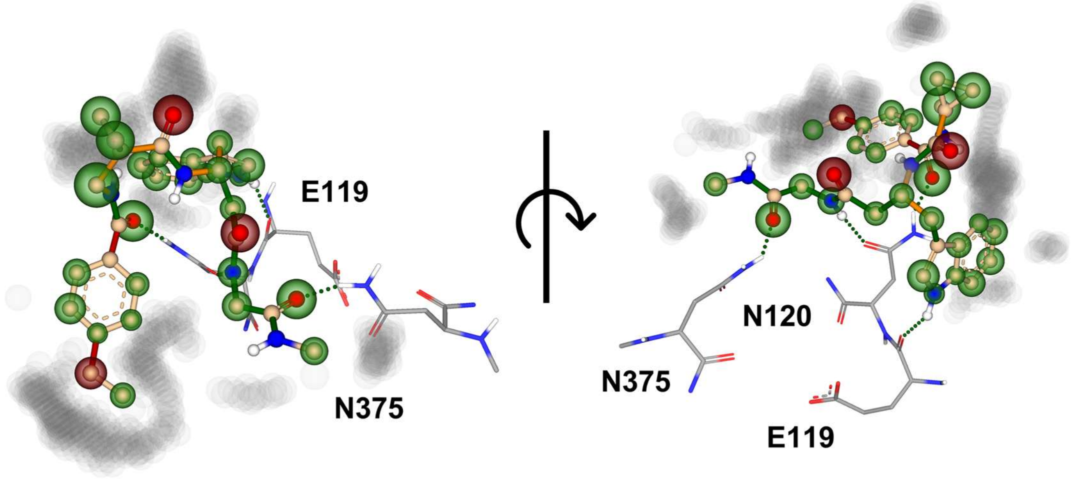

Figure S21: SeeSAR analysis by binding pose inspection of hit compound 3. Hyde coloring enabled the identification of contributing resp. unfavorable atom placements (colored spheres; green: contributing, red: unfavored); the peptide backbone's dihedral angles were evaluated for their torsional free energy (colored sticks). Characteristic hydrogen bonds are formed with the protein residues Glu119, Asn120, and Asn375. Potentially druggable yet unoccupied space in the protein binding pocket is highlighted by grey shadings.

Uncropped gel images

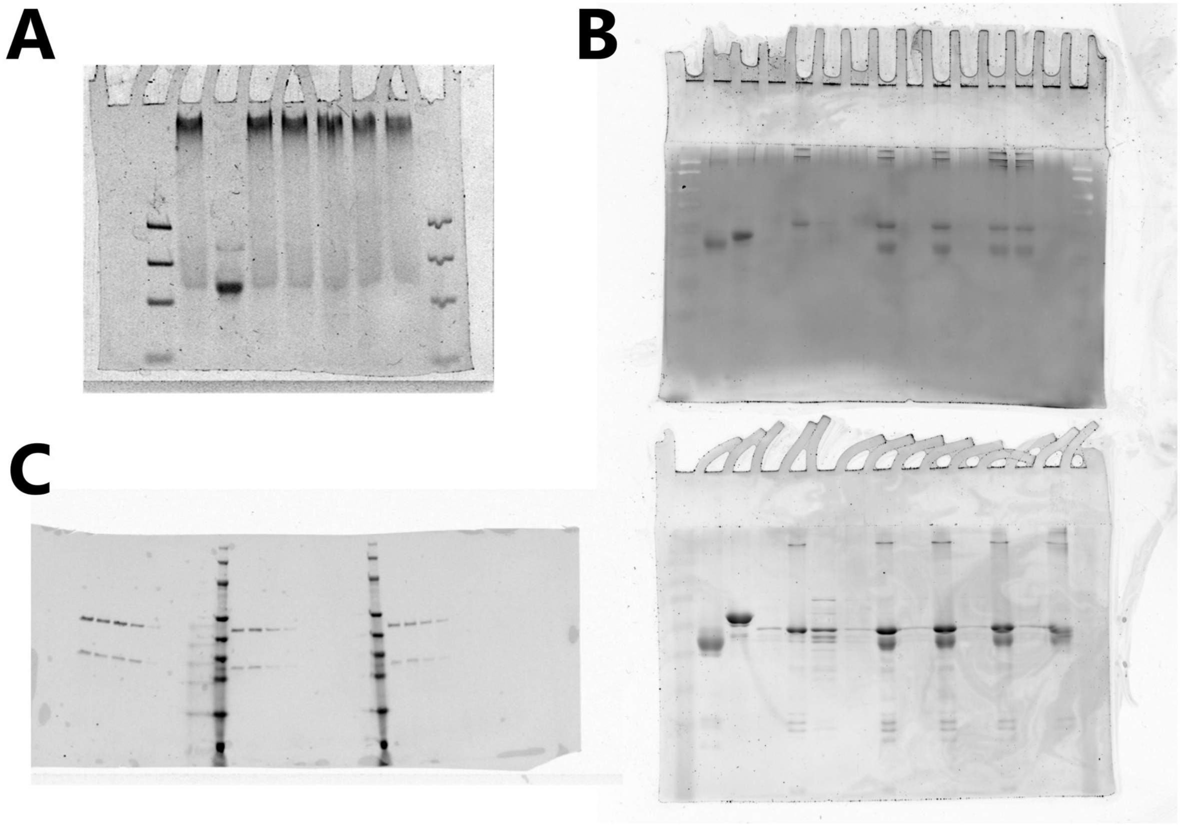

Figure S22: Uncropped PAGE and Western blot images. (**A**) EMSA. (**B**) DNMT2 interaction assays. (**C**) CETSA.

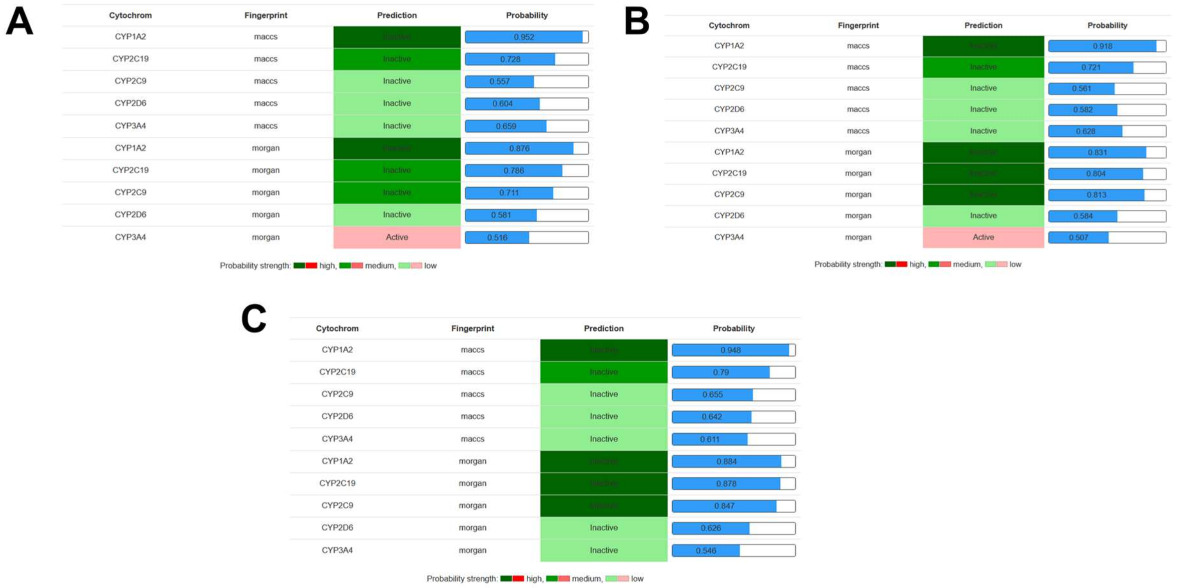

Figure S23: Cyp-enzyme reactivity profiles of compounds. 3 (**A**), **16** (**B**), and **34** (**C**) as determined by SuperCYPsPred.

Table S4: Overview of predicted physicochemical properties of cell evaluated compounds 3 (non-permeable control), 16 (cell-active), and 34 (taylor-made negative control). The values were obtained using the web tools molinspiration.com and SuperCYPsPred. Properties that violate Lipinski's Rule of Five are highlighted in red.

**Cpd.**	**structure**	**MW [g/mol]**	**logP**	**TPSA**	**#H-acceptors**	**#H-donors**	**rotatable bonds**
**3**	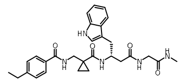	517.3	3.75	128.43	9	5	12
**16**	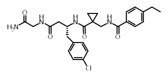	498.2	3.40	130.39	8	5	12
**34**	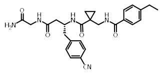	489.2	2.48	154.18	9	5	12

Data S1: Spectral appendix including NMR and LC/MS spectra of final compounds.

Compound 1:

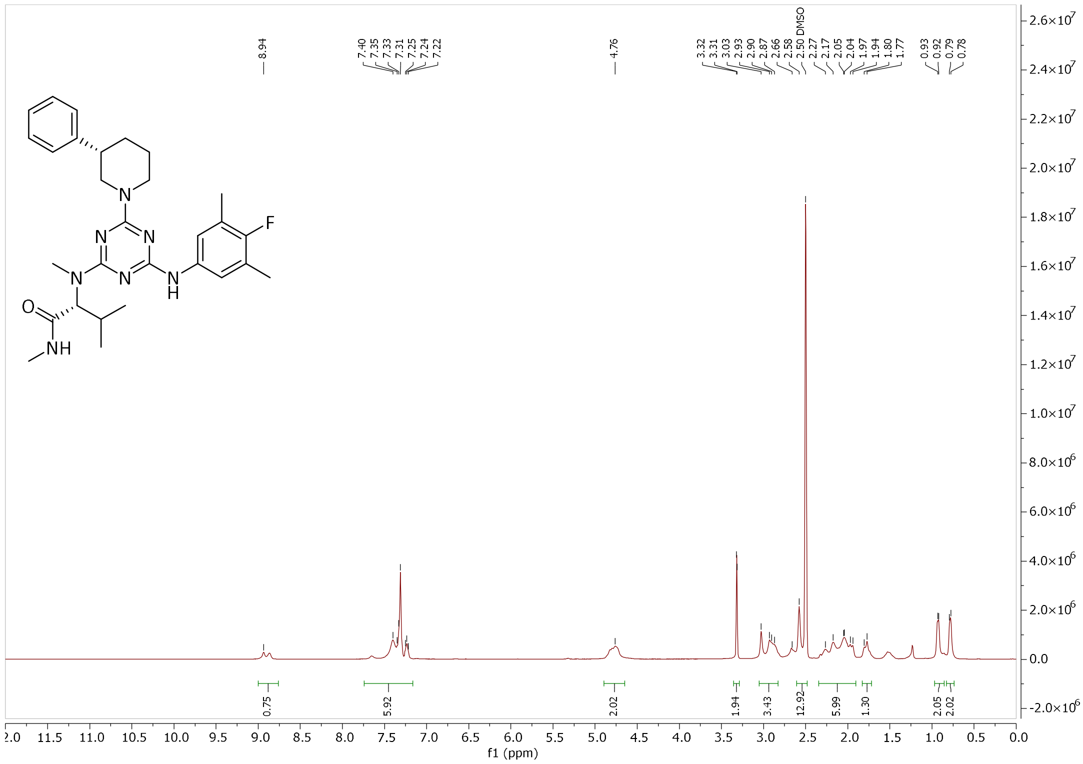

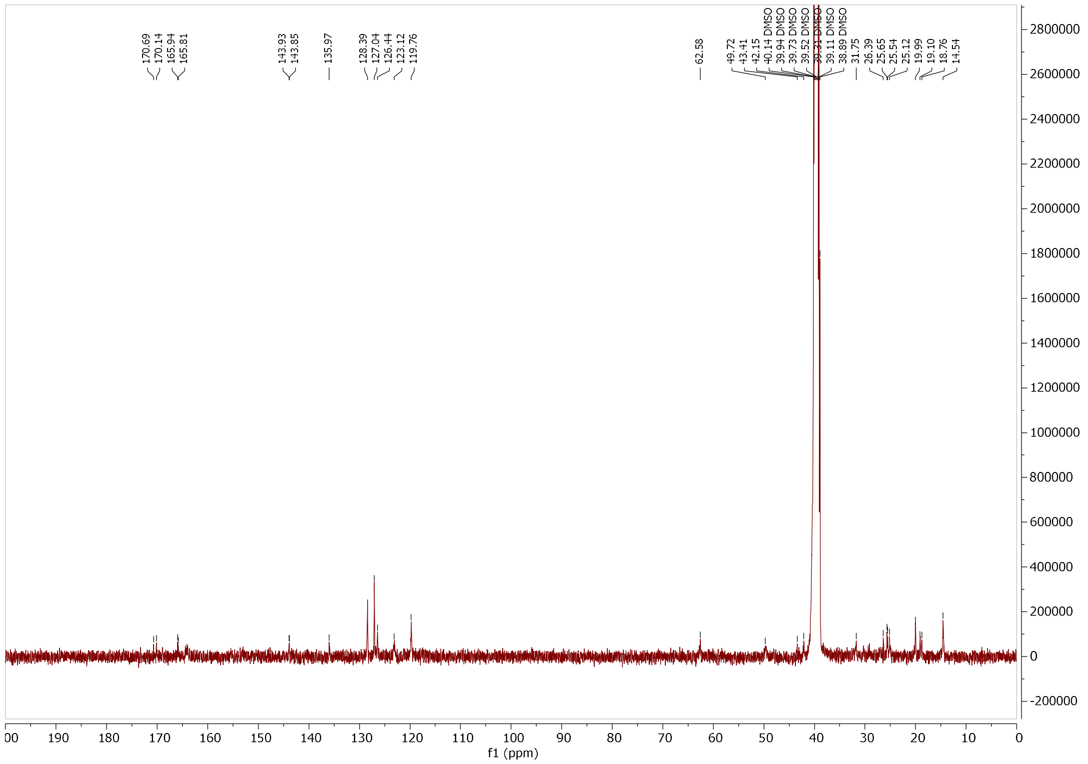

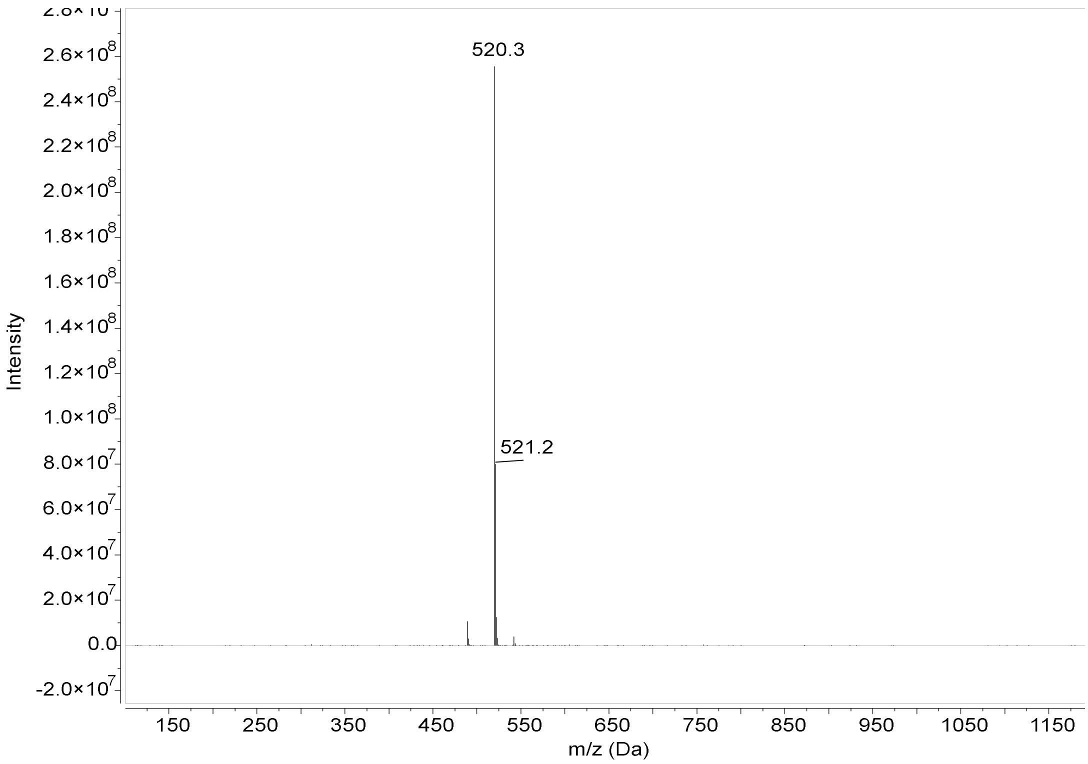
520.3
521.2
Intensity
m/z (Da)

Compound 2:

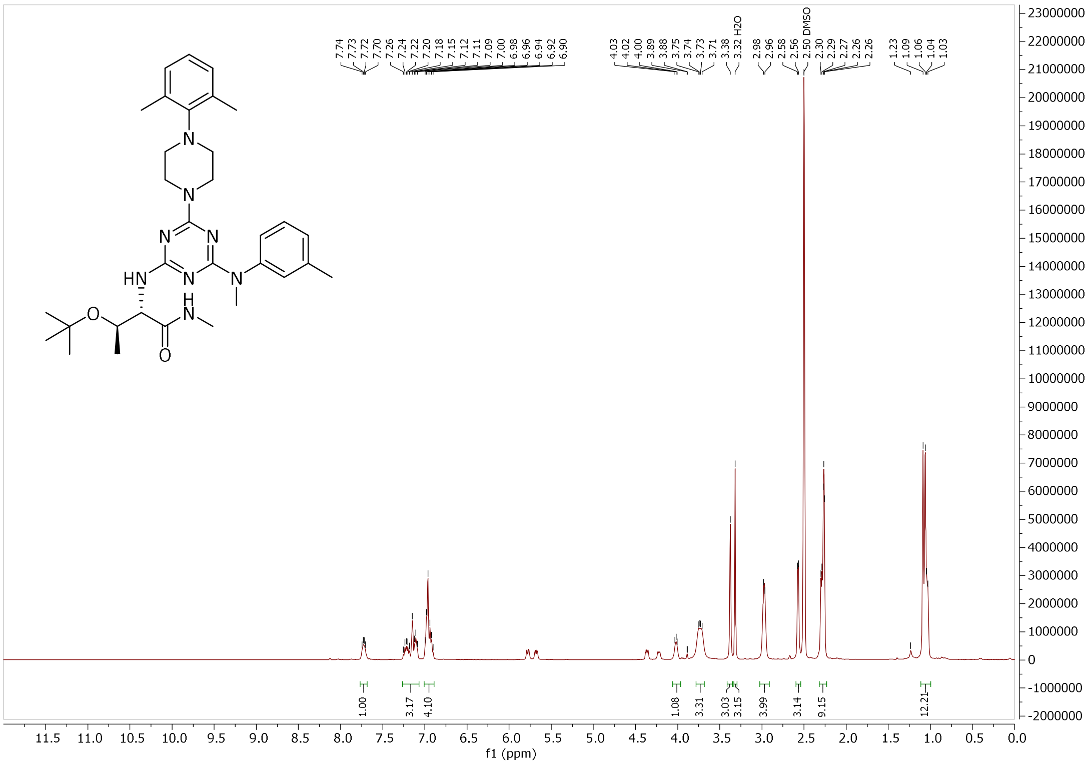

7.74 7.73 7.72 7.70 7.26 7.24 7.22 7.20 7.18 7.15 7.12 7.11 7.09 7.00 6.98 6.96 6.94 6.92 6.90
4.03 4.02 4.00 3.89 3.88 3.75 3.74 3.73 3.71 3.38 3.32 H2O
2.98 2.96 2.58 2.56 2.50 DMSO 2.30 2.29 2.27 2.26 2.26
1.23 1.09 1.06 1.04 1.03
1.00 3.17 4.10 1.08 3.31 3.03 3.15 3.99 3.14 9.15 12.21
f1 (ppm)

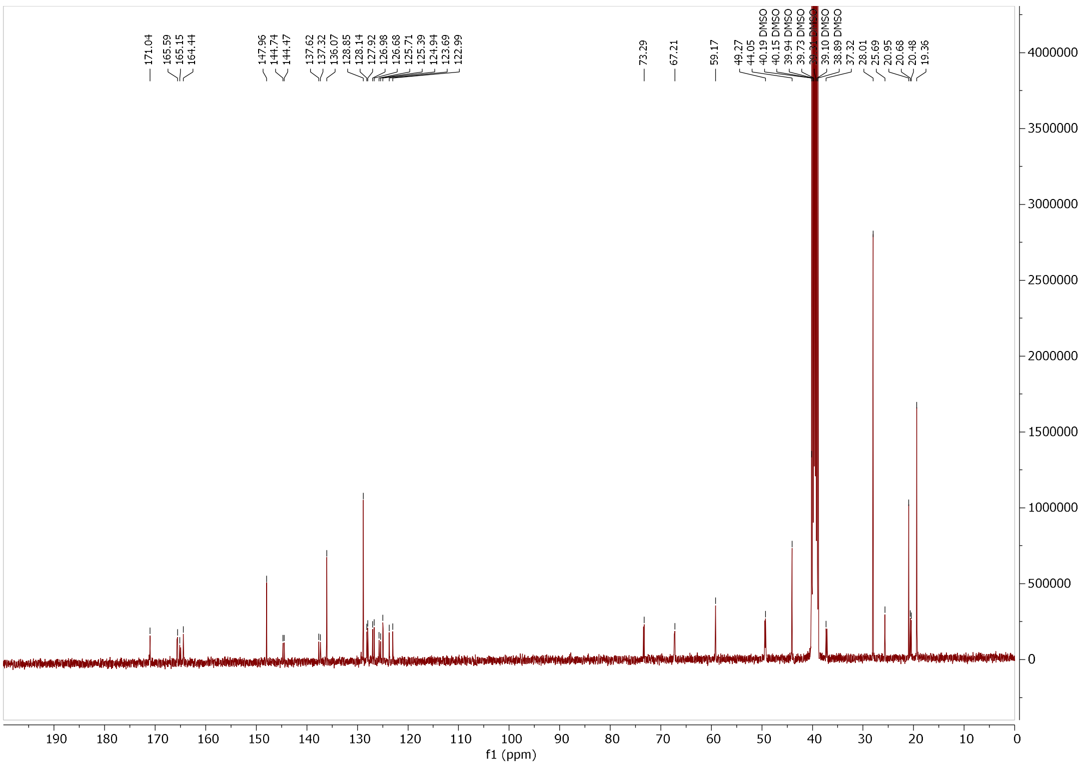

171.04 165.59 165.15 164.44
147.96 144.74 144.47
137.62 137.32 136.07 128.85 128.14 127.92 126.98 126.68 125.71 125.39 124.94 123.69 122.99
73.29 67.21 59.17
49.27 44.05 40.19 DMSO 40.15 DMSO 39.94 DMSO 39.73 DMSO 39.10 DMSO 38.89 DMSO 37.32 28.01 25.69 20.95 20.68 20.48 19.36
f1 (ppm)

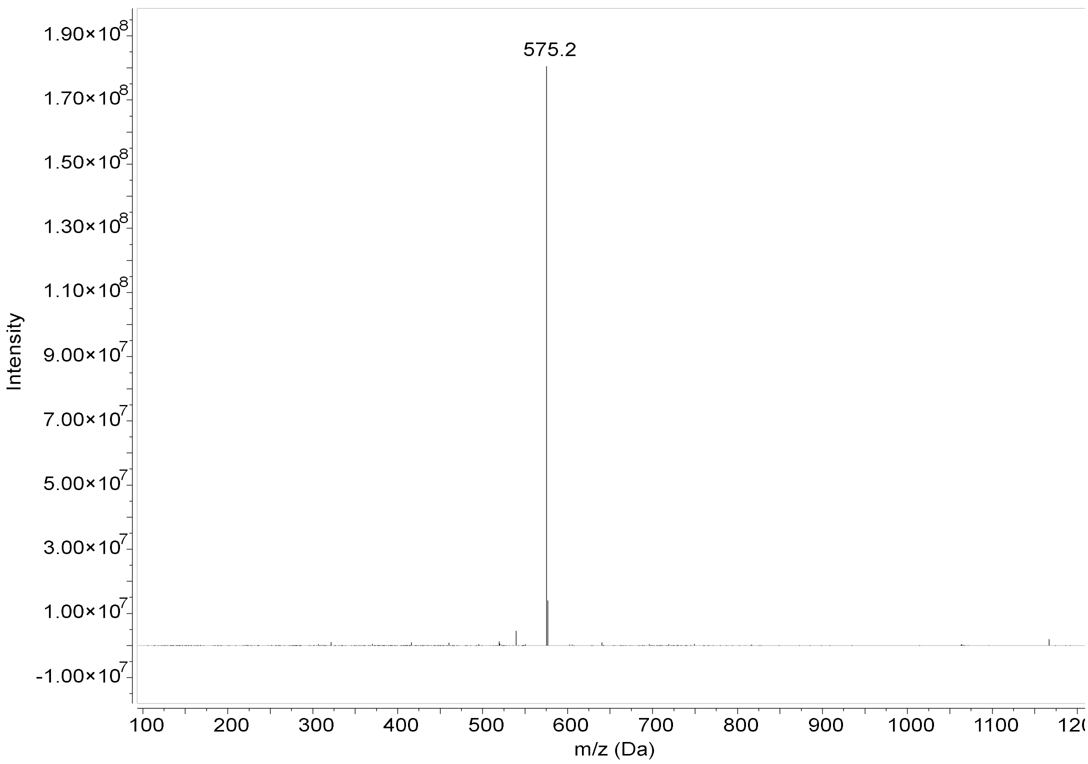
575.2
Intensity
1.90×10^8
1.70×10^8
1.50×10^8
1.30×10^8
1.10×10^8
9.00×10^7
7.00×10^7
5.00×10^7
3.00×10^7
1.00×10^7
-1.00×10^7
100
200
300
400
500
600
700
800
900
1000
1100
m/z (Da)

Compound 3:

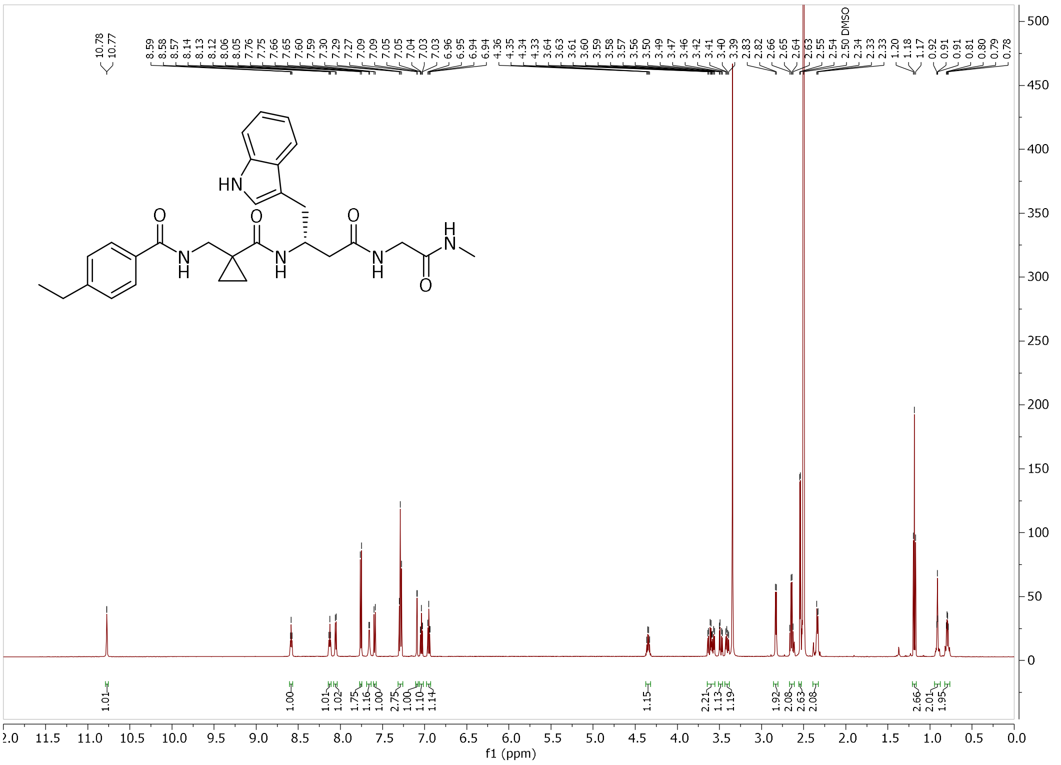

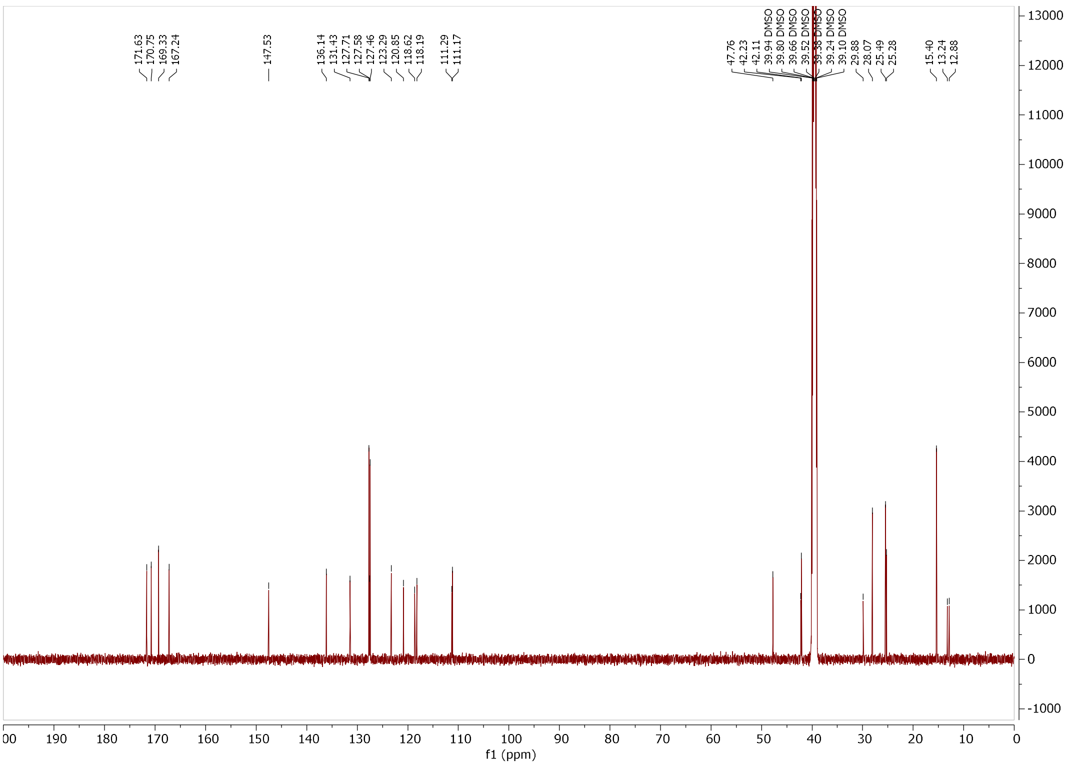

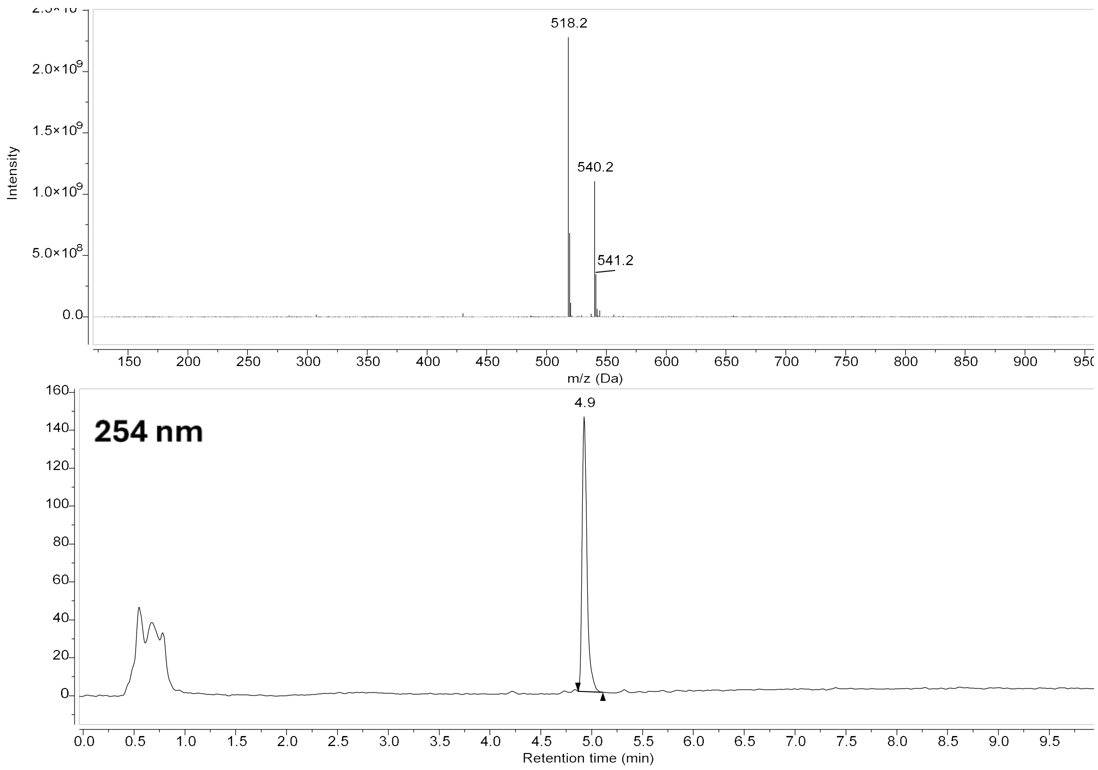
518.2
540.2
541.2
Intensity
m/z (Da)
254 nm
4.9
Retention time (min)

Compound 4:

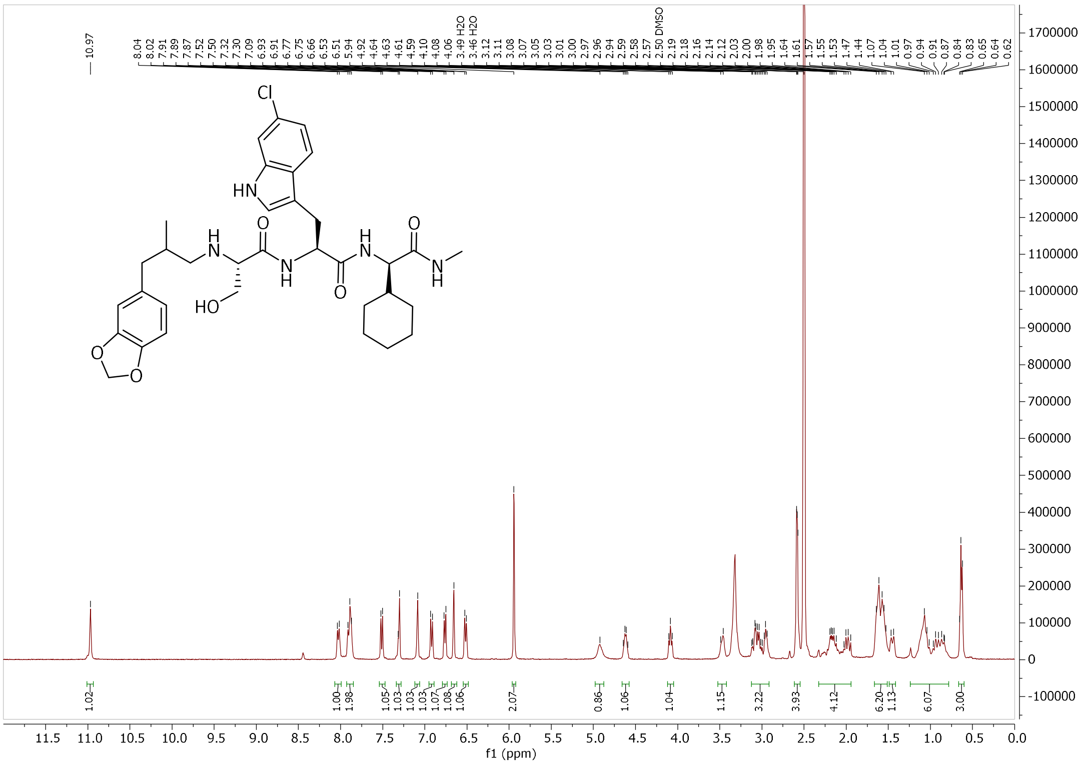

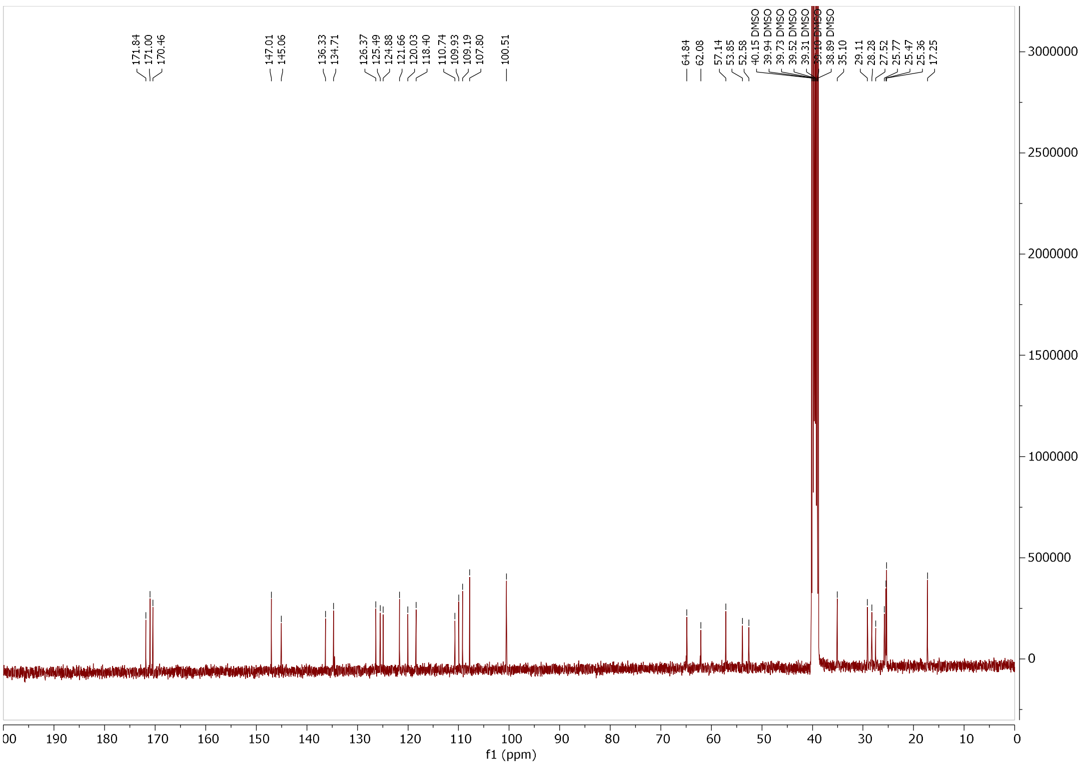

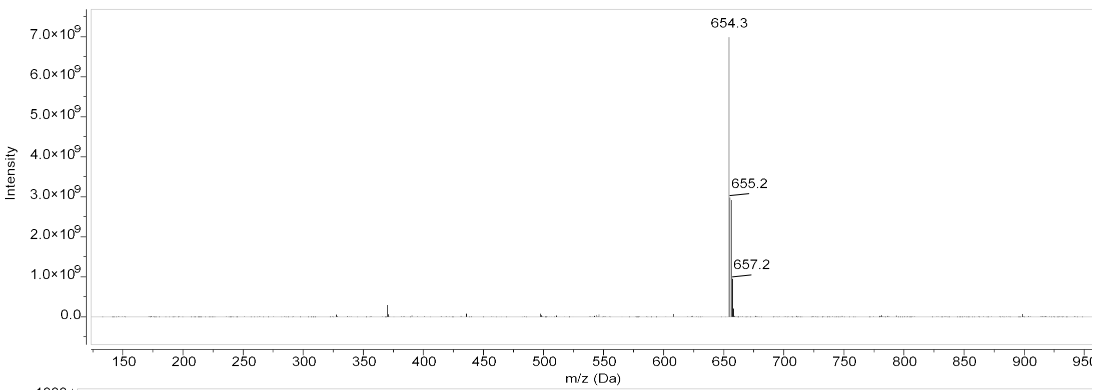
654.3
655.2
657.2
Intensity
7.0×10^9
6.0×10^9
5.0×10^9
4.0×10^9
3.0×10^9
2.0×10^9
1.0×10^9
0.0
150 200 250 300 350 400 450 500 550 600 650 700 750 800 850 900 950
m/z (Da)

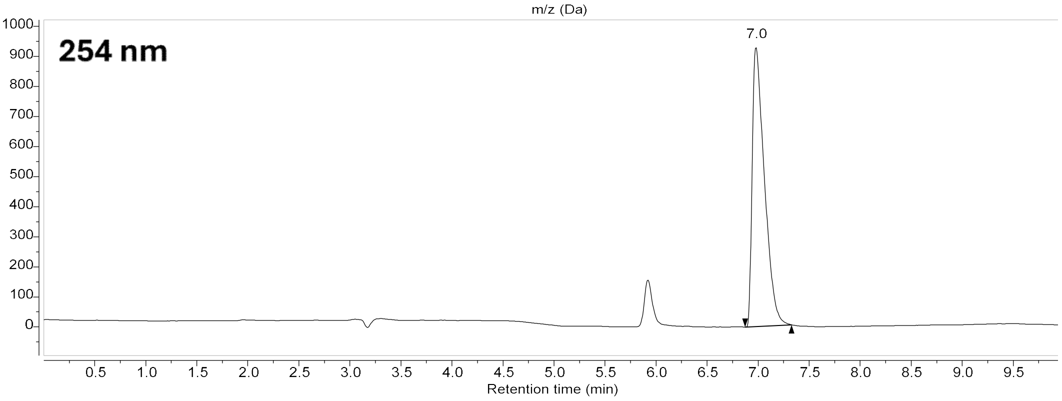
254 nm
7.0
1000 900 800 700 600 500 400 300 200 100 0
0.5 1.0 1.5 2.0 2.5 3.0 3.5 4.0 4.5 5.0 5.5 6.0 6.5 7.0 7.5 8.0 8.5 9.0 9.5
Retention time (min)

Compound 5:

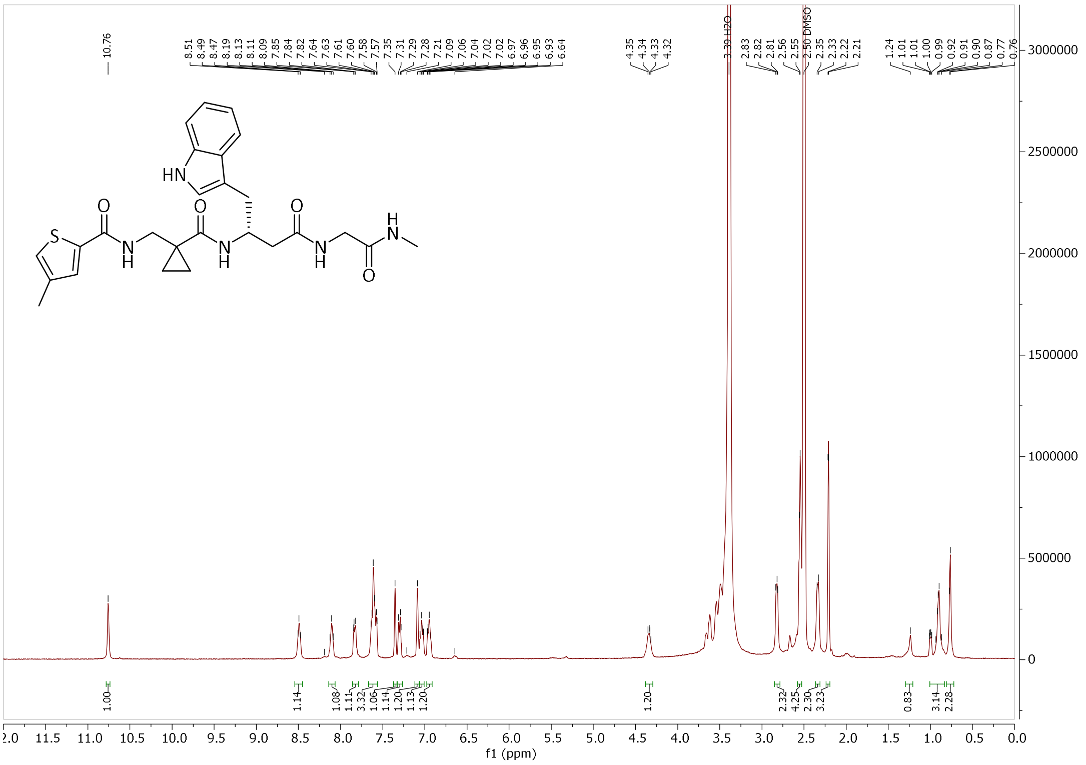
10.76
8.51 8.49 8.47 8.19 8.13 8.11 8.09 7.85 7.84 7.82 7.64 7.63 7.61 7.60 7.58 7.57 7.35 7.31 7.29 7.28 7.21 7.09 7.06 7.04 7.02 6.97 6.96 6.95 6.93 6.64
4.35 4.34 4.33 4.32
3.39 H2O
2.83 2.82 2.81 2.56 2.55 2.50 DMSO 2.35 2.33 2.22 2.21
1.24 1.01 1.01 1.00 0.99 0.92 0.91 0.90 0.87 0.77 0.76
HN
S
O
N
H
f1 (ppm)

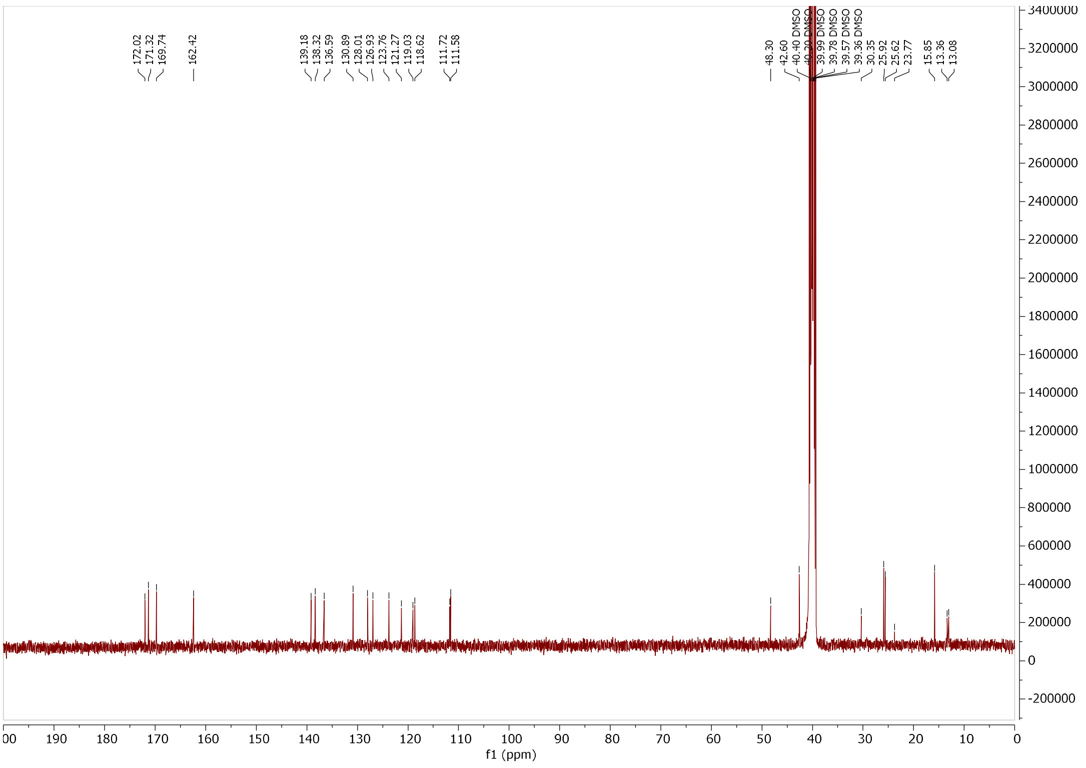
172.02 171.32 169.74 162.42
139.18 138.32 136.59 130.89 128.01 126.93 123.76 121.27 119.03 118.62 111.72 111.58
48.30 42.60 40.40 DMSO 40.20 DMSO 39.99 DMSO 39.78 DMSO 39.57 DMSO 39.36 DMSO 30.35 25.92 25.62 23.77 15.85 13.36 13.08
f1 (ppm)

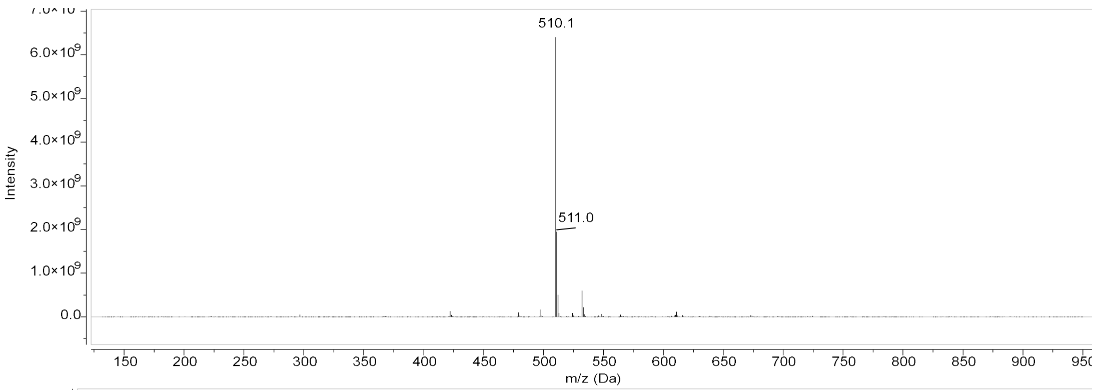
510.1
511.0
Intensity
m/z (Da)

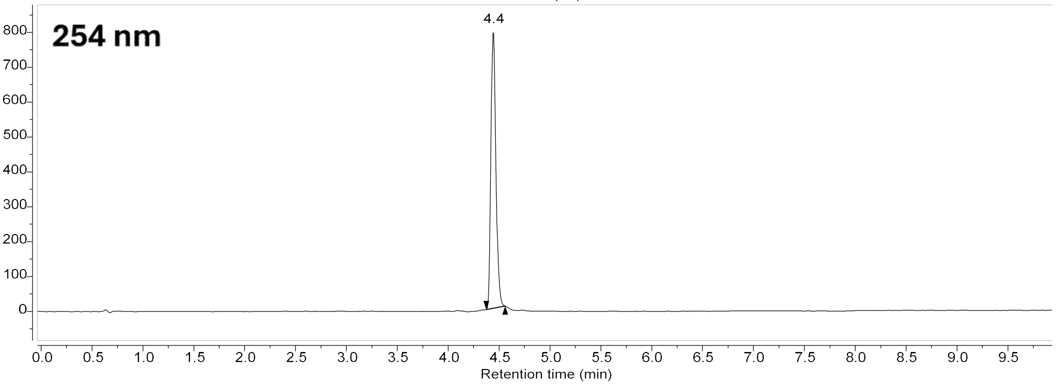
254 nm
4.4
Retention time (min)

Compound 10:

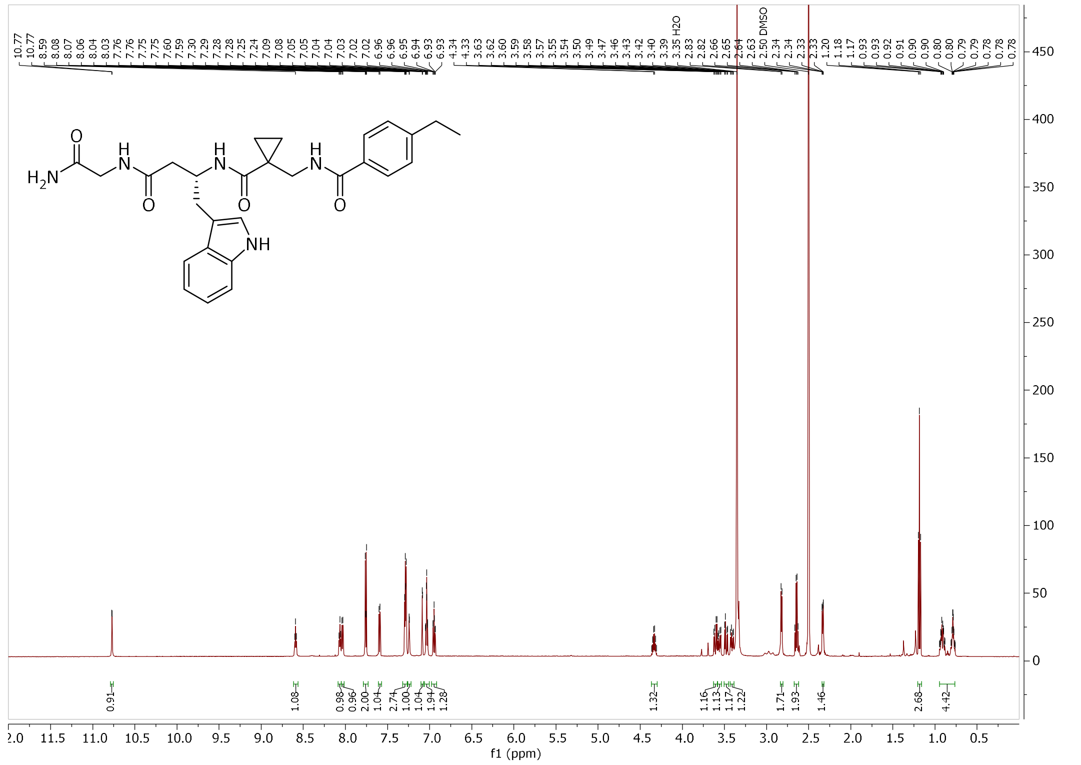

10.77 10.77 8.59 8.08 8.07 8.06 8.04 8.03 7.76 7.76 7.75 7.75 7.60 7.59 7.30 7.29 7.28 7.28 7.25 7.24 7.09 7.08 7.05 7.05 7.04 7.04 7.03 7.02 7.02 6.96 6.96 6.95 6.94 6.93 6.93 4.34 4.33 3.63 3.62 3.60 3.59 3.58 3.57 3.55 3.54 3.50 3.49 3.47 3.46 3.43 3.42 3.40 3.39 3.35 H2O 2.83 2.82 2.66 2.65 2.64 2.63 2.50 DMSO 2.34 2.34 2.33 2.33 1.20 1.18 1.17 0.93 0.93 0.92 0.91 0.90 0.90 0.80 0.80 0.79 0.79 0.78 0.78 0.78
0.91 1.08 0.98 0.96 2.00 1.04 2.74 1.00 1.04 1.94 1.28 1.32 1.16 1.13 1.17 1.22 1.71 1.93 1.46 2.68 4.42
f1 (ppm)

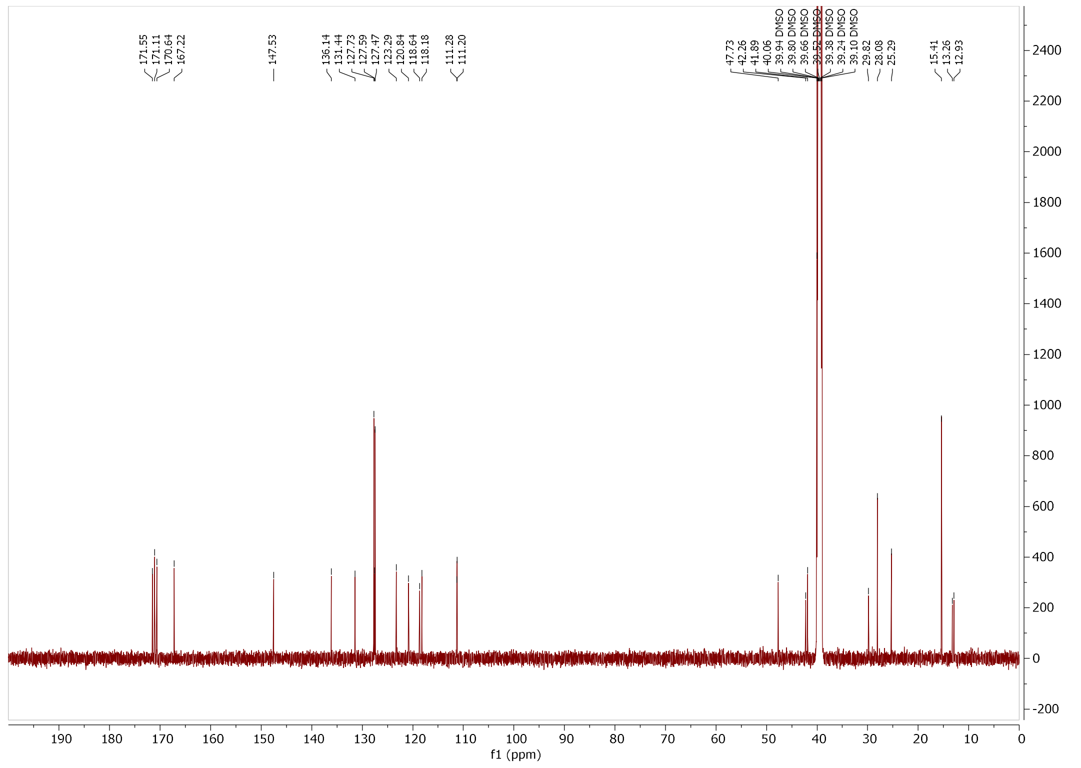

171.55 171.11 170.64 167.22 147.53 136.14 131.44 127.73 127.59 127.47 123.29 120.84 118.64 118.18 111.28 111.20 47.73 42.26 41.89 40.06 39.94 DMSO 39.80 DMSO 39.66 DMSO 39.52 DMSO 39.38 DMSO 39.24 DMSO 39.10 DMSO 29.82 28.08 25.29 15.41 13.26 12.93
f1 (ppm)

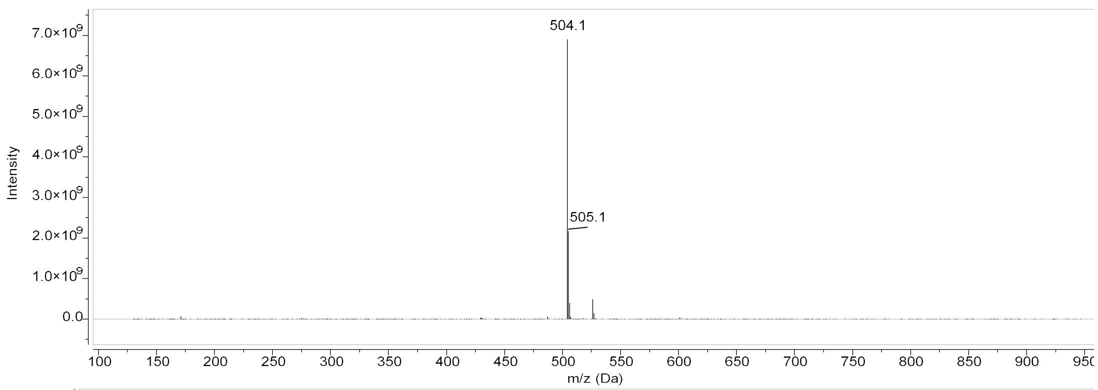
504.1
505.1
Intensity
7.0×10^9
6.0×10^9
5.0×10^9
4.0×10^9
3.0×10^9
2.0×10^9
1.0×10^9
0.0
100 150 200 250 300 350 400 450 500 550 600 650 700 750 800 850 900 950
m/z (Da)

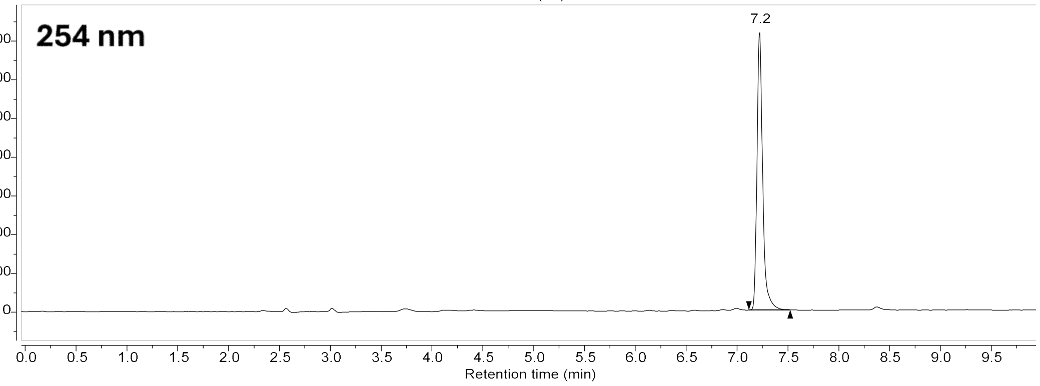
254 nm
7.2
700
600
500
400
300
200
100
0
0.0 0.5 1.0 1.5 2.0 2.5 3.0 3.5 4.0 4.5 5.0 5.5 6.0 6.5 7.0 7.5 8.0 8.5 9.0 9.5
Retention time (min)

Compound 18:

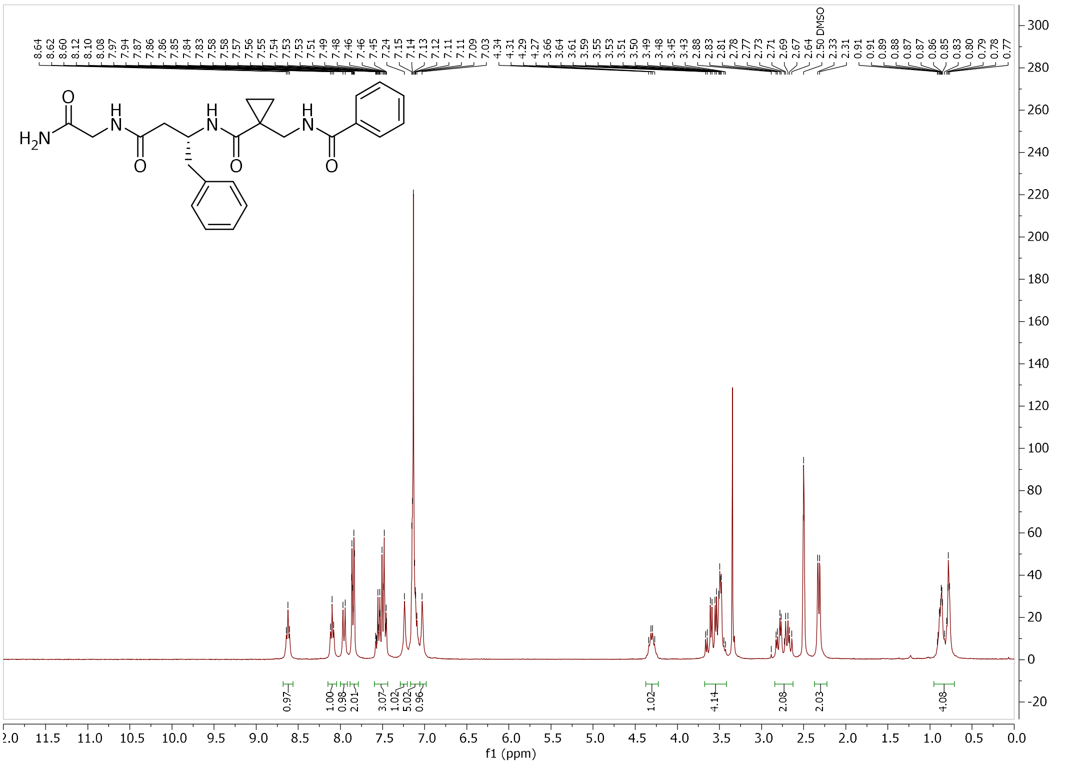

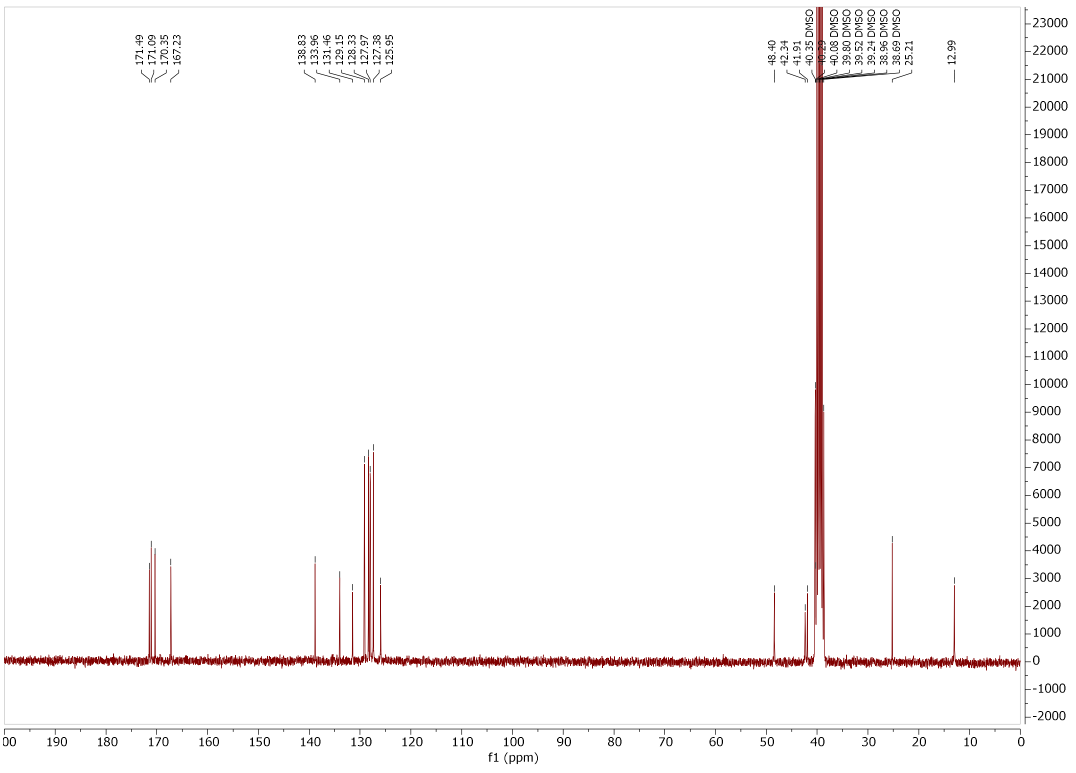

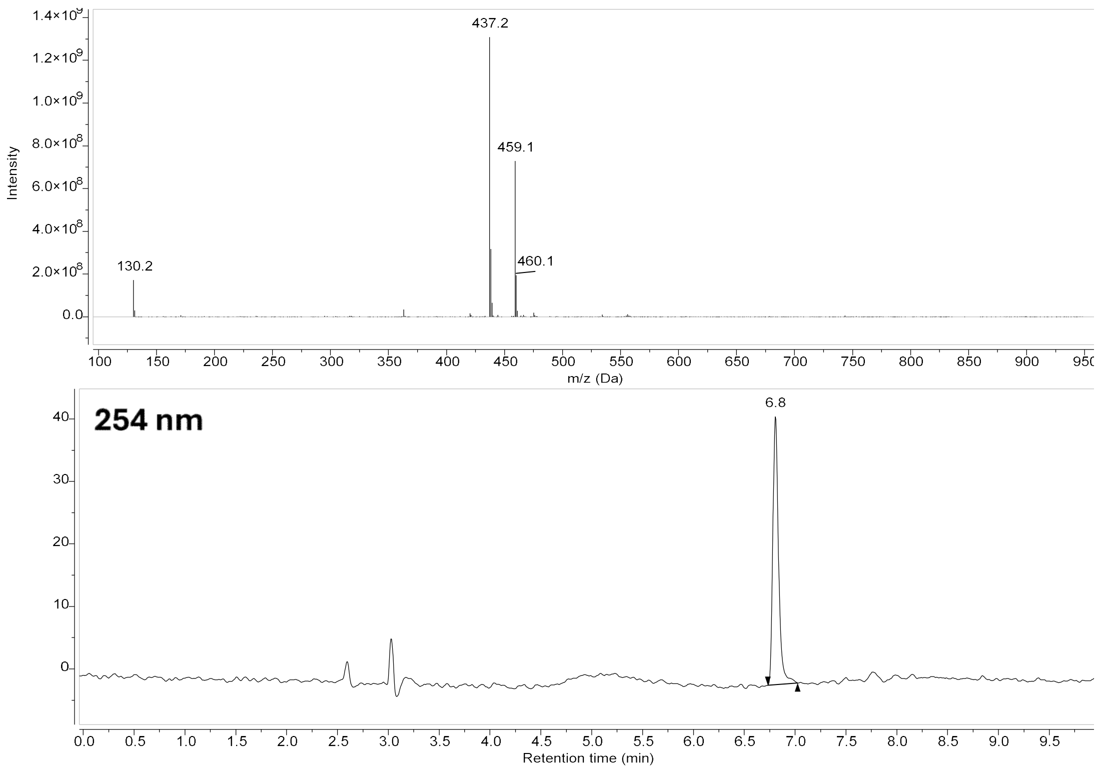

Intensity
437.2
459.1
460.1
130.2
m/z (Da)
254 nm
6.8
Retention time (min)

Compound 11:

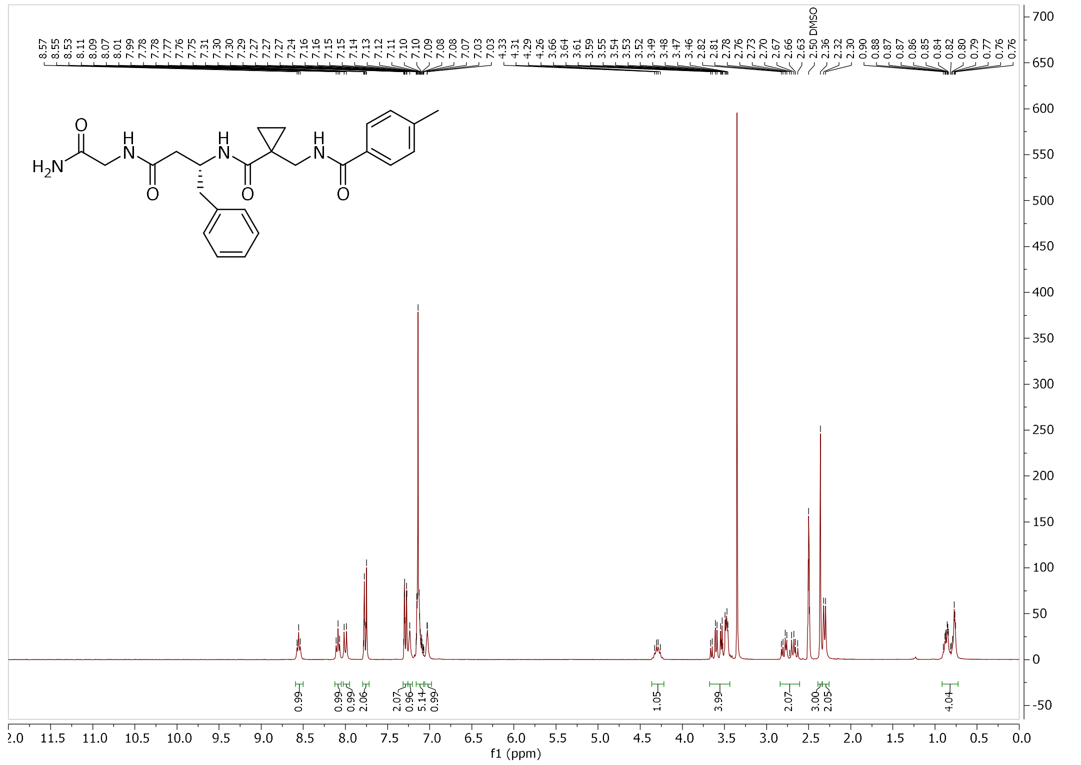

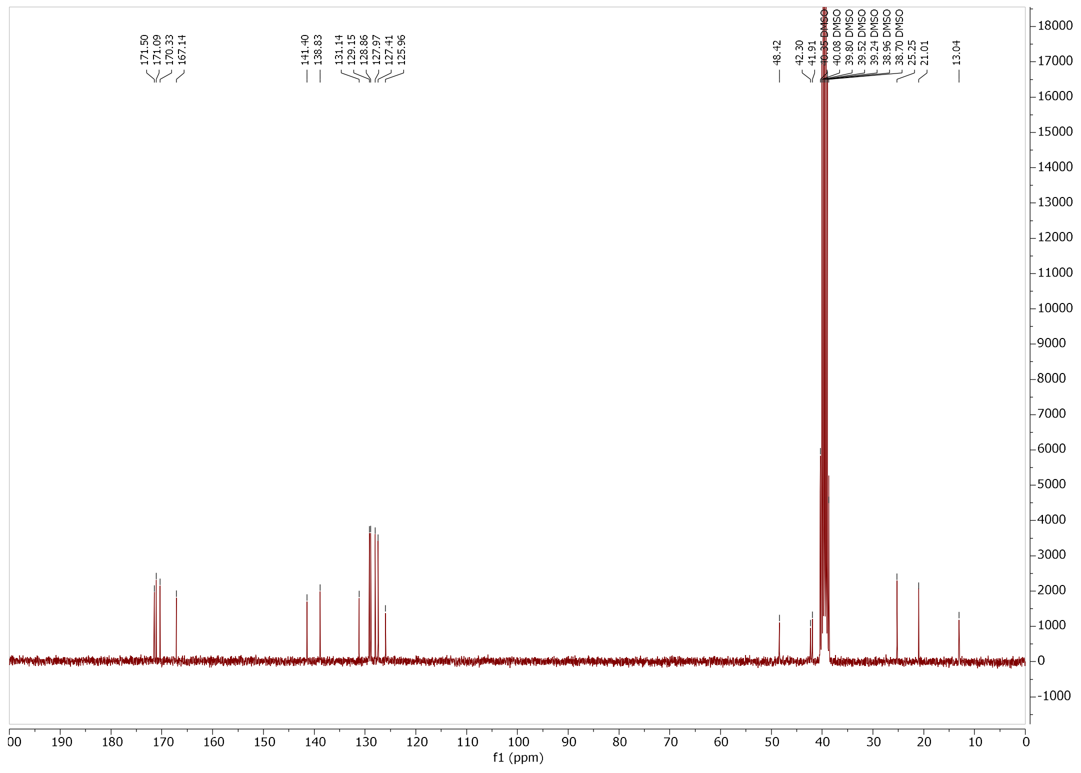

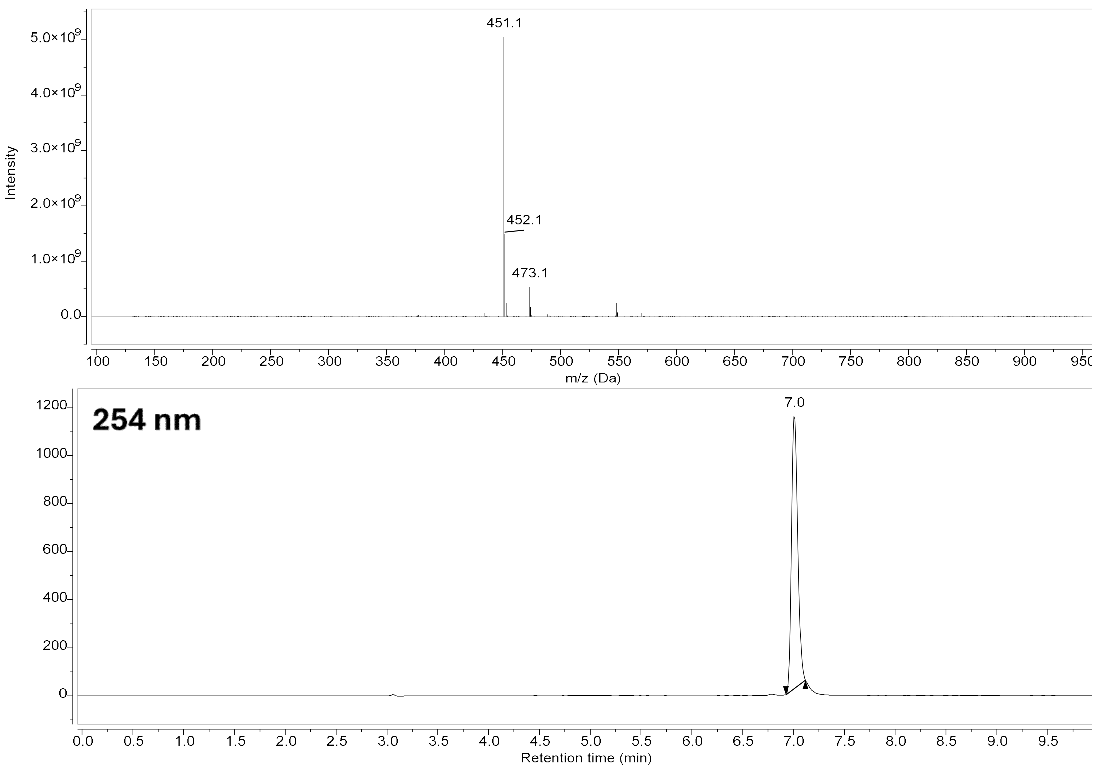
451.1
452.1
473.1
Intensity
5.0×10^9
4.0×10^9
3.0×10^9
2.0×10^9
1.0×10^9
0.0
m/z (Da)
254 nm
7.0
Retention time (min)

Compound 12:

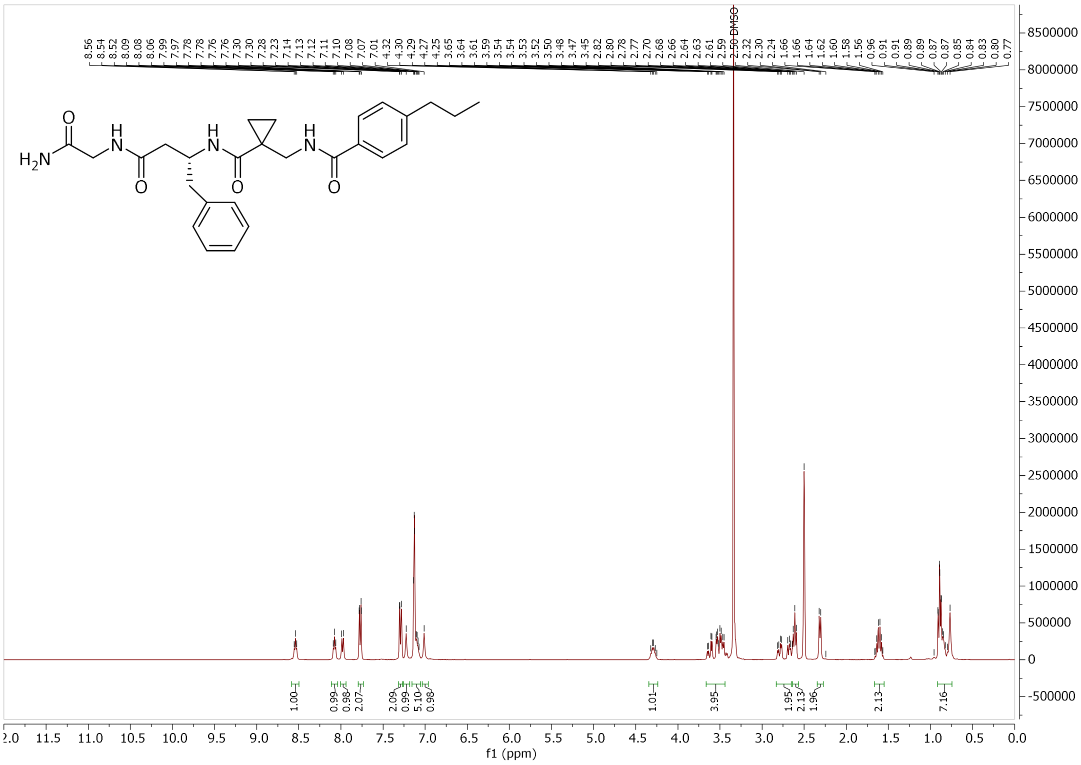

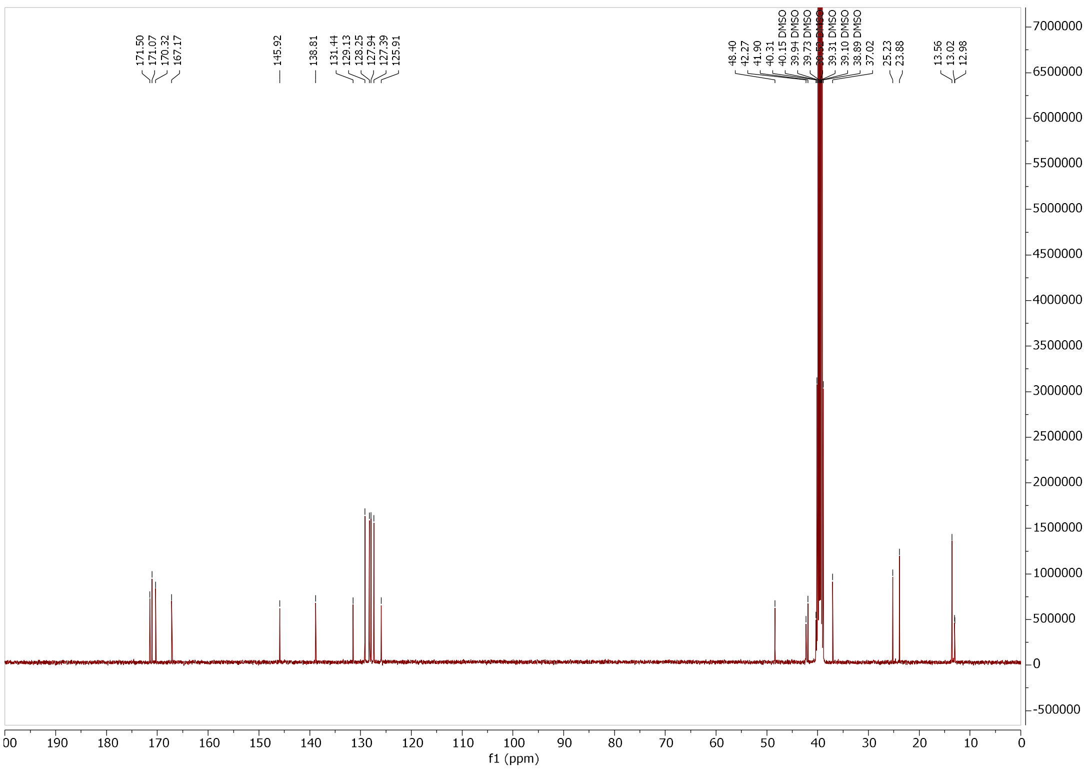

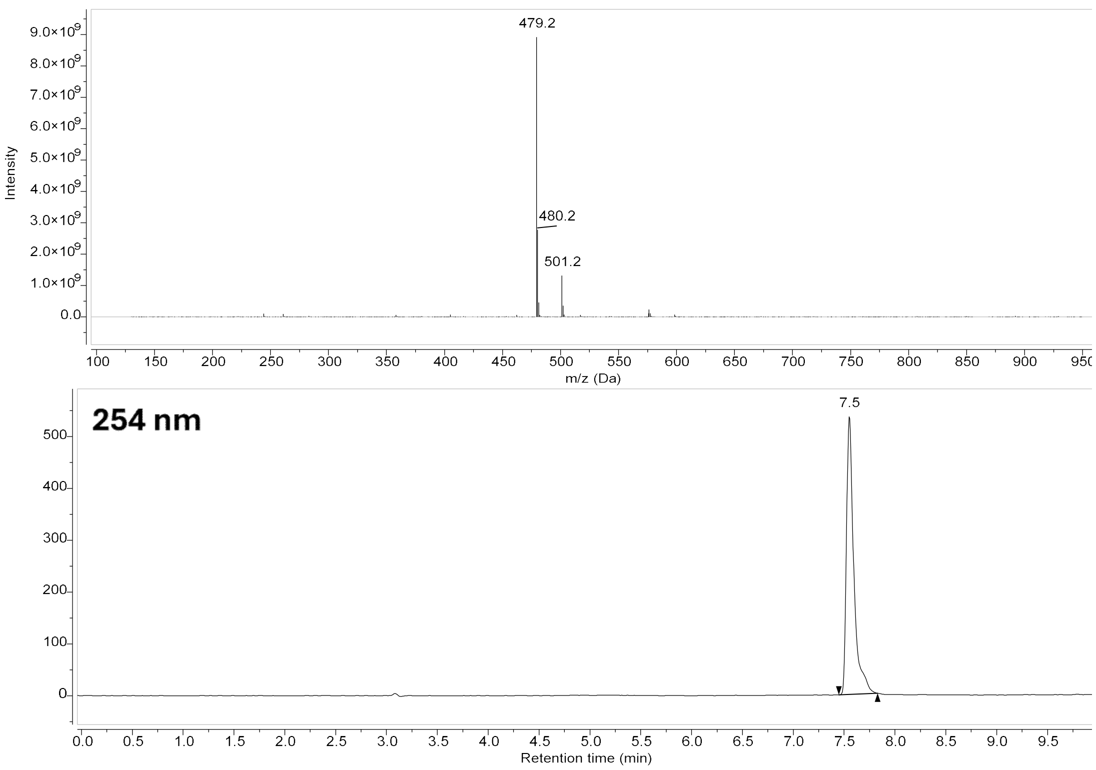

479.2
480.2
501.2
Intensity
m/z (Da)
254 nm
7.5
Retention time (min)

Compound 13:

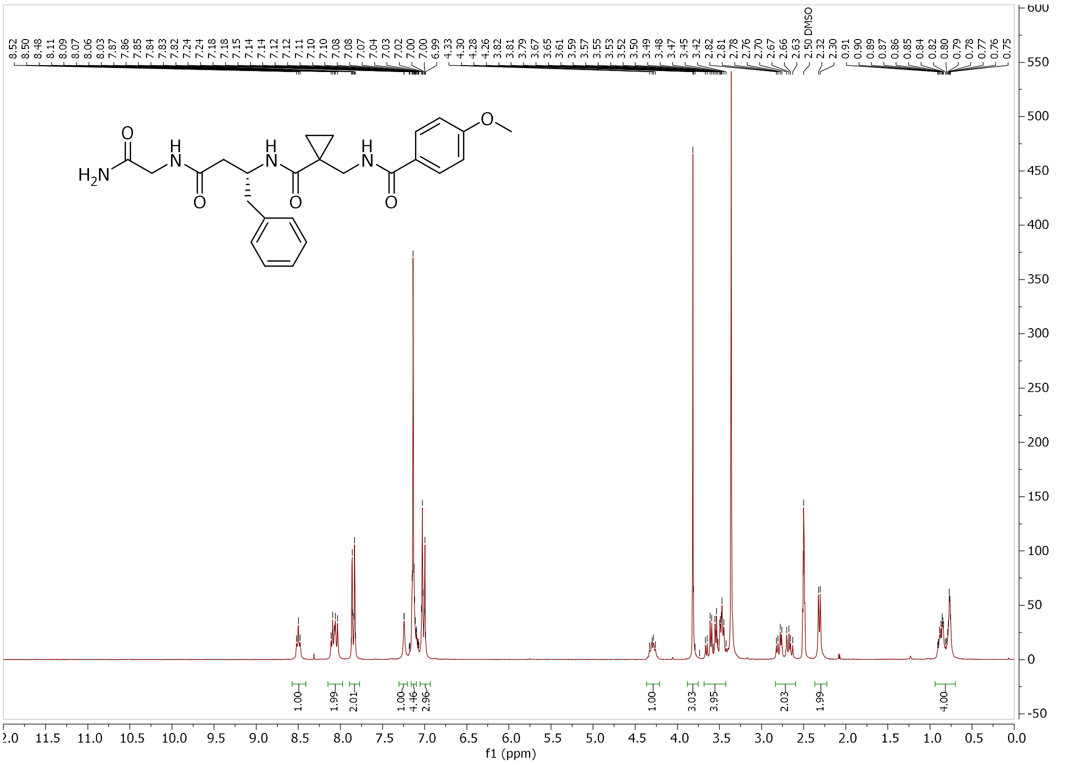

8.52 8.50 8.48 8.11 8.09 8.07 8.06 8.03 7.87 7.86 7.85 7.84 7.83 7.82 7.24 7.24 7.18 7.18 7.15 7.14 7.14 7.12 7.12 7.11 7.10 7.10 7.08 7.08 7.07 7.04 7.03 7.02 7.00 7.00 6.99
4.33 4.30 4.28 4.26 3.82 3.81 3.79 3.67 3.65 3.61 3.59 3.57 3.55 3.53 3.52 3.50 3.49 3.48 3.47 3.45 3.42 2.82 2.81 2.78 2.76 2.70 2.67 2.66 2.63 2.50 DMSO 2.32 2.30
0.91 0.90 0.89 0.87 0.86 0.85 0.84 0.82 0.80 0.79 0.78 0.77 0.76 0.75
1.00 1.99 2.01 1.00 4.46 2.96 1.00 3.03 3.95 2.03 1.99 4.00
f1 (ppm)

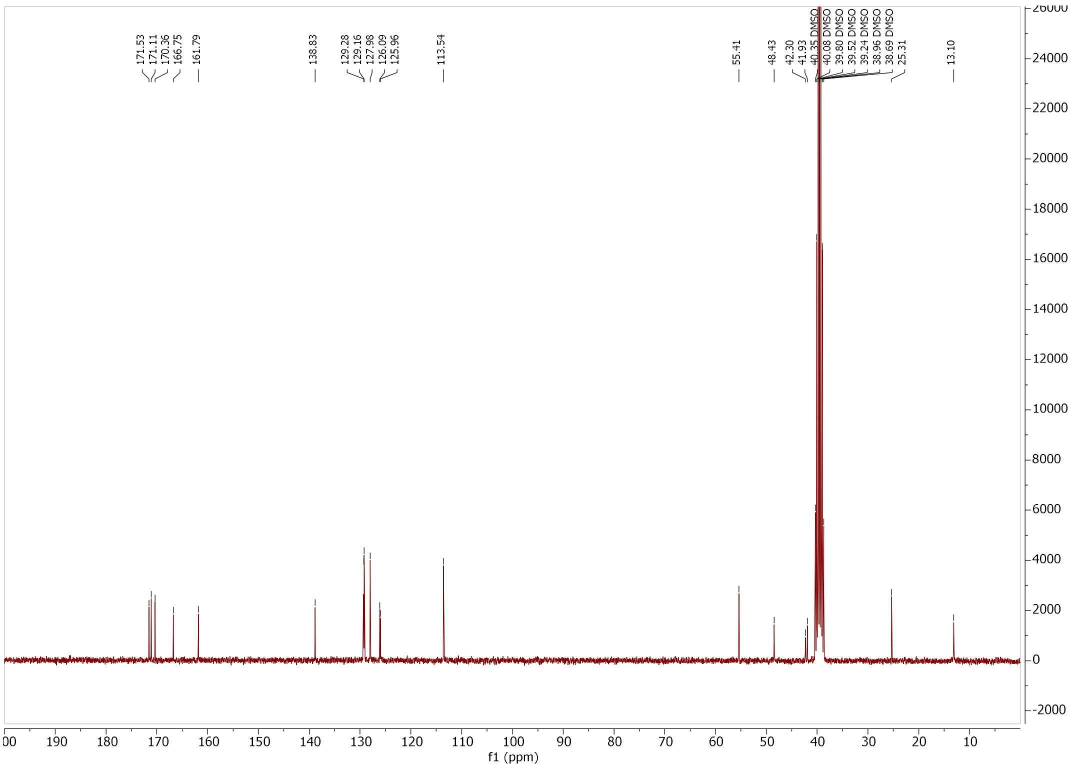

171.53 171.11 170.36 166.75 161.79 138.83 129.28 129.16 127.98 126.09 125.96 113.54 55.41 48.43 42.30 41.93 40.35 DMSO 40.08 DMSO 39.80 DMSO 39.52 DMSO 39.24 DMSO 38.96 DMSO 38.69 DMSO 25.31 13.10
f1 (ppm)

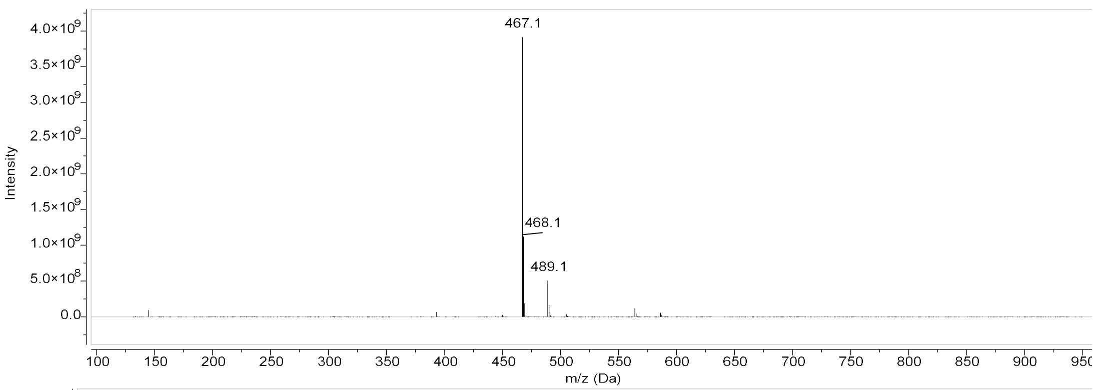
467.1
468.1
489.1
Intensity
m/z (Da)

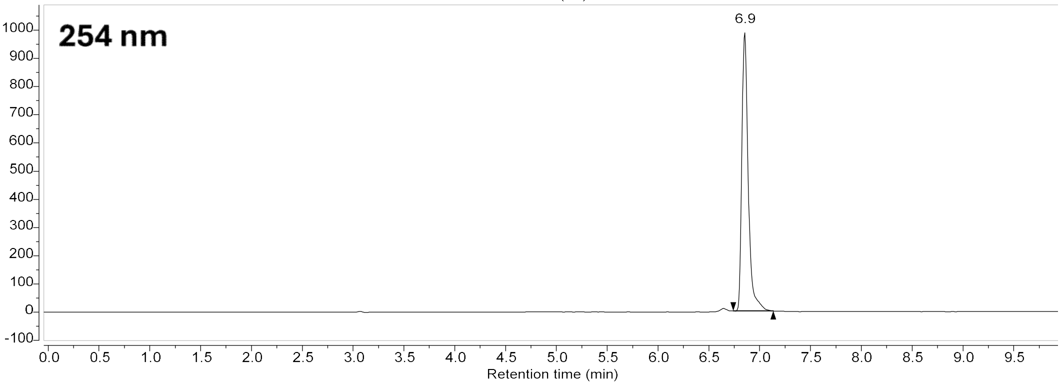
254 nm
6.9
Retention time (min)

Compound 19:

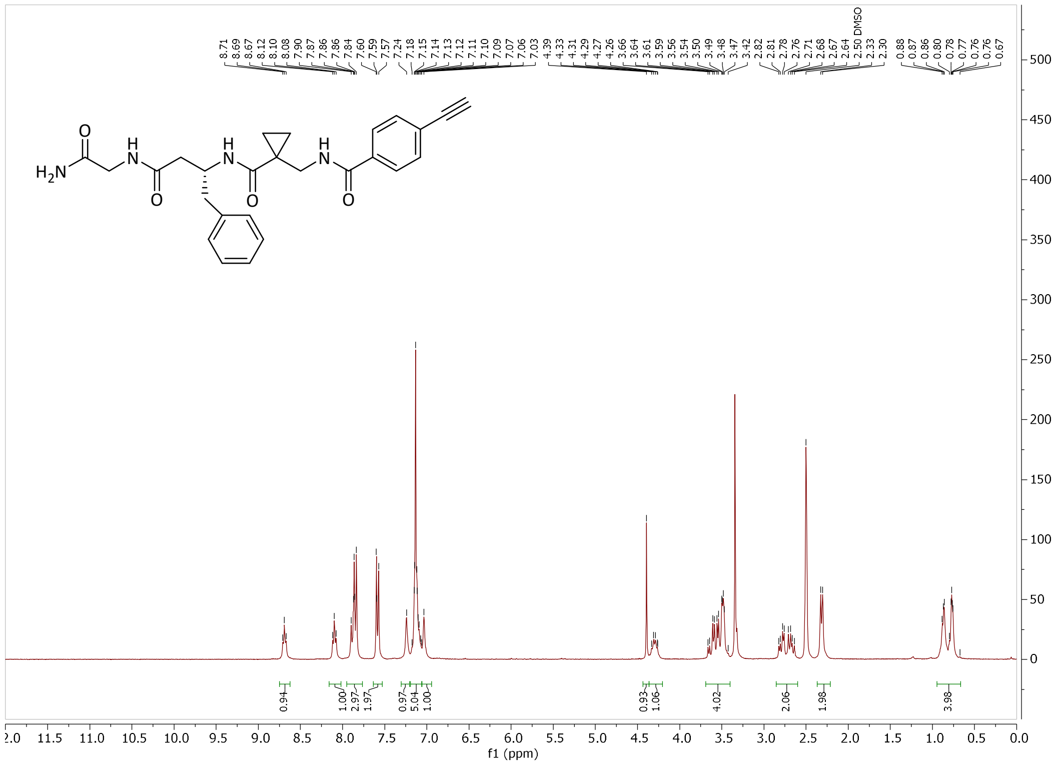

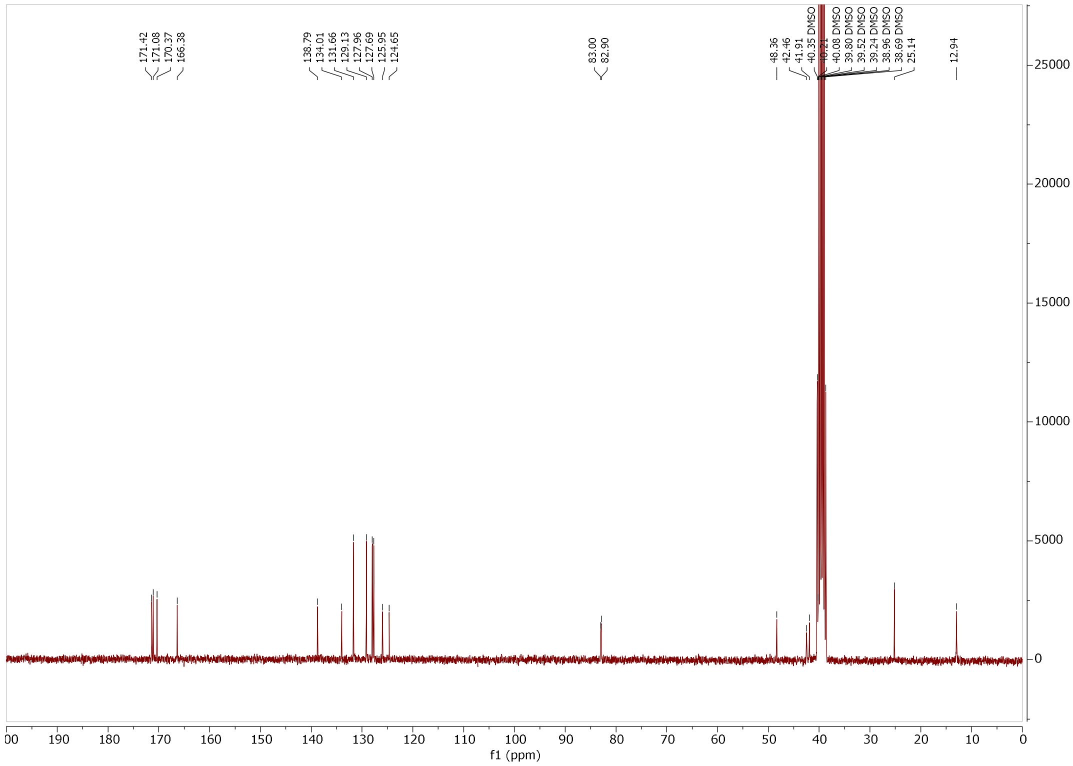

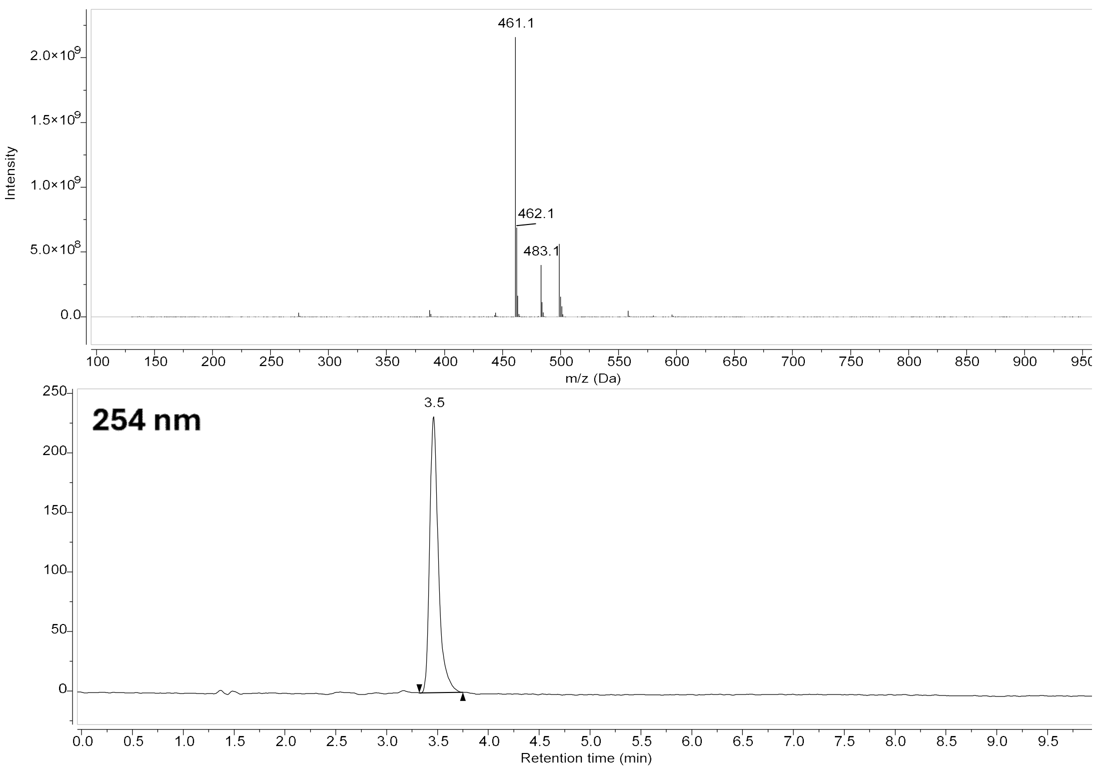
461.1
462.1
483.1
Intensity
2.0×10^9
1.5×10^9
1.0×10^9
5.0×10^8
0.0
100 150 200 250 300 350 400 450 500 550 600 650 700 750 800 850 900 950
m/z (Da)
254 nm
3.5
250
200
150
100
50
0
0.0 0.5 1.0 1.5 2.0 2.5 3.0 3.5 4.0 4.5 5.0 5.5 6.0 6.5 7.0 7.5 8.0 8.5 9.0 9.5
Retention time (min)

Compound 20:

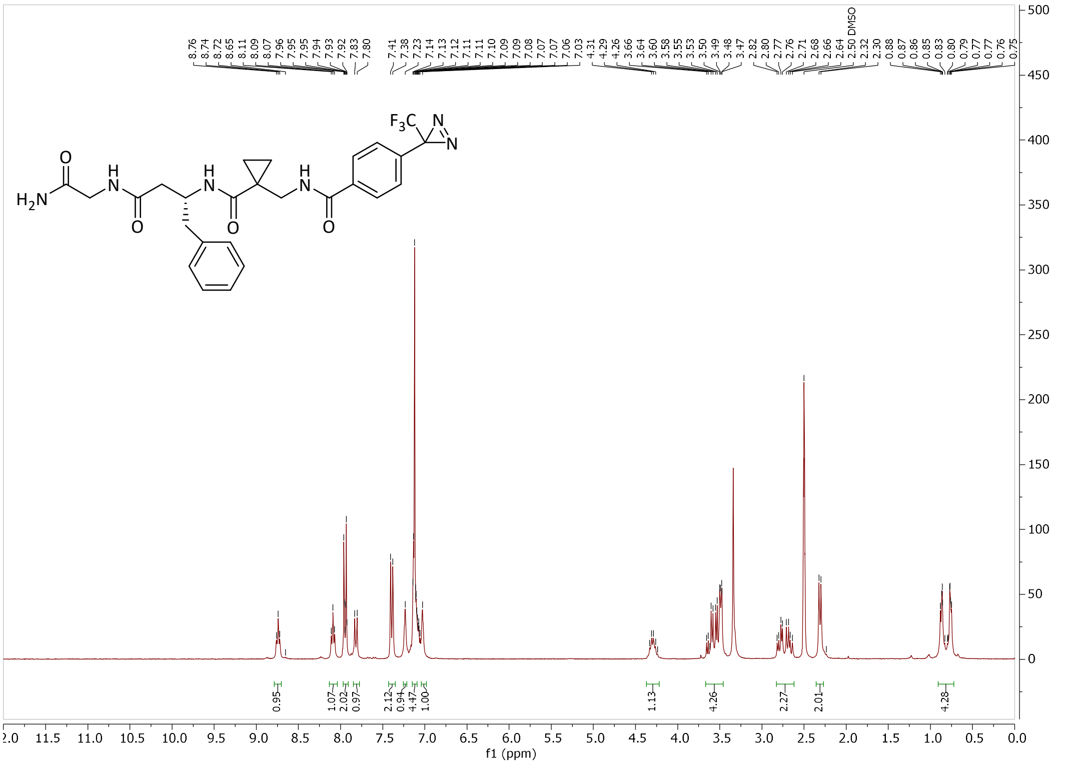

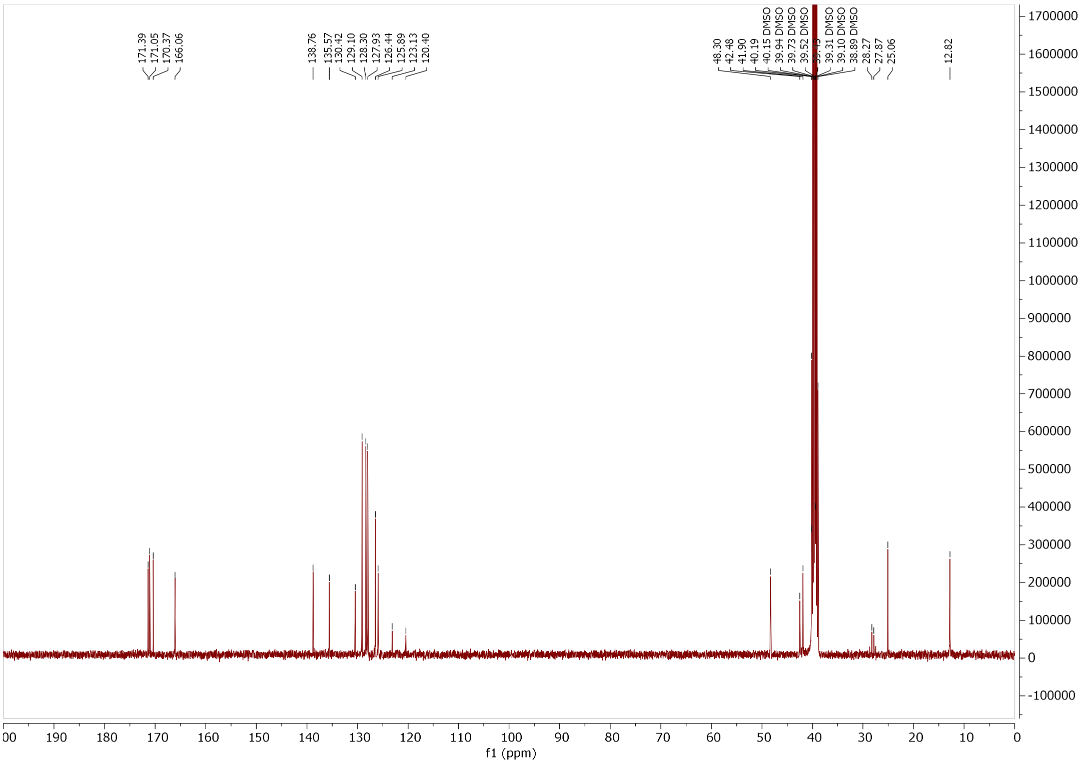

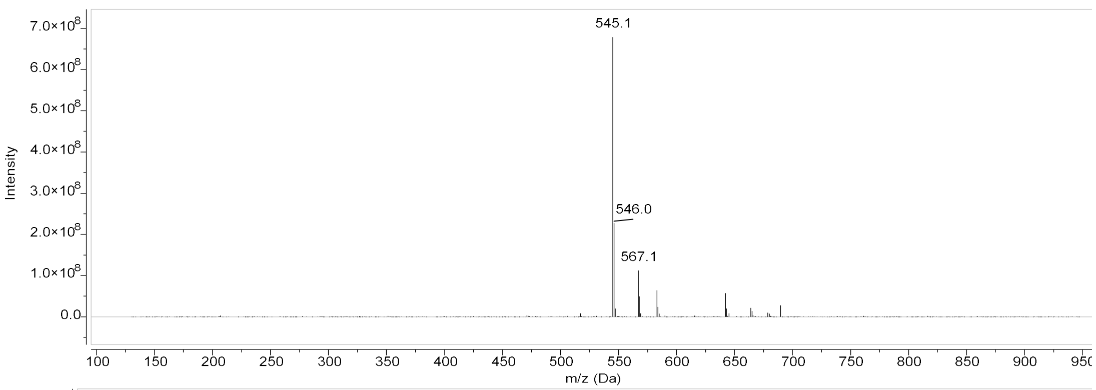

545.1
546.0
567.1
Intensity
m/z (Da)

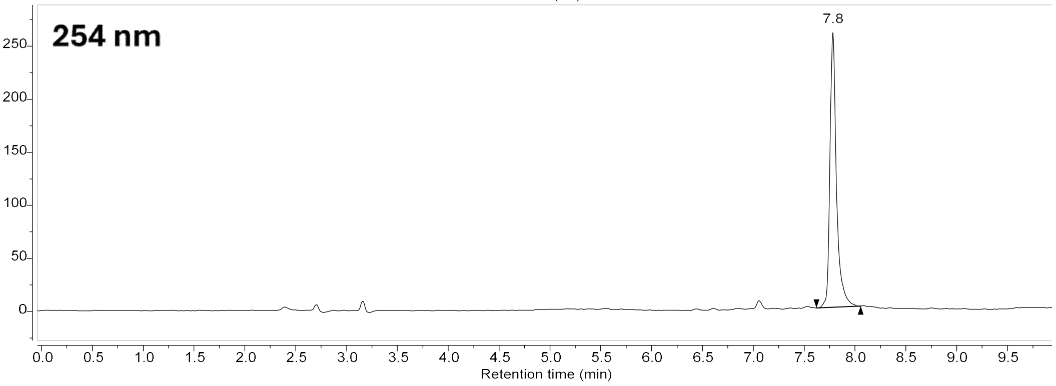

254 nm
7.8
Retention time (min)

Compound 21:

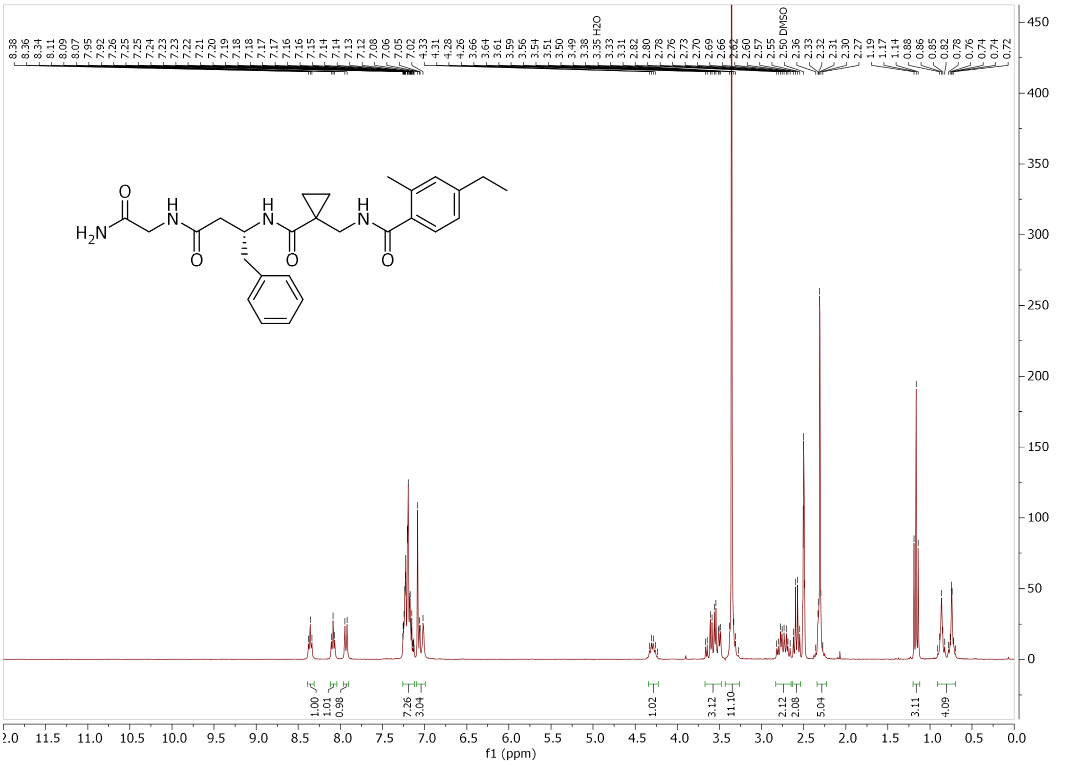
8.38 8.36 8.34 8.11 8.09 8.07 7.95 7.92 7.26 7.25 7.25 7.24 7.23 7.23 7.22 7.21 7.20 7.19 7.18 7.17 7.17 7.16 7.15 7.14 7.14 7.13 7.12 7.08 7.06 7.05 7.02 4.33 4.31 4.28 4.26 3.66 3.64 3.61 3.59 3.56 3.54 3.51 3.50 3.49 3.38 3.35 H2O 3.33 3.31 2.82 2.80 2.78 2.76 2.73 2.70 2.69 2.66 2.62 2.60 2.57 2.55 2.50 DMSO 2.36 2.33 2.32 2.31 2.30 2.27 1.19 1.17 1.14 0.88 0.86 0.85 0.82 0.78 0.76 0.74 0.72
O
H
N
H
N
H
N
H_2N
O
O
O
1.00
1.01
0.98
7.26
3.04
1.02
3.12
11.10
2.12
2.08
5.04
3.11
4.09
f1 (ppm)

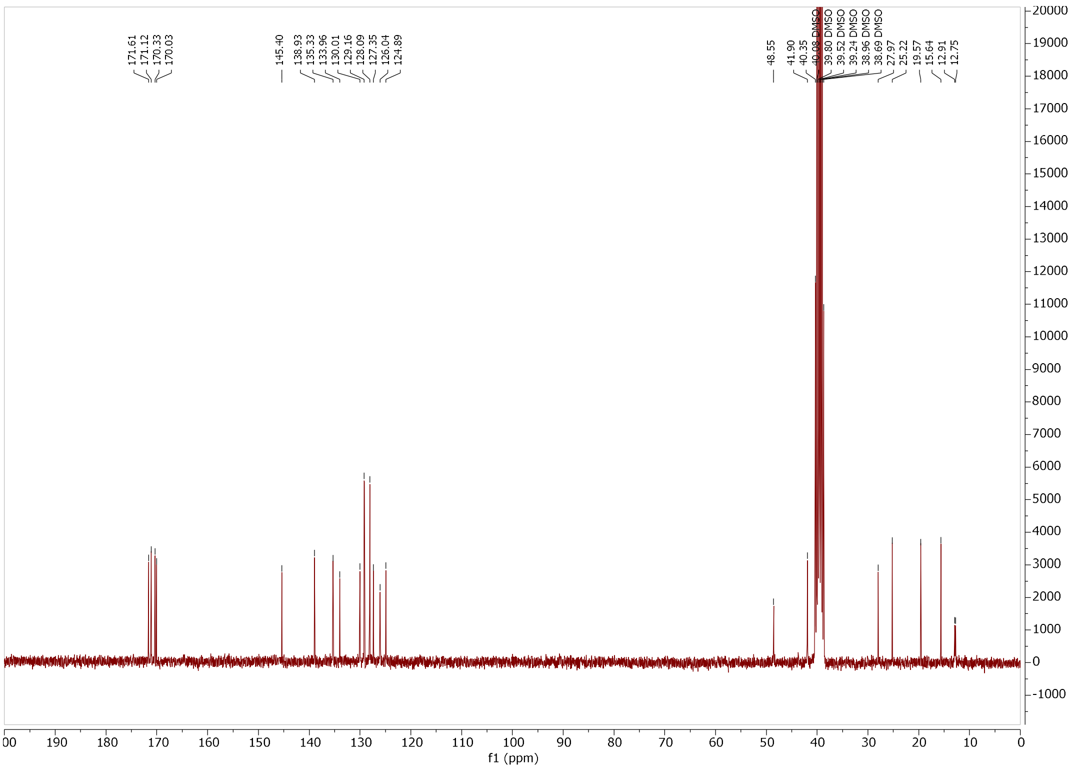
171.61 171.12 170.33 170.03
145.40
138.93 135.33 133.96 130.01 129.16 128.09 127.35 126.04 124.89
48.55
41.90 40.35 40.08 DMSO 39.80 DMSO 39.52 DMSO 39.24 DMSO 38.96 DMSO 38.69 DMSO 27.97 25.22 19.57 15.64 12.91 12.75
f1 (ppm)

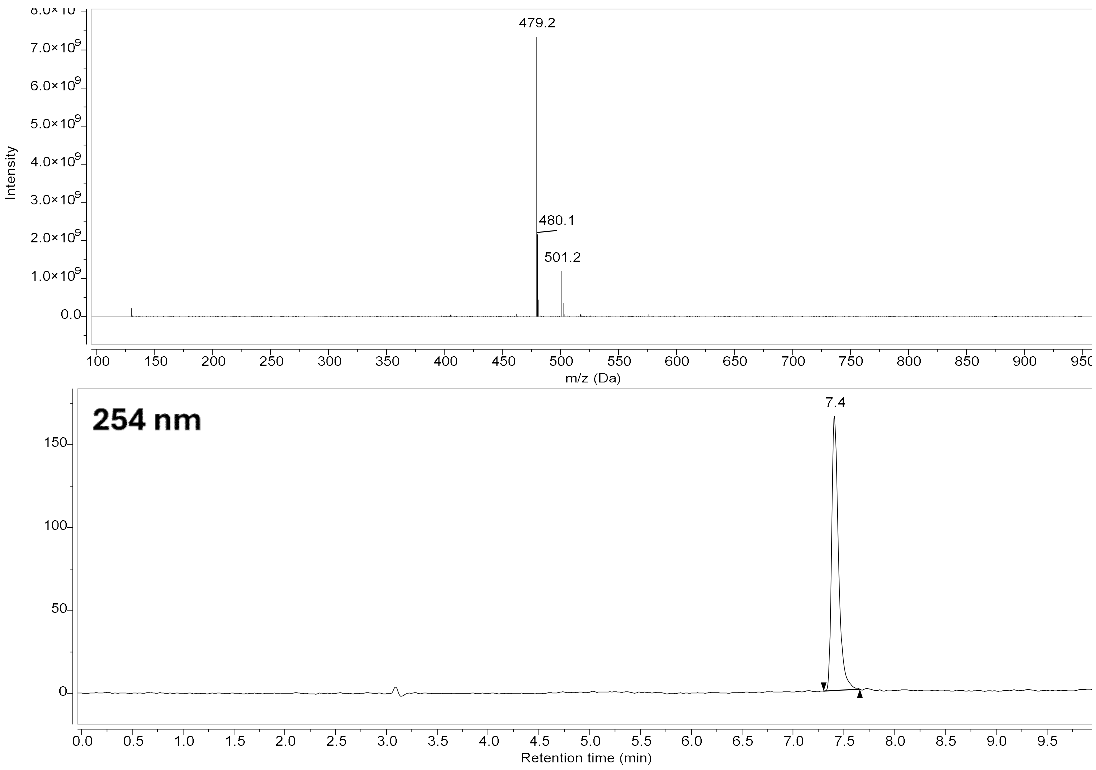

479.2
480.1
501.2
Intensity
m/z (Da)
254 nm
7.4
Retention time (min)

Compound 22:

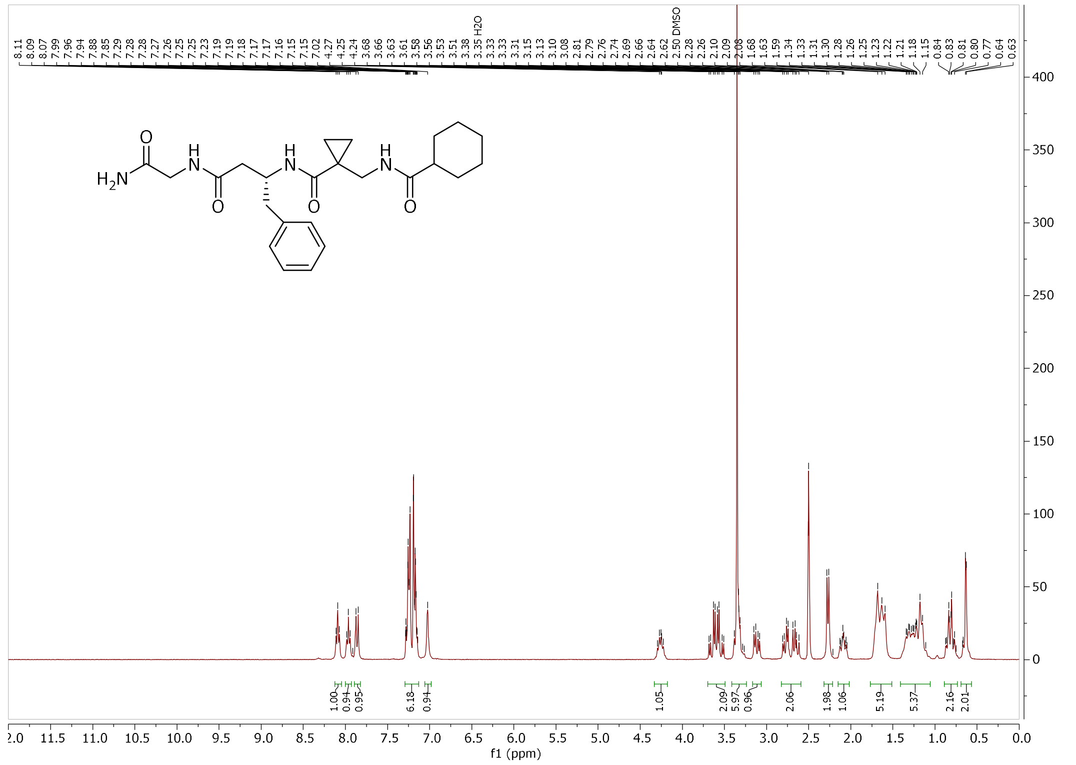

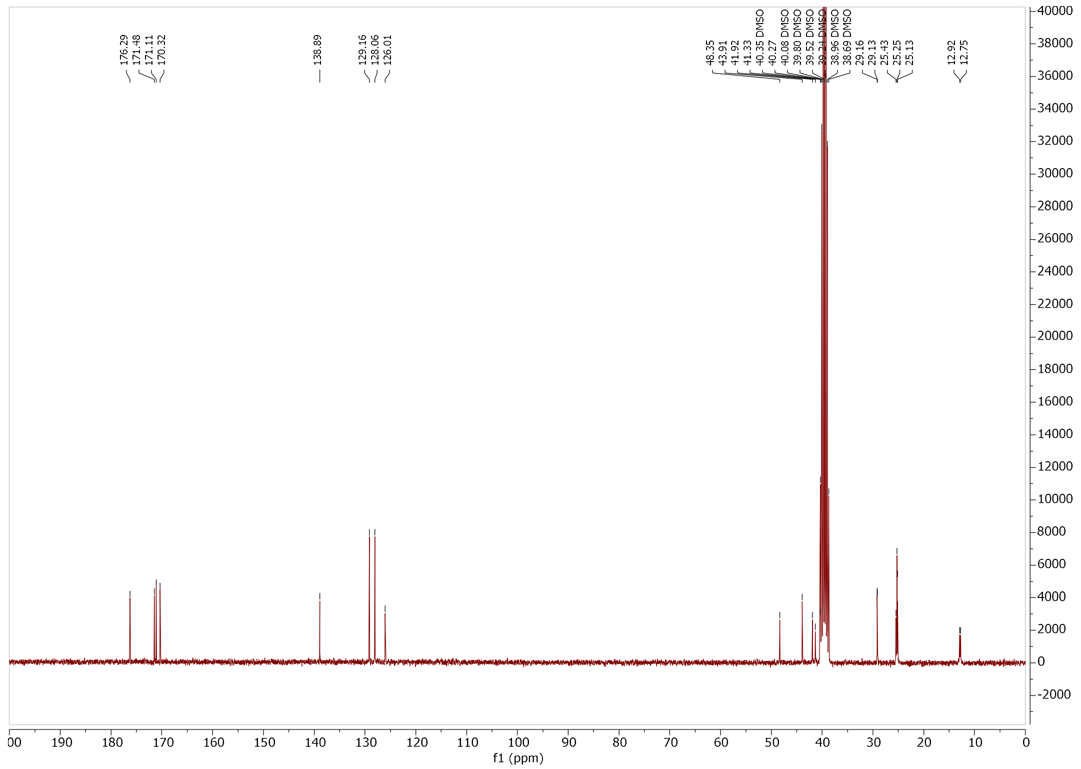

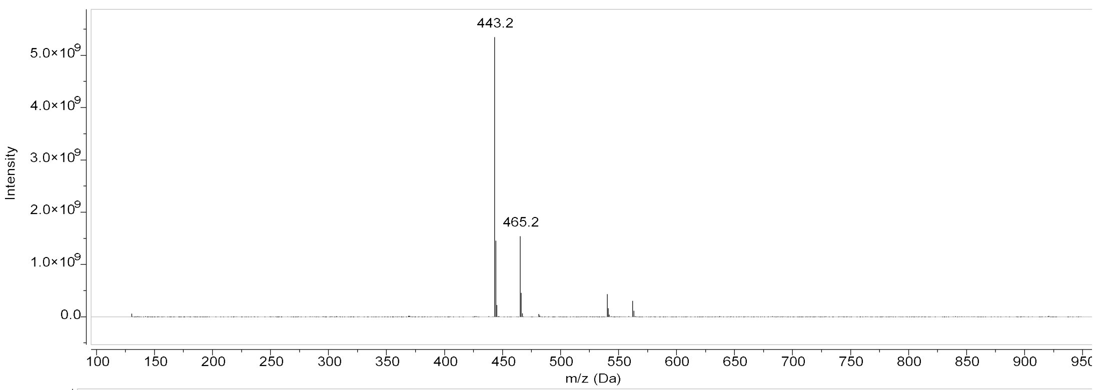
443.2
465.2
5.0×10^9
4.0×10^9
3.0×10^9
2.0×10^9
1.0×10^9
0.0
Intensity
100 150 200 250 300 350 400 450 500 550 600 650 700 750 800 850 900 950
m/z (Da)

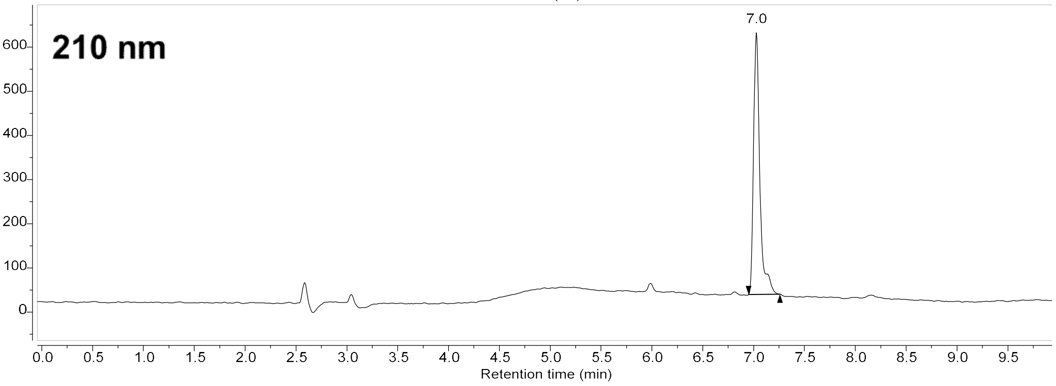
210 nm
7.0
600
500
400
300
200
100
0
0.0 0.5 1.0 1.5 2.0 2.5 3.0 3.5 4.0 4.5 5.0 5.5 6.0 6.5 7.0 7.5 8.0 8.5 9.0 9.5
Retention time (min)

Compound 23:

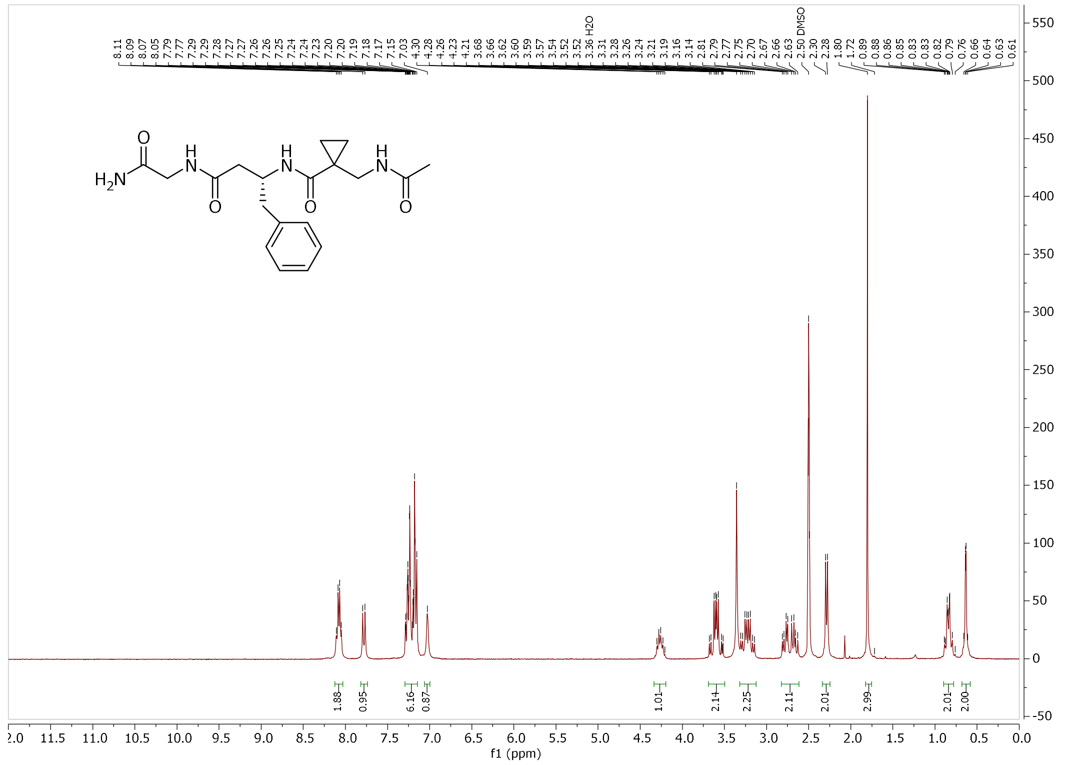

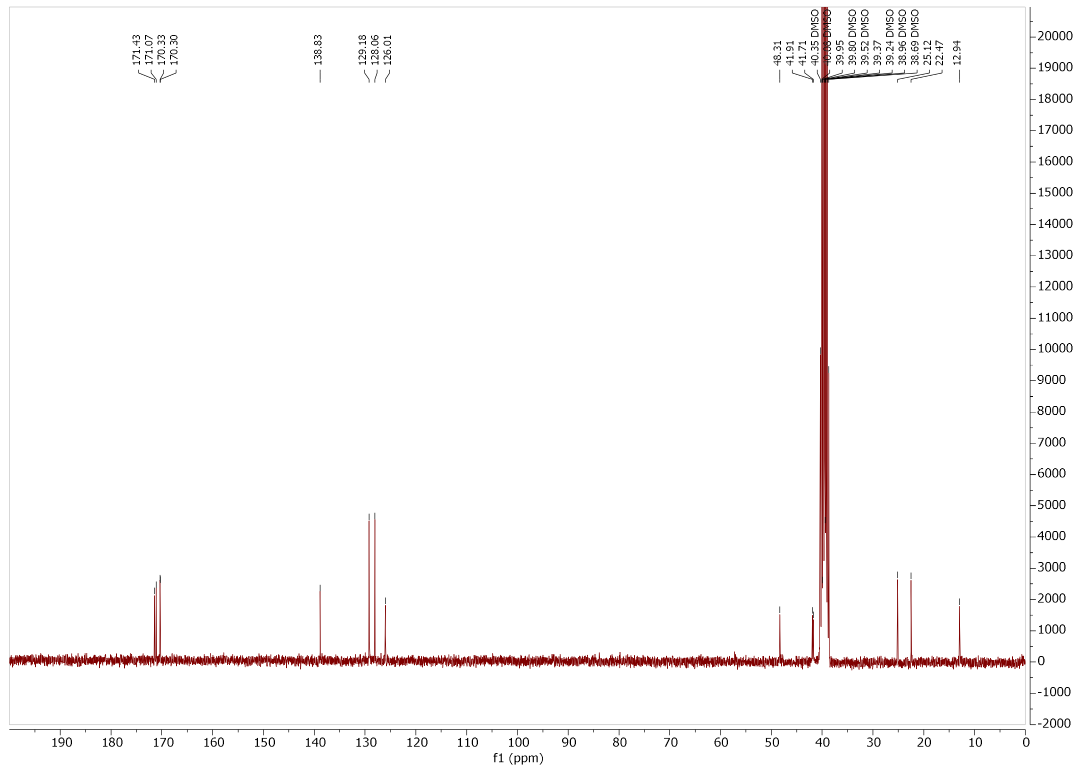

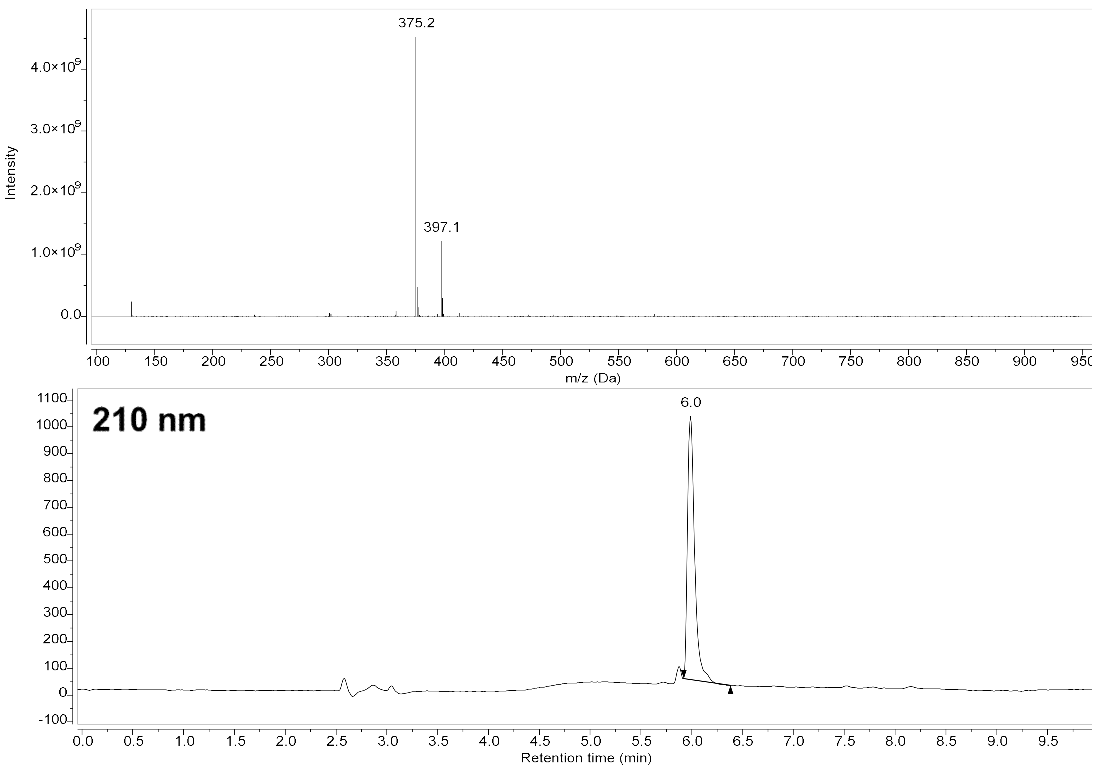

375.2
397.1
Intensity
4.0×10^9
3.0×10^9
2.0×10^9
1.0×10^9
0.0
m/z (Da)
210 nm
6.0
Retention time (min)

Compound 24:

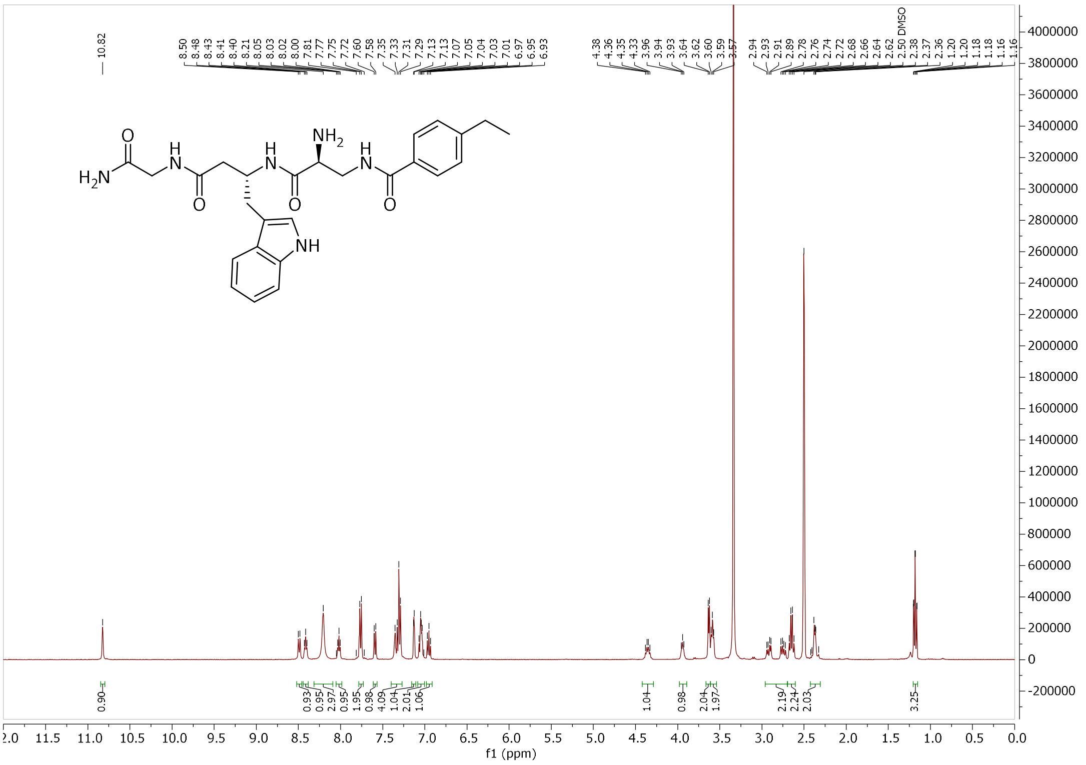

10.82
8.50 8.48 8.43 8.41 8.40 8.21 8.05 8.03 8.02 8.00 7.81 7.77 7.75 7.72 7.60 7.58 7.35 7.33 7.31 7.29 7.13 7.13 7.07 7.05 7.04 7.03 7.01 6.97 6.95 6.93
4.38 4.36 4.35 4.33 3.96 3.94 3.93 3.64 3.62 3.60 3.59 3.57
2.94 2.93 2.91 2.89 2.78 2.76 2.74 2.72 2.68 2.66 2.64 2.62 2.50 DMSO 2.38 2.37 2.36 1.20 1.20 1.18 1.18 1.16 1.16
0.90 0.93 0.95 2.97 0.95 1.95 0.98 4.09 1.04 2.01 1.06 1.04 0.98 2.04 1.97 2.19 2.24 2.03 3.25
f1 (ppm)

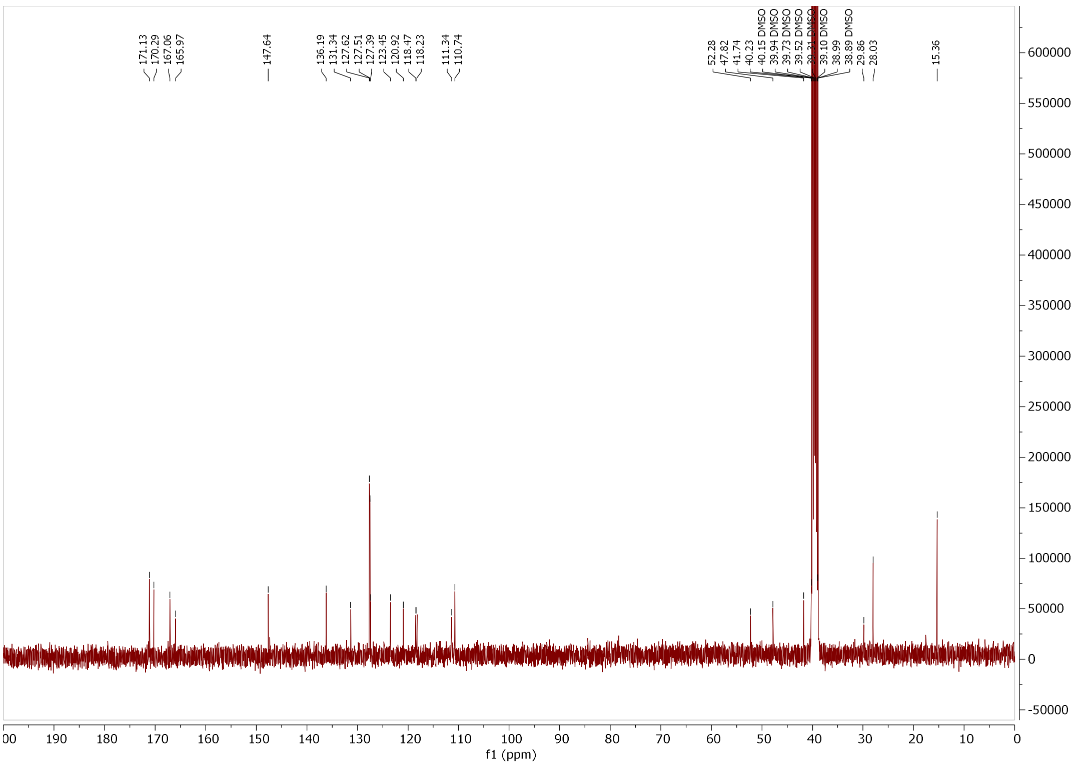

171.13 170.29 167.06 165.97
147.64
136.19 131.34 127.62 127.51 127.39 123.45 120.92 118.47 118.23
111.34 110.74
52.28 47.82 41.74 40.23 40.15 DMSO 39.94 DMSO 39.73 DMSO 39.52 DMSO 39.31 DMSO 39.10 DMSO 38.99 38.89 DMSO 29.86 28.03
15.36
f1 (ppm)

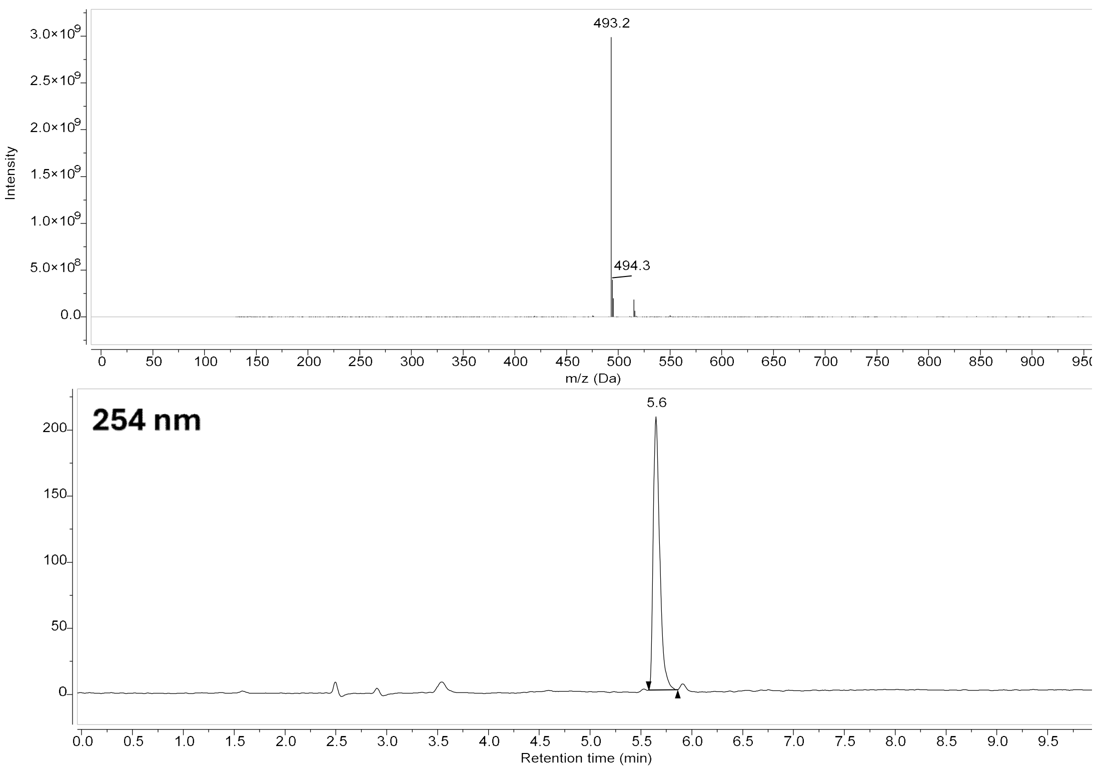

493.2
494.3
Intensity
m/z (Da)
254 nm
5.6
Retention time (min)

Compound 25:

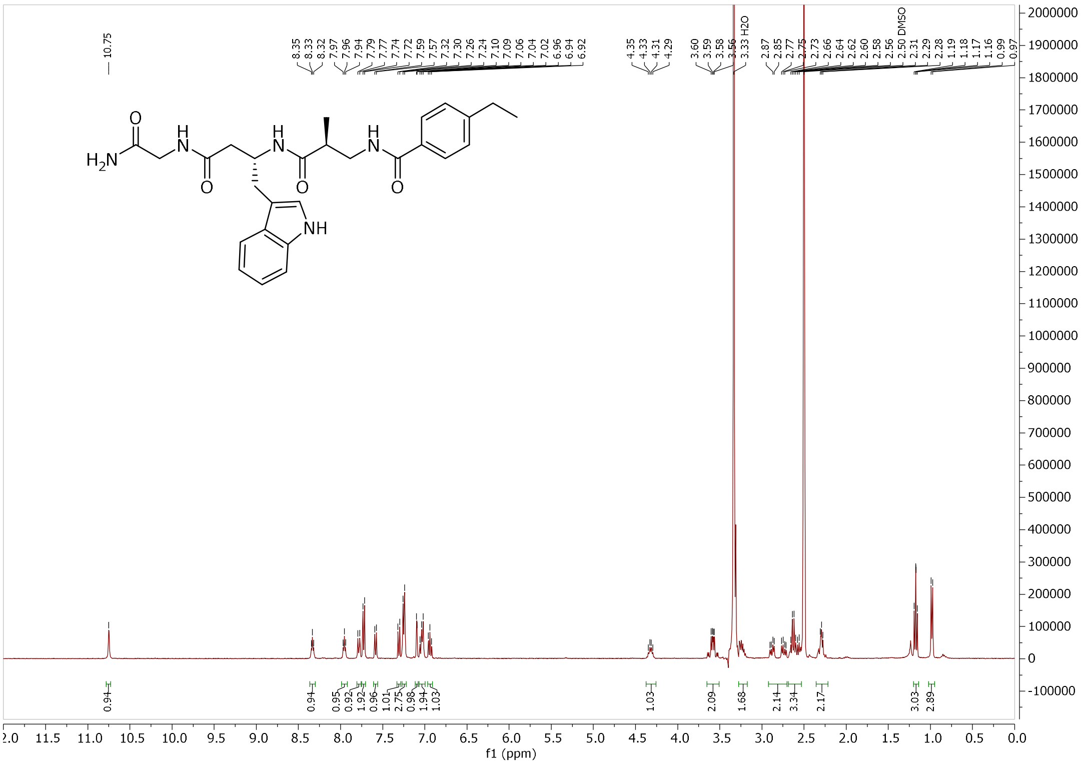
10.75
8.35
8.33
8.32
7.97
7.96
7.94
7.79
7.77
7.74
7.72
7.59
7.57
7.32
7.30
7.26
7.24
7.10
7.09
7.06
7.04
7.02
6.96
6.94
6.92
4.35
4.33
4.31
4.29
3.60
3.59
3.58
3.56
3.33 H2O
2.87
2.85
2.77
2.75
2.73
2.66
2.64
2.62
2.60
2.58
2.56
2.50 DMSO
2.31
2.29
2.28
1.19
1.18
1.17
1.16
0.99
0.97
H2N
NH
f1 (ppm)

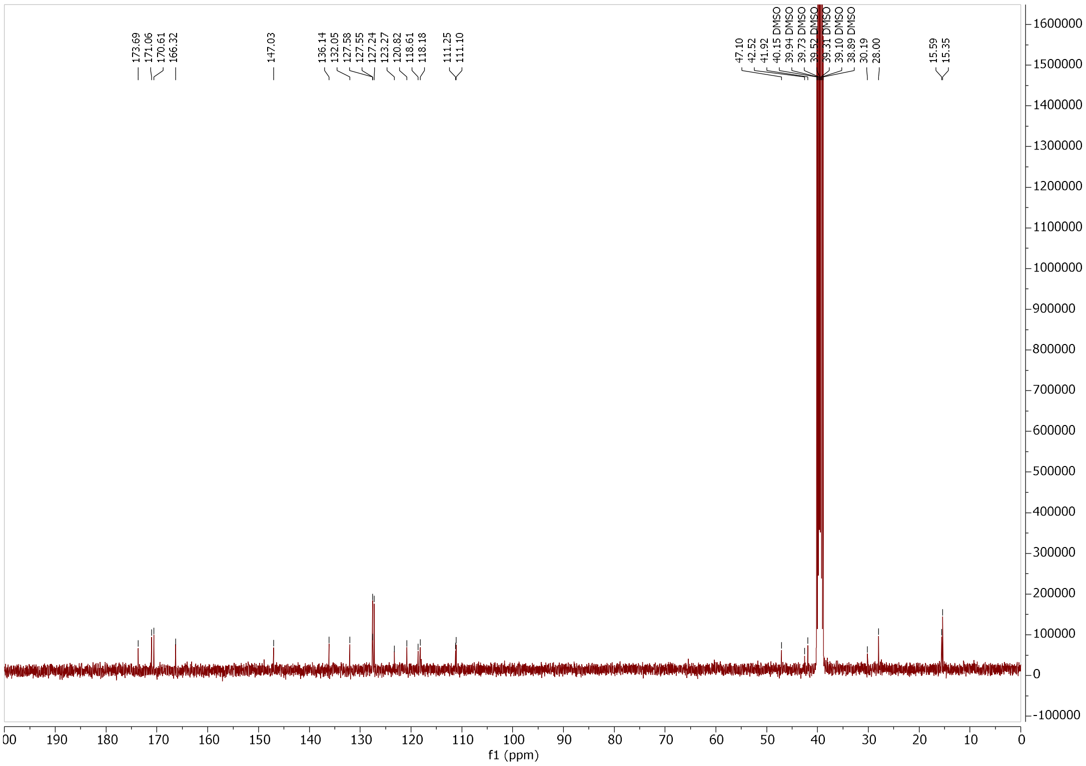
173.69
171.06
170.61
166.32
147.03
136.14
132.05
127.58
127.55
127.24
123.27
120.82
118.61
118.18
111.25
111.10
47.10
42.52
41.92
40.15 DMSO
39.94 DMSO
39.73 DMSO
39.52 DMSO
39.31 DMSO
39.10 DMSO
38.89 DMSO
30.19
28.00
15.59
15.35
f1 (ppm)

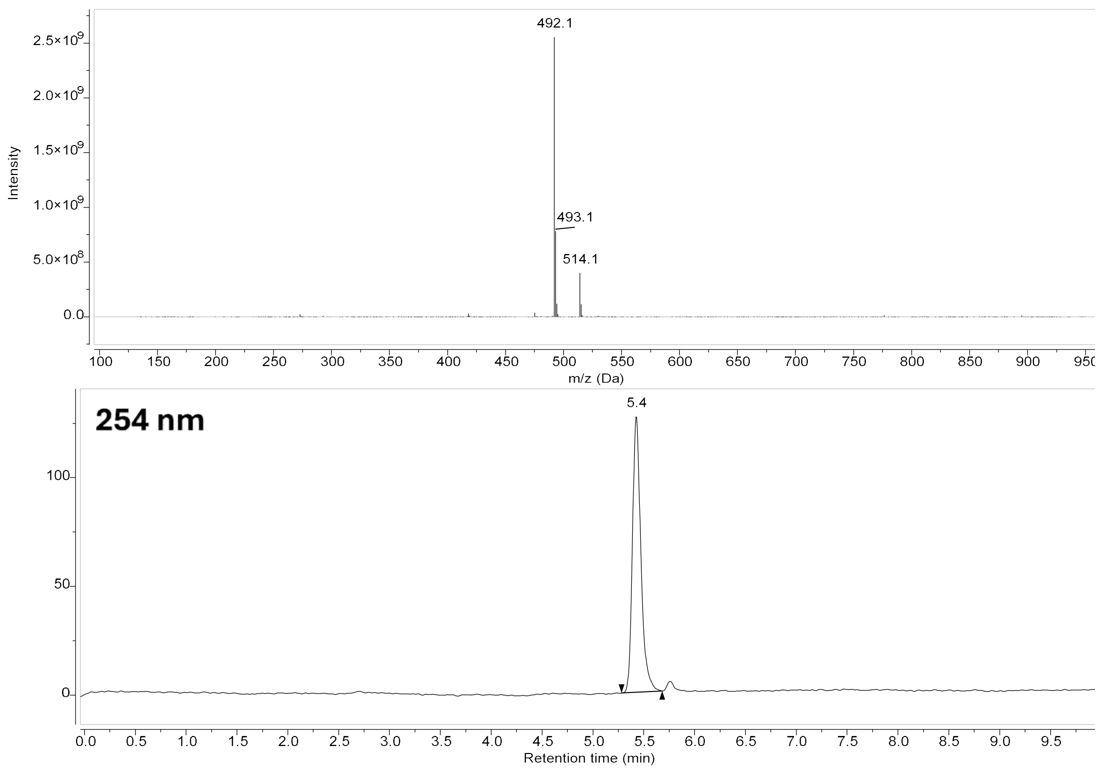

492.1
493.1
514.1
2.5×10^9
2.0×10^9
1.5×10^9
1.0×10^9
5.0×10^8
0.0
Intensity
100
150
200
250
300
350
400
450
500
550
600
650
700
750
800
850
900
950
m/z (Da)
254 nm
5.4
100
50
0
0.0
0.5
1.0
1.5
2.0
2.5
3.0
3.5
4.0
4.5
5.0
5.5
6.0
6.5
7.0
7.5
8.0
8.5
9.0
9.5
Retention time (min)

Compound 26:

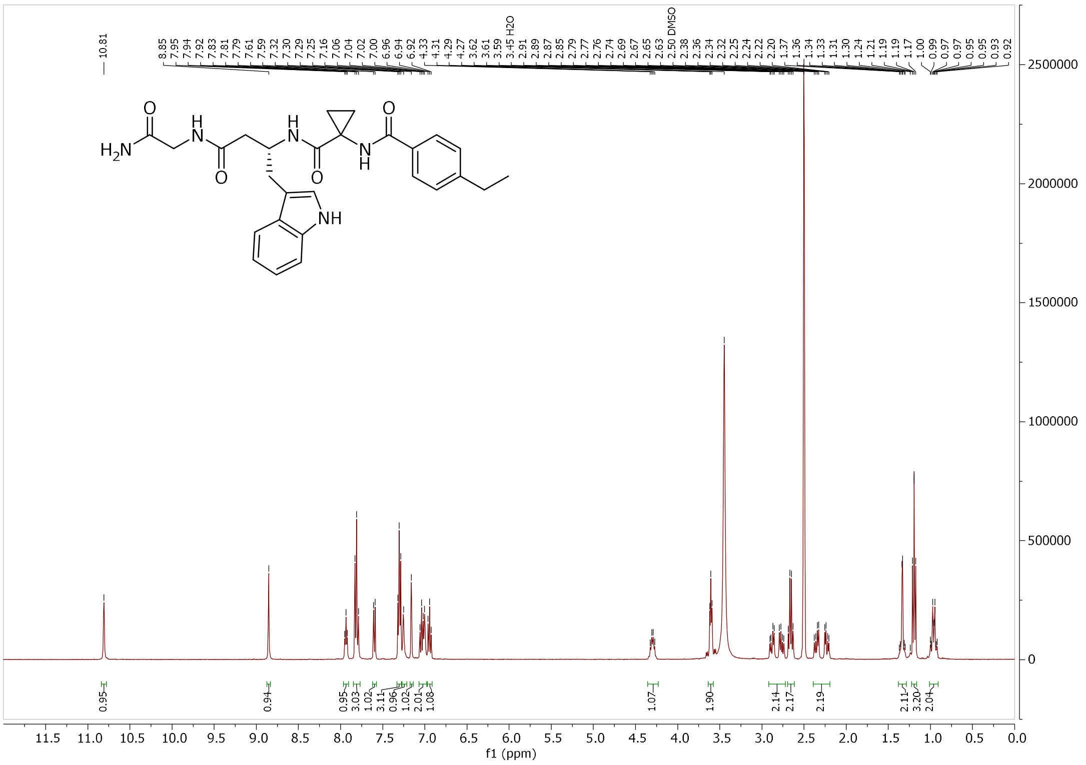

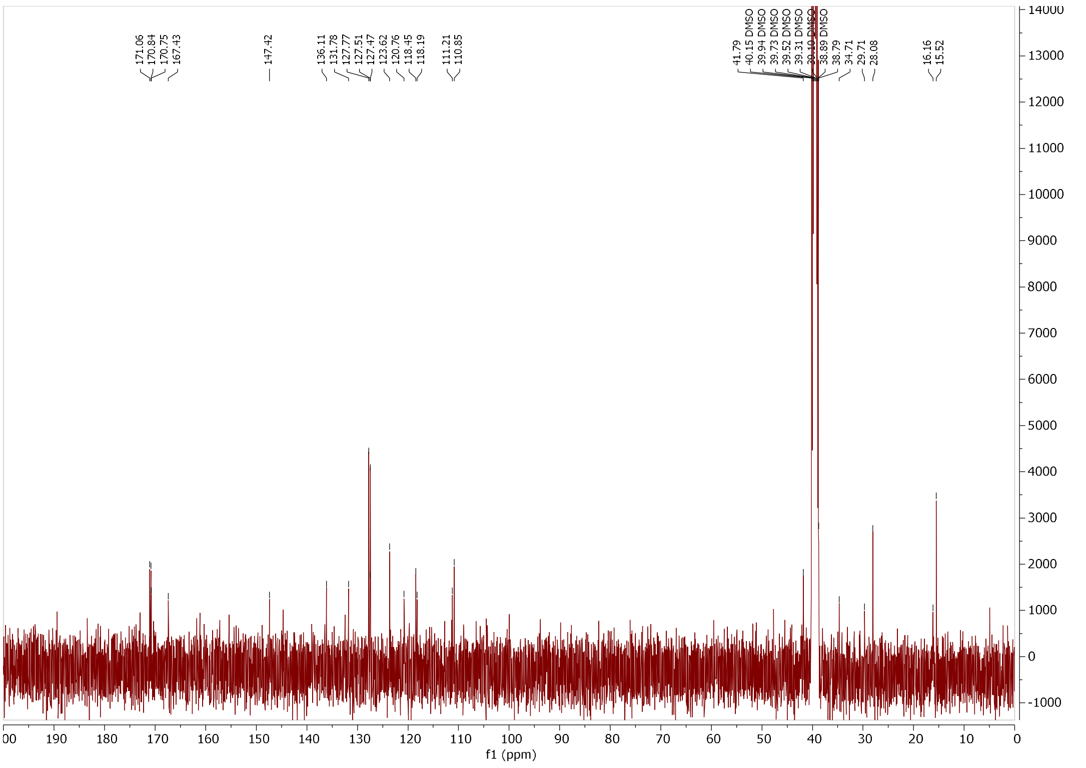

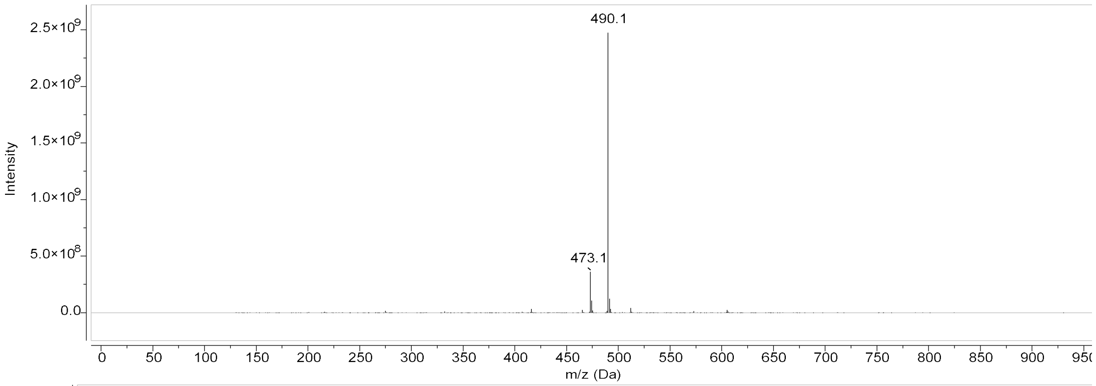

490.1
473.1
2.5×10^9
2.0×10^9
1.5×10^9
1.0×10^9
5.0×10^8
0.0
Intensity
0 50 100 150 200 250 300 350 400 450 500 550 600 650 700 750 800 850 900 950
m/z (Da)

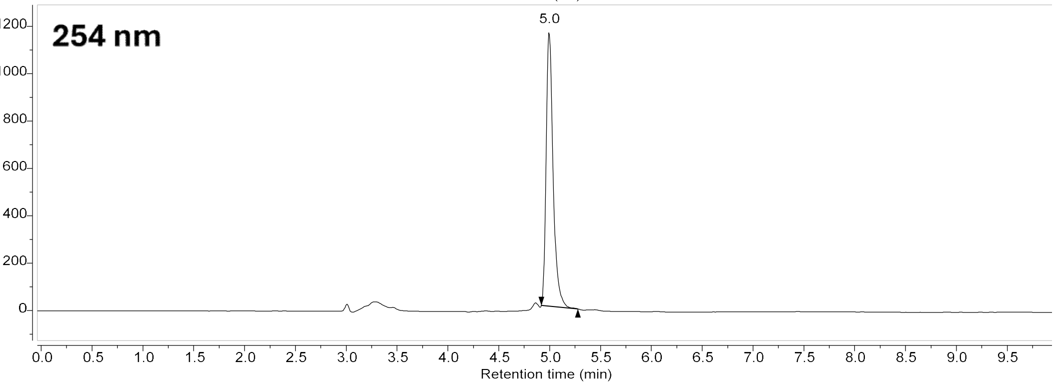

254 nm
5.0
1200
1000
800
600
400
200
0
0.0 0.5 1.0 1.5 2.0 2.5 3.0 3.5 4.0 4.5 5.0 5.5 6.0 6.5 7.0 7.5 8.0 8.5 9.0 9.5
Retention time (min)

Compound 27:

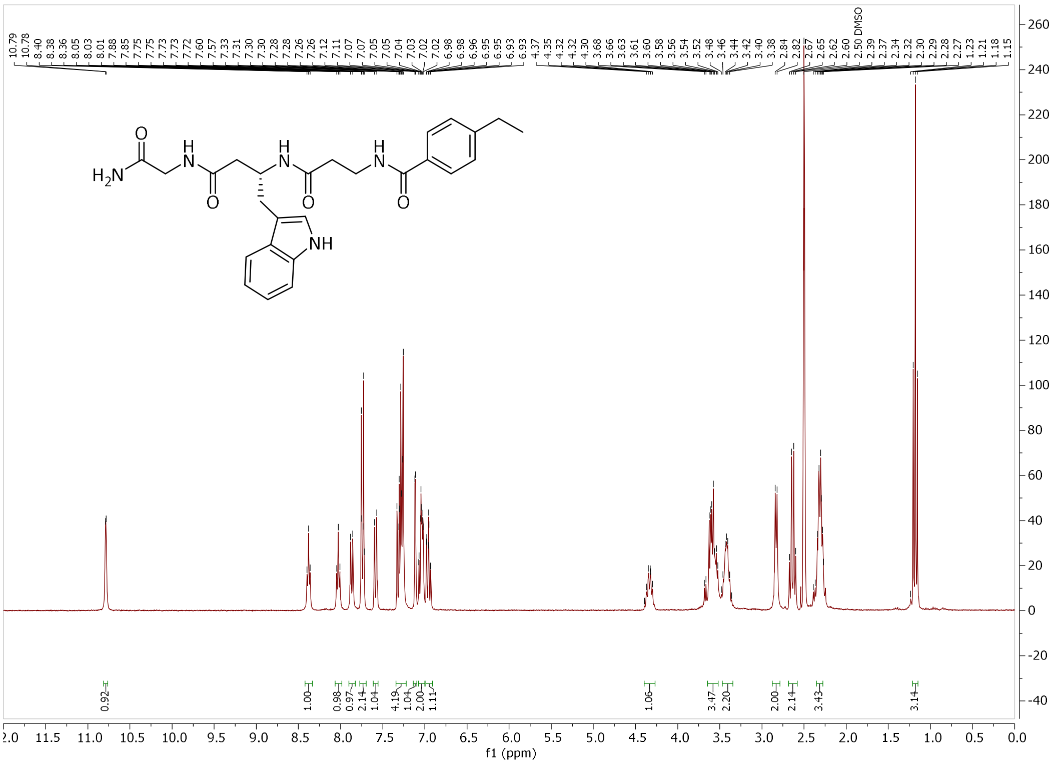

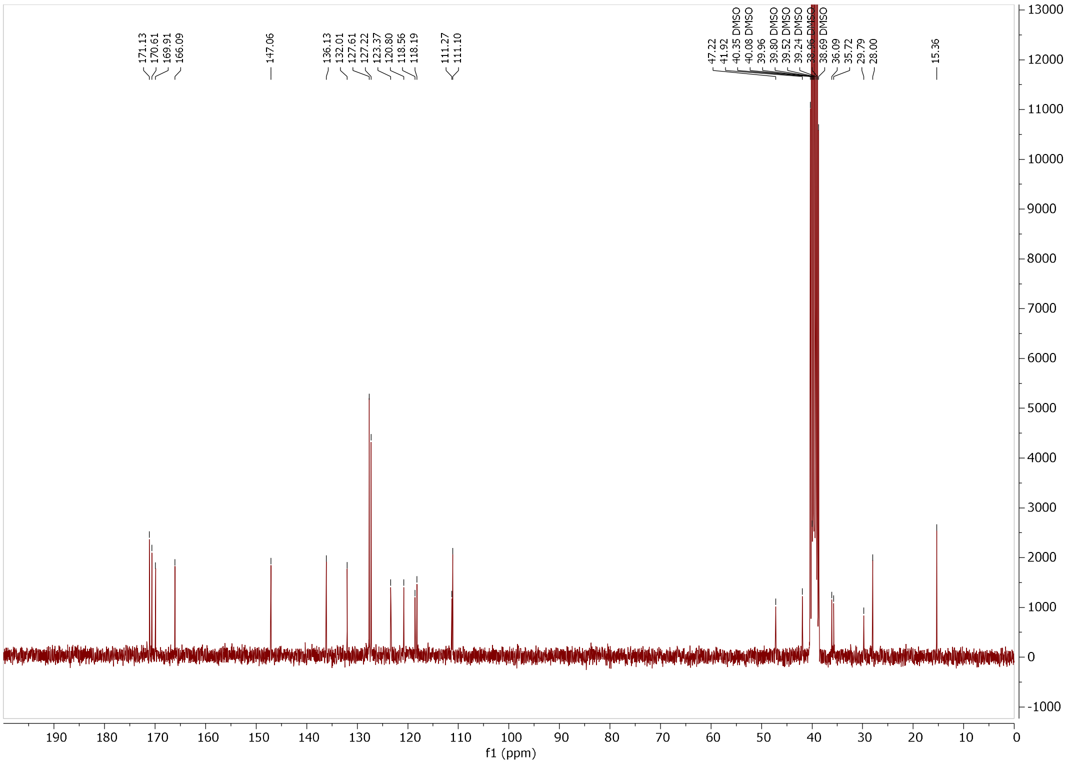

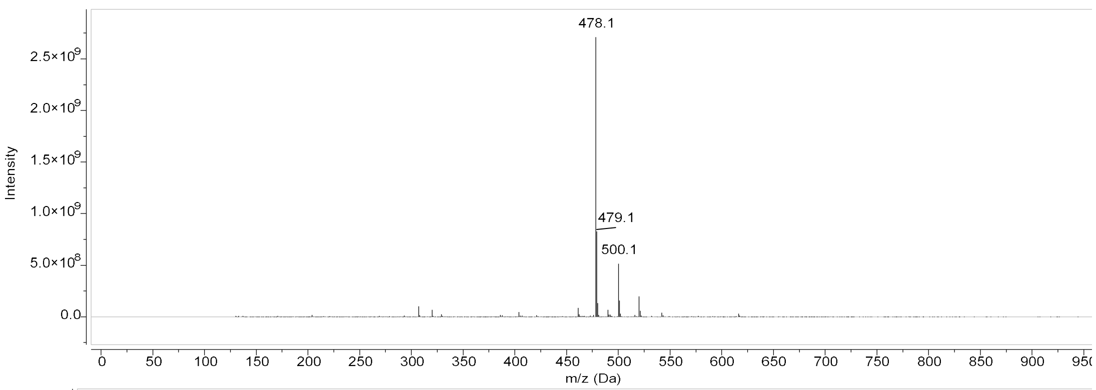
478.1
479.1
500.1
Intensity
2.5×10^9
2.0×10^9
1.5×10^9
1.0×10^9
5.0×10^8
0.0
0 50 100 150 200 250 300 350 400 450 500 550 600 650 700 750 800 850 900 950
m/z (Da)

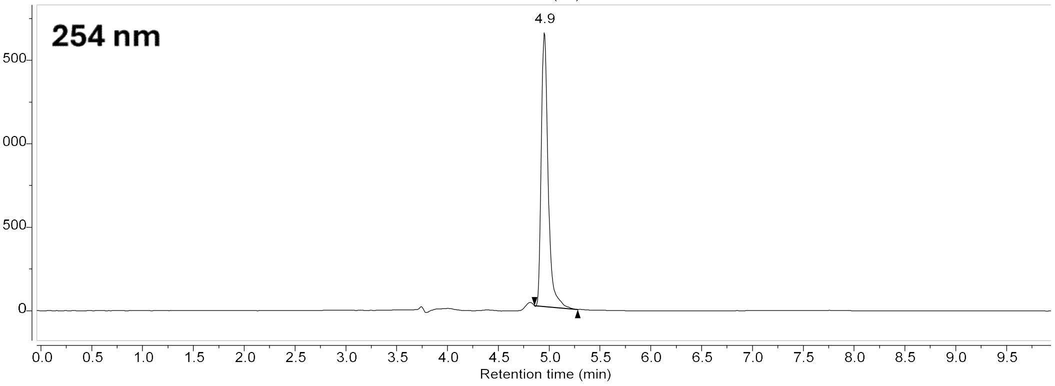
254 nm
4.9
1500
1000
500
0
0.0 0.5 1.0 1.5 2.0 2.5 3.0 3.5 4.0 4.5 5.0 5.5 6.0 6.5 7.0 7.5 8.0 8.5 9.0 9.5
Retention time (min)

Compound 28:

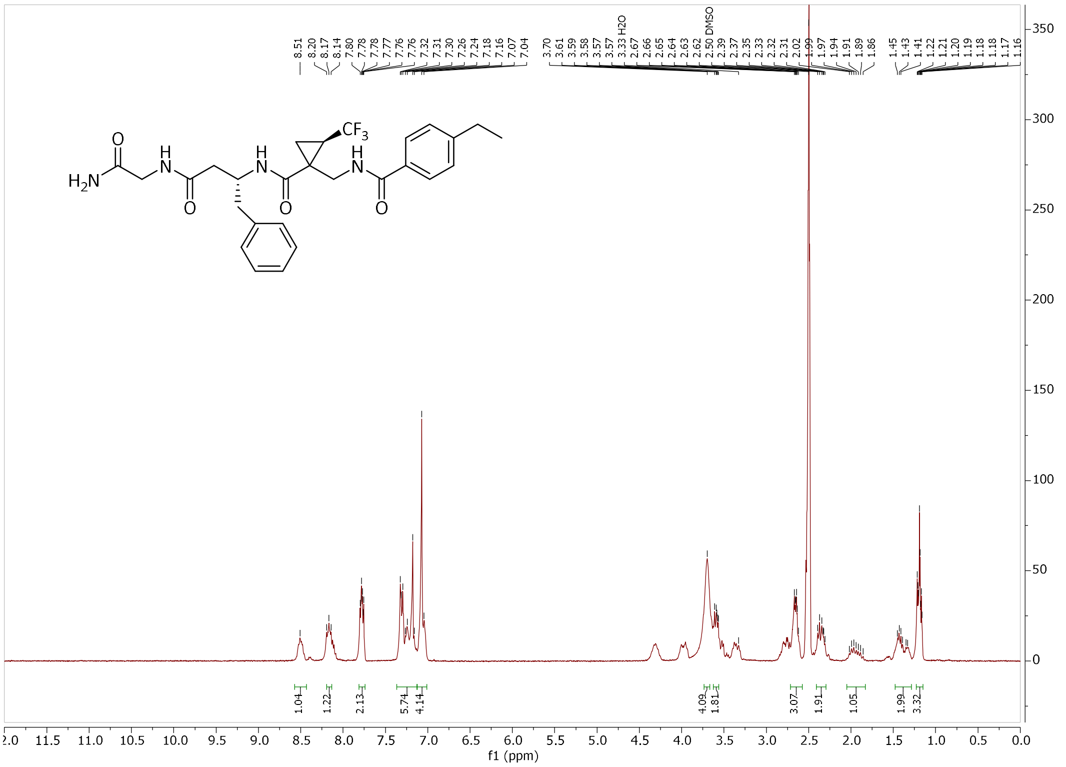

8.51
8.20
8.17
8.14
7.80
7.78
7.78
7.77
7.76
7.76
7.32
7.31
7.30
7.26
7.24
7.18
7.16
7.07
7.04
3.70
3.61
3.59
3.58
3.57
3.57
3.33 H2O
2.67
2.66
2.65
2.64
2.63
2.62
2.50 DMSO
2.39
2.37
2.35
2.33
2.32
2.31
2.02
1.99
1.97
1.94
1.91
1.89
1.86
1.45
1.43
1.41
1.22
1.21
1.20
1.19
1.18
1.18
1.17
1.16
CF_3
O
H_2N
H
N
O
H
N
O
H
N
O
1.04
1.22
2.13
5.74
4.14
4.09
1.81
3.07
1.91
1.05
1.99
3.32
f1 (ppm)

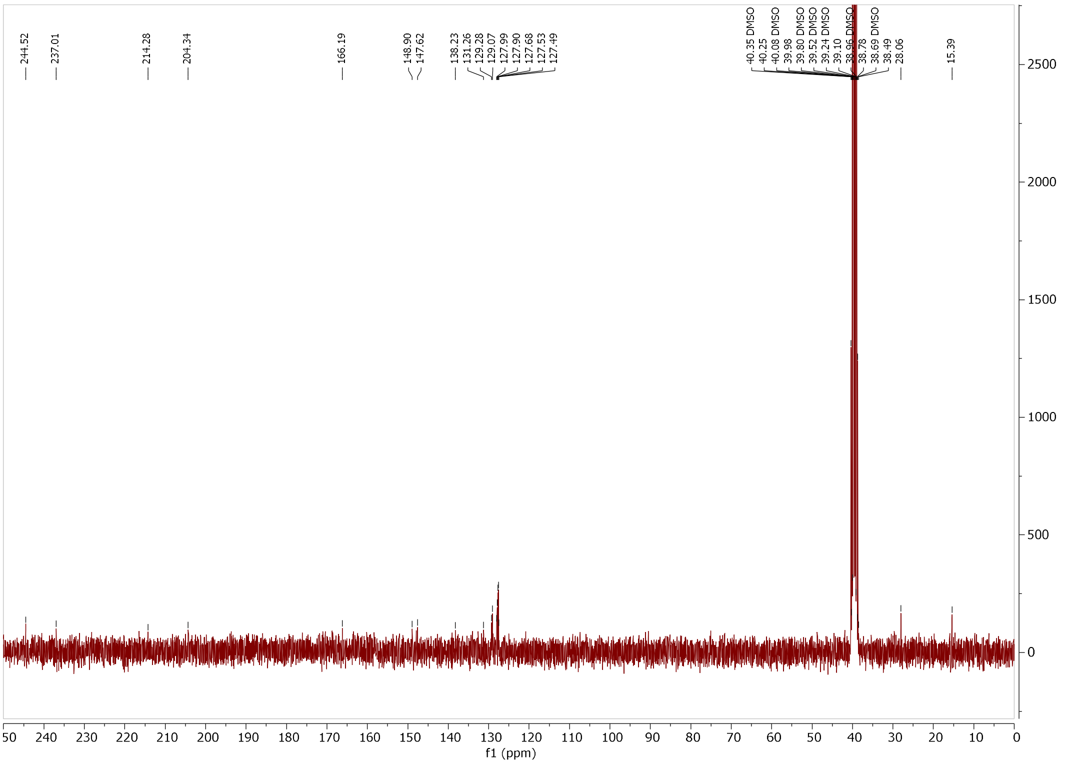

244.52
237.01
214.28
204.34
166.19
148.90
147.62
138.23
131.26
129.28
129.07
127.99
127.90
127.68
127.53
127.49
40.35 DMSO
40.25
40.08 DMSO
39.98
39.80 DMSO
39.52 DMSO
39.24 DMSO
39.10
38.96 DMSO
38.78
38.69 DMSO
38.49
28.06
15.39
f1 (ppm)

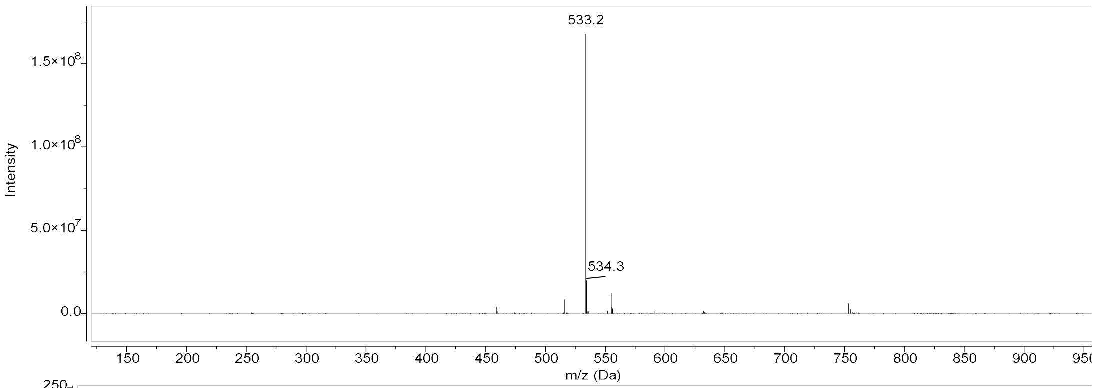

533.2
534.3
Intensity
1.5×10^8
1.0×10^8
5.0×10^7
0.0
150 200 250 300 350 400 450 500 550 600 650 700 750 800 850 900 950
m/z (Da)

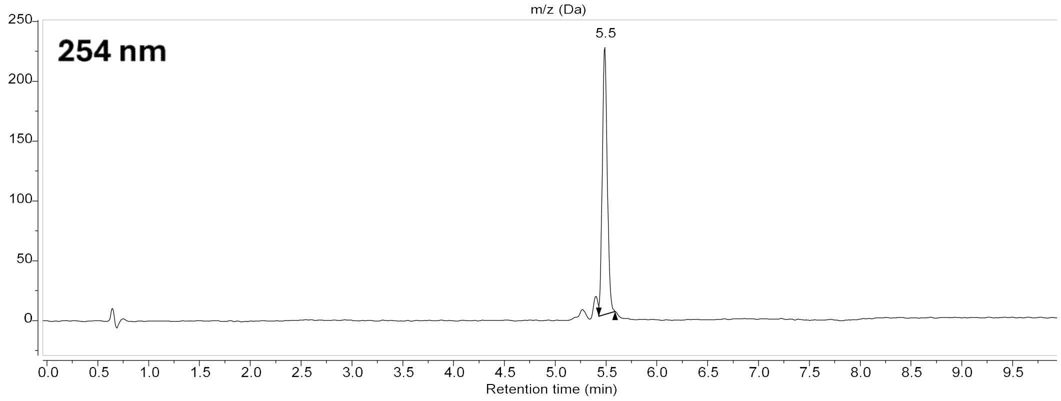

254 nm
5.5
250 200 150 100 50 0
0.0 0.5 1.0 1.5 2.0 2.5 3.0 3.5 4.0 4.5 5.0 5.5 6.0 6.5 7.0 7.5 8.0 8.5 9.0 9.5
Retention time (min)

Compound 29:

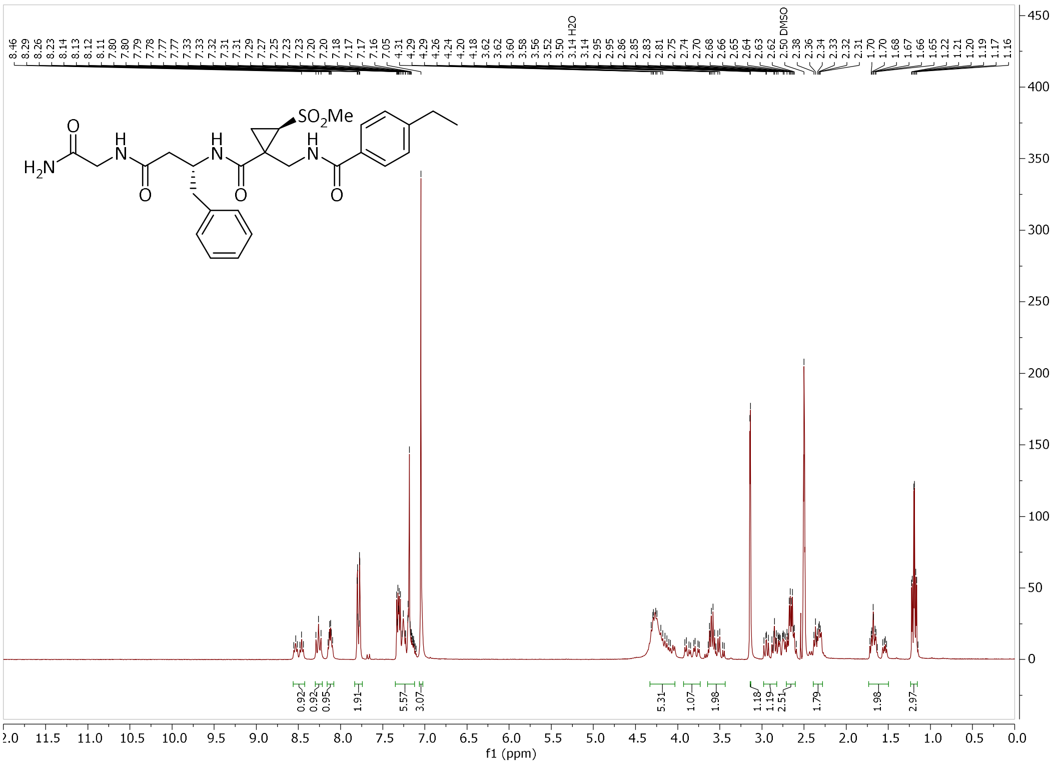

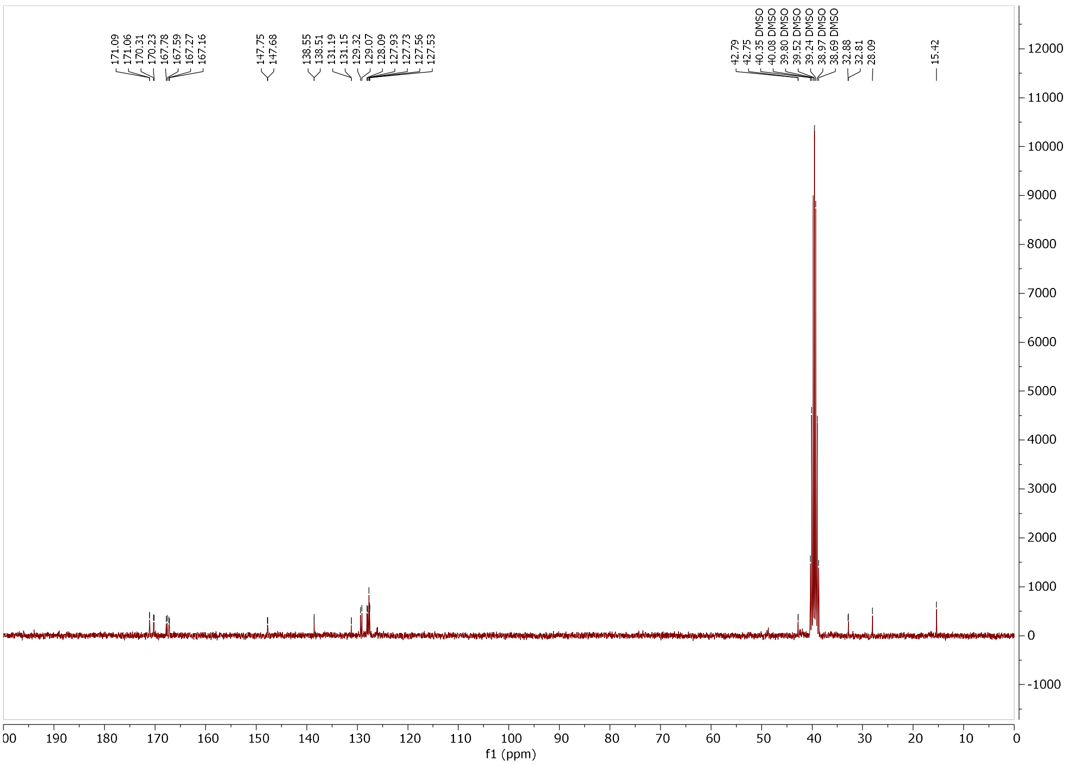

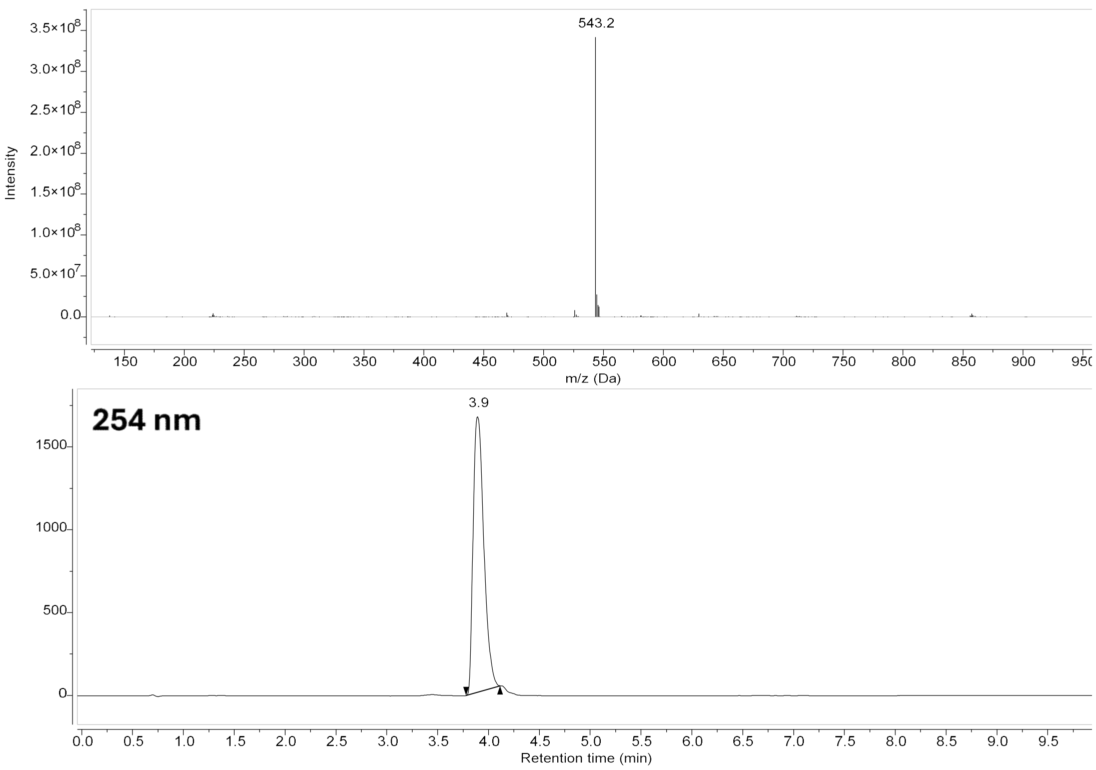

543.2
Intensity
3.5×10^8
3.0×10^8
2.5×10^8
2.0×10^8
1.5×10^8
1.0×10^8
5.0×10^7
0.0
150 200 250 300 350 400 450 500 550 600 650 700 750 800 850 900 950
m/z (Da)
254 nm
3.9
1500
1000
500
0
0.0 0.5 1.0 1.5 2.0 2.5 3.0 3.5 4.0 4.5 5.0 5.5 6.0 6.5 7.0 7.5 8.0 8.5 9.0 9.5
Retention time (min)

Compound 30:

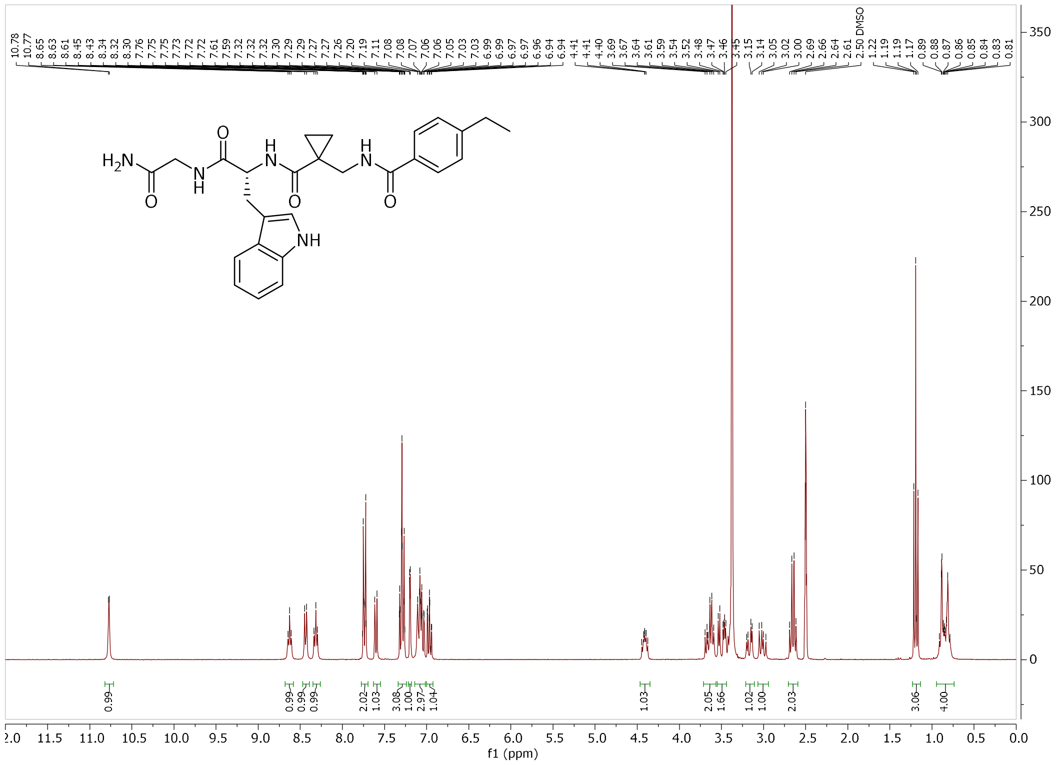

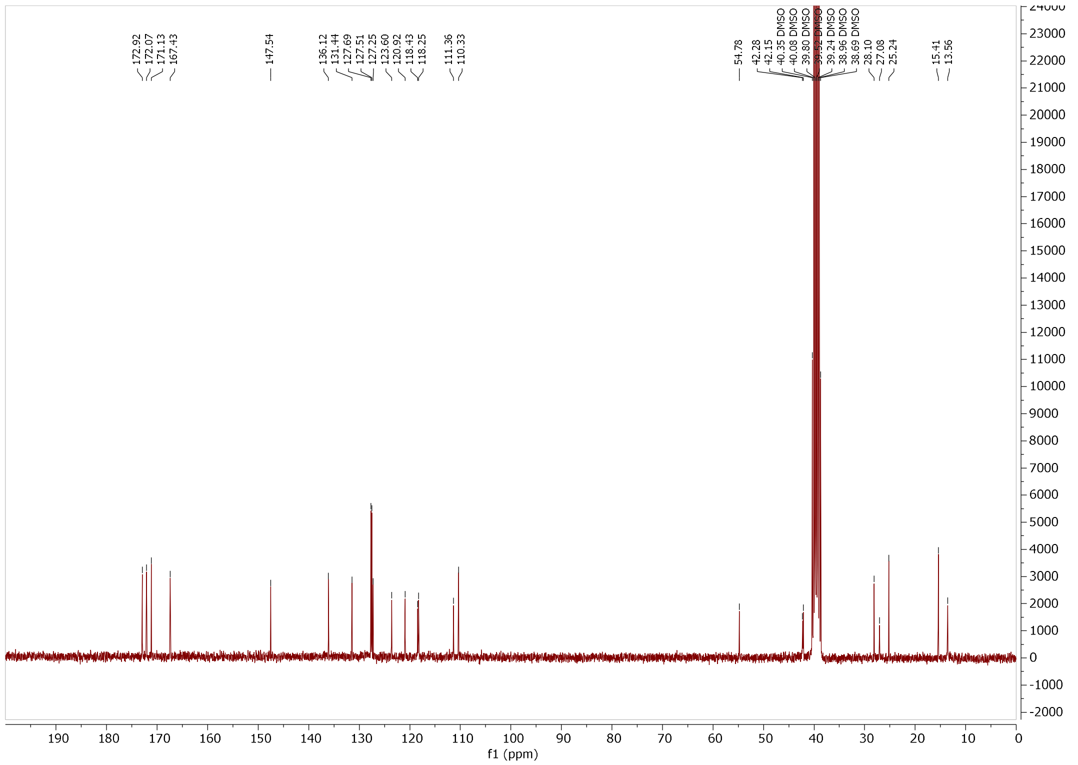

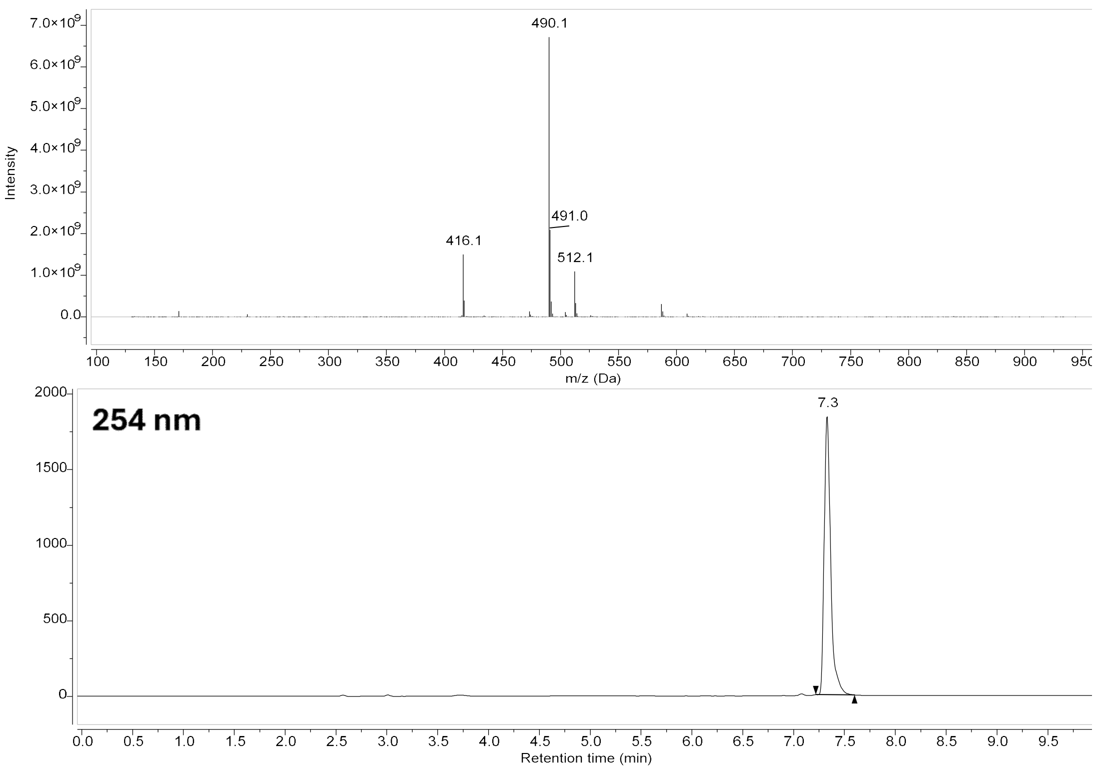

Intensity
490.1
491.0
416.1
512.1
m/z (Da)
254 nm
7.3
Retention time (min)

Compound 31:

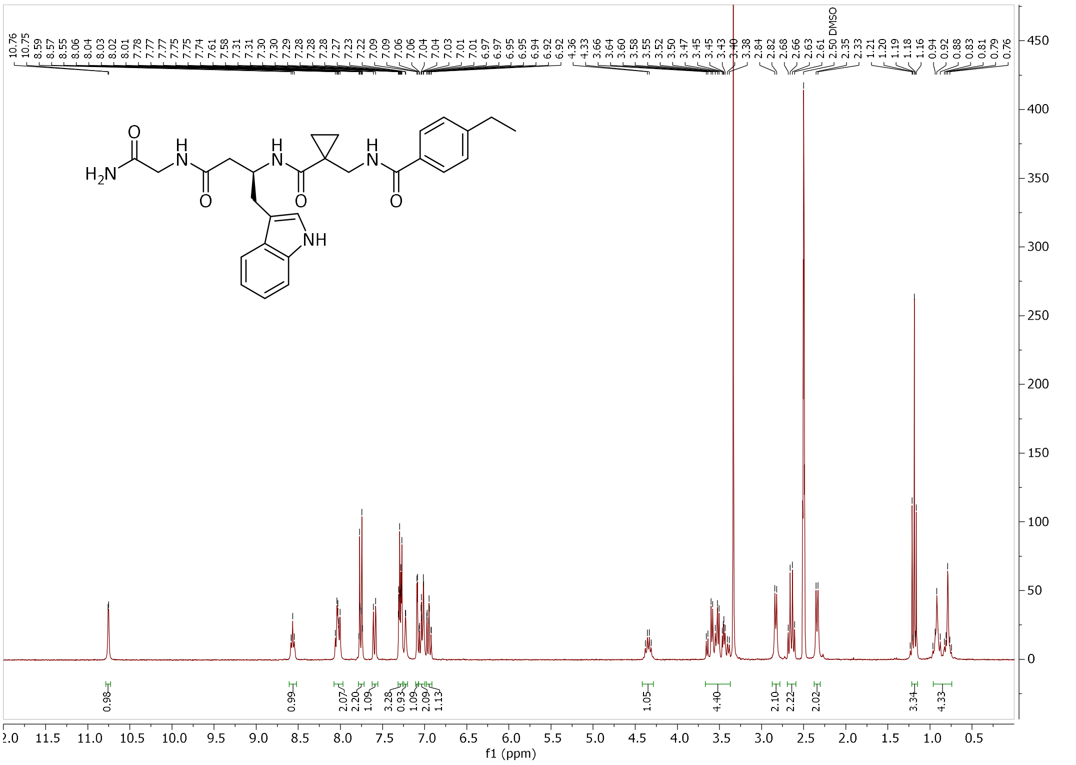

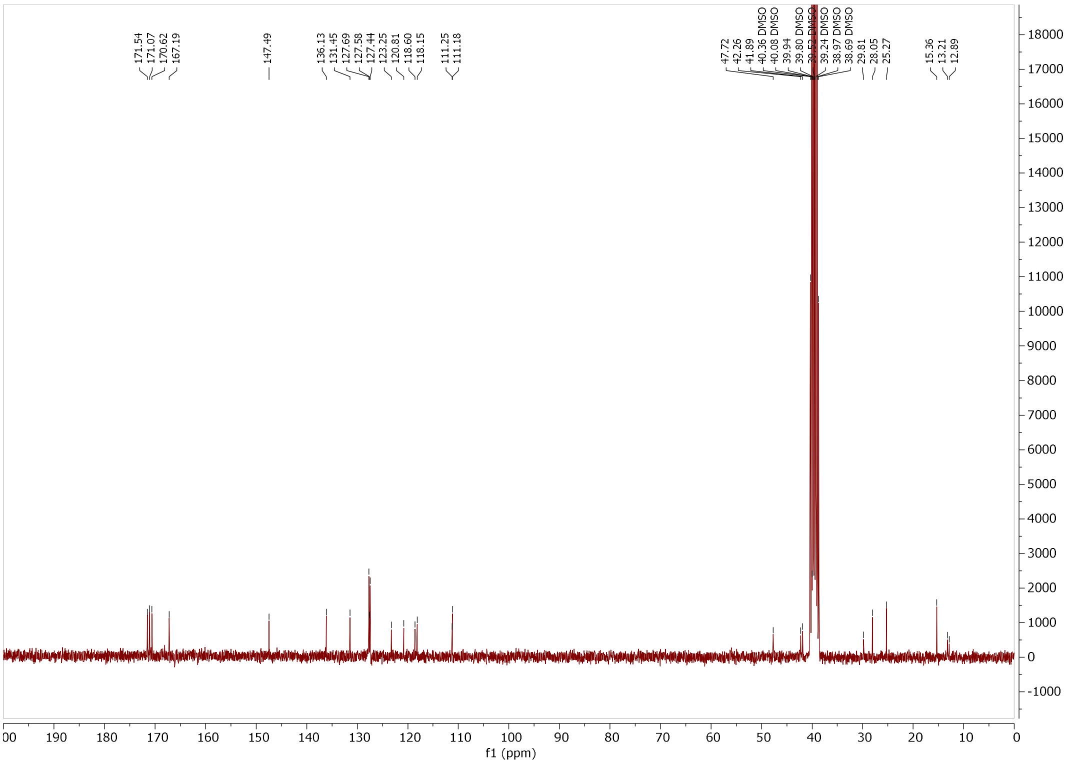

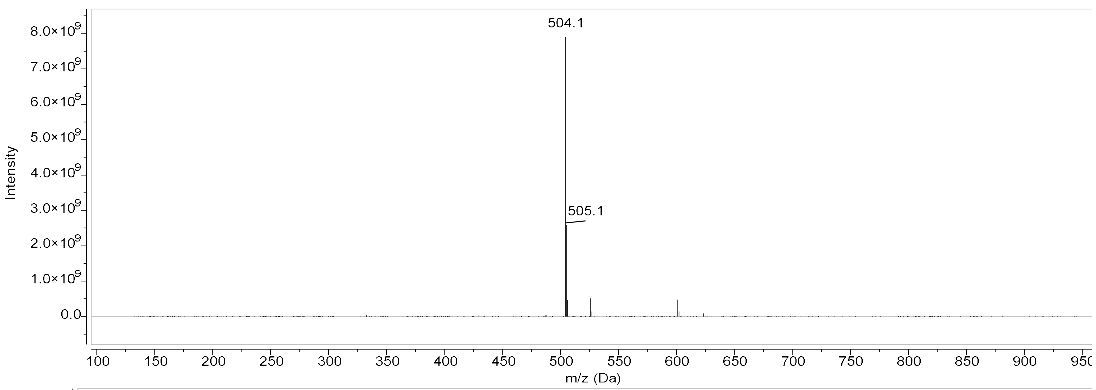
504.1
505.1
Intensity
8.0×10^9
7.0×10^9
6.0×10^9
5.0×10^9
4.0×10^9
3.0×10^9
2.0×10^9
1.0×10^9
0.0
m/z (Da)

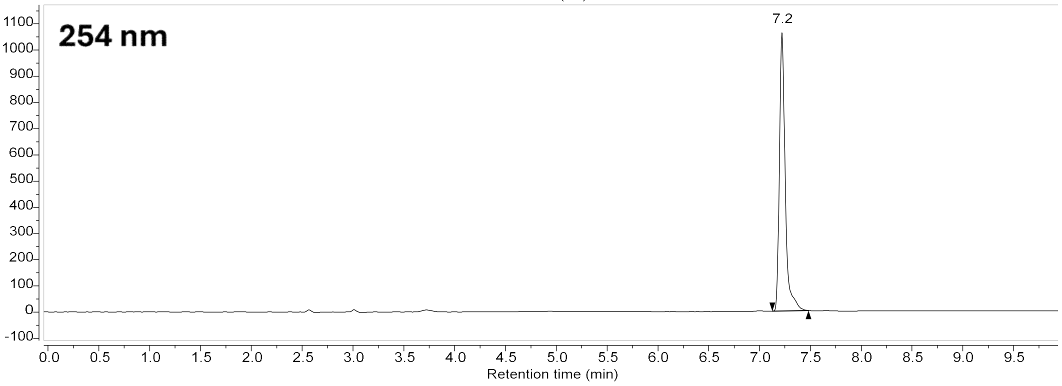
254 nm
7.2
Retention time (min)

Compound 32:

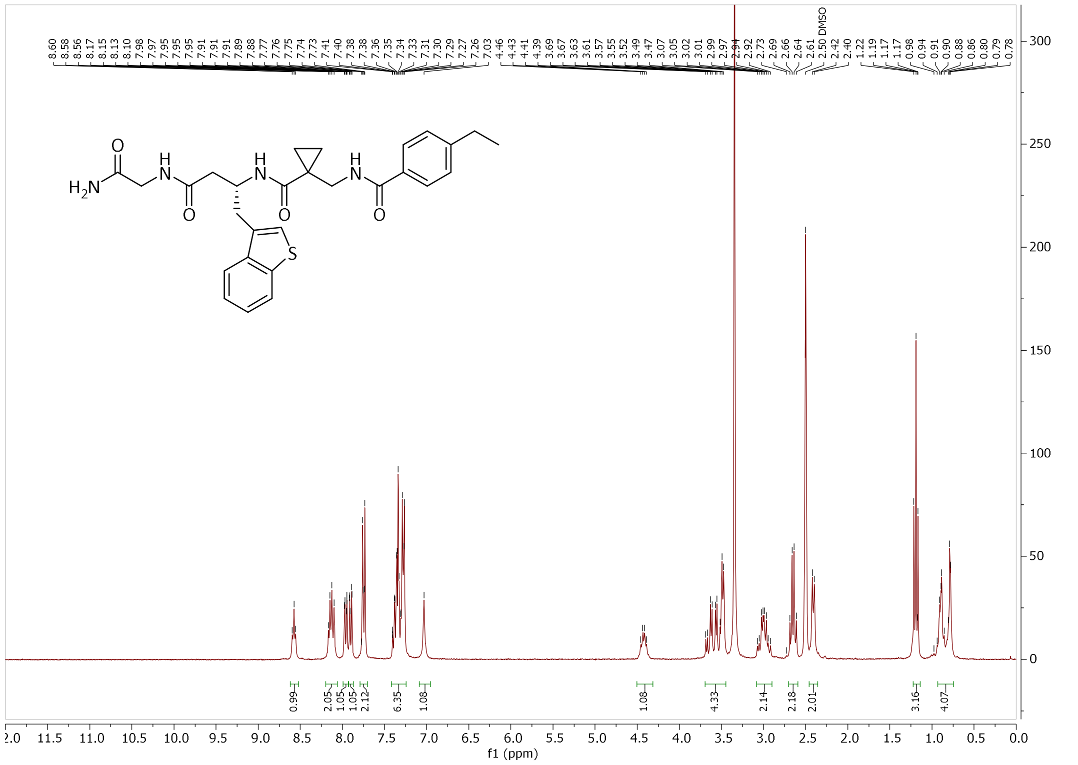
8.60 8.58 8.56 8.17 8.15 8.13 8.10 7.98 7.97 7.95 7.95 7.95 7.91 7.91 7.89 7.88 7.77 7.76 7.75 7.74 7.73 7.41 7.40 7.38 7.38 7.36 7.35 7.34 7.33 7.31 7.30 7.29 7.27 7.26 7.03
4.46 4.43 4.41 4.39 3.69 3.67 3.63 3.61 3.57 3.55 3.52 3.49 3.47 3.07 3.05 3.02 3.01 2.99 2.97 2.94 2.92 2.73 2.69 2.66 2.64 2.61
2.50 DMSO
2.42 2.40 1.22 1.19 1.17 0.98 0.94 0.91 0.90 0.88 0.86 0.80 0.79 0.78
H2N
O
H
N
O
H
N
O
H
N
O
S
0.99 2.05 1.05 1.05 2.12 6.35 1.08 1.08 4.33 2.14 2.18 2.01 3.16 4.07
f1 (ppm)

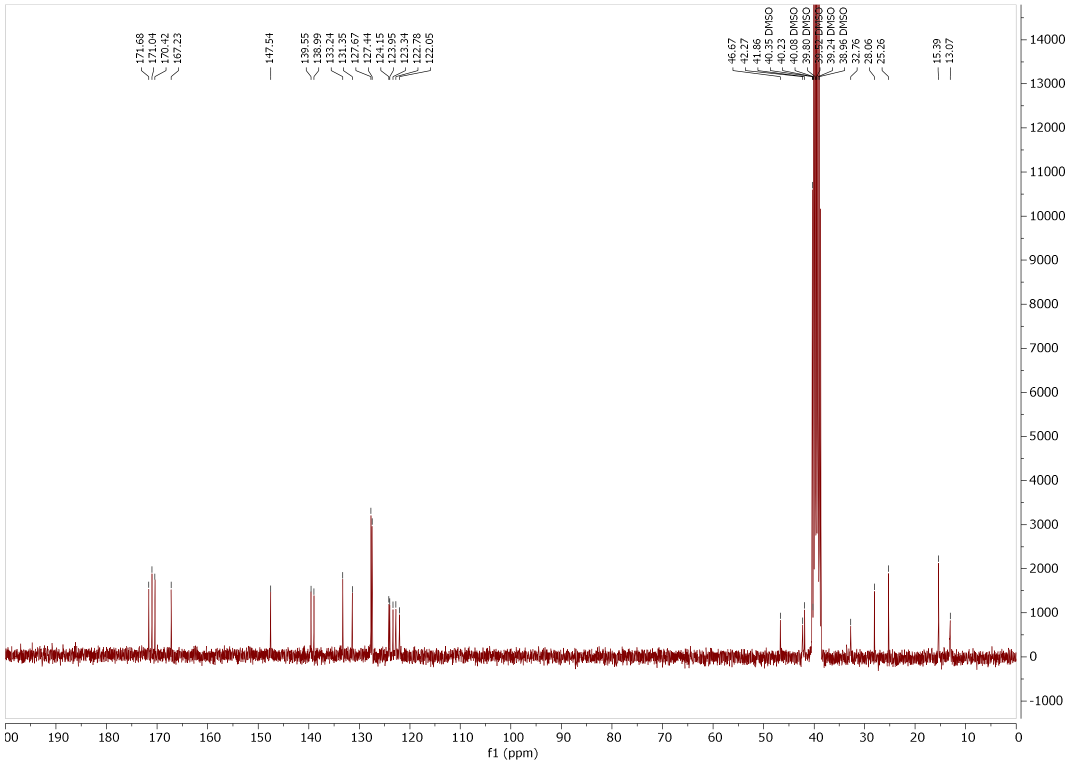
171.68 171.04 170.42 167.23
147.54
139.55 138.99 133.24 131.35 127.67 127.44 124.15 123.95 123.34 122.78 122.05
46.67 42.27 41.86
40.35 DMSO
40.23
40.08 DMSO
39.80 DMSO
39.52 DMSO
39.24 DMSO
38.96 DMSO
32.76 28.06 25.26
15.39 13.07
f1 (ppm)

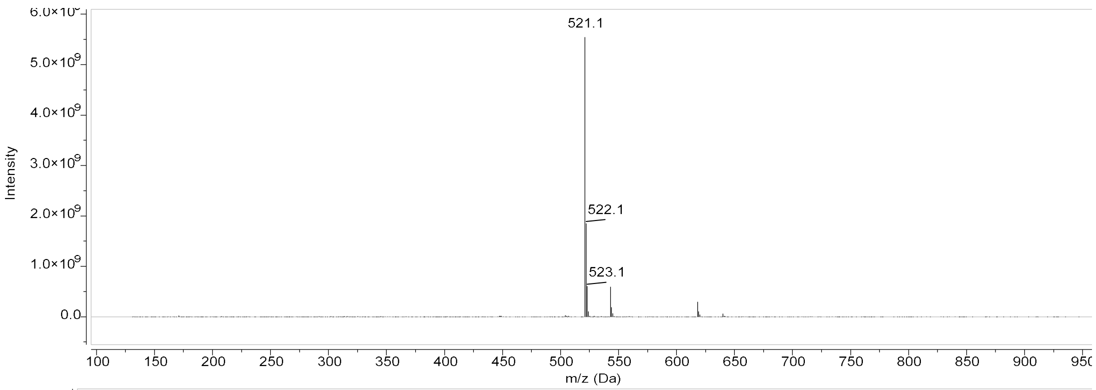
521.1
522.1
523.1
Intensity
m/z (Da)

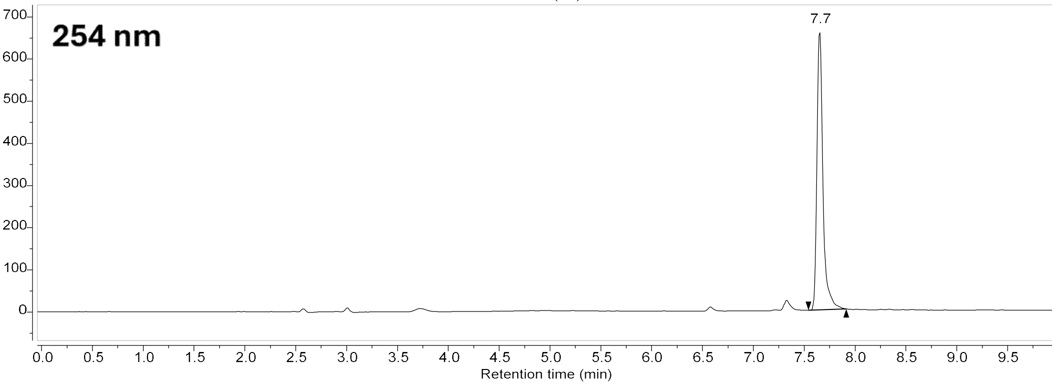
254 nm
7.7
Retention time (min)

Compound 14:

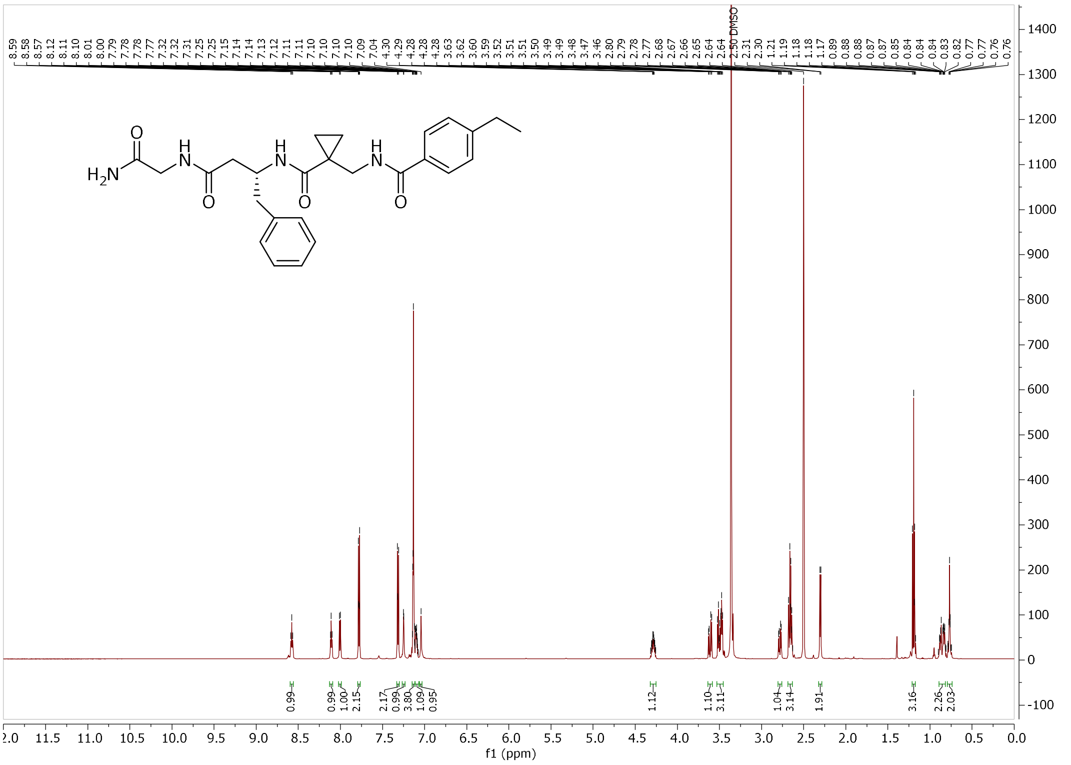
8.59 8.58 8.57 8.12 8.11 8.10 8.01 8.00 7.79 7.78 7.78 7.77 7.32 7.32 7.31 7.25 7.25 7.15 7.14 7.14 7.13 7.12 7.11 7.11 7.10 7.10 7.10 7.09 7.04 4.30 4.29 4.28 4.28 3.63 3.62 3.60 3.59 3.52 3.51 3.51 3.50 3.49 3.49 3.48 3.47 3.46 2.80 2.79 2.78 2.77 2.68 2.67 2.66 2.65 2.64 2.64 2.50 DMSO 2.31 2.30 1.21 1.19 1.18 1.18 1.17 0.89 0.88 0.88 0.87 0.87 0.85 0.84 0.84 0.84 0.83 0.82 0.77 0.77 0.76 0.76
H2N
O
H
N
O
H
N
O
H
N
O
0.99
0.99
1.00
2.15
2.17
0.99
3.80
1.09
0.95
1.12
1.10
3.11
1.04
3.14
1.91
3.16
2.26
2.03
f1 (ppm)

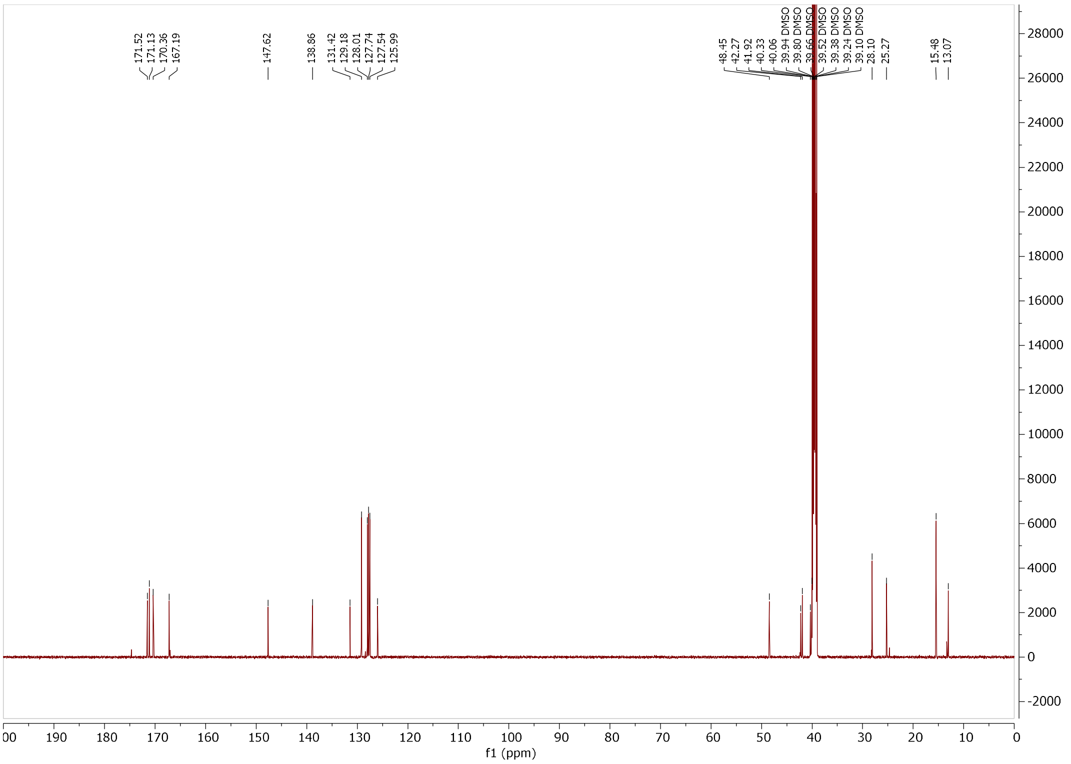
171.52 171.13 170.36 167.19
147.62
138.86
131.42 129.18 128.01 127.74 127.54 125.99
48.45 42.27 41.92 40.33 40.06 DMSO 39.94 DMSO 39.80 DMSO 39.66 DMSO 39.52 DMSO 39.38 DMSO 39.24 DMSO 39.10 DMSO 28.10 25.27
15.48 13.07
f1 (ppm)

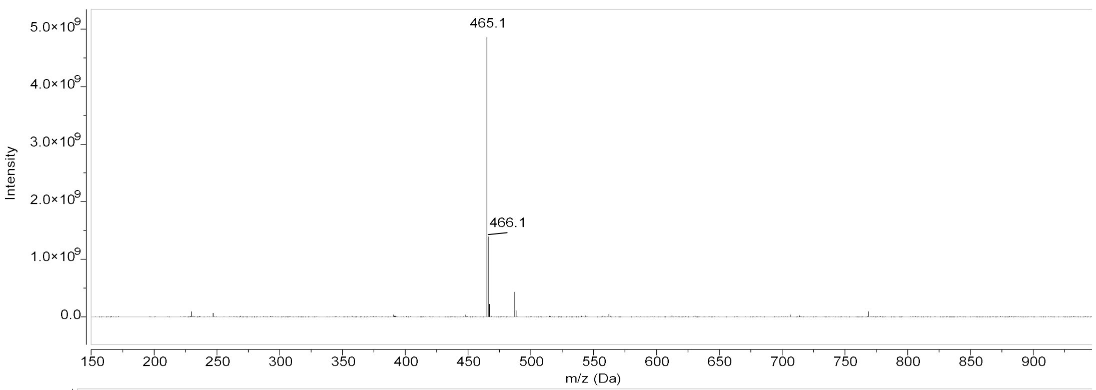
465.1
466.1
Intensity
m/z (Da)

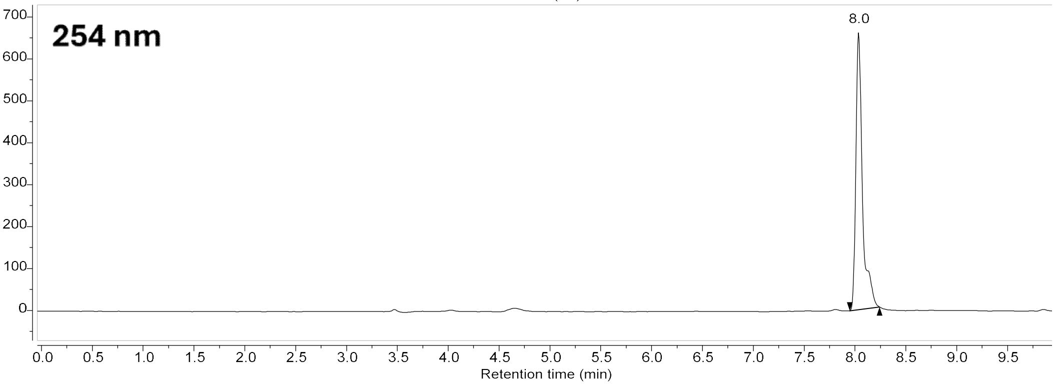
254 nm
8.0
Retention time (min)

Compound 15:

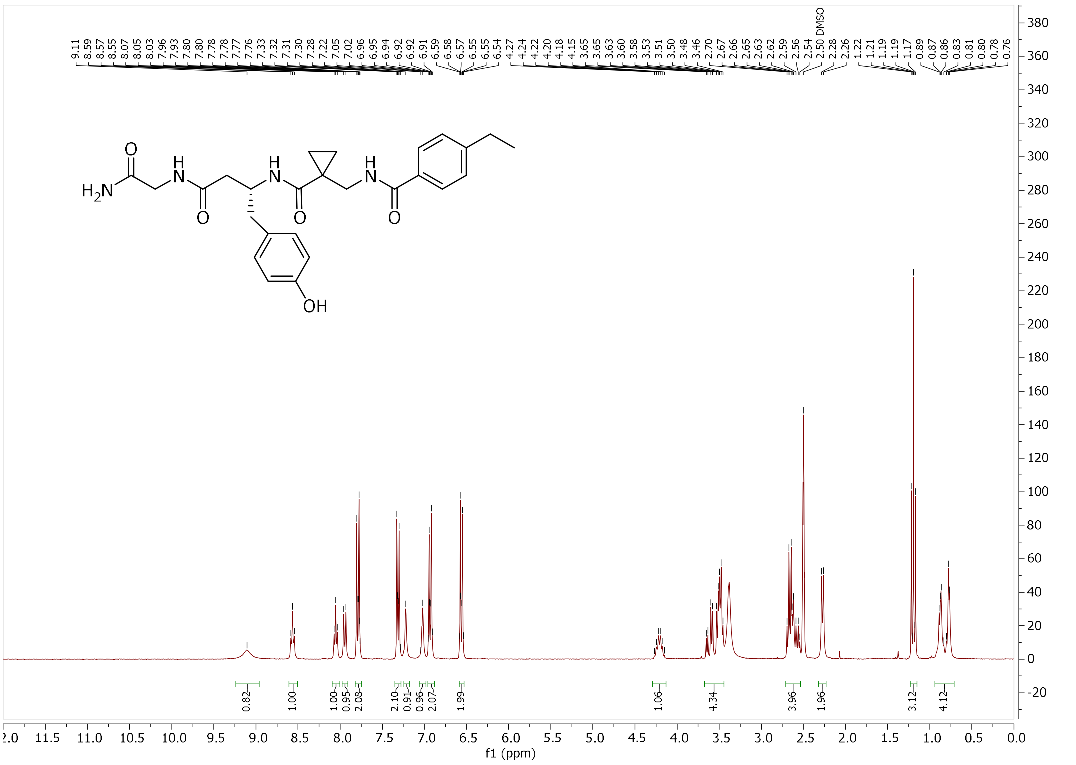

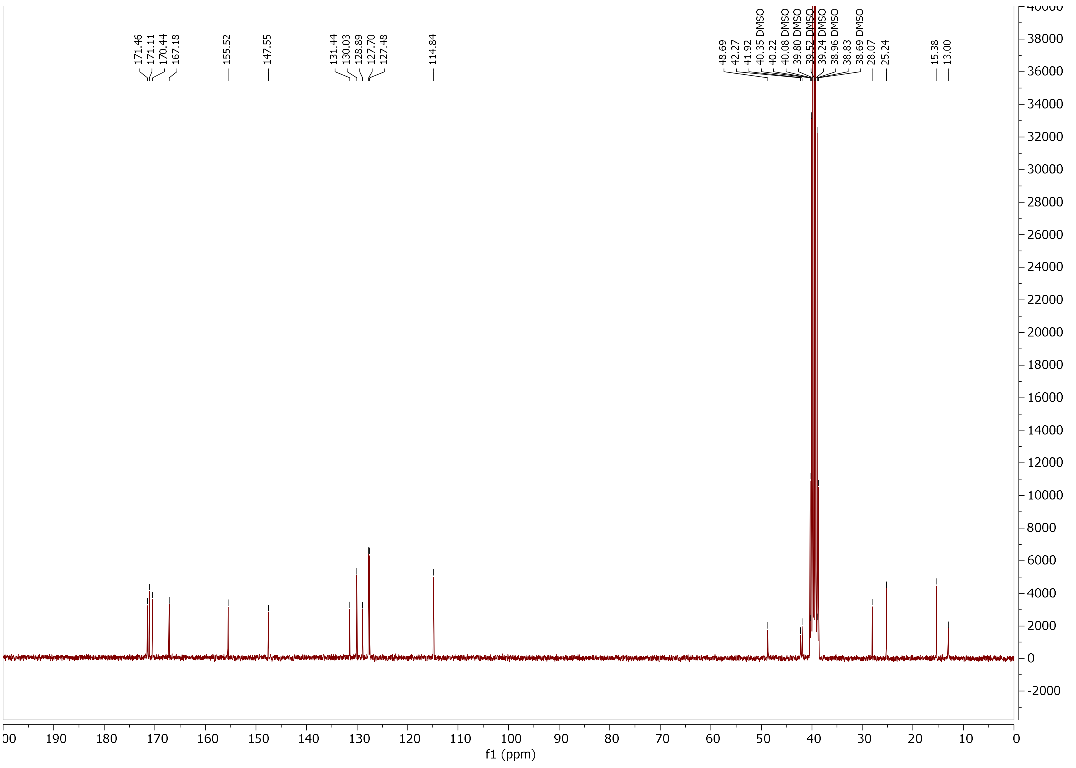

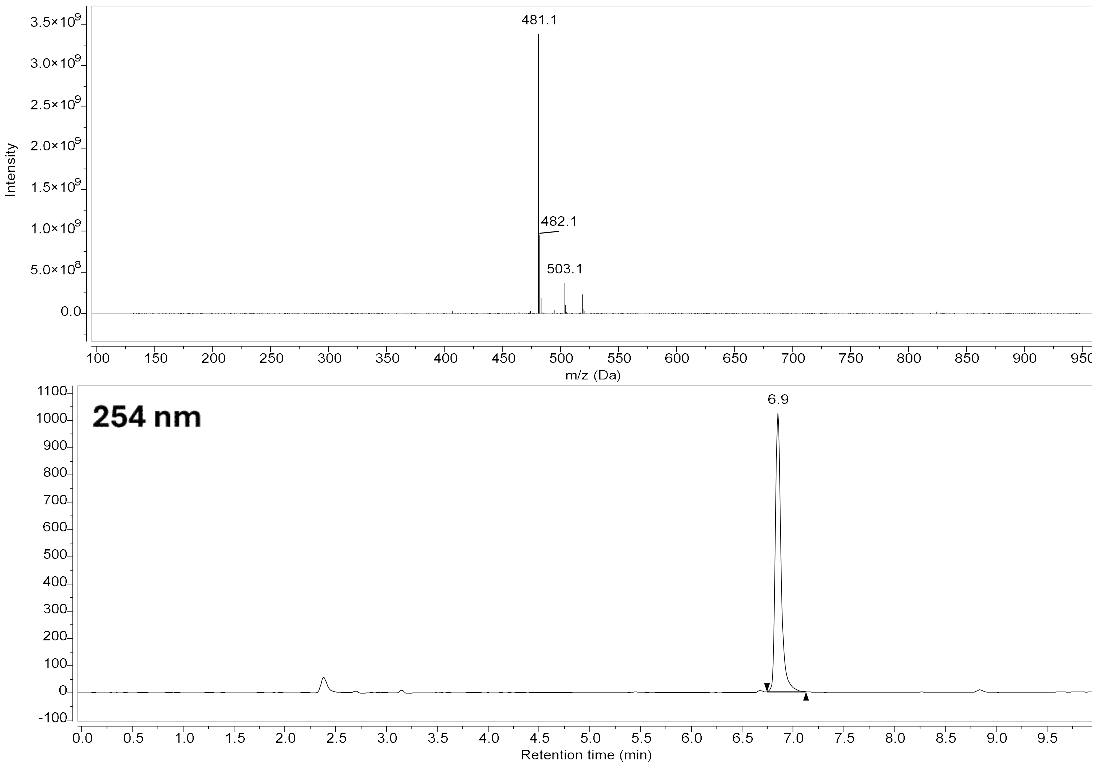
481.1
482.1
503.1
Intensity
m/z (Da)
254 nm
6.9
Retention time (min)

Compound 35:

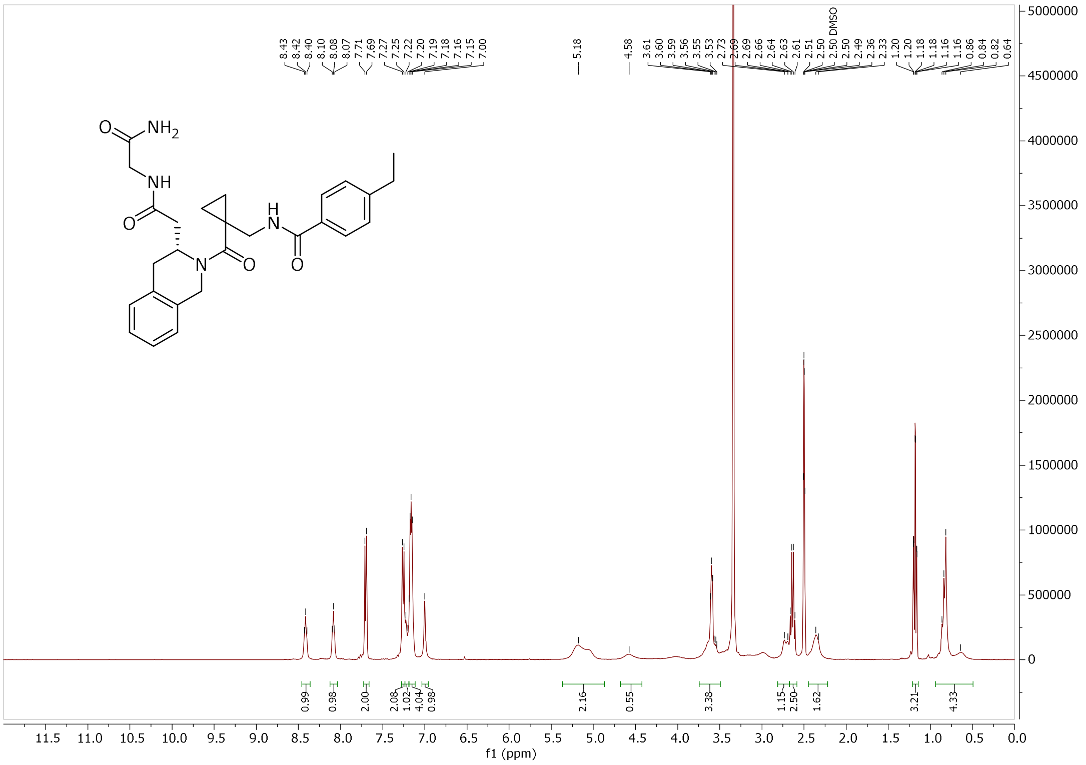
8.43
8.42
8.40
8.10
8.08
8.07
7.71
7.69
7.27
7.25
7.22
7.20
7.19
7.18
7.16
7.15
7.00
5.18
4.58
2.50 DMSO
0.99
0.98
2.00
2.08
1.02
4.04
0.98
2.16
0.55
3.38
1.15
2.50
1.62
3.21
4.33
f1 (ppm)

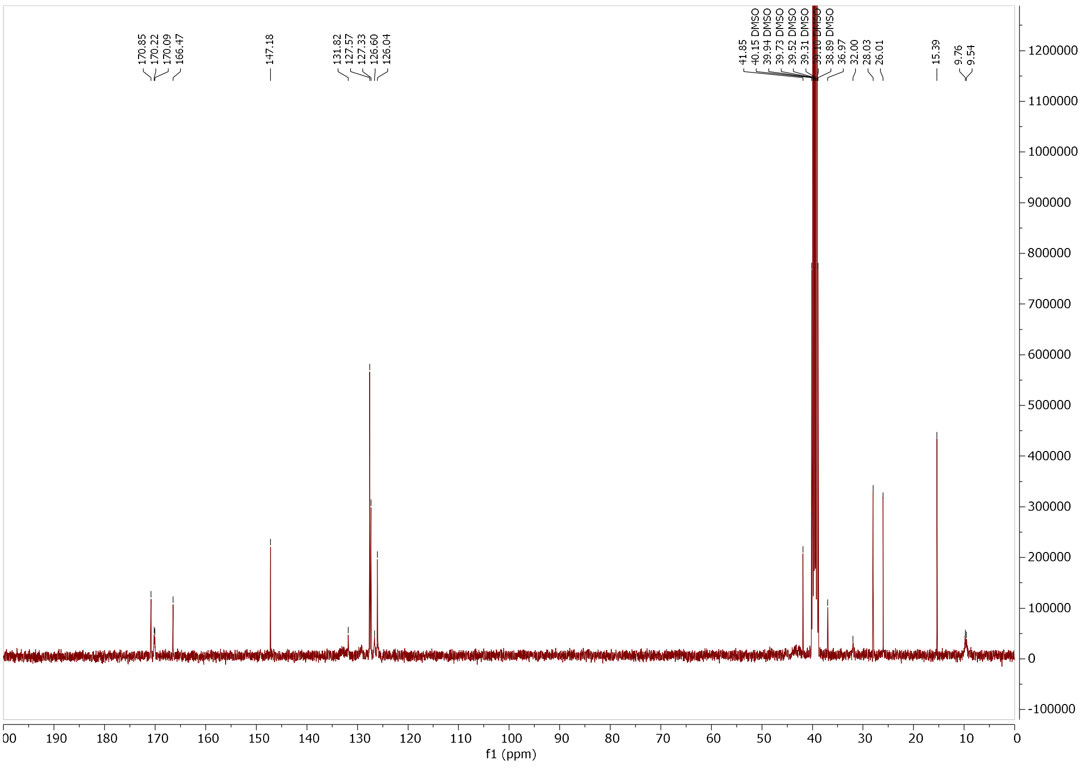
170.85
170.22
170.09
166.47
147.18
131.82
127.57
127.33
126.60
126.04
41.85
40.15 DMSO
39.94 DMSO
39.73 DMSO
39.52 DMSO
39.31 DMSO
38.89 DMSO
36.97
32.00
28.03
26.01
15.39
9.76
9.54
f1 (ppm)

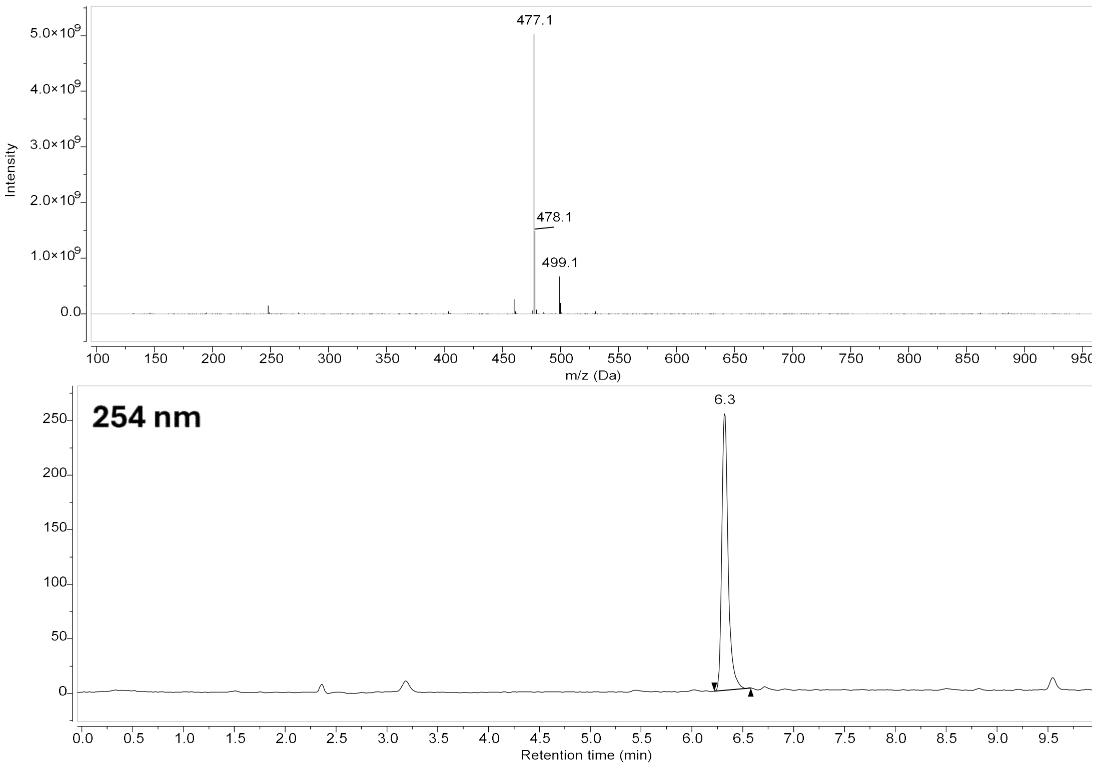
477.1
478.1
499.1
Intensity
5.0×10^9
4.0×10^9
3.0×10^9
2.0×10^9
1.0×10^9
0.0
m/z (Da)
254 nm
6.3
Retention time (min)

Compound 36:

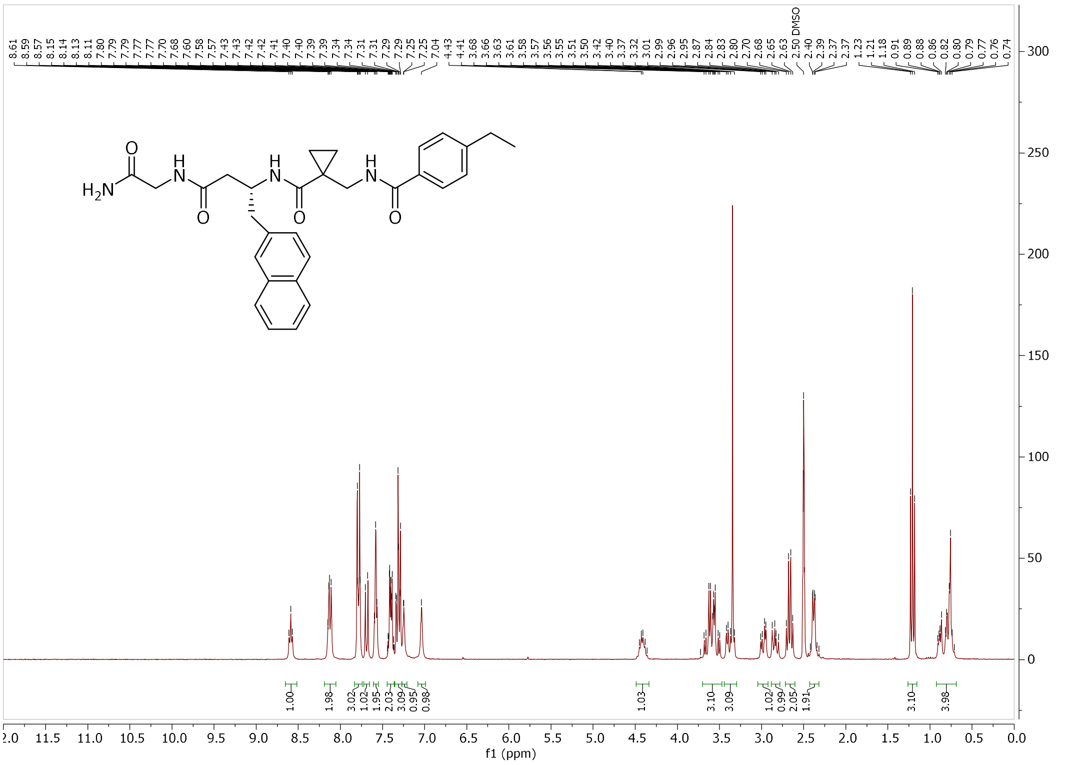

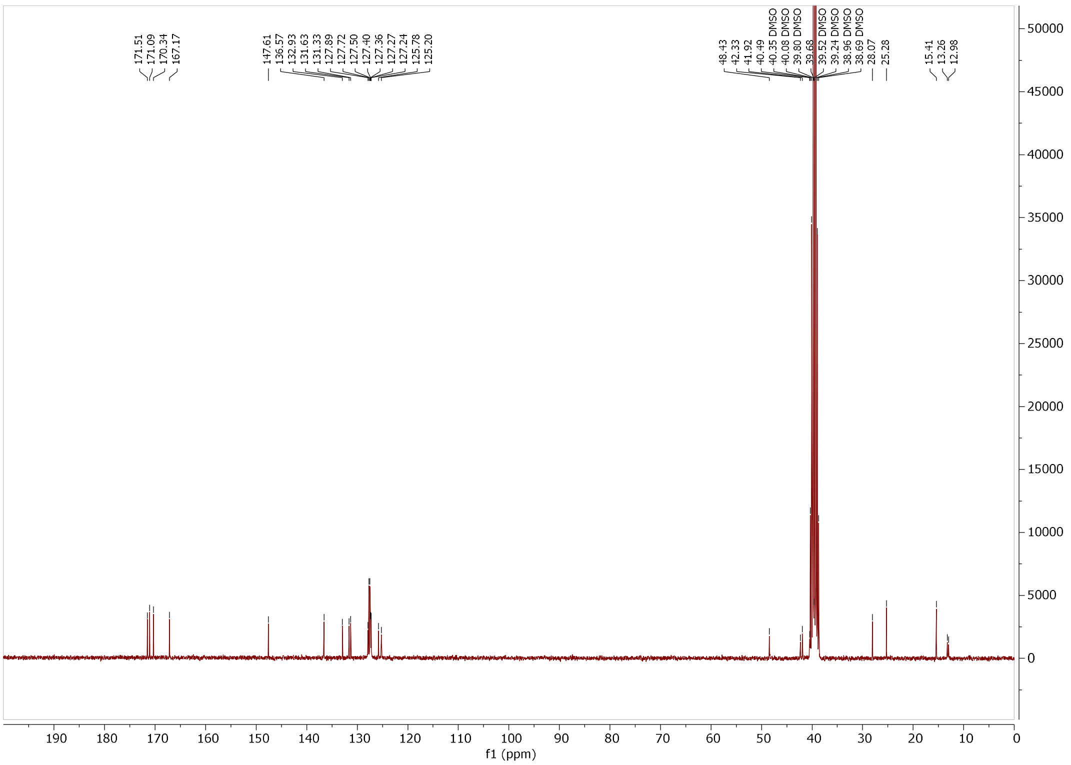

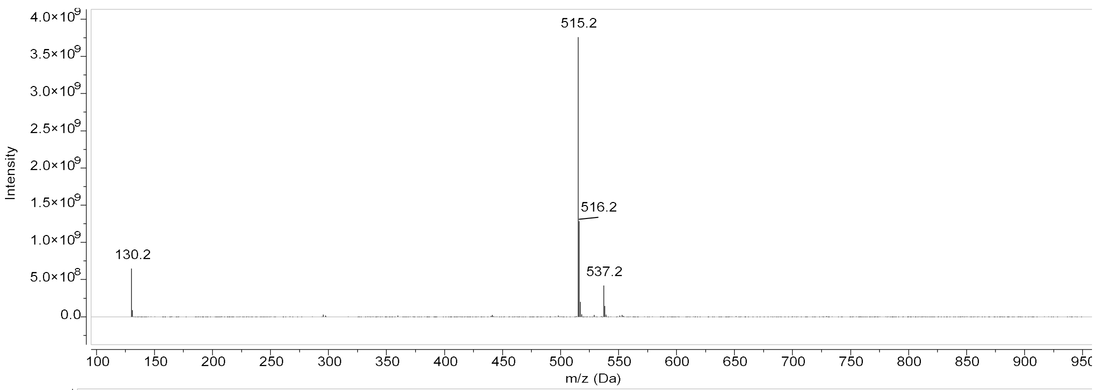

Intensity
m/z (Da)
130.2
515.2
516.2
537.2

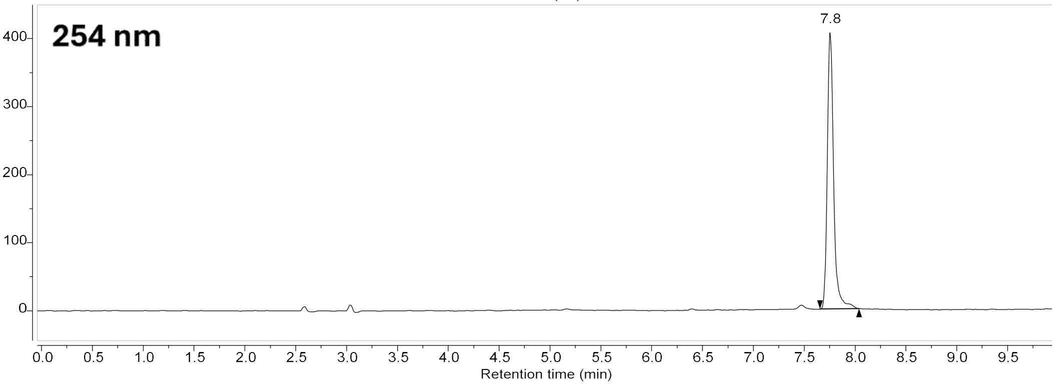

254 nm
7.8
Retention time (min)

Compound 37:

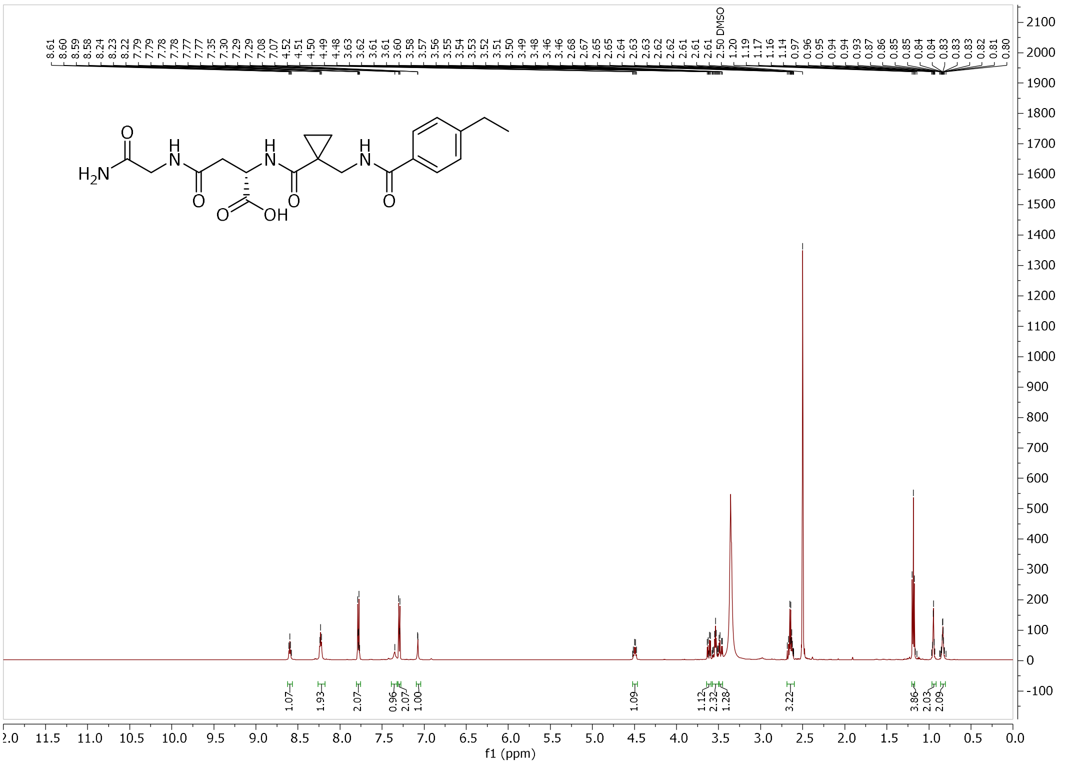
8.61 8.60 8.59 8.58 8.24 8.23 8.22 7.79 7.79 7.78 7.78 7.77 7.35 7.30 7.29 7.29 7.08 7.07 4.52 4.51 4.50 4.49 4.48 3.63 3.62 3.61 3.61 3.60 3.58 3.57 3.56 3.55 3.54 3.53 3.52 3.51 3.50 3.49 3.48 3.46 3.46 2.68 2.67 2.65 2.65 2.64 2.63 2.63 2.62 2.62 2.61 2.61 2.61 2.50 DMSO 1.20 1.19 1.17 1.16 1.14 0.97 0.96 0.95 0.94 0.94 0.93 0.87 0.86 0.85 0.85 0.84 0.84 0.83 0.83 0.83 0.82 0.81 0.80
1.07 1.93 2.07 0.96 2.07 1.00 1.09 1.12 2.32 1.28 3.22 3.86 2.03 2.09
f1 (ppm)

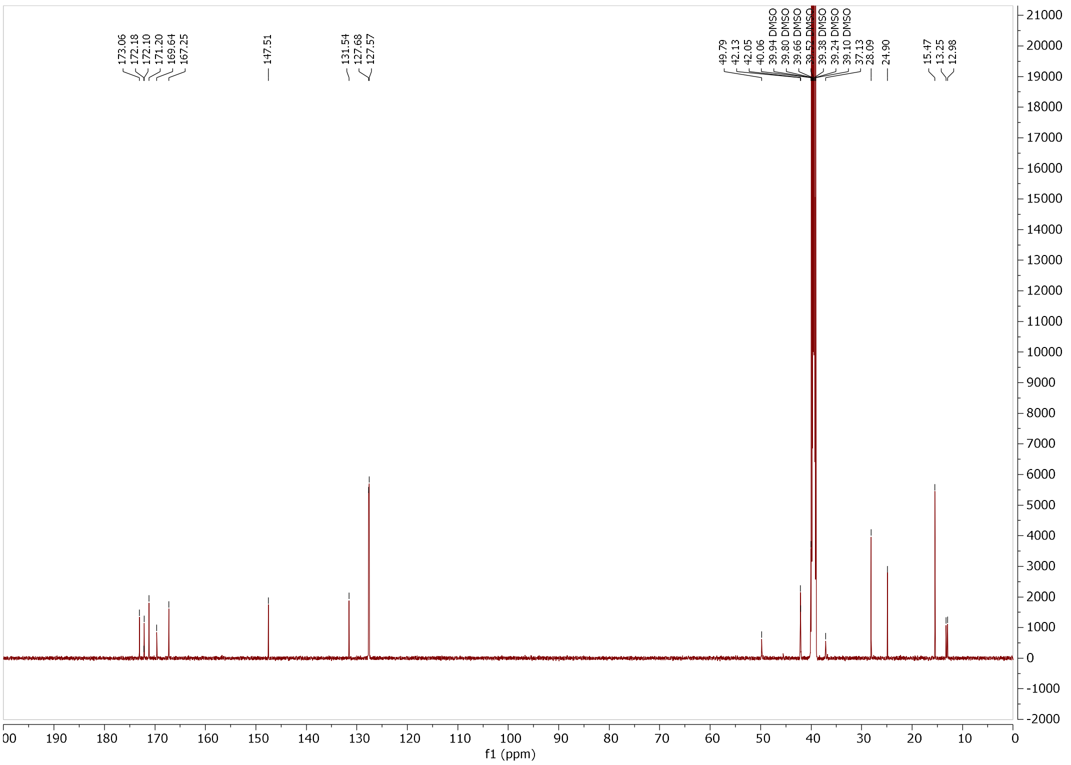
173.06 172.18 172.10 171.20 169.64 167.25
147.51
131.54 127.68 127.57
49.79 42.13 42.05 40.06 39.94 DMSO 39.80 DMSO 39.66 DMSO 39.52 DMSO 39.38 DMSO 39.24 DMSO 39.10 DMSO 37.13 28.09
24.90
15.47 13.25 12.98
f1 (ppm)

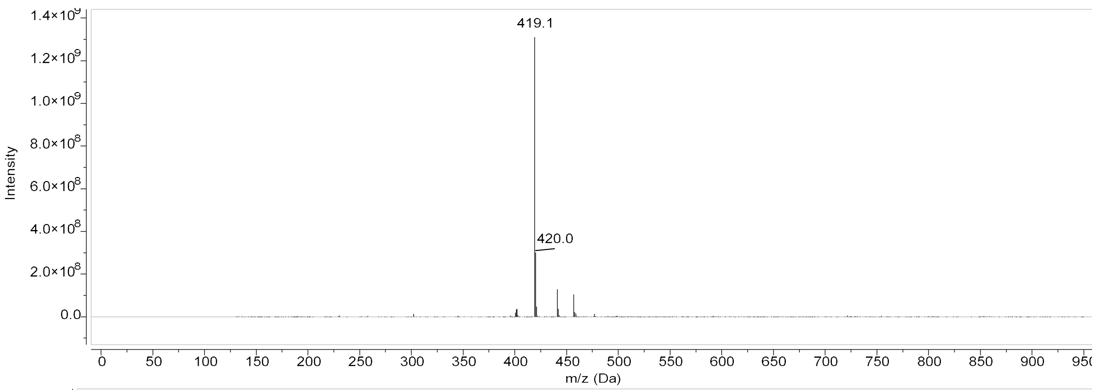
419.1
420.0
Intensity
m/z (Da)

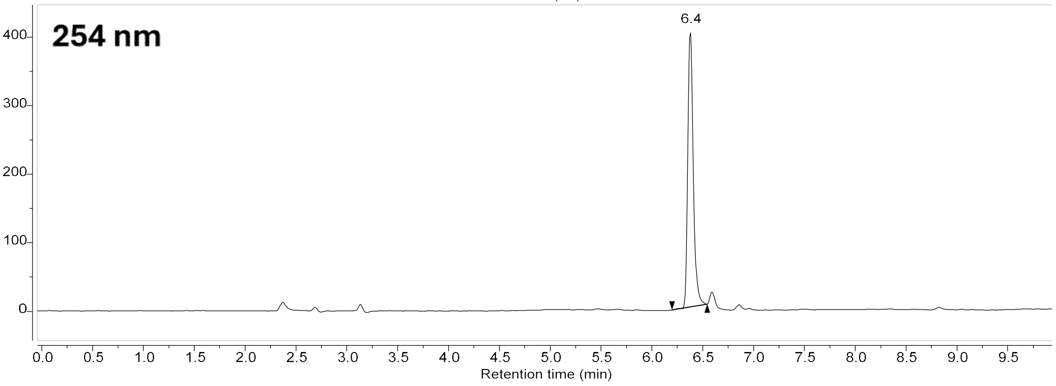
254 nm
6.4
Retention time (min)

Compound 38:

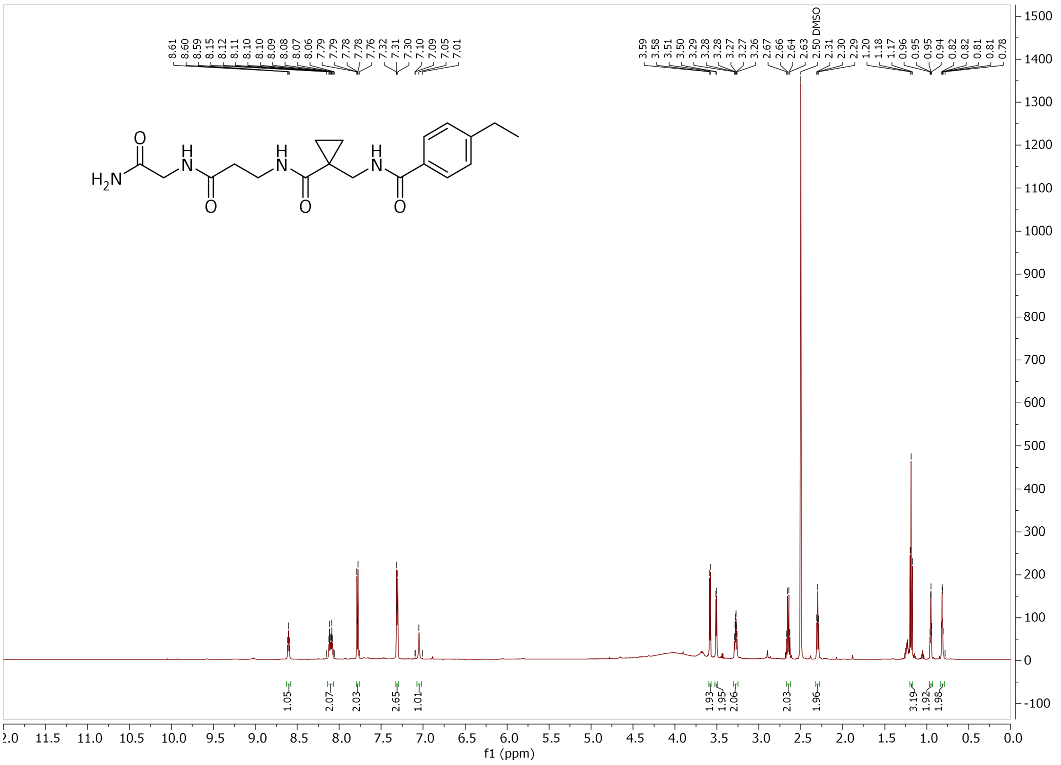

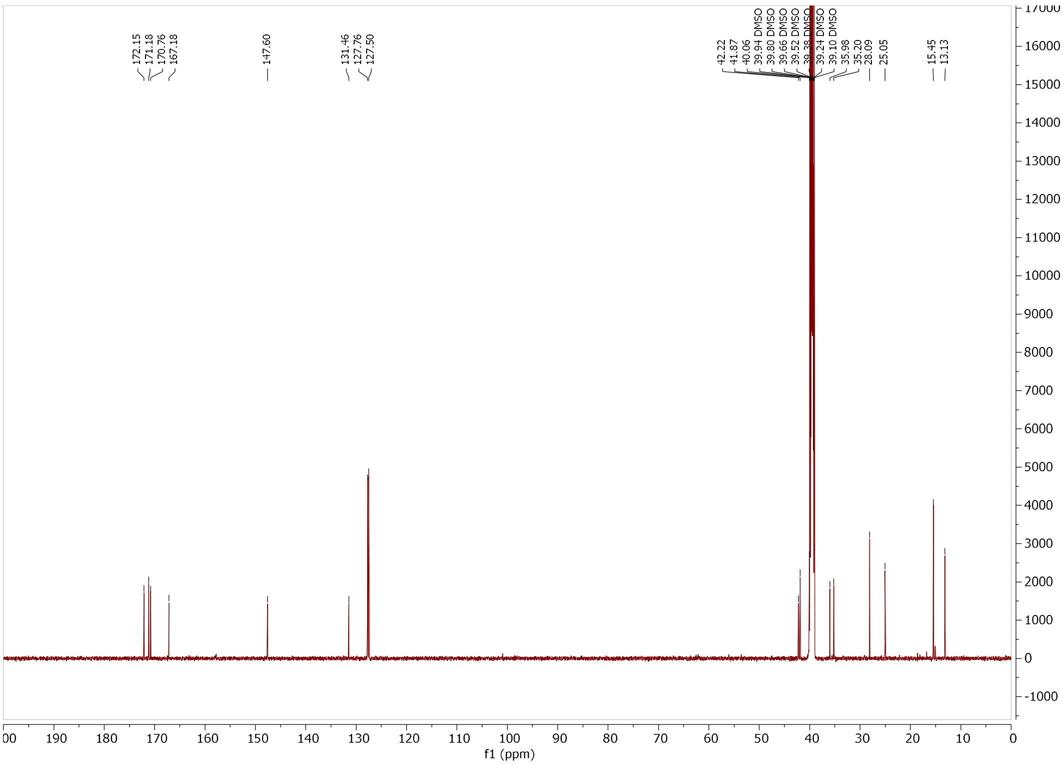

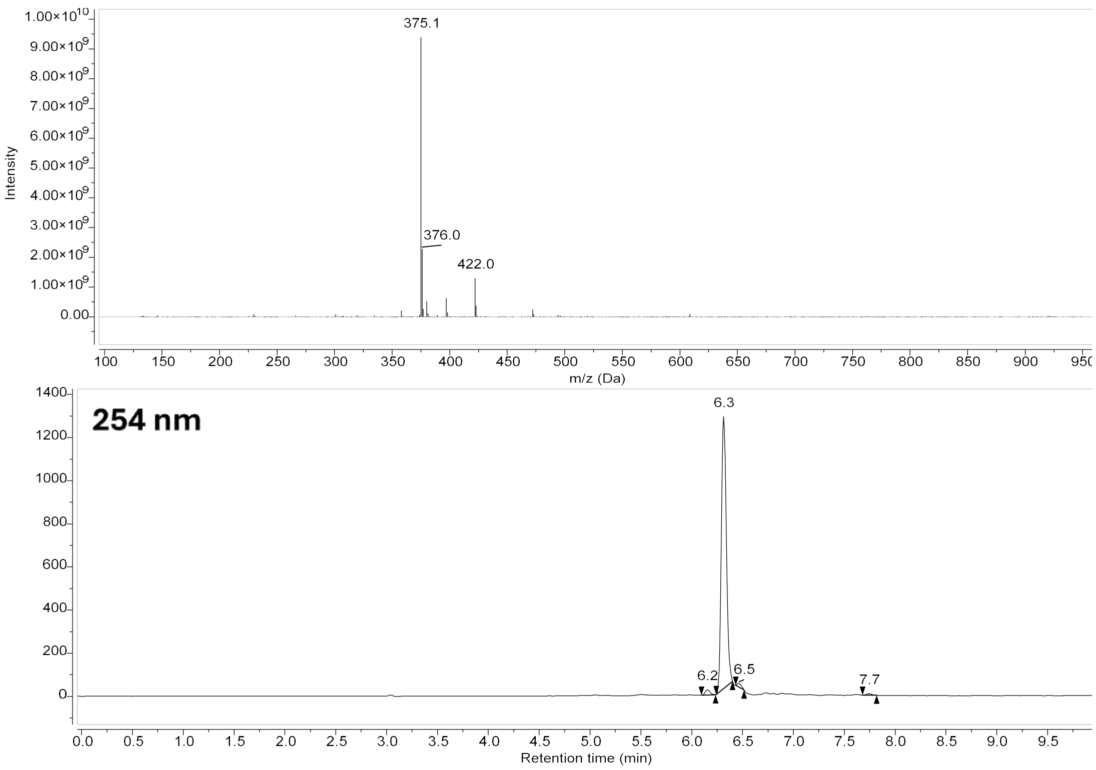

375.1
376.0
422.0
Intensity
1.00×10^10
9.00×10^9
8.00×10^9
7.00×10^9
6.00×10^9
5.00×10^9
4.00×10^9
3.00×10^9
2.00×10^9
1.00×10^9
0.00
100 150 200 250 300 350 400 450 500 550 600 650 700 750 800 850 900 950
m/z (Da)
254 nm
6.3
6.2
6.5
7.7
1400
1200
1000
800
600
400
200
0
0.0 0.5 1.0 1.5 2.0 2.5 3.0 3.5 4.0 4.5 5.0 5.5 6.0 6.5 7.0 7.5 8.0 8.5 9.0 9.5
Retention time (min)

Compound 16:

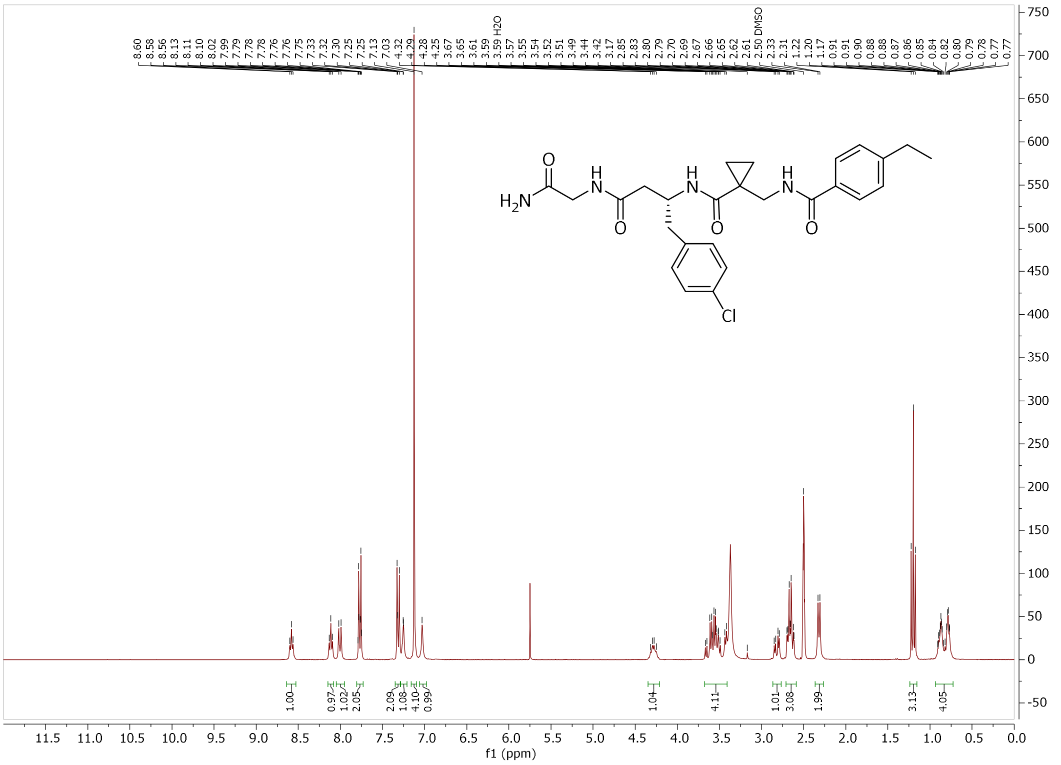

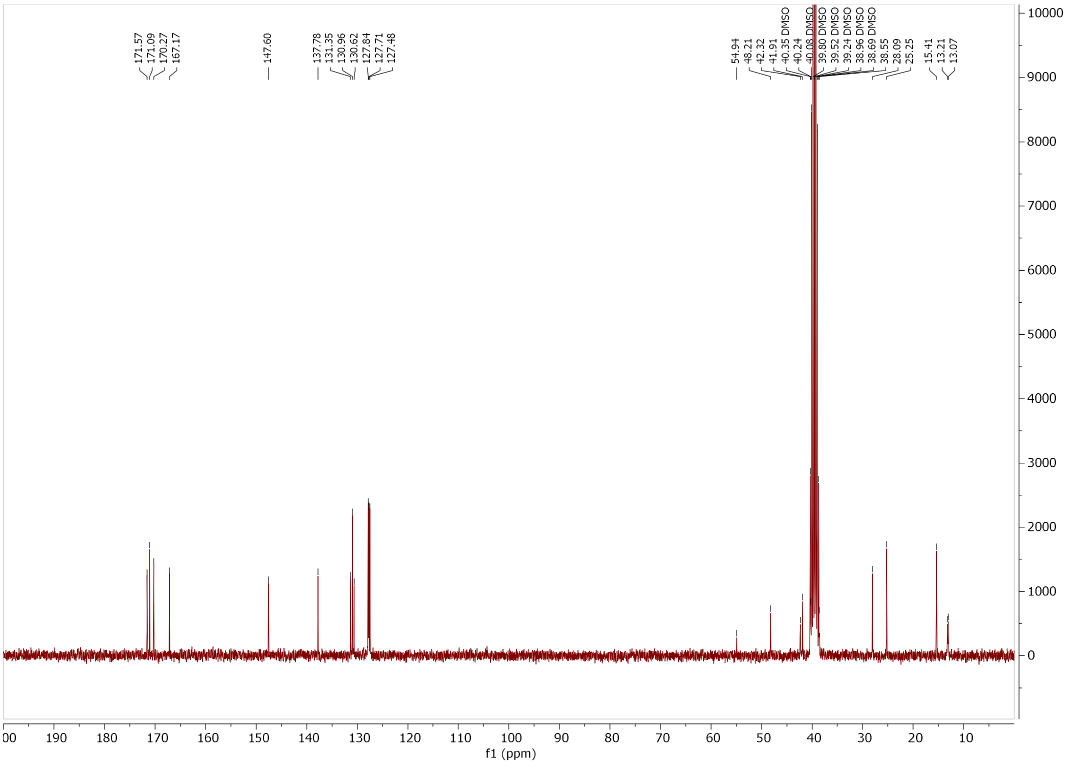

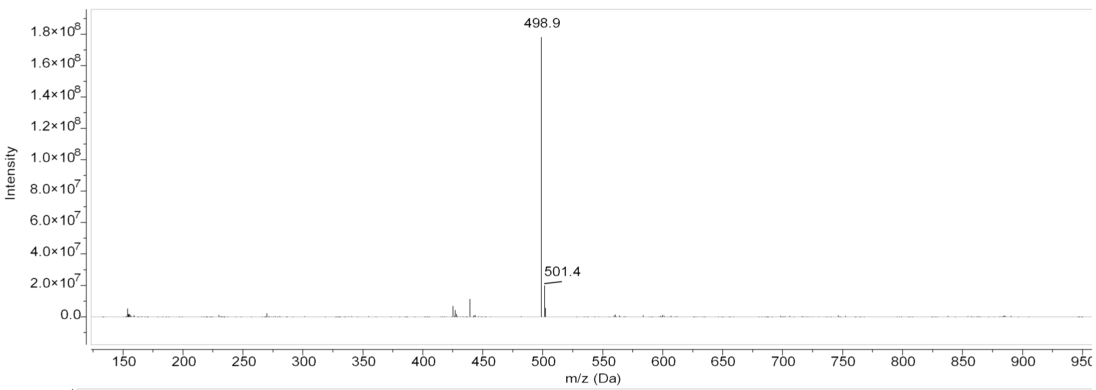
498.9
501.4
Intensity
m/z (Da)

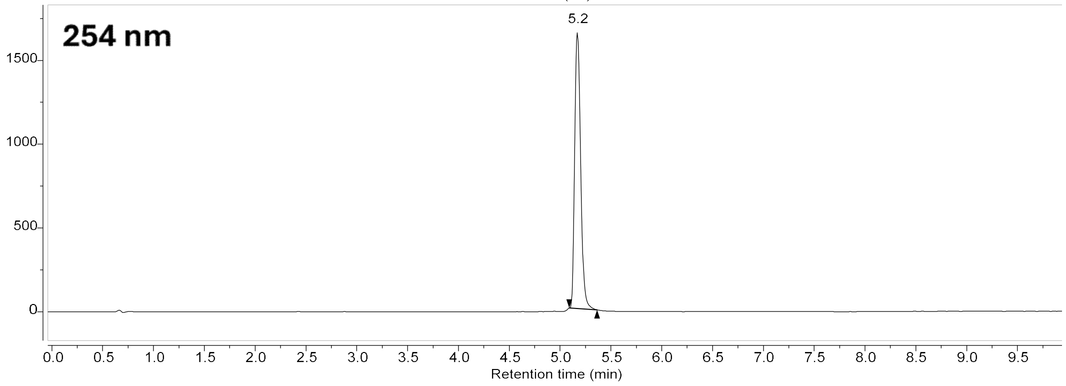
254 nm
5.2
Retention time (min)

Compound 17:

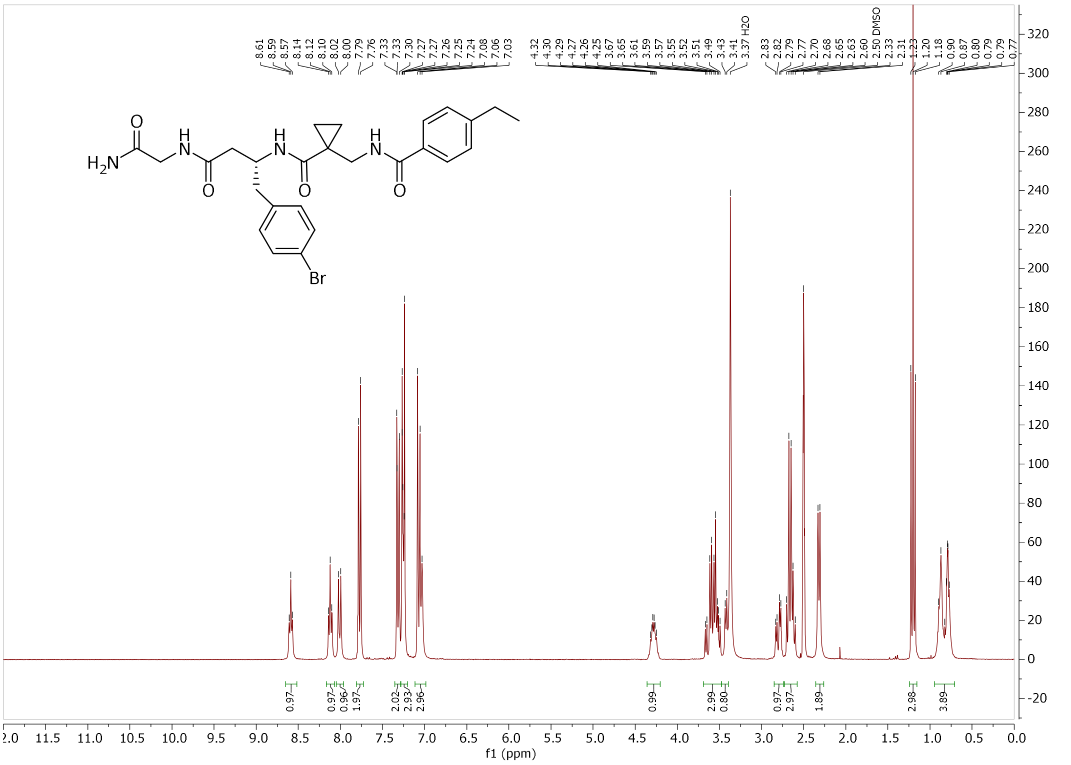

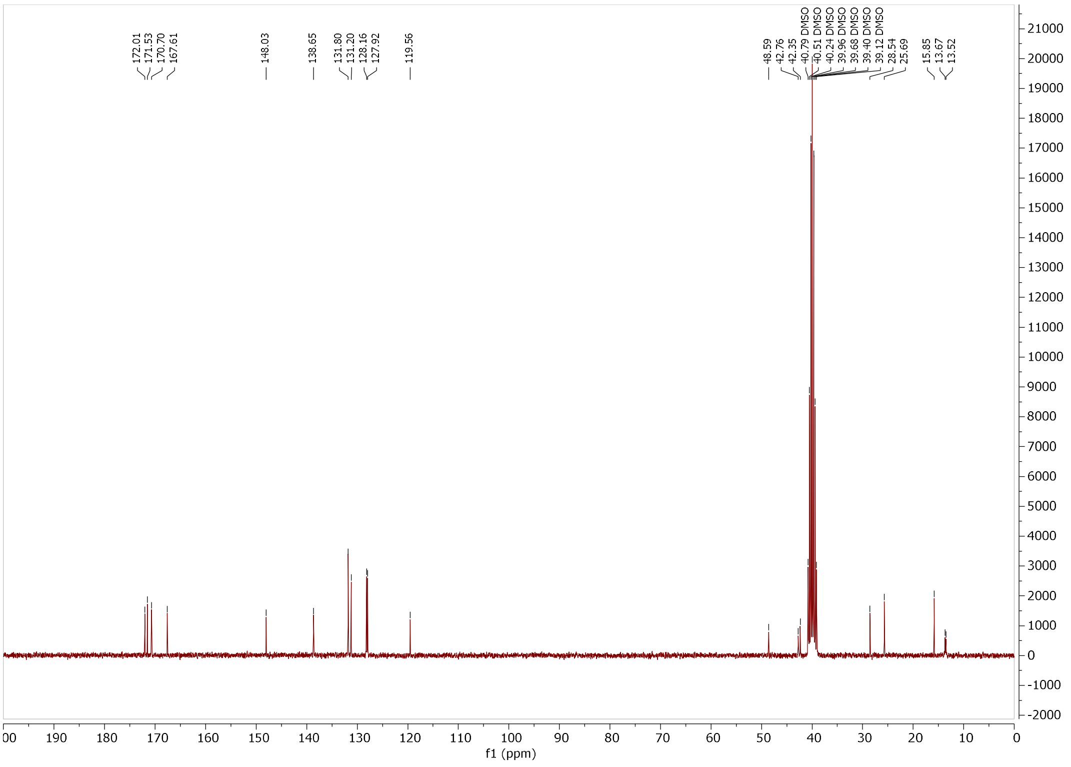

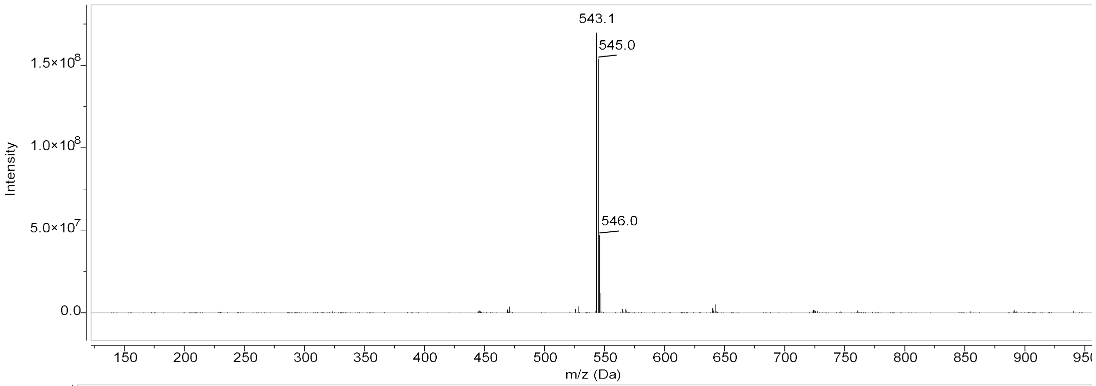

543.1
545.0
546.0
1.5×10^8
1.0×10^8
5.0×10^7
0.0
Intensity
150 200 250 300 350 400 450 500 550 600 650 700 750 800 850 900 950
m/z (Da)

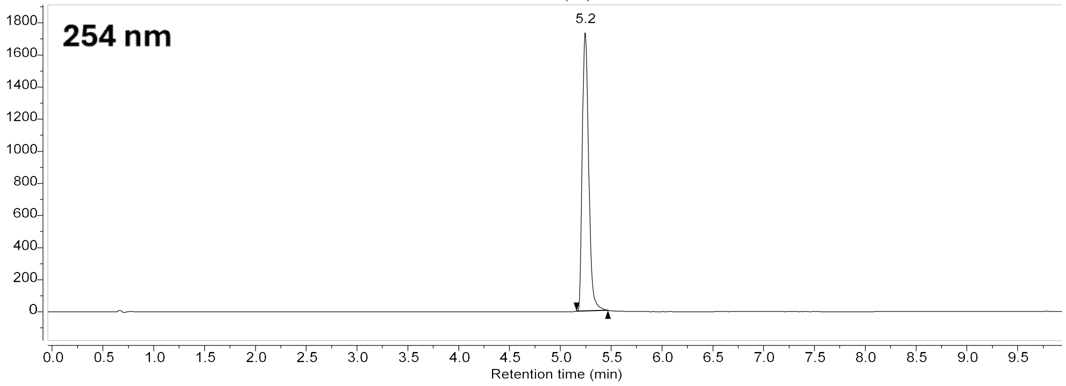

254 nm
5.2
1800 1600 1400 1200 1000 800 600 400 200 0
0.0 0.5 1.0 1.5 2.0 2.5 3.0 3.5 4.0 4.5 5.0 5.5 6.0 6.5 7.0 7.5 8.0 8.5 9.0 9.5
Retention time (min)

Compound 33:

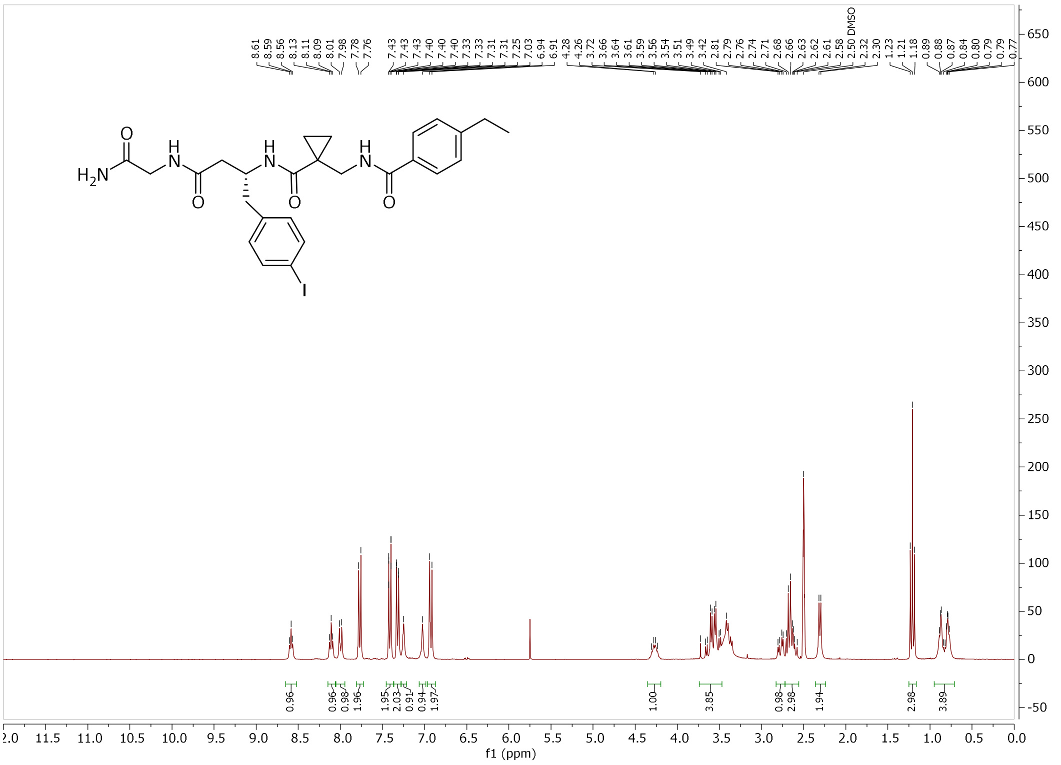

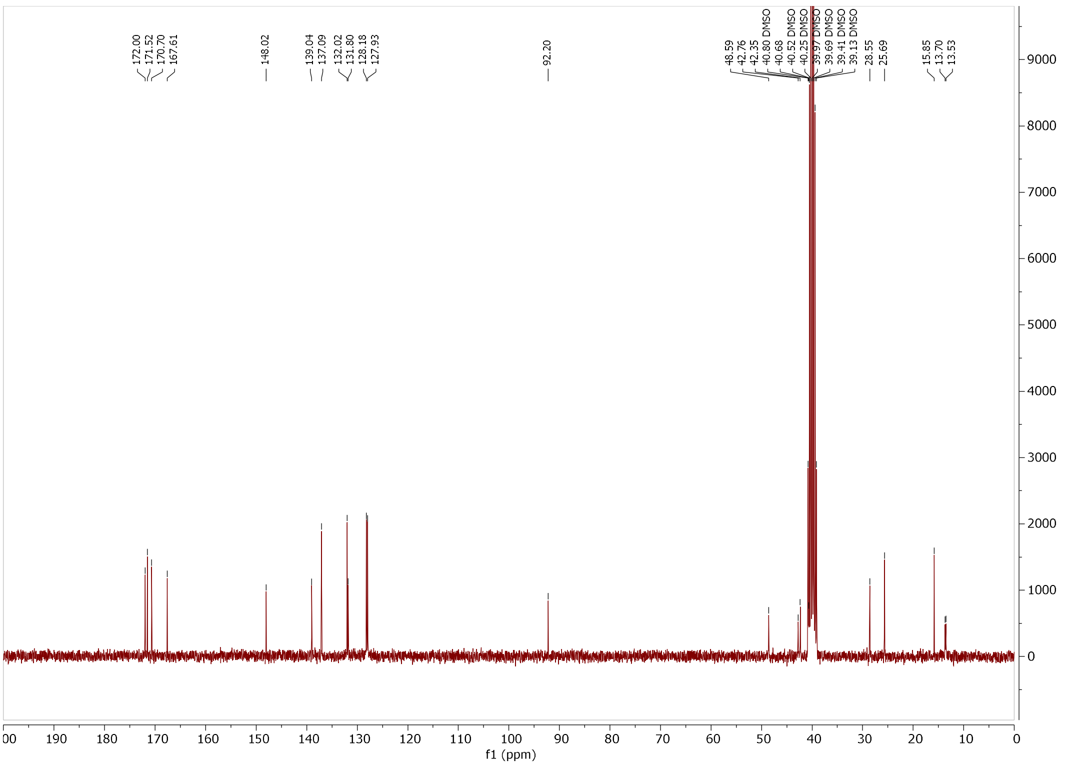

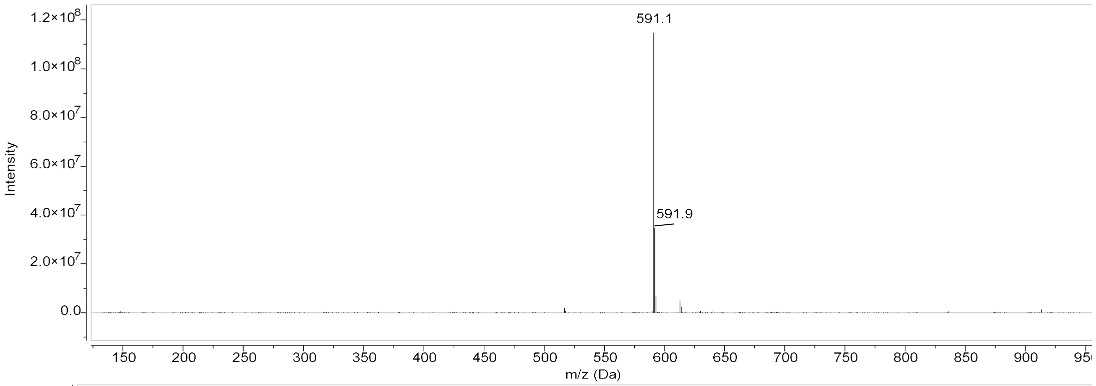
591.1
591.9
Intensity
m/z (Da)

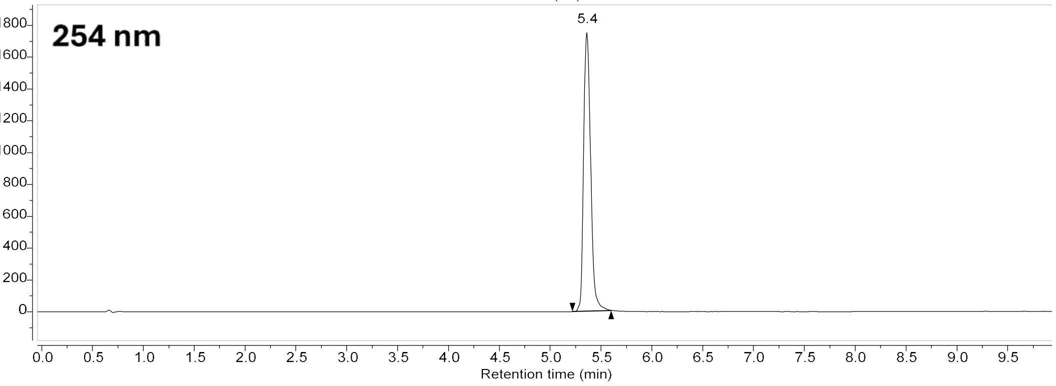
254 nm
5.4
Retention time (min)

Compound 34:

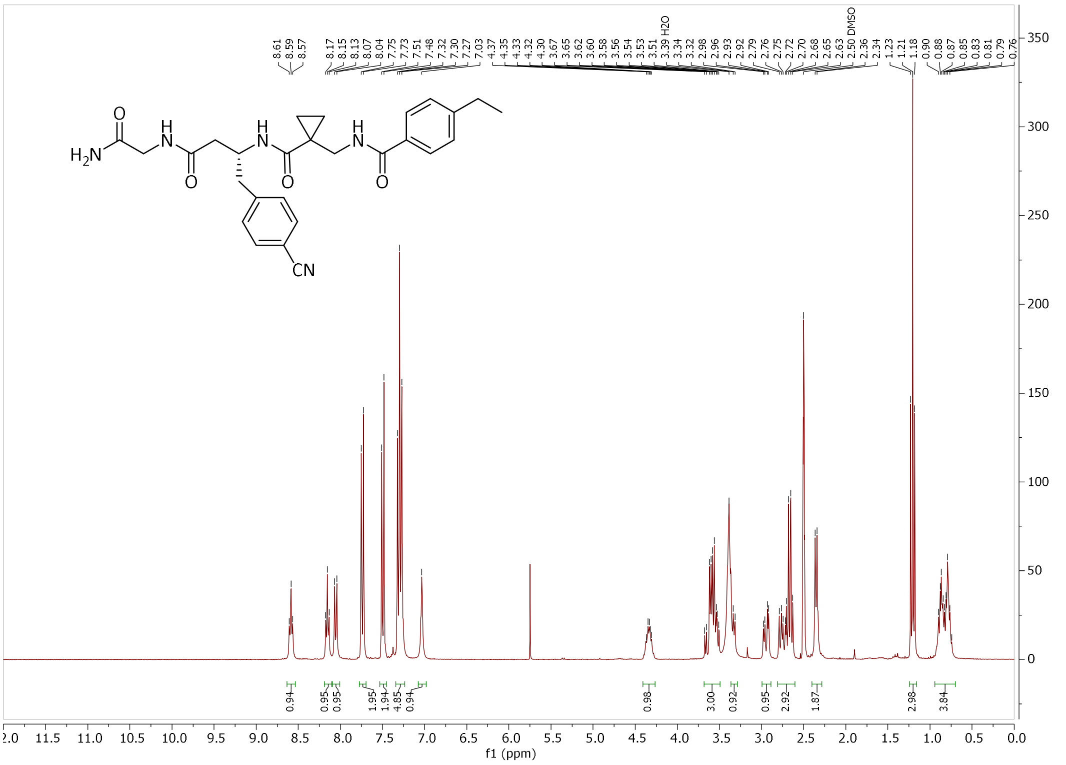

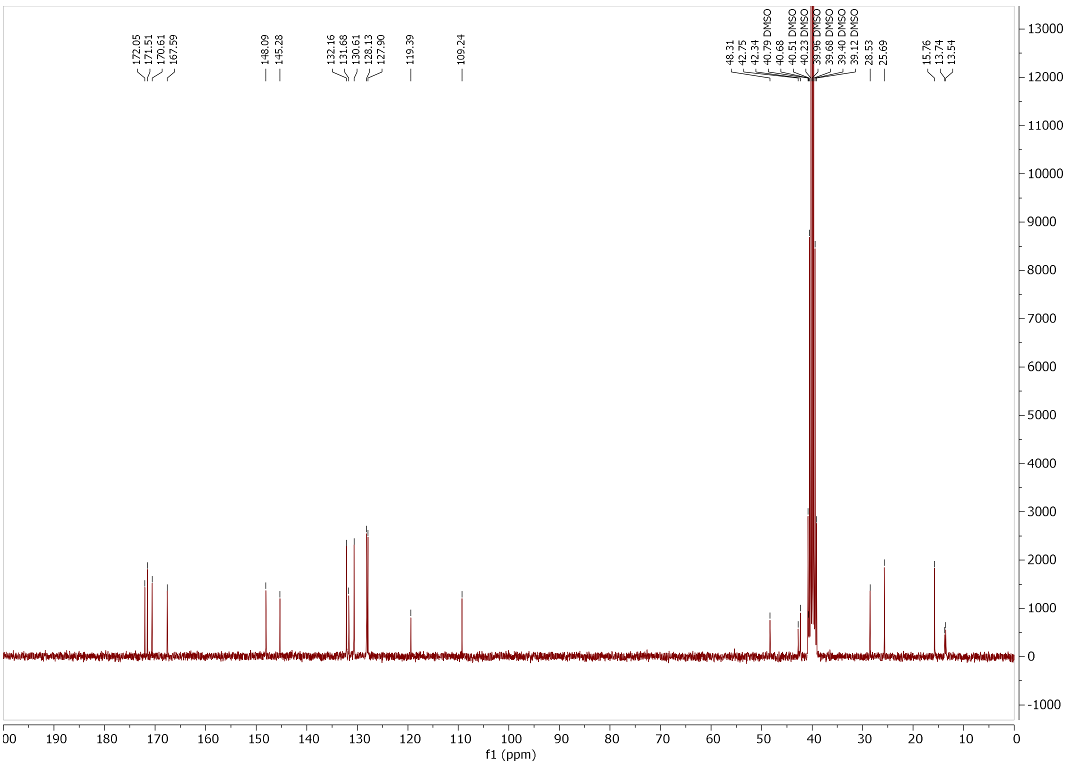

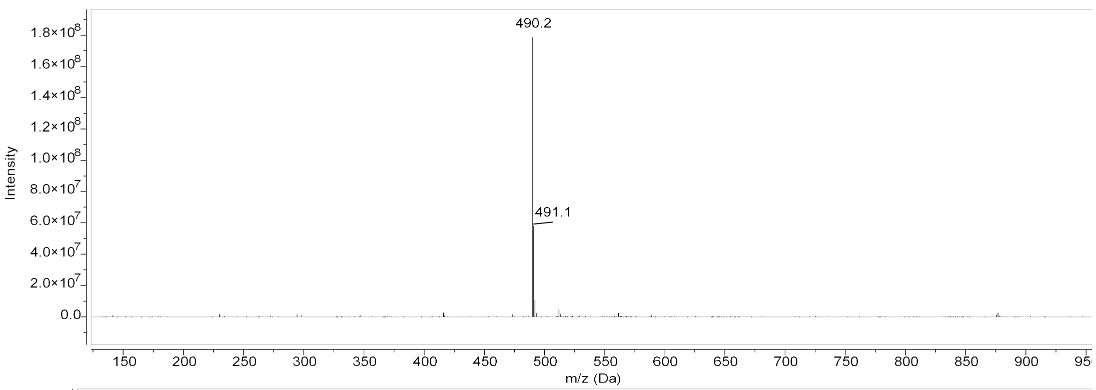
490.2
491.1
Intensity
1.8×10^8
1.6×10^8
1.4×10^8
1.2×10^8
1.0×10^8
8.0×10^7
6.0×10^7
4.0×10^7
2.0×10^7
0.0
150 200 250 300 350 400 450 500 550 600 650 700 750 800 850 900 950
m/z (Da)

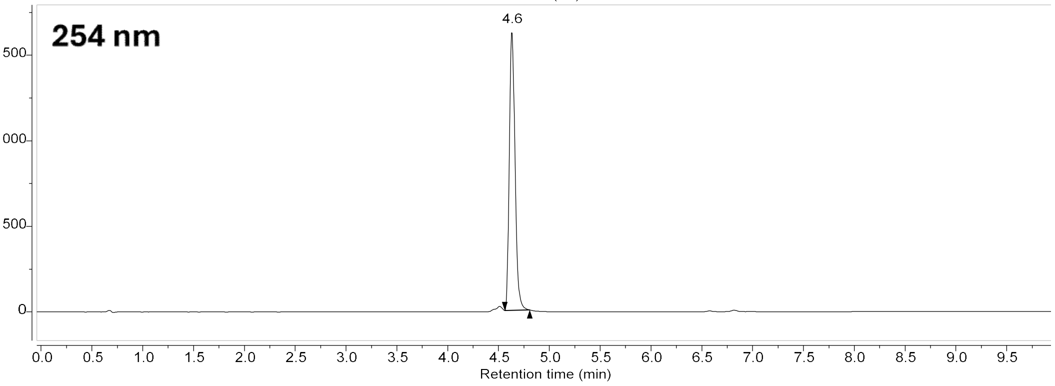
254 nm
4.6
1500
1000
500
0
0.0 0.5 1.0 1.5 2.0 2.5 3.0 3.5 4.0 4.5 5.0 5.5 6.0 6.5 7.0 7.5 8.0 8.5 9.0 9.5
Retention time (min)

Compound 39:

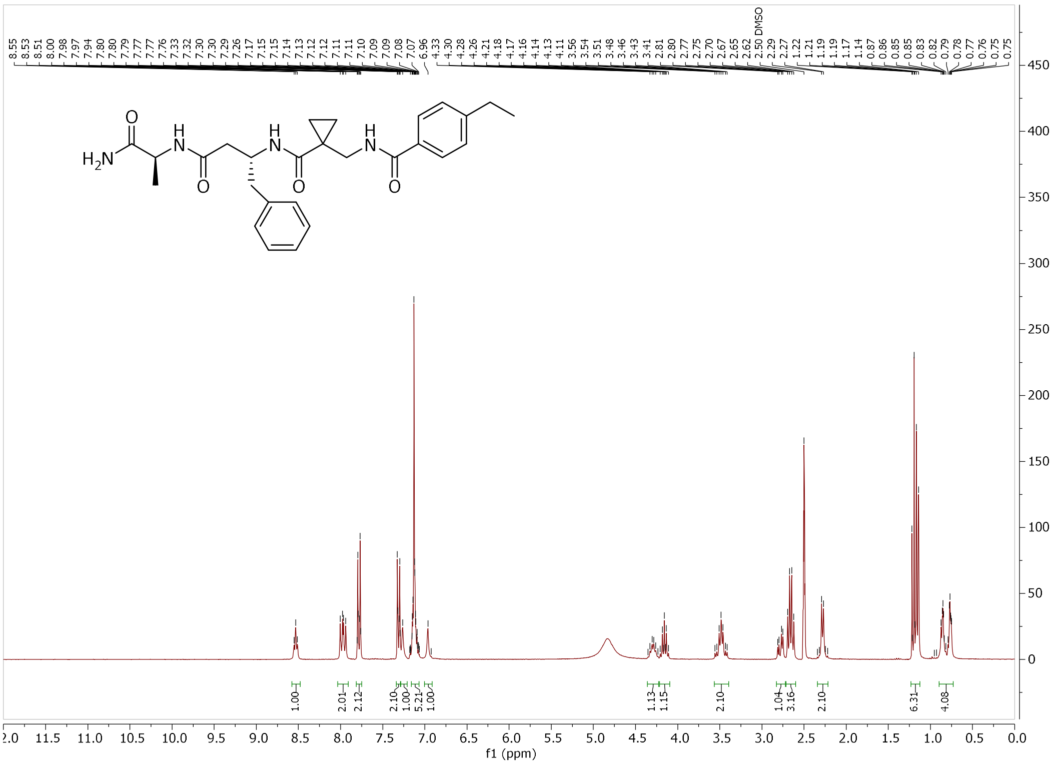

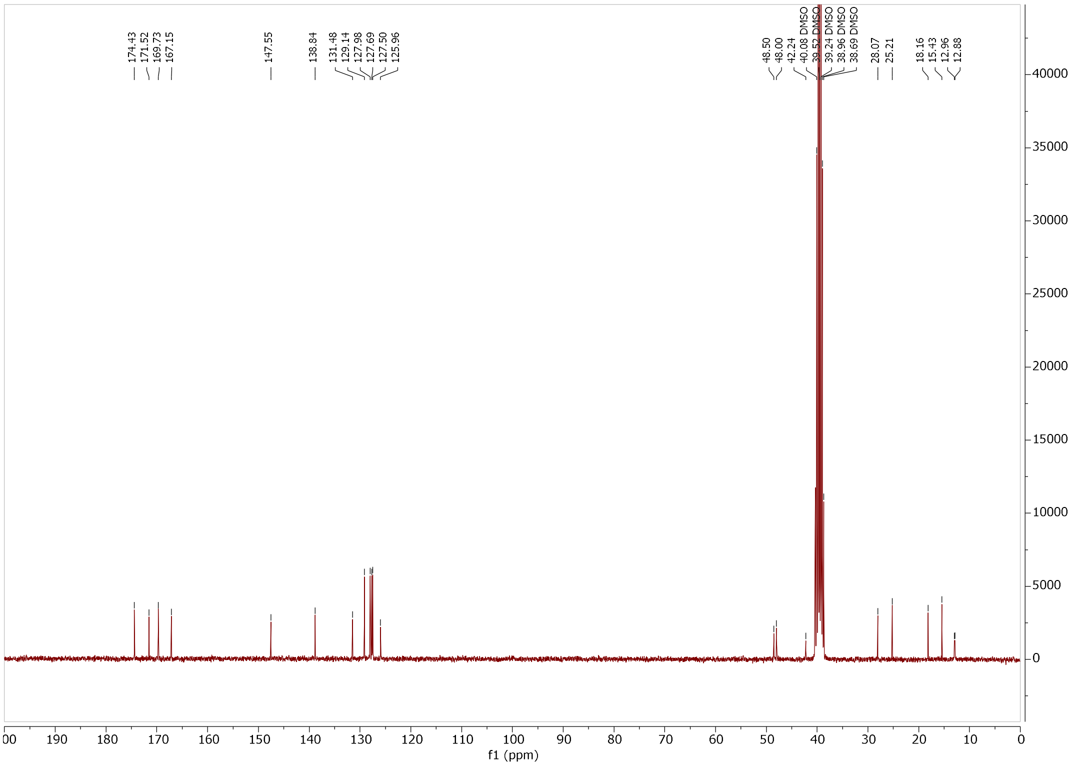

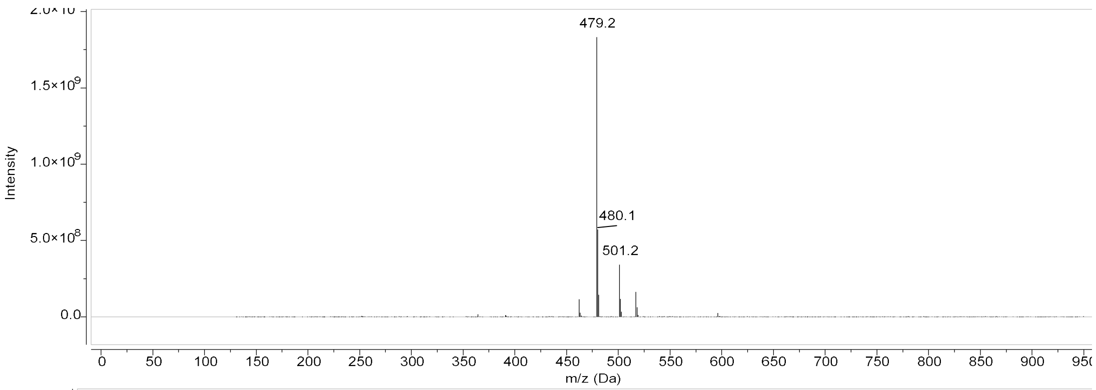
479.2
480.1
501.2
Intensity
m/z (Da)

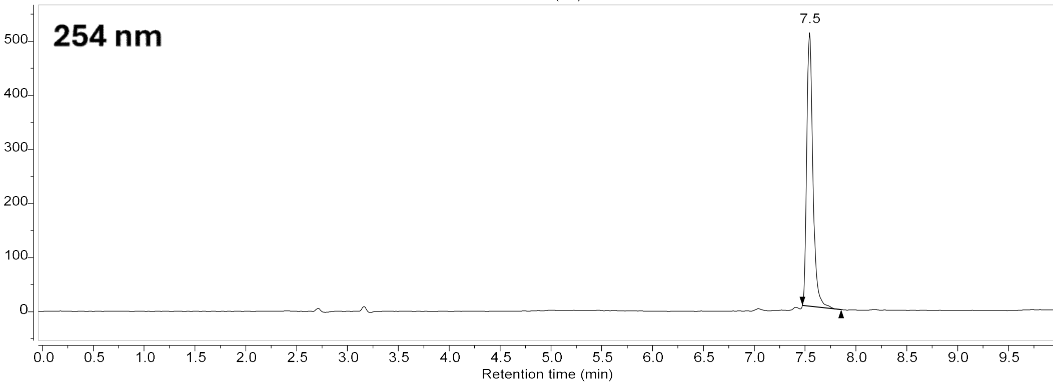
254 nm
7.5
Retention time (min)

Compound 40:

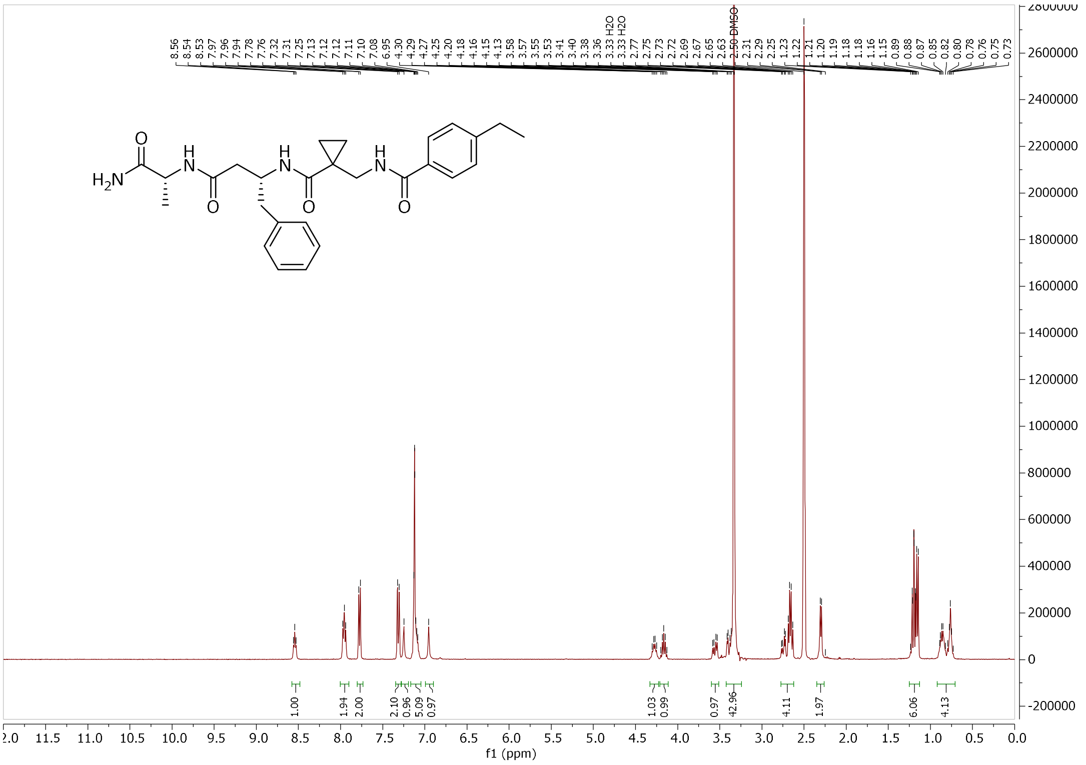

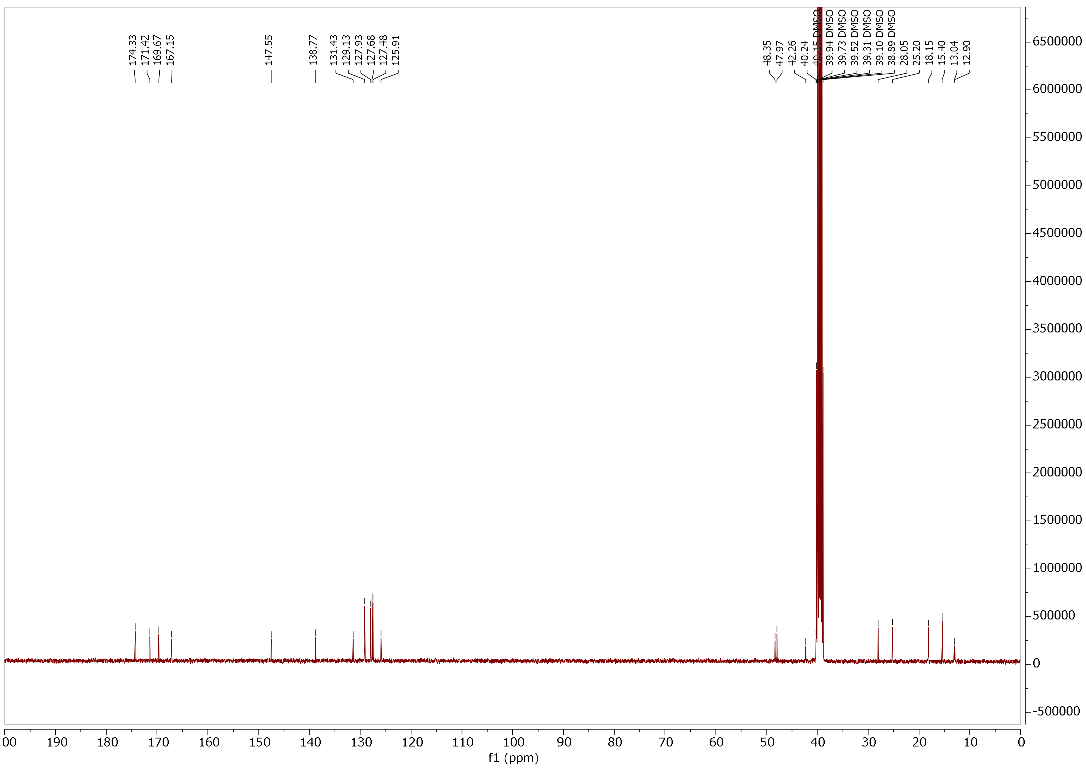

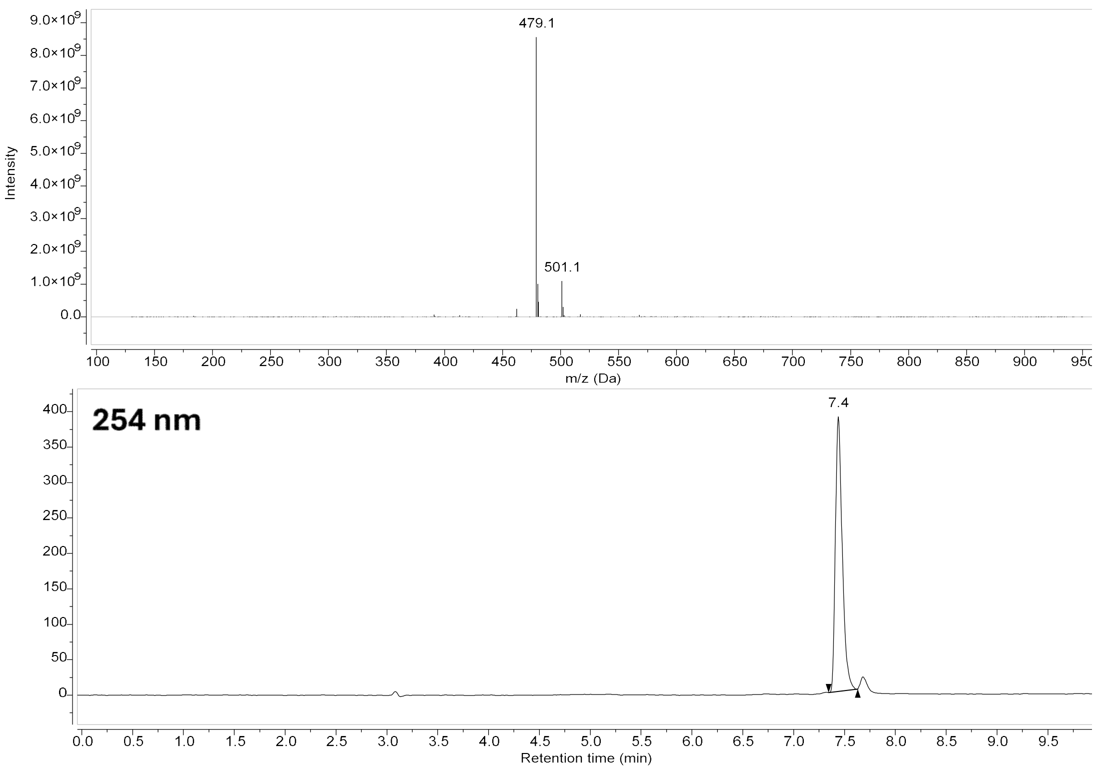

479.1
501.1
Intensity
m/z (Da)
254 nm
7.4
Retention time (min)

Compound 41:

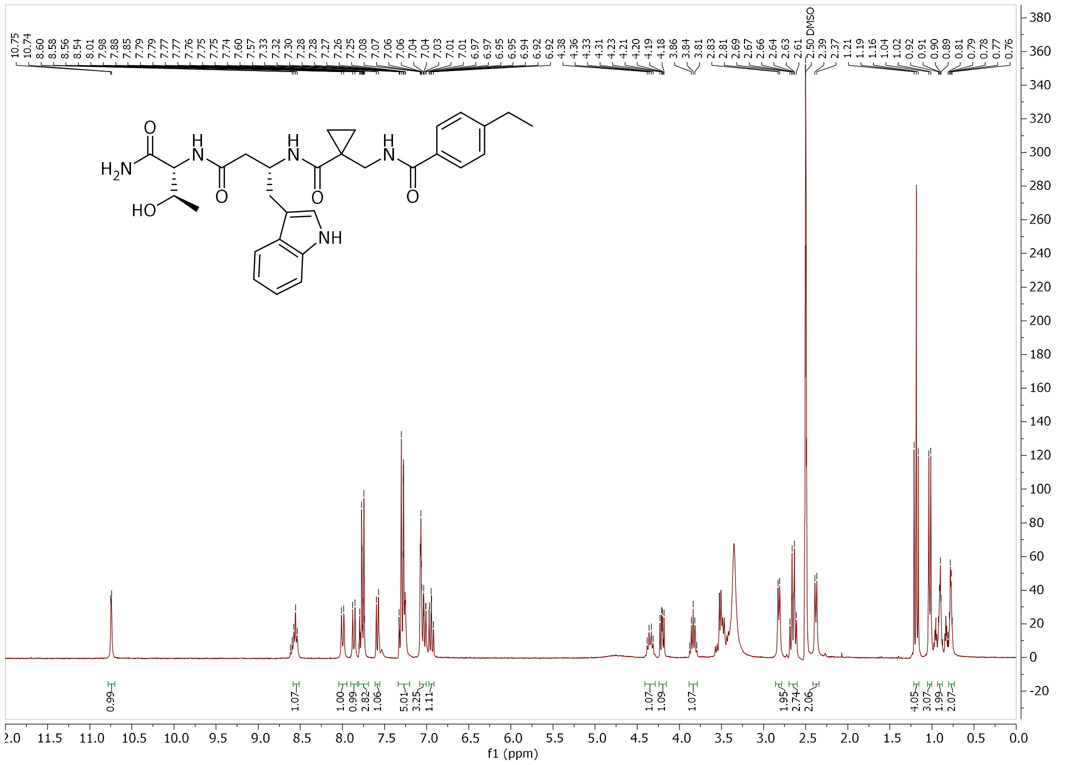

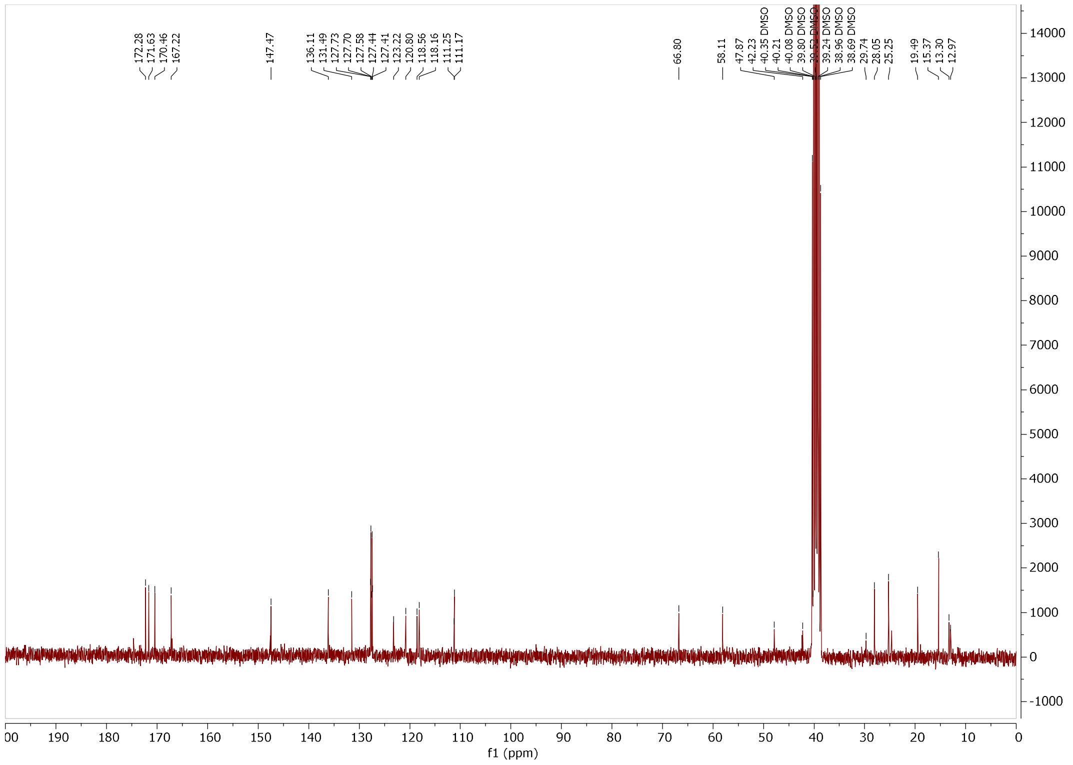

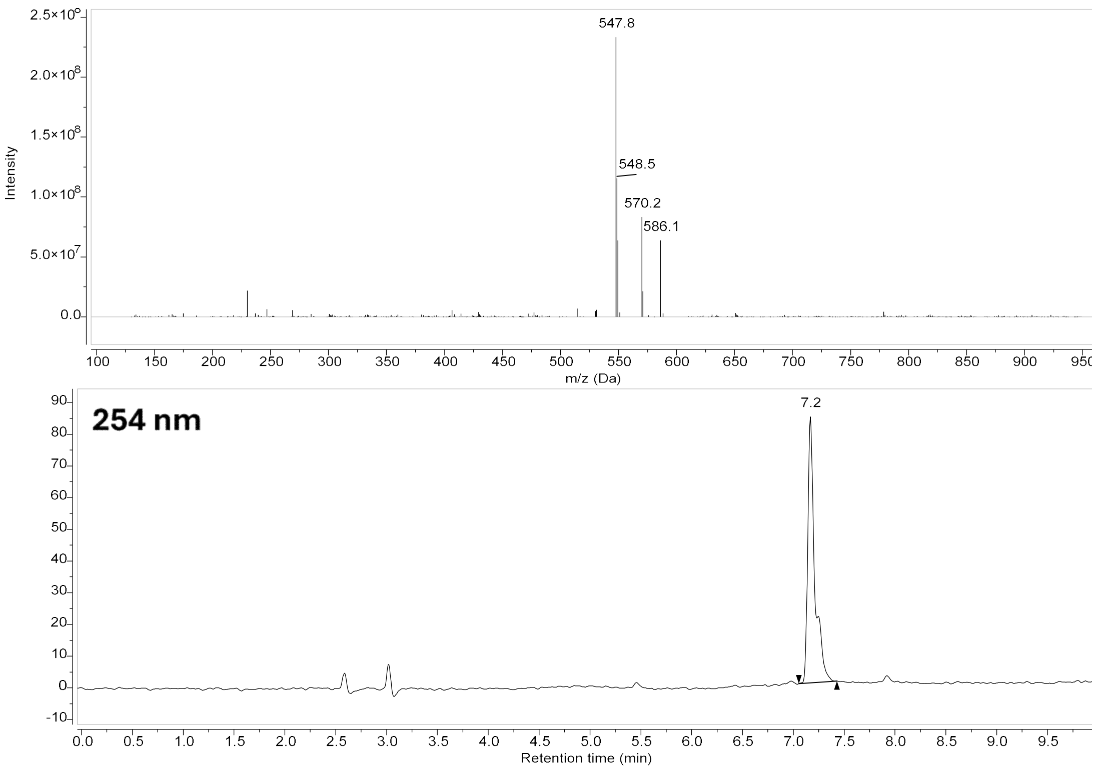

Intensity
m/z (Da)
547.8
548.5
570.2
586.1
254 nm
7.2
Retention time (min)

Compound 42:

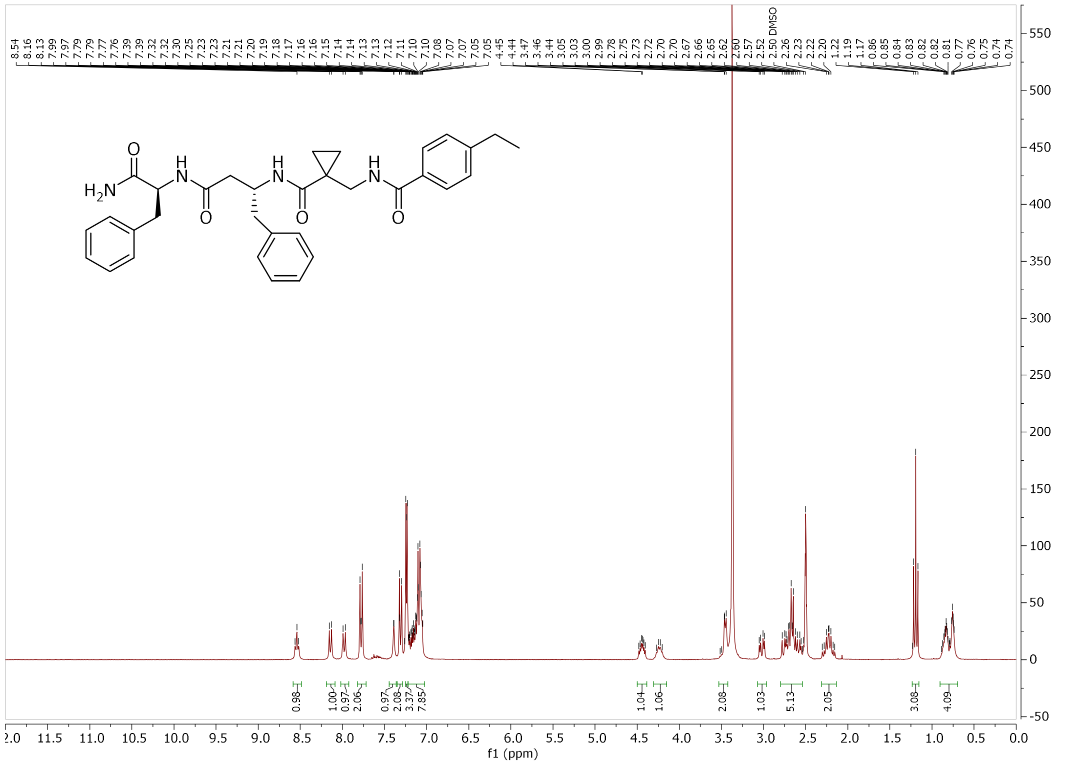

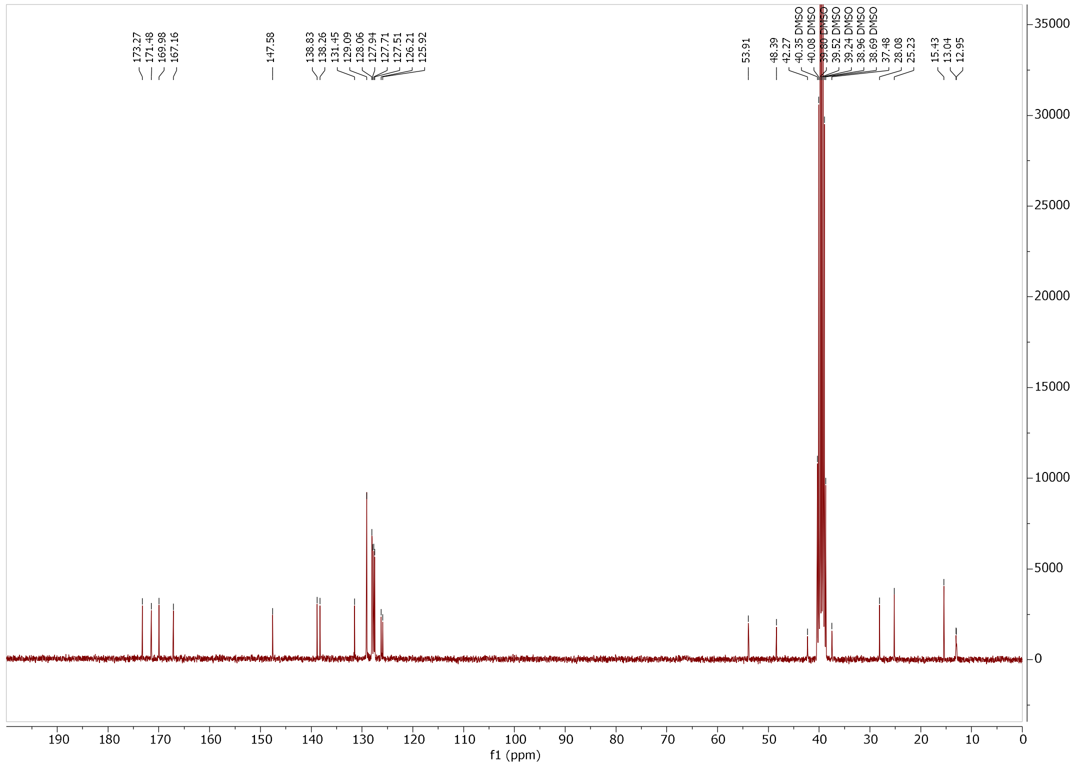

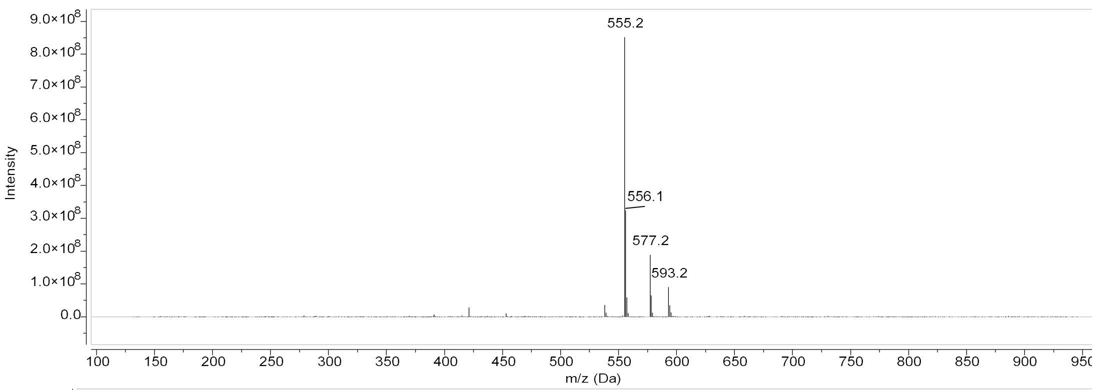
555.2
556.1
577.2
593.2
Intensity
m/z (Da)
9.0×10^8
8.0×10^8
7.0×10^8
6.0×10^8
5.0×10^8
4.0×10^8
3.0×10^8
2.0×10^8
1.0×10^8
0.0
100 150 200 250 300 350 400 450 500 550 600 650 700 750 800 850 900 950

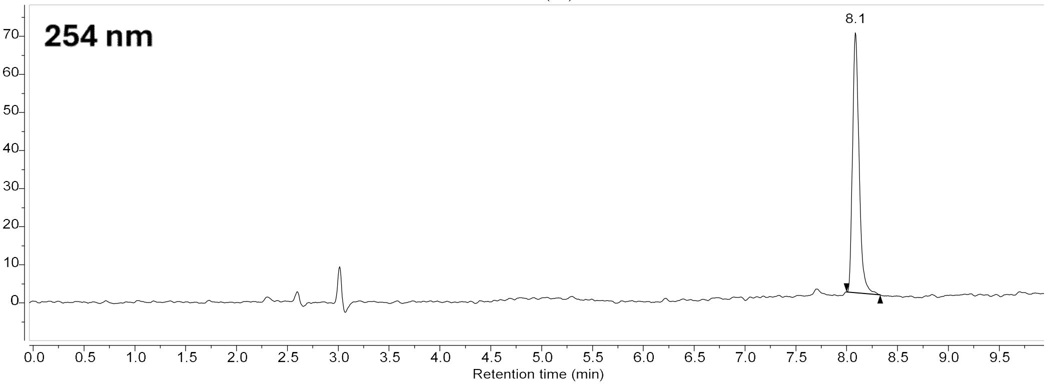
254 nm
8.1
70
60
50
40
30
20
10
0
0.0 0.5 1.0 1.5 2.0 2.5 3.0 3.5 4.0 4.5 5.0 5.5 6.0 6.5 7.0 7.5 8.0 8.5 9.0 9.5
Retention time (min)

Compound 43:

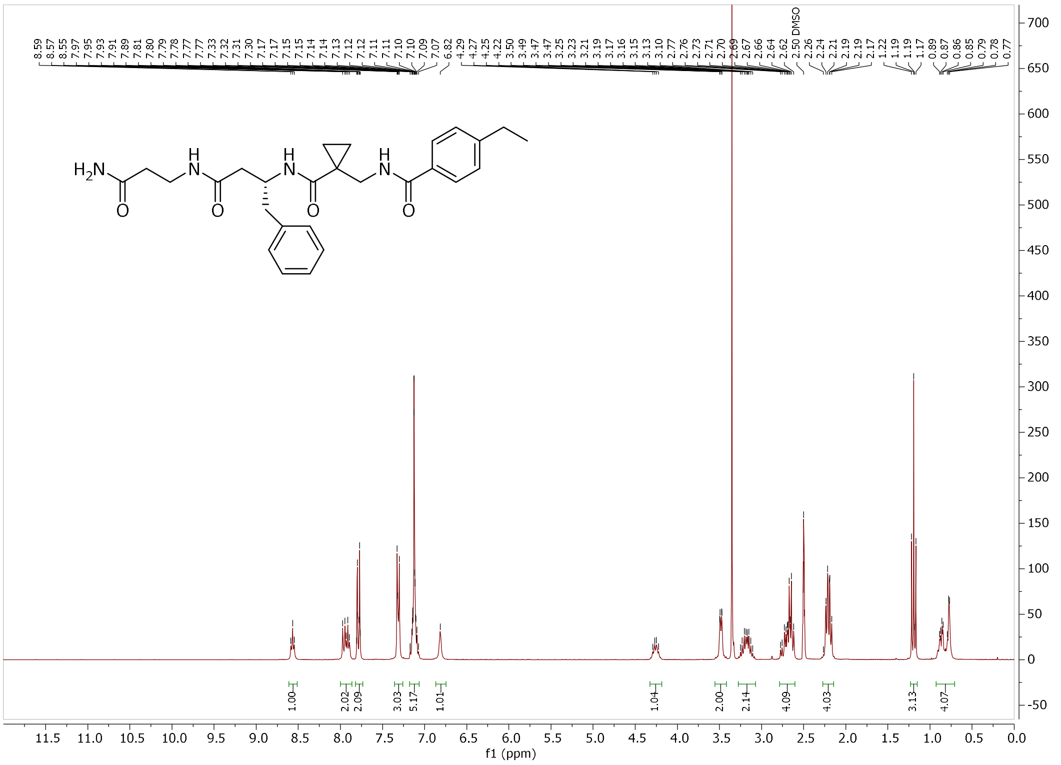

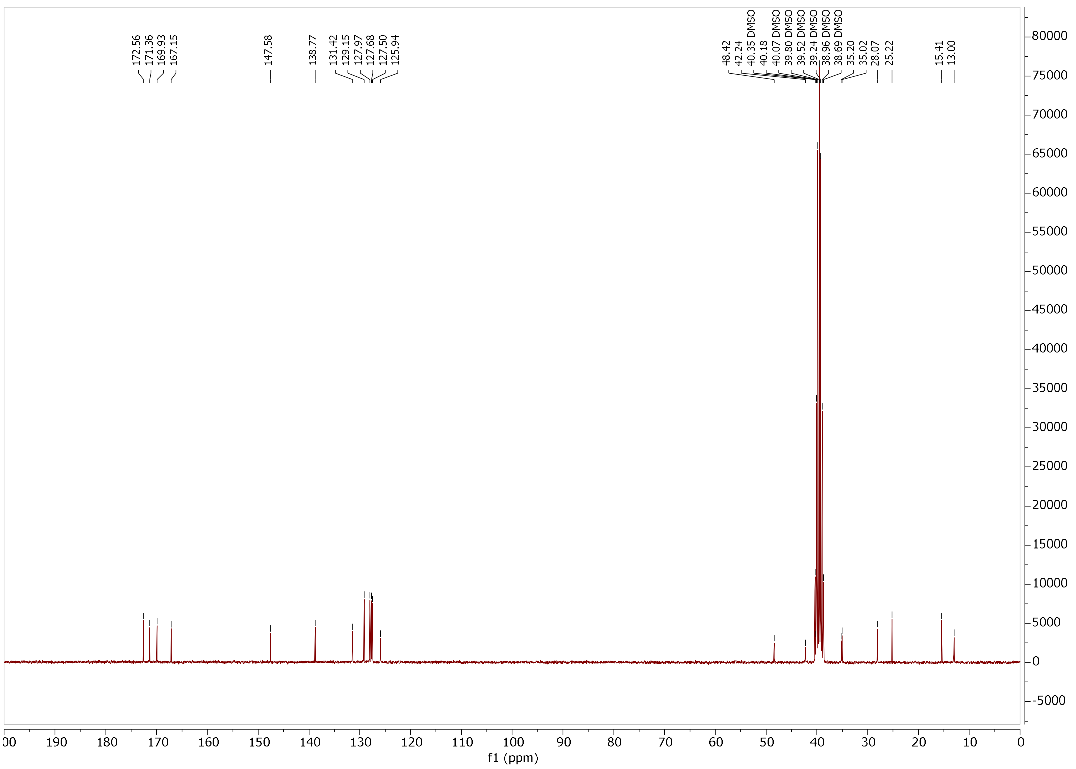

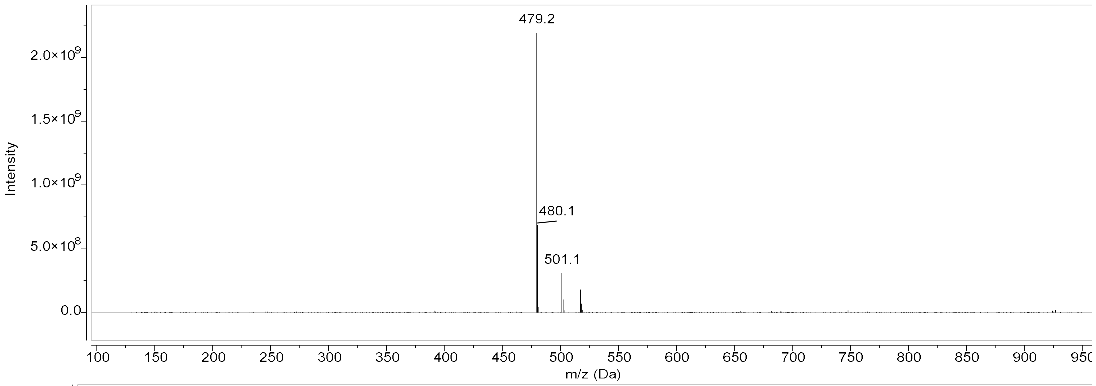

479.2
480.1
501.1
Intensity
2.0×10^9
1.5×10^9
1.0×10^9
5.0×10^8
0.0
100 150 200 250 300 350 400 450 500 550 600 650 700 750 800 850 900 950
m/z (Da)

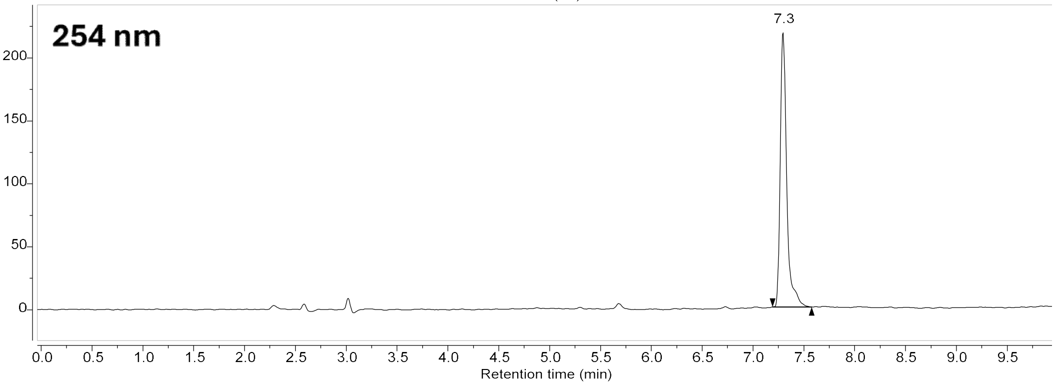

254 nm
7.3
200
150
100
50
0
0.0 0.5 1.0 1.5 2.0 2.5 3.0 3.5 4.0 4.5 5.0 5.5 6.0 6.5 7.0 7.5 8.0 8.5 9.0 9.5
Retention time (min)

Compound 45:

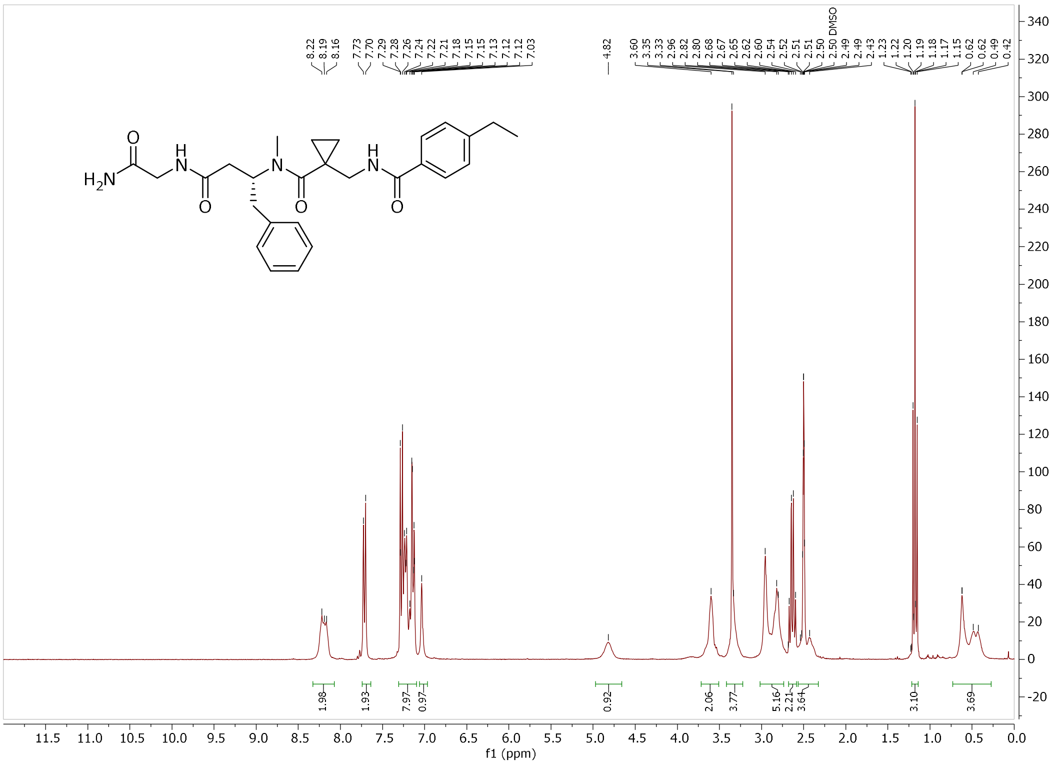

8.22
8.19
8.16
7.73
7.70
7.29
7.28
7.26
7.24
7.22
7.21
7.18
7.15
7.15
7.13
7.12
7.12
7.03
4.82
3.60
3.35
3.33
2.96
2.82
2.80
2.68
2.67
2.65
2.62
2.60
2.54
2.52
2.51
2.51
2.50
2.50 DMSO
2.49
2.49
2.43
1.23
1.22
1.20
1.19
1.18
1.17
1.15
0.62
0.62
0.49
0.42
1.98
1.93
7.97
0.97
0.92
2.06
3.77
5.16
2.21
3.64
3.10
3.69
f1 (ppm)

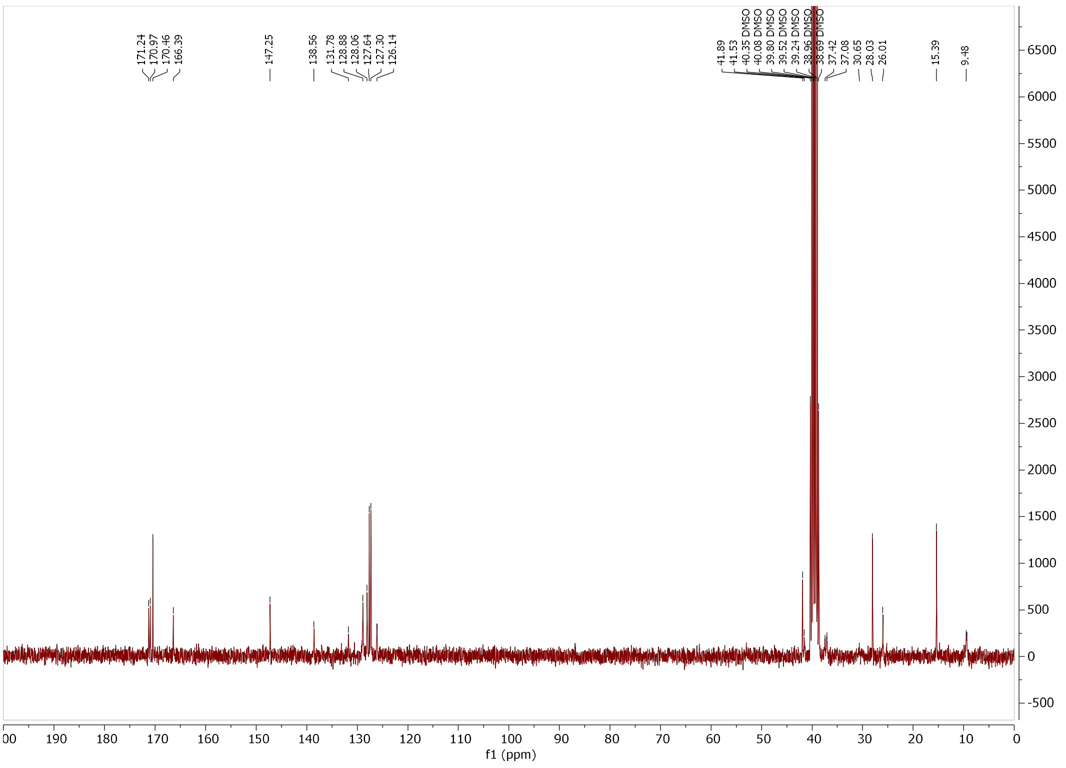

171.24
170.97
170.46
166.39
147.25
138.56
131.78
128.88
128.06
127.64
127.30
126.14
41.89
41.53
40.36 DMSO
40.08 DMSO
39.80 DMSO
39.52 DMSO
39.24 DMSO
38.96 DMSO
38.69 DMSO
37.42
37.08
30.65
28.03
26.01
15.39
9.48
f1 (ppm)

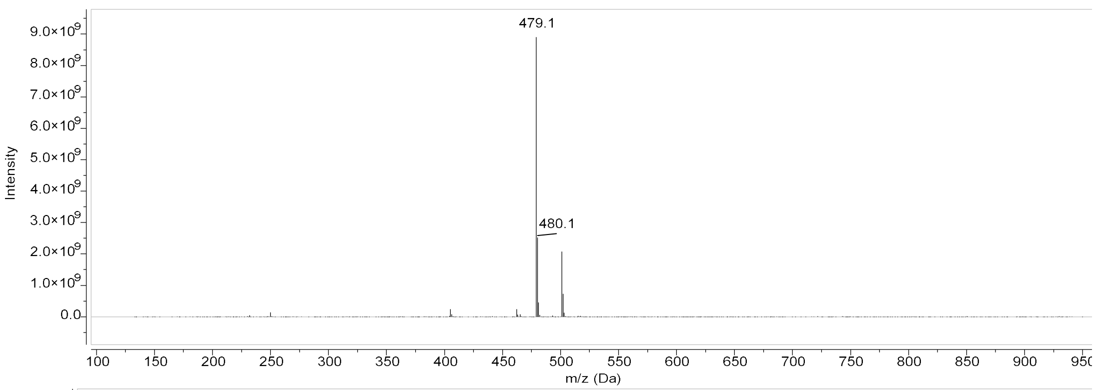

479.1
480.1
Intensity
9.0×10^9
8.0×10^9
7.0×10^9
6.0×10^9
5.0×10^9
4.0×10^9
3.0×10^9
2.0×10^9
1.0×10^9
0.0
100 150 200 250 300 350 400 450 500 550 600 650 700 750 800 850 900 950
m/z (Da)

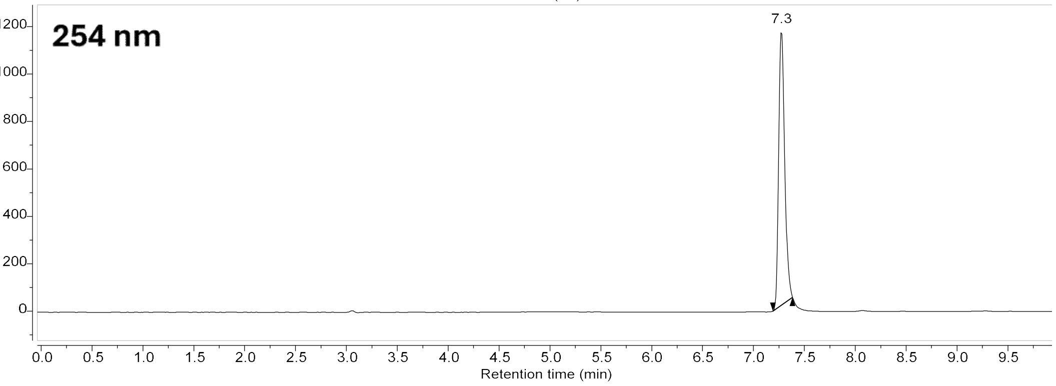

254 nm
7.3
1200
1000
800
600
400
200
0
0.0 0.5 1.0 1.5 2.0 2.5 3.0 3.5 4.0 4.5 5.0 5.5 6.0 6.5 7.0 7.5 8.0 8.5 9.0 9.5
Retention time (min)

Compound 47:

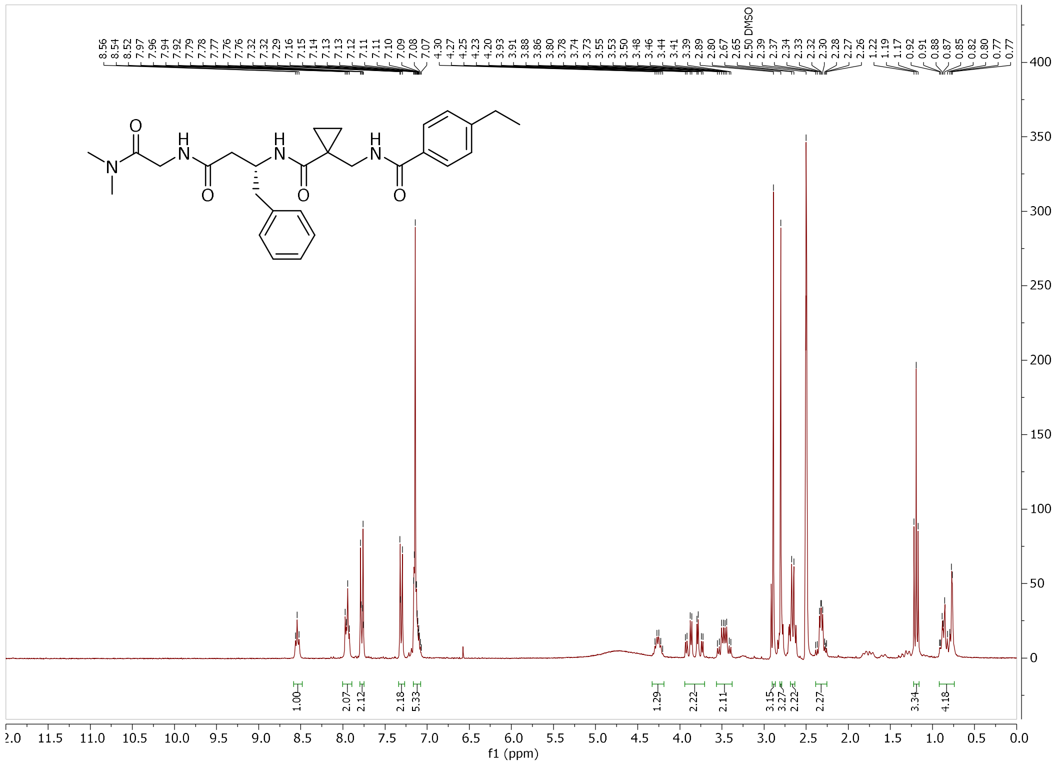
8.56 8.54 8.52 7.97 7.96 7.94 7.92 7.79 7.78 7.77 7.76 7.76 7.32 7.32 7.29 7.16 7.15 7.14 7.13 7.13 7.12 7.11 7.11 7.10 7.09 7.08 7.07 4.30 4.27 4.25 4.23 4.20 3.93 3.91 3.88 3.86 3.80 3.78 3.74 3.73 3.55 3.53 3.50 3.48 3.46 3.44 3.41 3.39 2.89 2.80 2.67 2.65 2.50 DMSO 2.39 2.37 2.34 2.33 2.32 2.30 2.28 2.27 2.26 1.22 1.19 1.17 0.92 0.91 0.88 0.87 0.85 0.82 0.80 0.77 0.77
O
N
H
N
H
N
H
N
O
O
O
1.00 2.07 2.12 2.18 5.33 1.29 2.22 2.11 3.15 3.27 2.22 2.27 3.34 4.18
f1 (ppm)

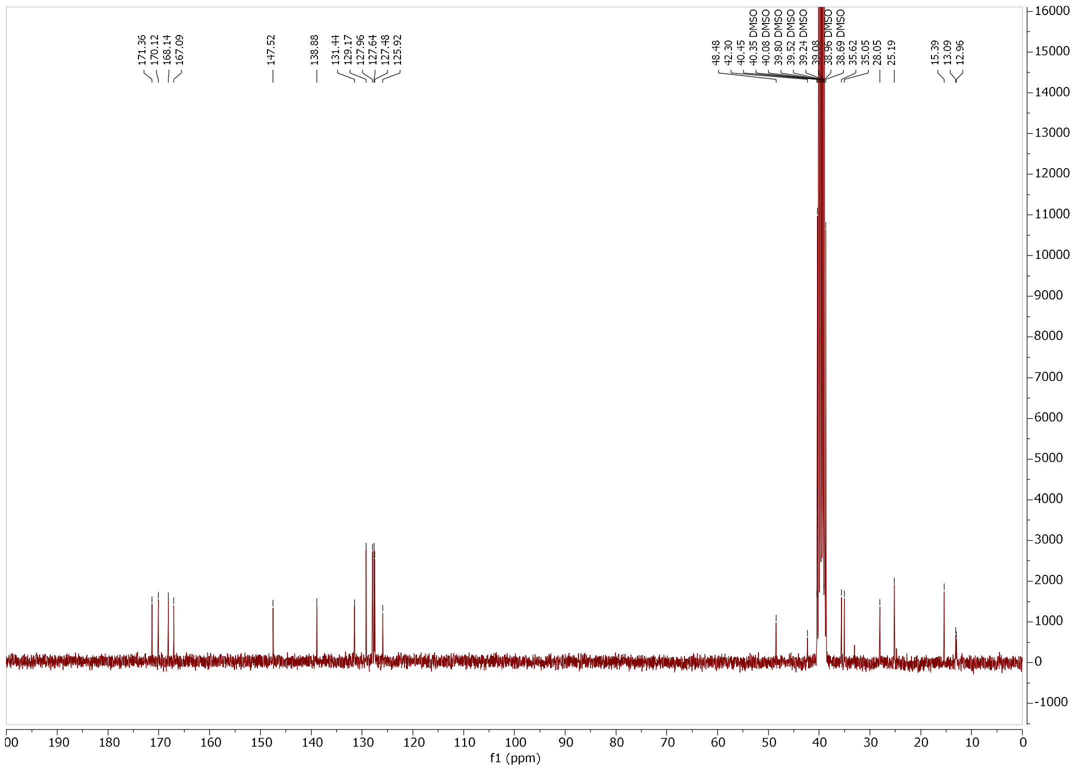
171.36 170.12 168.14 167.09 147.52 138.88 131.44 129.17 127.96 127.64 127.48 125.92
48.48 42.30 40.45 40.35 DMSO 40.08 DMSO 39.80 DMSO 39.52 DMSO 39.24 DMSO 38.96 DMSO 38.69 DMSO 35.62 35.05 28.05 25.19 15.39 13.09 12.96
f1 (ppm)

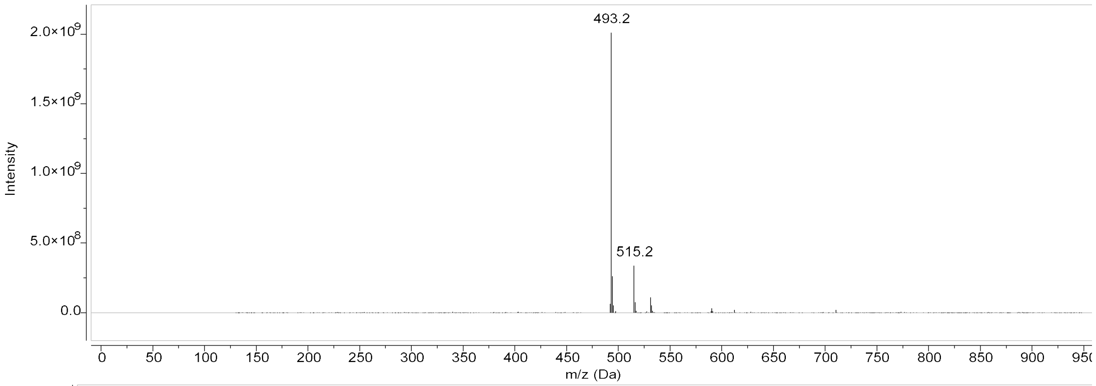
493.2
515.2
Intensity
2.0×10^9
1.5×10^9
1.0×10^9
5.0×10^8
0.0
m/z (Da)

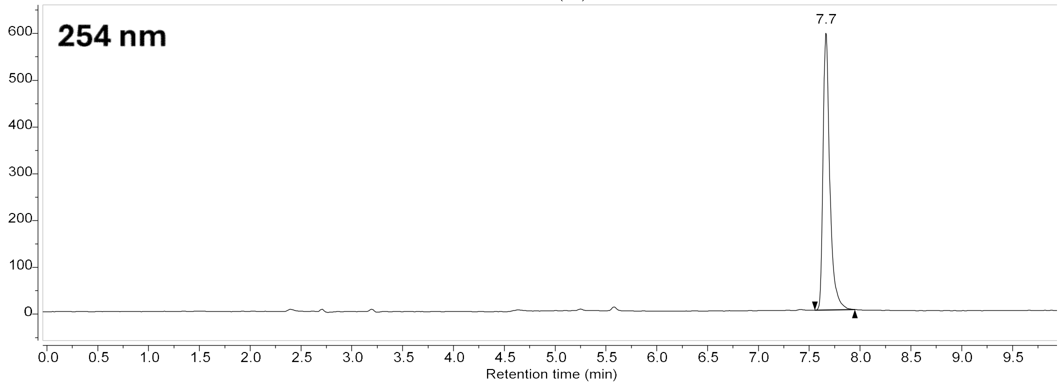
254 nm
7.7
Retention time (min)

Compound 46:

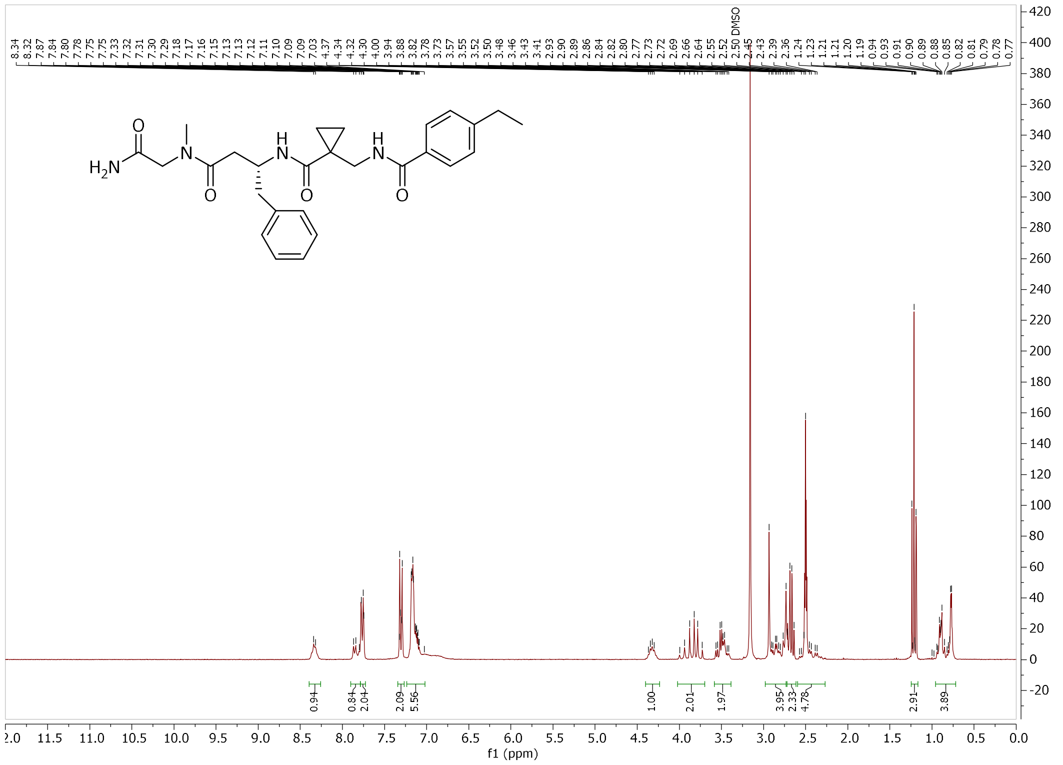

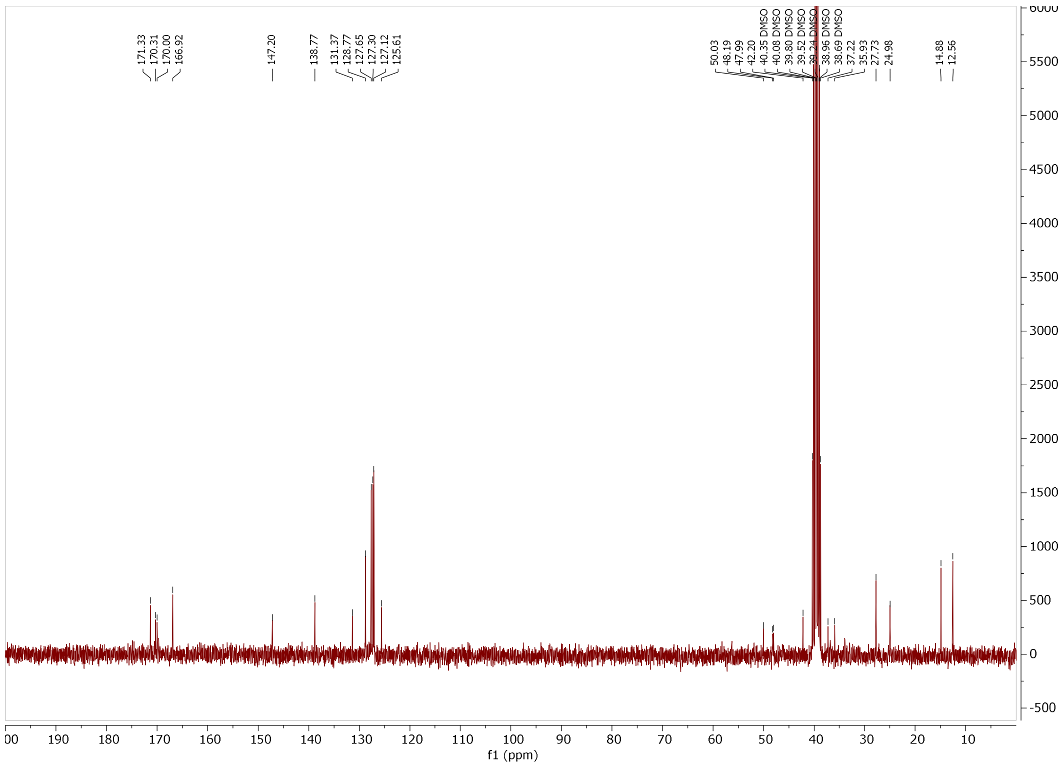

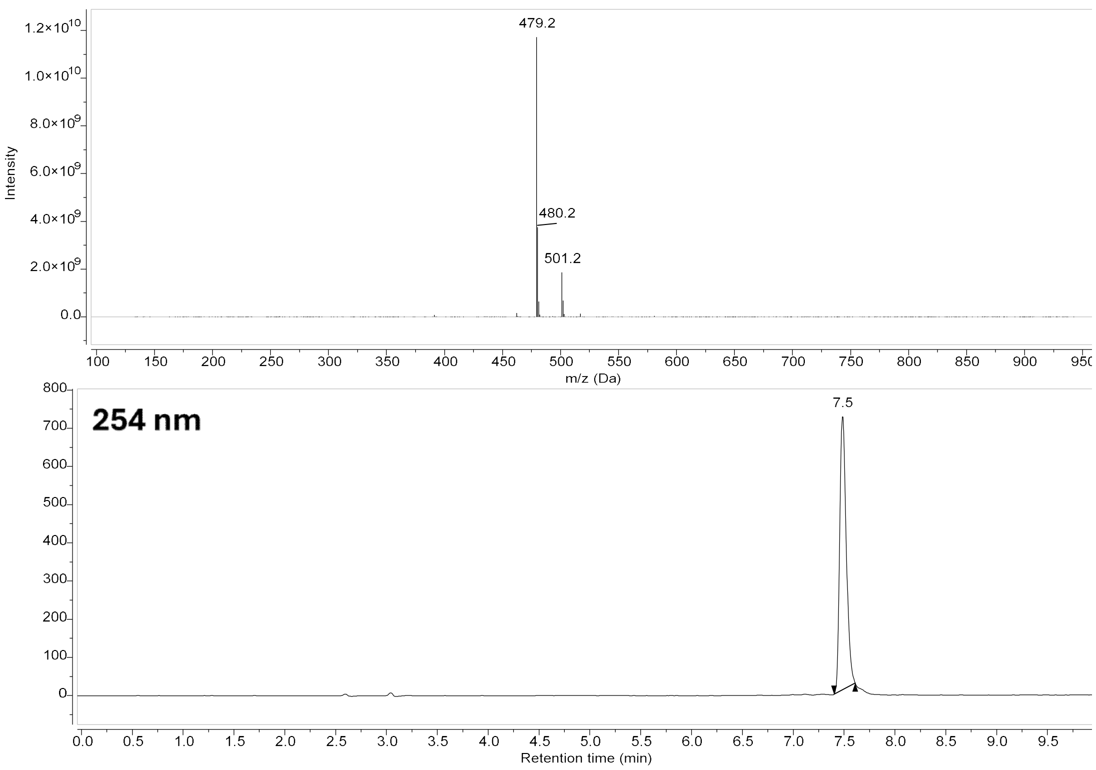

479.2
480.2
501.2
Intensity
m/z (Da)
254 nm
7.5
Retention time (min)

Compound 44:

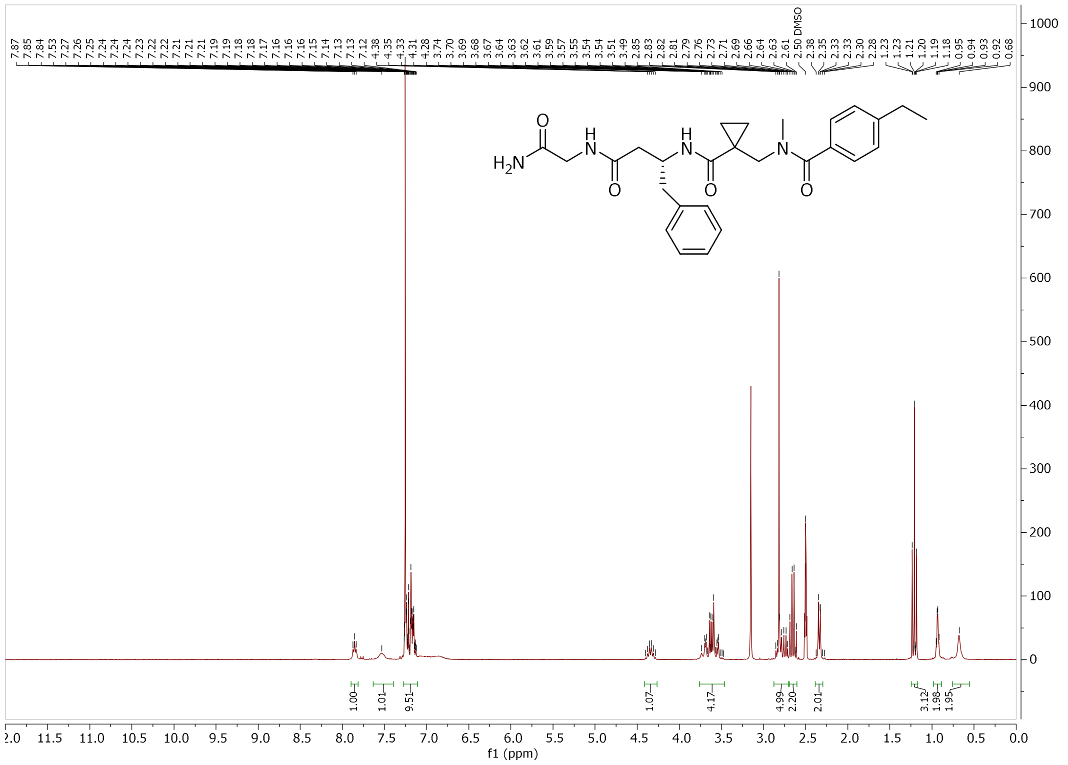
7.87
7.85
7.84
7.53
7.27
7.26
7.25
7.24
7.24
7.24
7.23
7.22
7.22
7.21
7.21
7.21
7.19
7.19
7.18
7.18
7.17
7.16
7.16
7.16
7.15
7.14
7.13
7.13
7.12
4.38
4.35
4.33
4.31
4.28
3.74
3.70
3.69
3.68
3.67
3.64
3.63
3.62
3.61
3.59
3.57
3.55
3.54
3.54
3.51
3.49
2.85
2.83
2.82
2.81
2.79
2.76
2.73
2.71
2.69
2.66
2.64
2.63
2.61
2.50 DMSO
2.38
2.35
2.33
2.33
2.30
2.28
1.23
1.23
1.21
1.20
1.19
1.18
0.95
0.94
0.93
0.92
0.68
H
H
O
H2N
N
N
N
O
O
O
1.00
1.01
9.51
1.07
4.17
4.99
2.20
2.01
3.12
1.98
1.95
f1 (ppm)

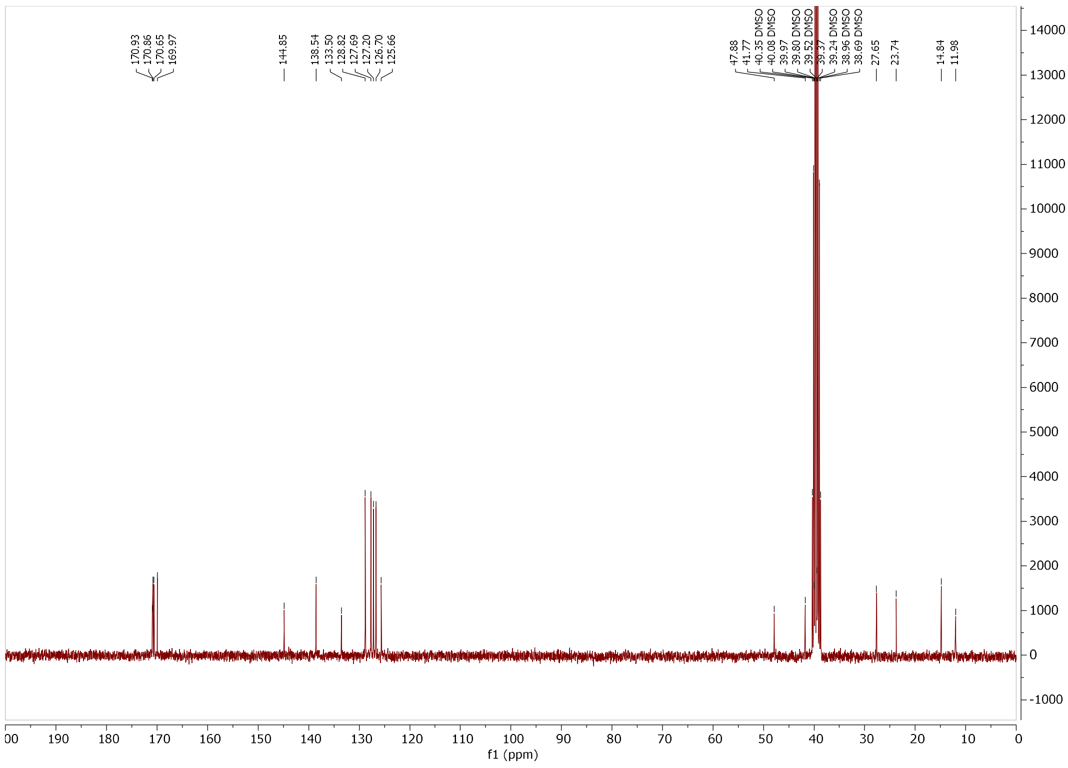
170.93
170.86
170.65
169.97
144.85
138.54
133.50
128.82
127.69
127.20
126.70
125.66
47.88
41.77
40.35 DMSO
40.08 DMSO
39.97
39.80 DMSO
39.52 DMSO
39.37
39.24 DMSO
38.96 DMSO
38.69 DMSO
27.65
23.74
14.84
11.98
f1 (ppm)

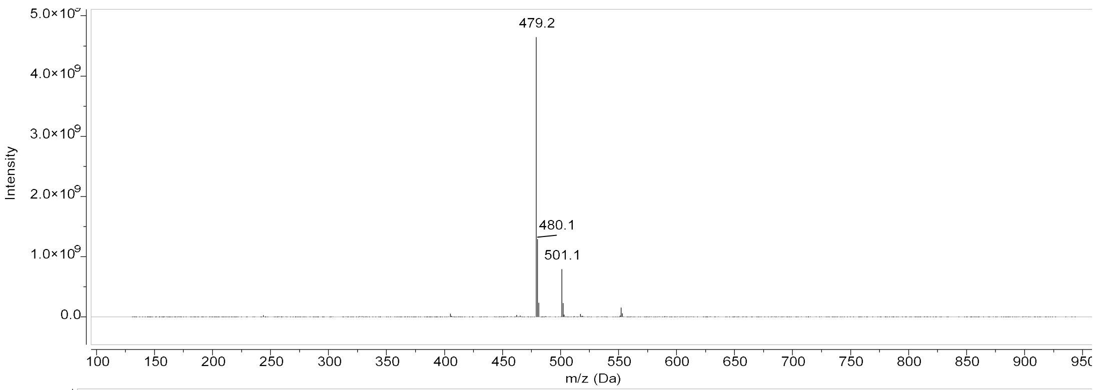

5.0×10⁹
4.0×10⁹
3.0×10⁹
2.0×10⁹
1.0×10⁹
0.0
Intensity
479.2
480.1
501.1
100 150 200 250 300 350 400 450 500 550 600 650 700 750 800 850 900 950
m/z (Da)

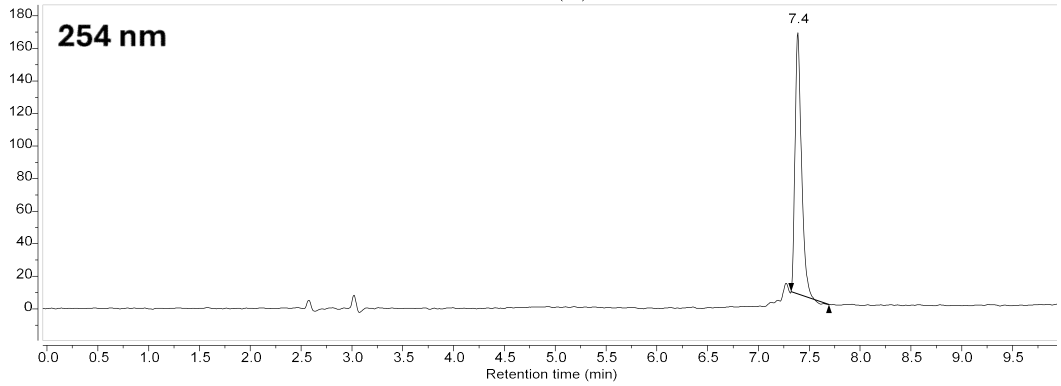

254 nm
7.4
180 160 140 120 100 80 60 40 20 0
0.0 0.5 1.0 1.5 2.0 2.5 3.0 3.5 4.0 4.5 5.0 5.5 6.0 6.5 7.0 7.5 8.0 8.5 9.0 9.5
Retention time (min)

Compound 9:

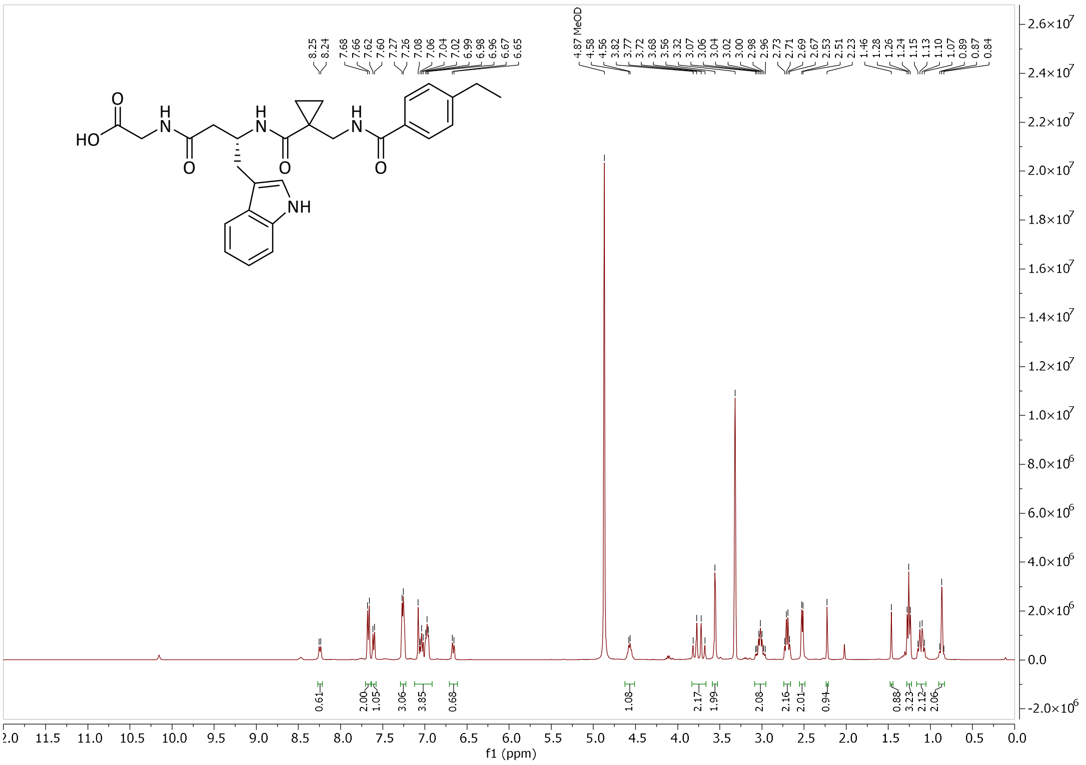

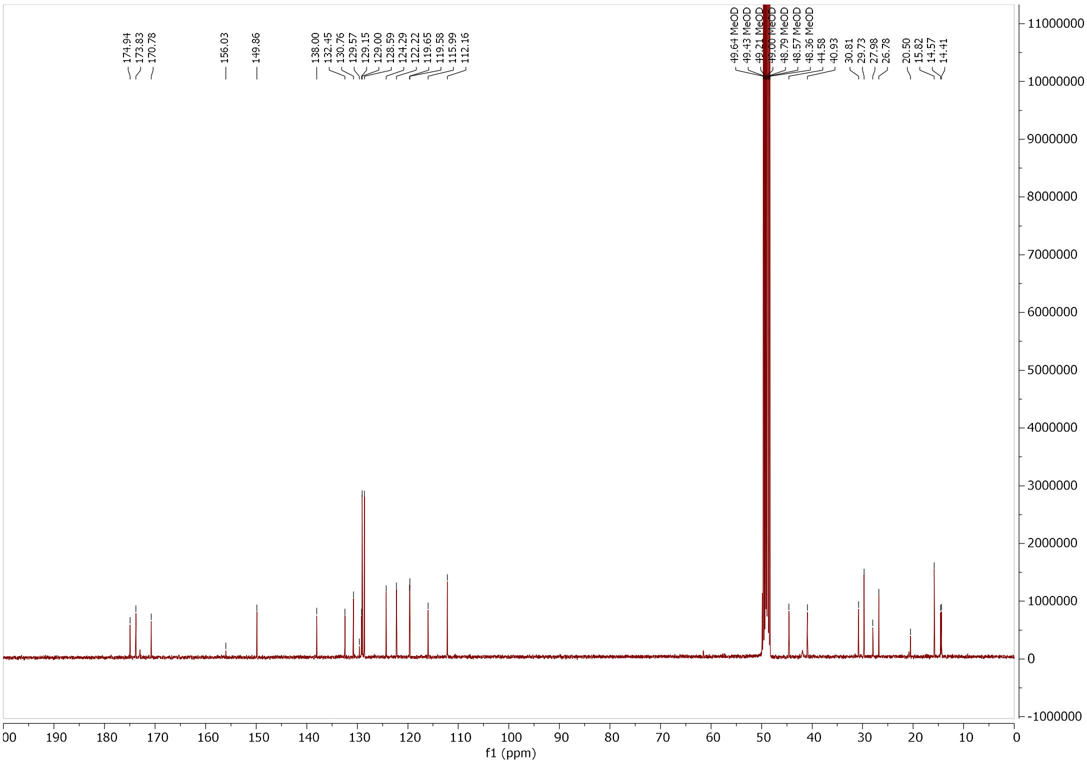

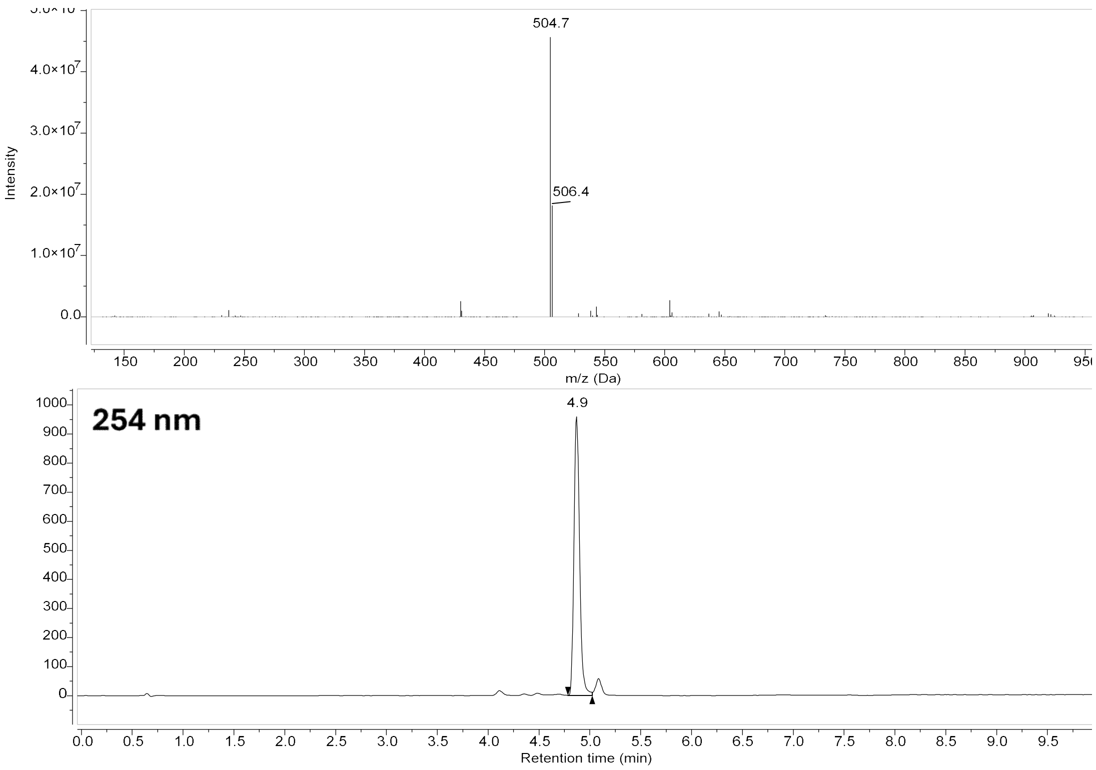
504.7
506.4
Intensity
m/z (Da)
254 nm
4.9
Retention time (min)

Compound 48:

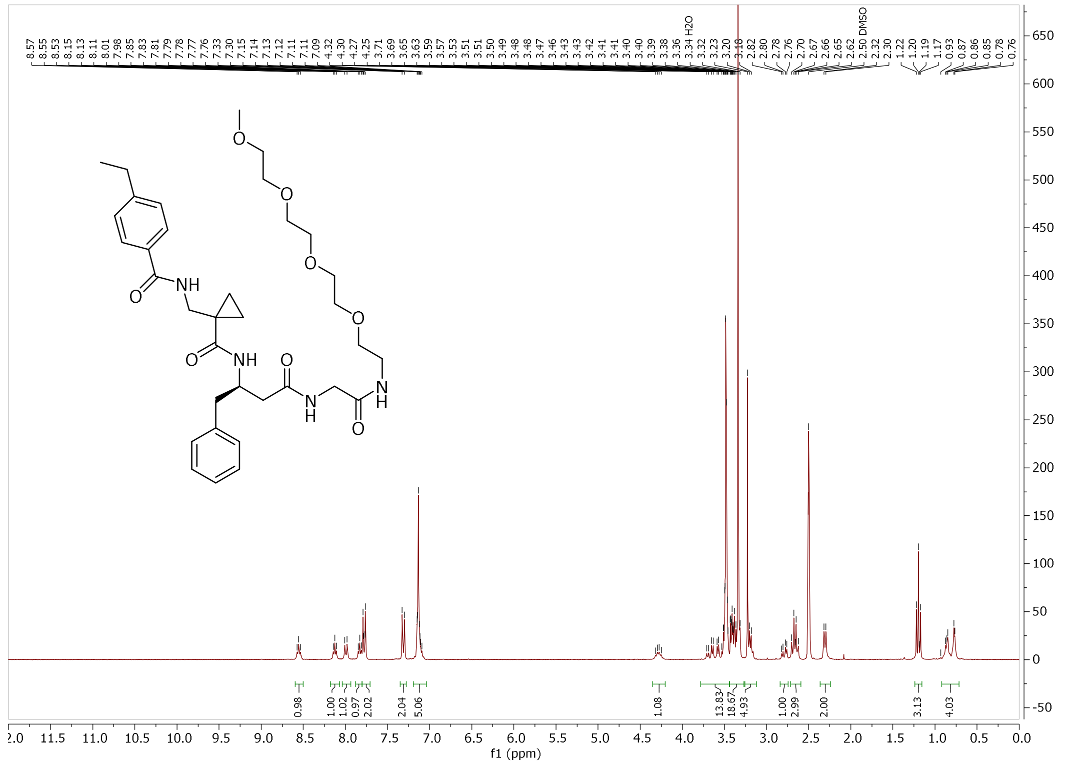

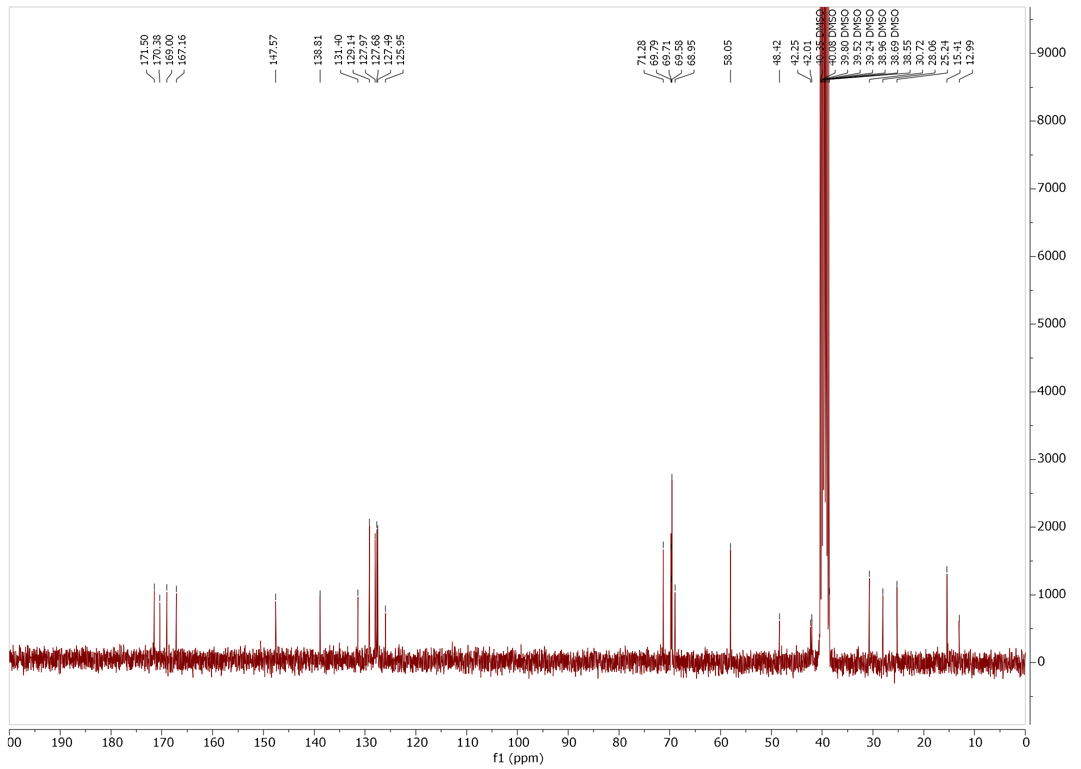

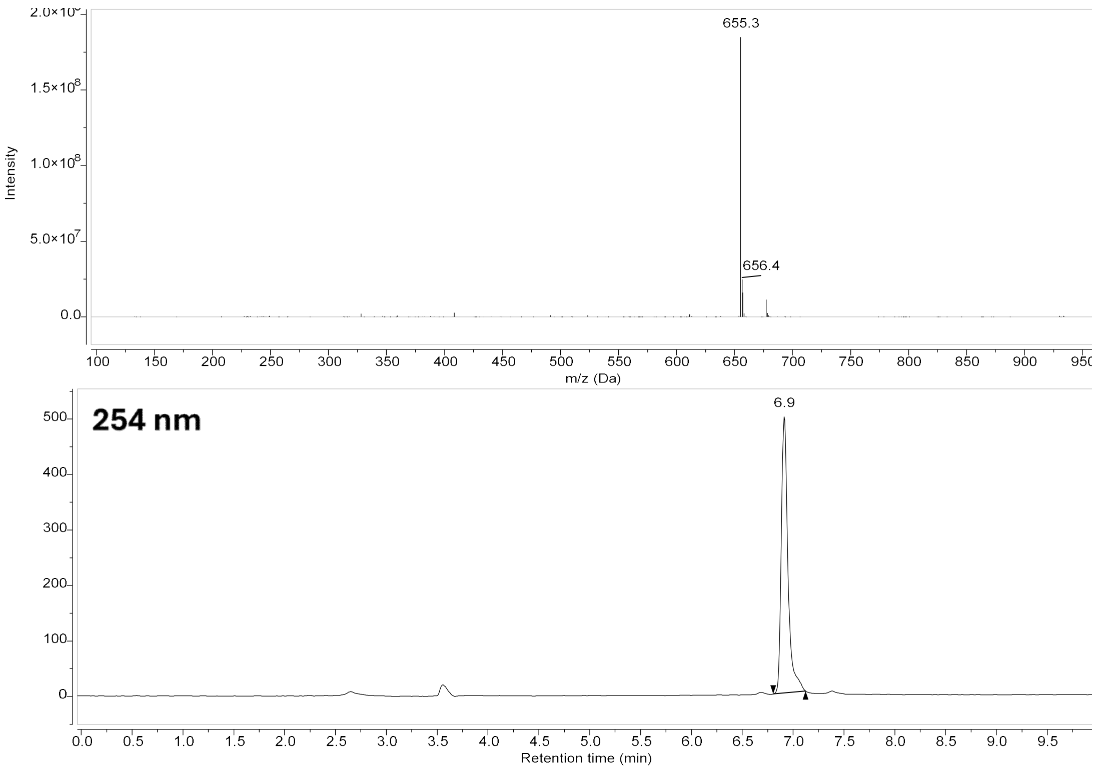

655.3
656.4
Intensity
m/z (Da)
254 nm
6.9
Retention time (min)

Compound 7:

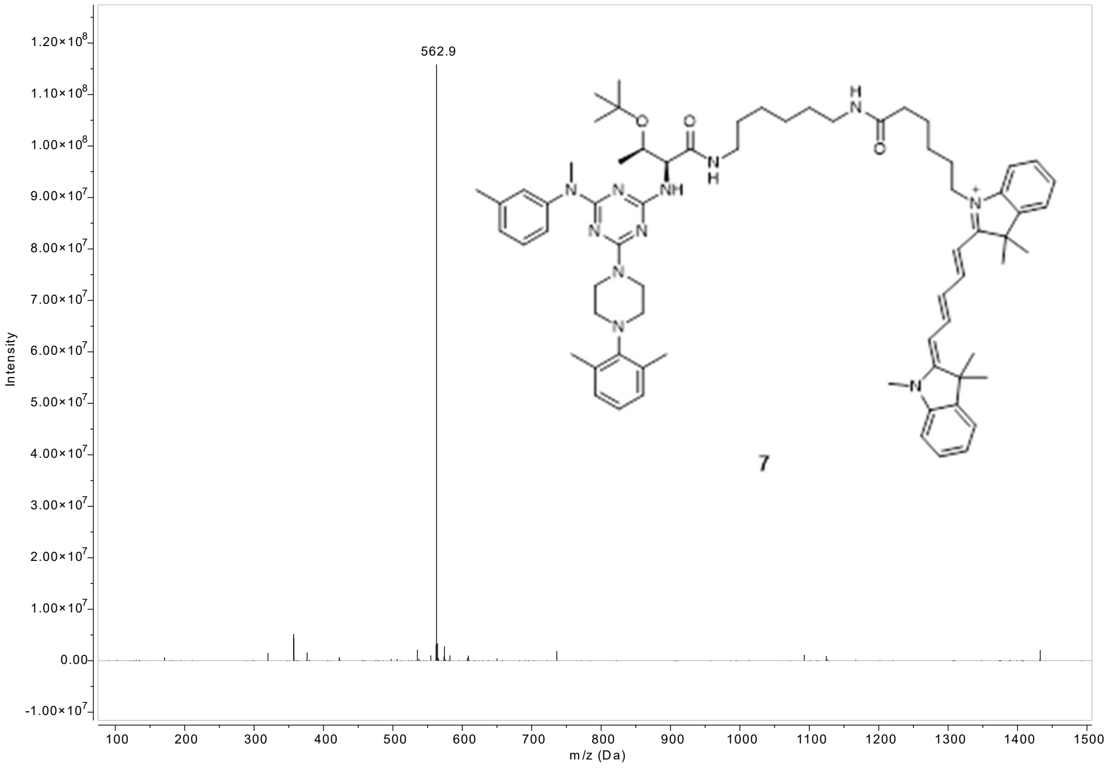

Compound 6:

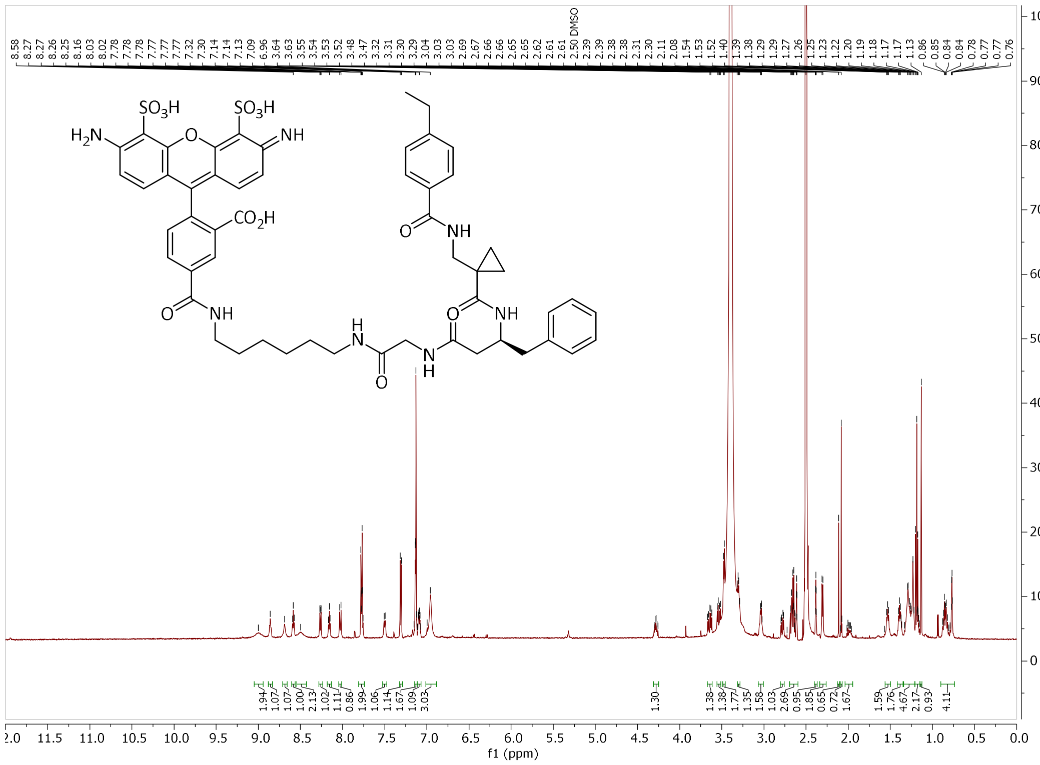

f1 (ppm)

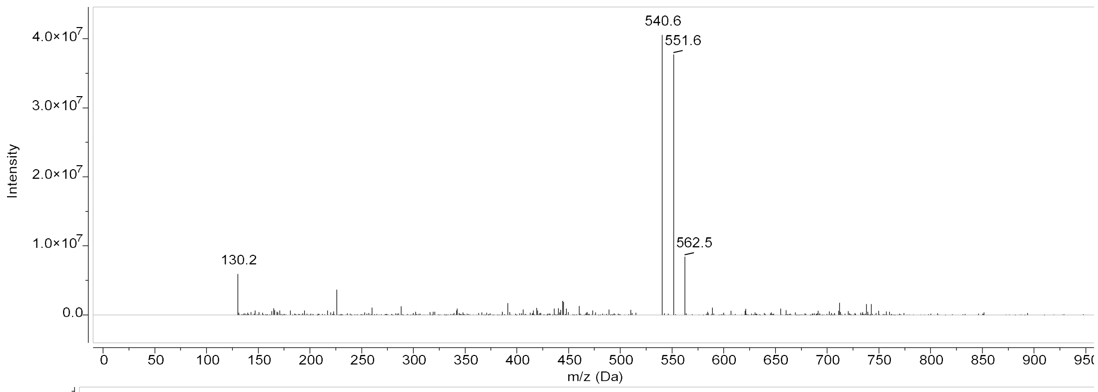

540.6
551.6
562.5
130.2
Intensity
m/z (Da)

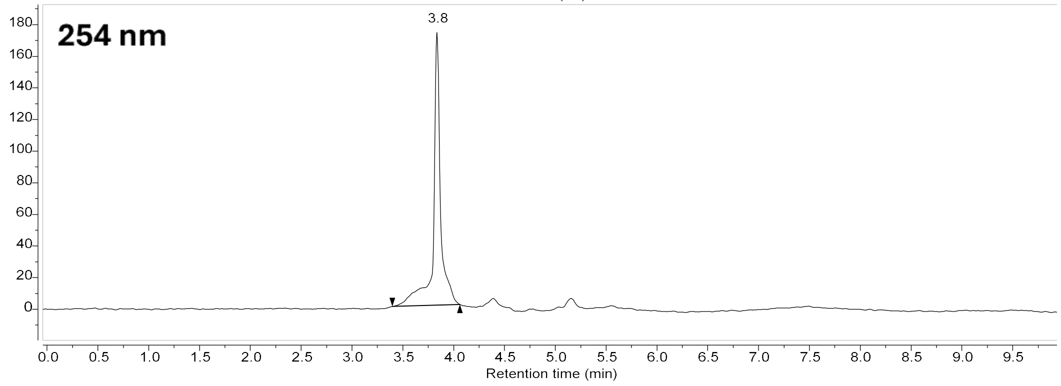

254 nm
3.8
Retention time (min)

Compound 8:

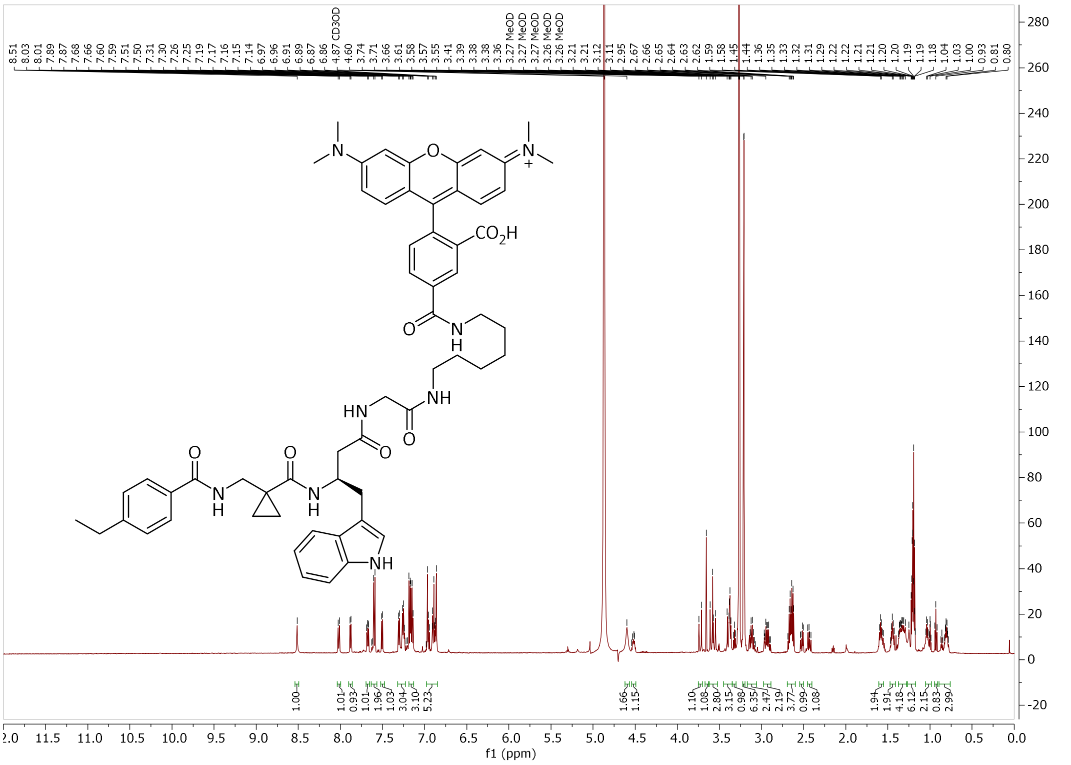
8.51 8.03 8.01 7.89 7.87 7.68 7.66 7.60 7.59 7.51 7.50 7.31 7.30 7.26 7.25 7.19 7.17 7.16 7.15 7.14 6.97 6.96 6.91 6.89 6.87 6.86 4.87 CD3OD 4.60 3.74 3.71 3.66 3.61 3.58 3.57 3.55 3.41 3.39 3.38 3.36 3.27 MeOD 3.27 MeOD 3.27 MeOD 3.26 MeOD 3.26 MeOD 3.21 3.21 3.12 3.11 2.95 2.67 2.66 2.65 2.64 2.63 2.62 1.59 1.58 1.45 1.44 1.36 1.35 1.33 1.32 1.31 1.29 1.22 1.22 1.21 1.21 1.20 1.20 1.19 1.19 1.18 1.04 1.03 1.00 0.93 0.81 0.80
N
O
N
+
CO_2H
O
N
H
NH
HN
O
O
O
O
N
H
N
H
NH
f1 (ppm)

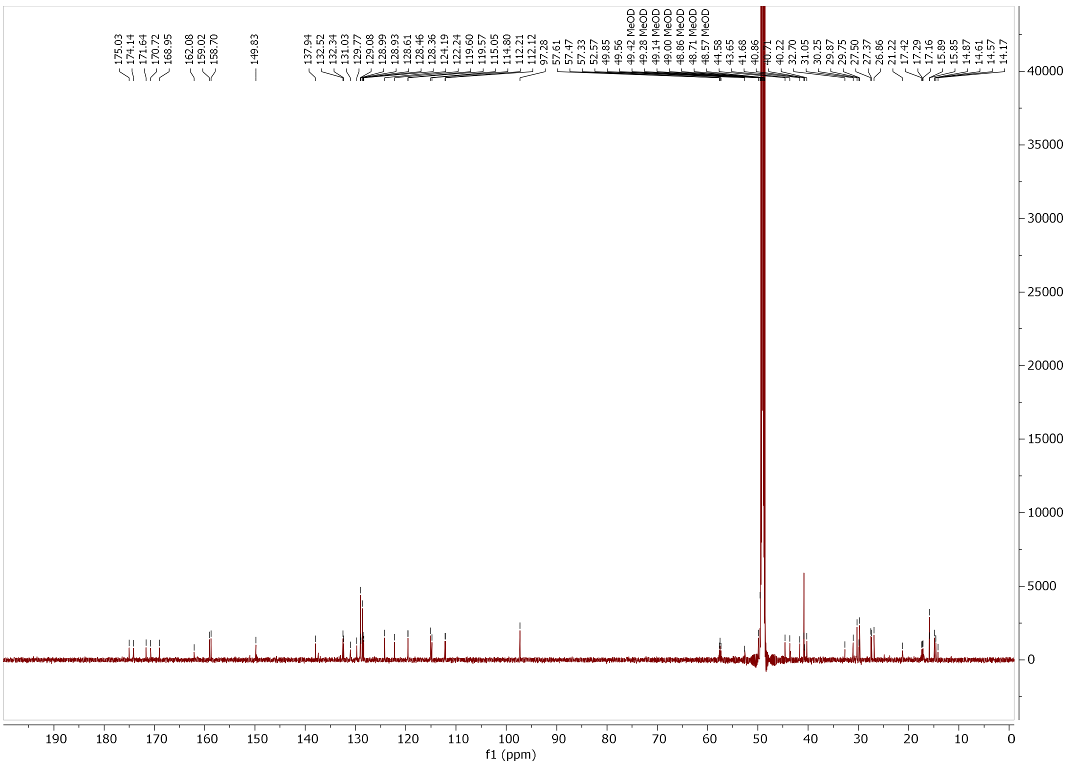
175.03 174.14 171.64 170.72 168.95 162.08 159.02 158.70 149.83 137.94 132.52 132.34 131.03 129.77 129.08 128.99 128.93 128.61 128.46 128.36 124.19 122.24 119.60 119.57 115.05 114.80 112.21 112.12 97.28 57.61 57.47 57.33 52.57 49.85 49.56 49.42 MeOD 49.28 MeOD 49.14 MeOD 49.00 MeOD 48.86 MeOD 48.71 MeOD 48.57 MeOD 44.58 43.65 41.68 40.86 40.71 40.22 32.70 31.05 30.25 29.87 29.75 27.50 27.37 26.86 21.22 17.42 17.29 17.16 15.89 15.85 14.87 14.61 14.57 14.17
f1 (ppm)

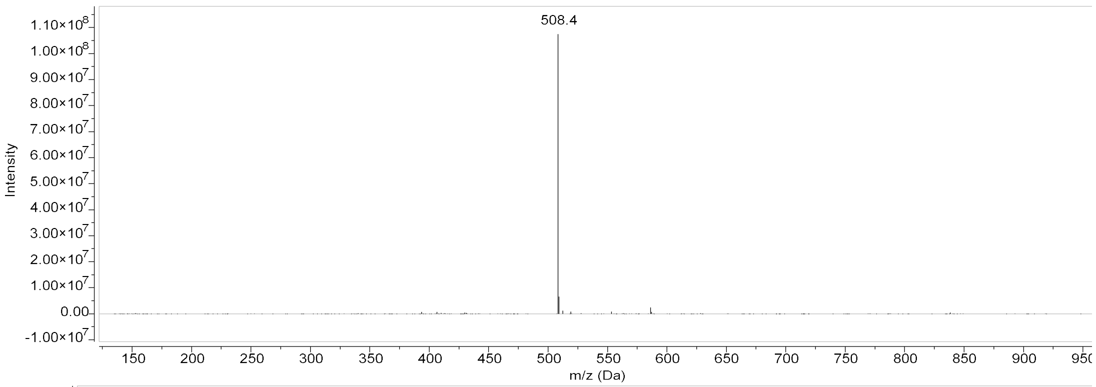
508.4
Intensity
1.10×10^8
1.00×10^8
9.00×10^7
8.00×10^7
7.00×10^7
6.00×10^7
5.00×10^7
4.00×10^7
3.00×10^7
2.00×10^7
1.00×10^7
0.00
-1.00×10^7
150 200 250 300 350 400 450 500 550 600 650 700 750 800 850 900 950
m/z (Da)

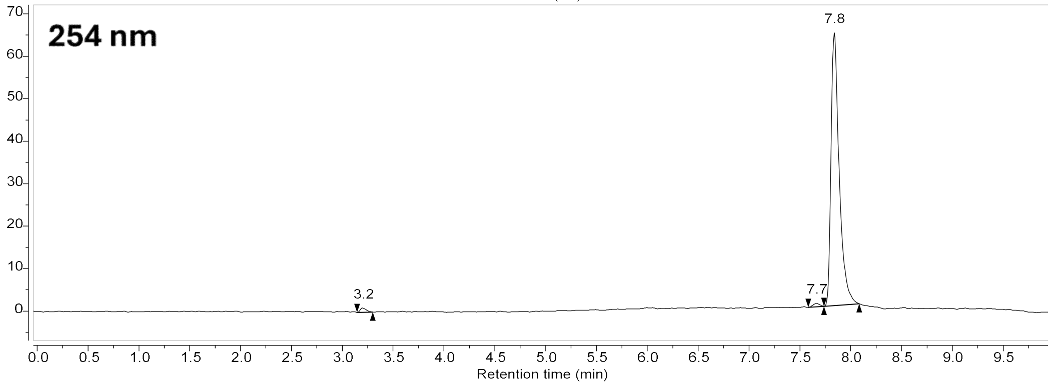
254 nm
7.8
3.2
7.7
70
60
50
40
30
20
10
0
0.0 0.5 1.0 1.5 2.0 2.5 3.0 3.5 4.0 4.5 5.0 5.5 6.0 6.5 7.0 7.5 8.0 8.5 9.0 9.5
Retention time (min)
